# Global, regional, and national burden and risk factors of ischemic heart disease, 1990–2021: an analysis of the global burden of disease study

**DOI:** 10.3389/fpubh.2025.1563631

**Published:** 2025-04-25

**Authors:** Quankai Cheng, Sheng Zhou, Haicheng Zhong, Ziming Wang, Chang Liu, Jingjing Sun, Jie Deng

**Affiliations:** ^1^Department of Cardiology, The Second Affiliated Hospital of Xi’an Jiaotong University, Xi’an, Shaanxi, China; ^2^Department of Respiratory and Critical Care Medicine, The Second Affiliated Hospital of Xi’an Jiaotong University, Xi’an, Shaanxi, China

**Keywords:** ischemic heart disease, global burden of disease, risk factors, decomposition analysis, forecast

## Abstract

**Background:**

With a rapidly growing and aging world population, ischemic heart disease (IHD) remains a major burden. This study aimed to reassess the prevalence trend of IHD from 1990 to 2021 from multiple dimensions to improve the shortcomings of the existing studies and provide a solid scientific basis for policymakers.

**Methods:**

This study extracted data on the prevalence, incidence, mortality, disability-adjusted life years (DALYs), and associated risk factors of IHD from the global burden of disease (GBD) 2021 study. Descriptive, decomposition, and risk factor analyses were used to provide insights into the epidemiologic patterns of IHD from 1990 to 2021 and project the burden of IHD from 2022 to 2045. Potential differences in burden and risk factors based on age, sex, 21 GBD geographic regions, five social development index (SDI) regions, and 204 countries are highlighted.

**Results:**

Globally, the age-standardized prevalence rate (ASPR) of IHD is increasing, while the age-standardized incidence rate (ASIR), age-standardized mortality rate (ASMR), and age-standardized disability-adjusted life years (ASDR) are decreasing. ASPR, ASIR, ASMR, and ASDR were highest in the low-middle SDI regions and lowest in the high SDI regions. ASMR and ASDR were highest in Nauru and lowest in Portugal. Men had an overall heavier burden of IHD than women; the 65–69 age group had the largest burden, and those aged >95 years had the highest crude incidence rate. In addition, the burden of IHD was negatively correlated with SDI across regions and countries, while decomposition analyses suggest that the main reasons for the current increase in the burden of IHD are aging and population growth. Risk factors have changed relatively little over the 32 years, with metabolic risk still ranking first. We forecast that the absolute burden of IHD will continue to increase till 2045; however, ASIR, ASMR, and ASDR will gradually decline.

**Conclusion:**

From 1990 to 2021, the global burden of IHD generally increased and varied across regions, sex, and age groups. Due to increasing population growth and aging, there is an urgent need for strategically directed measures to reduce the burden of IHD.

## 1 Introduction

Ischemic heart disease (IHD) is one of the major causes of death and morbidity worldwide. IHD has high prevalence and mortality rates, with significant long-term effects on quality of life, highlighting its status as a serious public health challenge (1). Approximately 9.13 million IHD-related deaths and 180 million disability-adjusted life years (DALYs) were recorded in the 2019 Global Burden of Disease (GBD) research (2). Although IHD-related death rates have decreased in a number of high-income countries due to advancements in medical technology and treatment strategies, notable discrepancies still exist, particularly in low- and middle-income countries (3–5). IHD is mostly caused by coronary atherosclerosis-induced myocardial ischemia, and its main risk factors include smoking, diabetes mellitus, hypertension, and hyperlipidemia. Global data reveal that as the prevalence of these risk factors changes, disparities in the burden of IHD across regions become more pronounced (4). For instance, in sub-Saharan Africa and South Asia, the IHD-related mortality rate is significantly higher than the global average (6). Furthermore, the increasing incidence of IHD is linked to global demographic aging. In countries with an aging population, such as Japan, although the IHD-related mortality rate has been reduced through aggressive cardiovascular management, the high prevalence of IHD has led to increased long-term healthcare needs and economic burden (7–9).

To address the global health crisis posed by IHD, among the 2030 Sustainable Development Goals, the World Health Organization has designated lowering the death rate due to cardiovascular disease as a primary goal (10), highlighting the need for more robust public health initiatives and strategies in low- and middle-income countries. The global epidemiological status of IHD has been studied in the past using data from the 2019 GBD database, revealing declines in IHD age-standardized prevalence rate (ASPR), age-standardized incidence rate (ASIR), age-standardized mortality rate (ASMR), and age-standardized disability-adjusted life years (ASDR) except in low social development index (LSDI) and middle SDI (MSDI) regions (11). However, this overall improvement masks significant differences between countries. A previous study showed that countries with higher SDI instead bear a greater burden of CVD, which calls for high-income countries to prioritize the introduction of effective management strategies, while low- and middle-income countries need to strengthen early prevention and diagnosis (12). At the same time, this disparity is more pronounced in specific populations. Hu et al. revealed significant geographic differences in IHD morbidity and mortality among women of childbearing age, calling for governments to strengthen early detection and improve healthcare services (13). More grimly, Shi et al. analyzed the 2021 GBD data and predicted that IHD will remain a major public health challenge till 2050 (14, 15). The cumulative evidence indicates the ongoing severity of IHD burden worldwide, highlighting the critical need for region-specific control strategies and revised burden estimation.

This study summarizes the changes in the absolute burden, ASPR, ASIR, ASMR, and ASDR of IHD from 1990 to 2021 based on statistics from the 2021 GBD database stratified by SDI, age, and sex. Similarly, insights into the aging, demographically and epidemiologically adjusted burden of IHD are enhanced to further assess the burden and trends of IHD. The study aimed to provide a scientific basis for the development of effective disease prevention strategies and health interventions to mitigate the adverse impacts of IHD on global health and the economy.

## 2 Methods

### 2.1 Data sources

This study evaluated health losses using the GBD 2021 dataset (16). Data were sourced from the Global Health Data Exchange (GHDx),^1^ spanning 204 countries and regions, ensuring a broad and representative sample size (17). For transparency and trustworthiness, this study complied with the GATHER principles and was approved by the University of Washington’s Institutional Review Board with a waiver for informed consent (18, 19).

### 2.2 Study overview

In this study, we extracted estimates of IHD incidence, prevalence, mortality, and DALYs for the global population and 204 countries and regions across all age groups from the GBD 2021 dataset, including 95% uncertainty intervals (UIs). We also obtained corresponding IHD data for five SDI and 21 GBD regions. Additionally, we gathered estimates of DALYs associated with IHD risk factors for the global population, the five SDI regions, and the 21 GBD regions.

### 2.3 Relevant definitions

#### 2.3.1 Prevalence, incidence, mortality, and DALYs

Estimates of IHD prevalence, incidence, mortality, and DALYs rely on the integration of various data sources and advanced modeling techniques. Data extrapolations were performed using a spatiotemporal Gaussian process regression model (ST-GPR) and Disease Modeling Bayesian Meta-Regression version 2.1 (DisMod-MR 2.1). The model generates internally consistent estimates of prevalence, morbidity, and mortality based on age, sex, location, and year while ensuring consistency between these different health indicators (20). In addition, the model employs a cause-of-death ensemble model that incorporates vital registration, cause-of-death determination, and IHD surveillance data to estimate mortality rates and years of life lost (YLLs) (8, 18). The years lost to disability (YLDs) were calculated by multiplying the estimated morbidity rates by the corresponding disability weights, while the DALYs represent the sum of the YLDs and YLLs due to disability (21).

#### 2.3.2 SDI

The SDI is a composite indicator that assesses social and demographic development, summarizing the overall status of income, education, and fertility levels in a given country or region and is assessed by dividing the global population into five quintiles: low SDI (LSDI), low-middle SDI (LMSDI), middle SDI (MSDI), high-middle SDI (HMSDI) and high SDI (HSDI) regions (22, 23).

#### 2.3.3 Twenty-one GBD geographic regions

The GBD study divides the globe into 21 major regions based on epidemiologic similarities and geographic proximity, including Andean Latin America, Oceania, Caribbean, Central Asia, Central Europe, Central Latin America, Central Sub-Saharan Africa, East Asia, Eastern Europe, Eastern Sub-Saharan Africa, High-Income Asia-Pacific, High-Income North America, North Africa and the Middle East, Oceania, South Asia, South-East Asia, Southern Latin America, Southern Sub-Saharan Africa, Tropical Latin America, Western Europe, and Western Sub-Saharan Africa (1).

#### 2.3.4 Risk factors

The GBD study identified a total of 88 risk factors by assessing specific and aggregate risk factors at the global, regional, and national levels in 204 countries and territories. The risk factors were grouped into four tiers: Tier 1 included behavioral, environmental, metabolic, and occupational factors; Tier 2 included 20 individual risk factors or groups of risk factors; Tier 3 included 52 individual risk factors or groups of risk factors; and Tier 4 included 69 specific risk factors. Among these, DisMod-MR 2.1 and ST-GPR were used to quantify the impact of these risk factors on the burden of disease, allowing us to analyze the distribution of the exposure to the risk factors in different populations and regions (8).

### 2.4 Statistical analysis

#### 2.4.1 Descriptive analysis

In this study, the prevalence, incidence, mortality, and DALYs of IHD were descriptively analyzed and visualized at the global, regional, and national levels. Similarly, we calculated the age-standardized rate (ASR) for these indicators and used estimated annual percentage change (EAPC) to assess trends from 1990 to 2021. In addition, at the level of the five SDI regions, we compared differences in burden by year, age, and sex. Moreover, at the 21 GBD regions and national levels, the relationship between ASR and SDI was demonstrated using Spearman correlation analysis (24).

The ASR was calculated as follows: $ASR=\frac{\overset{n}{\underset{i=1}{\sum }}\left(r_{i}w_{i}\right)}{\overset{n}{\underset{i=1}{\sum }}w_{i}}$×100,000. ($r_{i}$: the specific rate for the i age groups such as prevalence, incidence, or mortality rates; $w_{i}$: the standard population weight for the i age group; n: the total number of age groups).

The EAPC is calculated based on a linear regression model and is an age-standardized ratio that describes changes over time and can be used as a reliable indicator to monitor changes in disease patterns (25). It was calculated as follows: $\mathrm{EAPC}=\left(e^{\beta }-1\right)\times 100\%$. (β: the regression coefficient derived from a linear regression model, estimated from the logarithmic, linear relationship between time and the ASR). The specific steps are as follows: (1) the natural logarithm of ASR, denoted as ln $\left(ASR\right)=\alpha$+β×t, where t represents the year, α is the constant term, and β is the regression coefficient for the time variable was taken; (2) β was substituted into the EAPC formula to calculate the annual percentage change. If the EAPC and the lower limit of its 95% CI is greater than 0, it indicates an increasing trend in the ASR; if it is less than 0, it indicates a decreasing trend in the ASR. If neither condition is met, the ASR is considered stable (26).

#### 2.4.2 Decomposition analysis

To further analyze the contribution of population growth, aging, and epidemiological changes to changes in the burden of IHD between 1990 and 2021, we grouped IHD prevalence, morbidity, mortality, DALYs, YLLs, and YLDs by region (globally and in the five SDI regions), and by sex (men and women). We disaggregated and analyzed the above six indicators. Using morbidity as an example, the decomposition analysis equation is as follows: $\mathrm{Incidence}s_{m,P,r}=\overset{k}{\underset{j=1}{\sum }}\left(b_{j,t}\times P_{t}\times f_{j,t}\right)$ (${\mathrm{Incidences}}_{m,P,r}$: the number of incidences, based on age structure, population size, and incidence rates in the yeart;$b_{j,t}$: the proportion of thej-th age group in the yeart, reflecting the share of that age group’s population within the total population;$P_{t}$: the total population in the yeart; and$f_{j,t}$: the incidence rate for thej-th age group in the yeart) (27, 28).

#### 2.4.3 Risk factor analysis

We investigated specific risk factors affecting DALYs for IHD in 1990 and 2021 in the GBD database. We ranked them in descending order of their share of the disease burden, which was further visualized using heat maps. Second, we focused on the top five risk factors: metabolic risks, behavioral risks, high systolic blood pressure, dietary risks, and environmental/occupational risks, further analyzing the ASDR data attributed to these factors and stratifying by global, five SDI regions, and 21 GBD regional districts, using bar charts to show the magnitude of the contribution of these risk factors relative to all other factors in 1990 and 2021. This approach demonstrates the contribution of the top five risk factors to IHD, providing a deeper understanding from a disease prevention and development perspective (29).

#### 2.4.4 Predictive analysis

The above analysis primarily demonstrates changes in the burden of IHD between 1990 and 2021. To further guide the development of public health strategies and the implementation of interventions, we used a Bayesian age-period-cohort (BAPC) model to project the burden of IHD globally from 2022 to 2045 stratified by sex. The BAPC model used the Integrated Nested Laplace Approximation method of the full Bayesian inference to generate age-specific and ASPR. When interested in the predictive distribution, it automatically incorporates Poisson noise, which improves the accuracy and efficiency of predictions compared with traditional APC (30–32).

R software (version 4.3.3) was used for all computations, analyses, and visualizations, adhering to the relevant research protocols. Statistical significance was defined as *p* < 0.05.

## 3 Results

### 3.1 Global trend

#### 3.1.1 Prevalence

**Figure 1:**
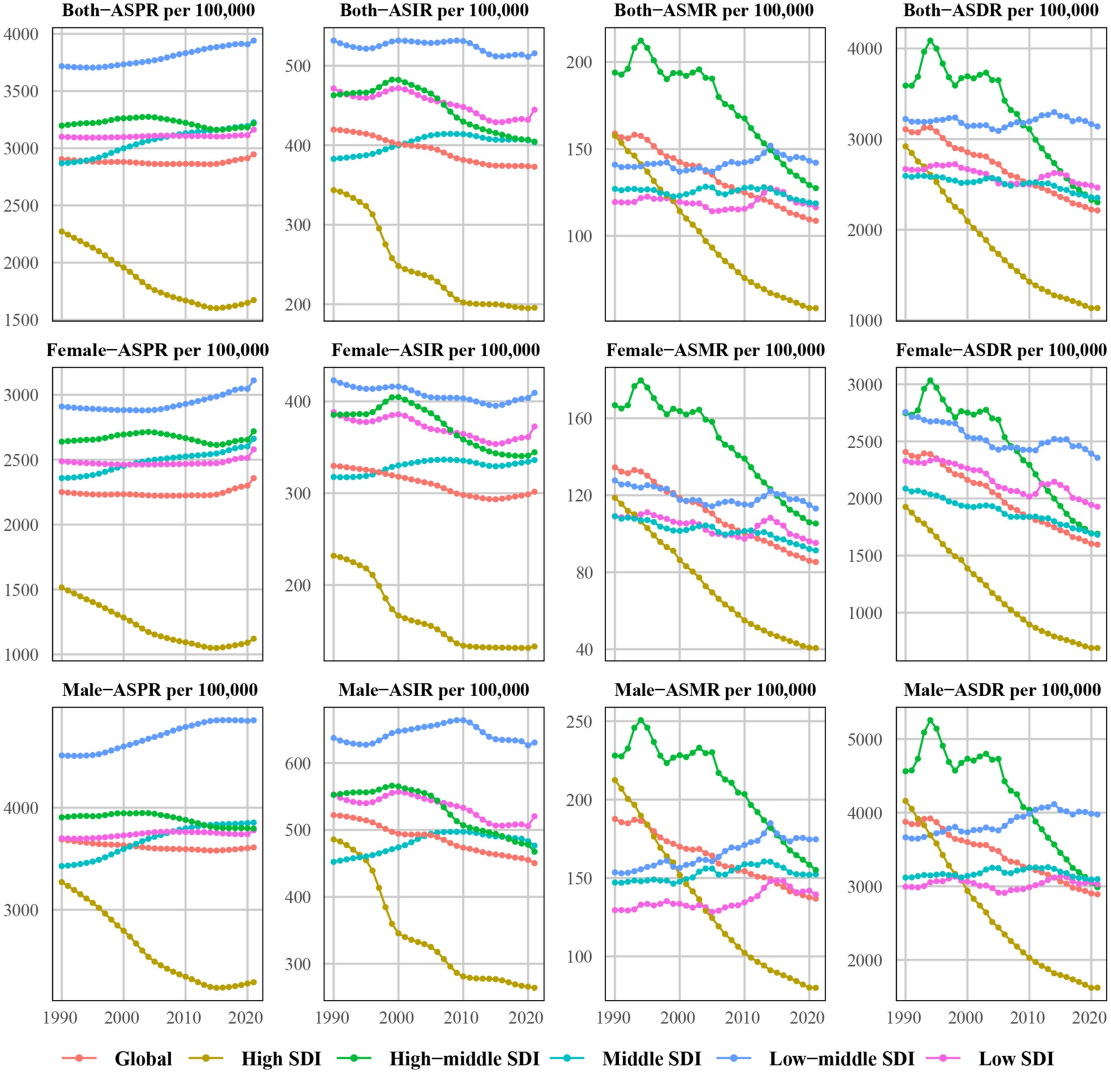
Trends in age-standardized prevalence (ASPR), age-standardized incidence (ASIR), age-standardized mortality (ASMR) and age-standardized disability-adjusted life years rate (ASDR) for ischemic heart disease from1990 to 2021.

**Figure 2:**
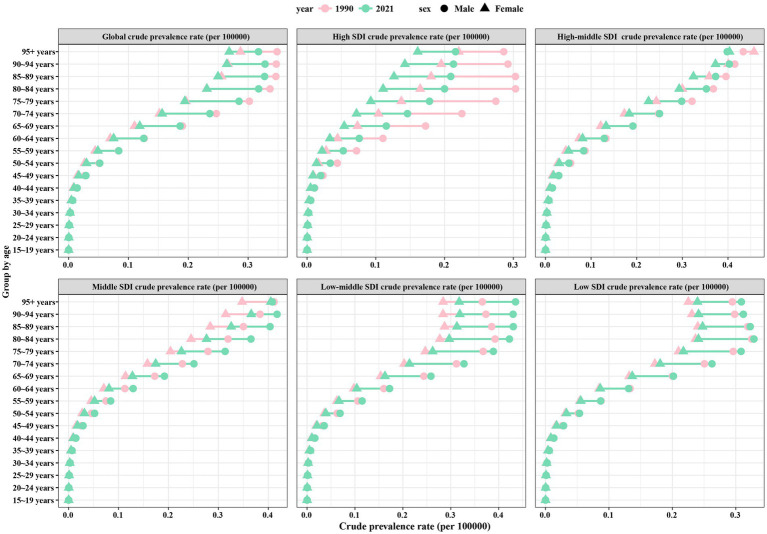
Crude prevalence rate of IHD by sex, age group, and SDI, 1990 and 2021. IHD, ischemic heart disease; SDI, sociodemographic index.

**Figure 3:**
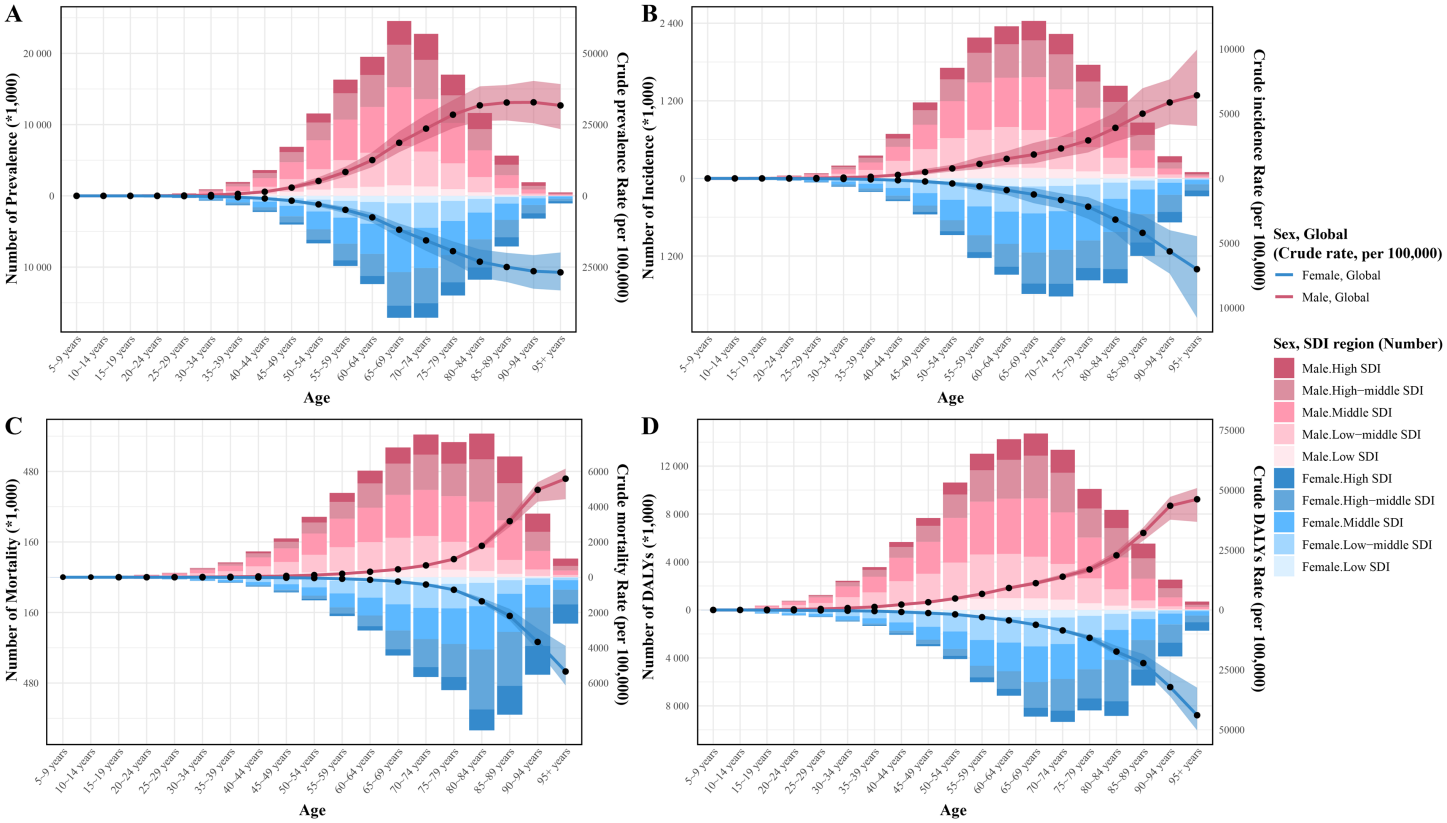
Age-sex-specific numbers and crude rates of IHD burden by SDI region in 2021. **(A)** Crude prevalence rate for IHD; **(B)** Crude incidence rate for IHD; **(C)** Crude morality rate for IHD; and **(D)** Crude DALYs rate for IHD. IHD, ischemic heart disease; DALYs, Disability-Adjusted Life Years; SDI, sociodemographic index.

In 1990, the global prevalent cases of IHD were 112,169,488.4 (95% UI: 99,416,741–125,730,168.7), whereas by 2021, the number of cases has increased to 254,276,267.9 (95% UI: 221,446,458.1–295,493,092.7), a 2.27-fold increase from 1990 (Table 1). Notably, only the total number of cases increased significantly globally. However, the ASPR increased slightly from 2,904.72/100,000 people (95% UI: 2,575.99–3,248.1) in 1990 to 2,946.38/100,000 people (95% UI: 2,572.69–3,424.32) in 2021. However, with an EAPC of 0.01 (95% CI: −0.02–0.03), the ASPR remains flat overall. During this period, the increase in the burden of disease was characterized by a decrease in ASPR in men and an increase in women; however, the ASPR was consistently higher in men than in women (Table 1 and Figure 1). Following further stratification by age, the burden of disease for IHD increased with age. In particular, the crude prevalence rate among those aged <40 years has remained essentially unchanged, with little difference in ASPR between men and women; the crude prevalence rate among those aged 40–74 years has declined slightly compared with that in 1990, showing a decrease in men and an increase in women; and the crude prevalence rate among those aged >75 years has declined significantly, predominantly in men (Figure 2). Specifically, in 2021, the majority of patients with IHD globally will be aged >60 years, with the highest number of cases occurring among those aged 65–74 years; however, those aged >80 years will still have the highest crude prevalence rate of IHD, with a slightly higher crude prevalence rate in men than in women (Figure 3A).

**Table 1:** The prevalence of ischemic heart disease cases and age-standardized rate at global and regional level in 1990 and 2021, with EAPC (1990–2021).

Location	1990		2021		EAPC (95% CI)
	Number	ASR	Number	ASR	
Global	112169488.4 (99416741, 125730168.7)	2904.72 (2575.99, 3248.1)	254276267.9 (221446458.1, 295493092.7)	2946.38 (2572.69, 3424.32)	0.01 (−0.02, 0.03)
Male	64980529.9 (57603504.6, 73044652.3)	3688.47 (3281.54, 4117.92)	145307703.3 (125889573.4, 167448323.8)	3610.24 (3153.05, 4164.95)	−0.08 (−0.1, −0.07)
Female	47188958.6 (41760636.1, 53056584.6)	2250.59 (1998.46, 2539.08)	108968564.5 (95254725.7, 127385890)	2357.61 (2063.31, 2751.95)	0.06 (0.02, 0.1)
High SDI	25246967.8 (22384997.7, 28275596.8)	2271.86 (2020.89, 2539)	34231046.2 (30152992.7, 39201712.3)	1671.6 (1475.88, 1910.43)	−1.21 (−1.35, −1.07)
High-middle SDI	30745716.5 (27332573.3, 34434267)	3197.56 (2857.64, 3581.49)	63826218.3 (55672804.8, 74555301)	3217.58 (2814.34, 3742.18)	−0.06 (−0.09, −0.02)
Middle SDI	27913691.2 (24588240.4, 31686021.3)	2868.41 (2526.85, 3236.77)	85371063.1 (73614482.5, 100118565.1)	3226.3 (2806.67, 3798.24)	0.39 (0.37, 0.42)
Low-middle SDI	21499369.6 (19030104.6, 24197902.2)	3717.21 (3292.3, 4187.06)	55198197.2 (48035746, 63649428.1)	3941.42 (3448.94, 4577.03)	0.22 (0.2, 0.23)
Low SDI	6620304.7 (5896789.5, 7438261.2)	3100.35 (2764.33, 3477.56)	15420053.3 (13308339.2, 17818345.5)	3162.53 (2763.3, 3679.6)	0.03 (0.02, 0.04)
Andean Latin America	411051.4 (374685.4, 453409.1)	2041.83 (1855.5, 2255.33)	1320142.3 (1175584.1, 1473053.8)	2236.34 (1992.49, 2499.74)	0.34 (0.29, 0.39)
Australasia	575088.4 (531489.9, 625054.1)	2451.65 (2266.93, 2660.32)	1055922.1 (937657.9, 1198106.7)	1966.99 (1749.66, 2225.89)	−0.85 (−1.01, −0.69)
Caribbean	817912.2 (748442.7, 895355.2)	3182.4 (2910.4, 3484.14)	1719015.2 (1545788, 1913703.1)	3184 (2861.96, 3542.79)	−0.04 (−0.1, 0.02)
Central Asia	1826093.9 (1697743.4, 1985355.9)	4095.93 (3802.28, 4441.37)	3360424.6 (3054199.1, 3688626.4)	4408.38 (4040.59, 4801.11)	0.24 (0.21, 0.27)
Central Europe	5510994.9 (4914268.3, 6121939)	3763.21 (3383.24, 4156.9)	7088998.3 (6244109.9, 7996466.6)	3192.86 (2824.44, 3571.8)	−0.77 (−0.87, −0.66)
Central Latin America	2186085.6 (1964375.1, 2431257)	2694.29 (2423.74, 2989.19)	6550181.1 (5738031.5, 7491879.7)	2625.17 (2306.27, 2997.16)	−0.15 (−0.18, −0.11)
Central Sub-Saharan Africa	471511.6 (428138.5, 519525.4)	2274.81 (2061.01, 2502.92)	1121950.9 (1003281.6, 1251660.2)	2151.75 (1934.11, 2416.46)	−0.24 (−0.28, −0.2)
East Asia	20306815.5 (17497234.2, 23369855.7)	2534.1 (2206.04, 2917.75)	65378738.6 (55703355.2, 78423916.9)	3031.24 (2597.74, 3606.16)	0.61 (0.54, 0.69)
Eastern Europe	12233736.4 (10760238.4, 13888310.9)	4531.32 (3997.32, 5112.99)	17417637.4 (15088871.1, 20450190.5)	4942.65 (4299.11, 5766.77)	0.2 (0.12, 0.29)
Eastern Sub-Saharan Africa	1511536.3 (1341342.3, 1689486)	2114.08 (1870.7, 2361.79)	3667714.7 (3201109.3, 4187513.1)	2224.88 (1931.93, 2554.41)	0.12 (0.1, 0.14)
High-income Asia Pacific	1927510 (1676871.7, 2219259.3)	967.93 (844.87, 1108.66)	3826068.7 (3304656.7, 4472351.5)	821.73 (714.58, 948.26)	−0.77 (−0.88, −0.66)
High-income North America	10084084.6 (8571681.5, 11762329)	2850.67 (2436.07, 3310.94)	9762663 (8311763, 11497495.8)	1494.62 (1280.2, 1746.9)	−2.47 (−2.65, −2.28)
North Africa and Middle East	10286345.5 (9619257.9, 11065821.8)	6435.15 (6033.55, 6897.62)	28353618.5 (25902216.1, 31330582)	6404.84 (5872.02, 7041.08)	−0.07 (−0.09, −0.04)
Oceania	71569.4 (65254.7, 78323.6)	2810.98 (2578.7, 3077.14)	193152.3 (172499.8, 215291.8)	2912.09 (2623.82, 3229.74)	0.14 (0.12, 0.16)
South Asia	22654345.8 (19415380.4, 26136299.2)	4173.32 (3601.46, 4808.33)	64277536 (54216694.5, 76364441.8)	4455.73 (3796.73, 5339.2)	0.22 (0.21, 0.23)
Southeast Asia	4659271.9 (4164301.6, 5206583.6)	2001.92 (1796.9, 2238.15)	12934270.8 (11289451.1, 14655743.1)	2088.43 (1847.37, 2376.73)	0.17 (0.14, 0.19)
Southern Latin America	832055.7 (761052, 919385.8)	1805.87 (1654.58, 1985.54)	1342051.3 (1209575.7, 1499258.1)	1538.85 (1388.1, 1718.57)	−0.71 (−0.79, −0.62)
Southern Sub- Saharan Africa	743247.2 (646889.9, 849314.8)	2858.77 (2487.01, 3285.62)	1557029 (1326447.6, 1829630.2)	2780.69 (2387.17, 3277.9)	−0.19 (−0.24, −0.14)
Tropical Latin America	1768219.9 (1514249.4, 2025632.5)	1954.51 (1676.11, 2253.78)	5084851.7 (4282245.7, 5980523.6)	1977.99 (1672.38, 2324.96)	0.07 (0.04, 0.1)
Western Europe	11268545.9 (10086320.2, 12564353)	1947.72 (1747.81, 2162.57)	13285326.7 (11713332.8, 15180897.2)	1480.14 (1305.92, 1679.62)	−1.05 (−1.11, −0.98)
Western Sub-Saharan Africa	2023466.2 (1790034.6, 2270457.9)	2399.14 (2121.66, 2703.68)	4978974.6 (4339864, 5706176.2)	2624.72 (2284.48, 3000.35)	0.32 (0.3, 0.34)

#### 3.1.2 Incidence

For incidence, the number of IHD cases in 2021 was 31,872,778.2 (95% UI: 26,284,920.9–38,267,834.3), a 2.002-fold increase from that in 1990 (15,813,618.6, 95% UI: 13,180,529.4–18,849,478.5). However, ASIR declined from 419.54/100,000 people (95% UI: 351.07–498.15) in 1990 to 372.9/100,000 people (95% UI: 307.95–444.19) in 2021, with an EAPC of −0.44 (95% CI: −0.47 to −0.42), and the ASIR for both men and women showed a decreasing trend; however, that of men was always higher than that of women (Table 2 and Figure 1). Comparing 1990 with 2021, the difference in crude incidence rates between men and women was not significant and remained essentially stable among those aged <54 years; however, among those aged >55 years, the crude incidence rate showed a significant downward trend and was most prominent in men, with the greatest magnitude in the older age group of ≥95 years (Supplementary Figure S1). In 2021, the crude incidence rate of IHD increased with age, with those aged 65–69 years having the highest crude incidence rate of IHD. The 65–69 age group has the highest number of cases, and more men are involved than women, while the ≥95 years age group has the highest crude incidence rate, with slightly more women involved than men (Figure 3B).

**Table 2:** The incidence of ischemic heart disease cases and age-standardized rate at global and regional level in 1990 and 2021, with EAPC (1990–2021).

Location	1990		2021		EAPC (95% CI)
	Number	ASR	Number	ASR	
Global	15813618.6 (13180529.4, 18849478.5)	419.54 (351.07, 498.15)	31872778.2 (26284920.9, 38267834.3)	372.9 (307.95, 444.19)	−0.44 (−0.47, −0.42)
Male	9070633.4 (7534343, 10889169.9)	522.16 (436.31, 617.87)	17961630.3 (14860851.6, 21521548.3)	450.39 (373.66, 534.62)	−0.48 (−0.5, −0.46)
Female	6742985.3 (5591820.6, 8076377)	329.74 (273.29, 392.76)	13911147.9 (11446945.7, 16678573.7)	301.57 (248.57, 360.73)	−0.42 (−0.47, −0.37)
High SDI	3768111.3 (3189322.5, 4475354)	343.51 (290.81, 407.03)	3989835.2 (3384024, 4709126.3)	195.63 (164.52, 231.53)	−2.04 (−2.28, −1.81)
High-middle SDI	4242517.6 (3542999.3, 5036433.9)	462.74 (386.71, 546.34)	7874112.4 (6457244.1, 9448768)	404.44 (331.92, 480.98)	−0.58 (−0.69, −0.47)
Middle SDI	3654917.8 (2965634, 4446596.1)	382.77 (314.26, 459.87)	10499399.9 (8567480.1, 12702596.1)	403.84 (330.37, 481.69)	0.22 (0.15, 0.28)
Low-middle SDI	3125084.9 (2574308.2, 3805366.5)	531.54 (440.44, 637.69)	7292860.1 (6121118.8, 8702139.9)	515.6 (433.64, 614.95)	−0.09 (−0.13, −0.05)
Low SDI	1004366.9 (814679.1, 1236950.5)	471.41 (384.07, 572.6)	2190922.8 (1807960.1, 2673792.3)	444.61 (362.9, 537.75)	−0.3 (−0.36, −0.25)
Andean Latin America	50619.5 (41422, 61339.6)	249.99 (203.02, 303.51)	139102.9 (113541.6, 169610)	233.09 (188.74, 282.95)	−0.31 (−0.38, −0.23)
Australasia	84214.4 (73573.8, 97267.8)	365.94 (321.53, 421.77)	116017.3 (95474.5, 139639.3)	220.63 (181.56, 267.7)	−1.7 (−1.98, −1.41)
Caribbean	104244.2 (87044.2, 122816.7)	412.47 (346.61, 484.8)	195155.8 (161425.9, 231734.5)	360.97 (298.09, 428.1)	−0.48 (−0.59, −0.37)
Central Asia	281995.5 (245768.8, 329015.9)	641.97 (560.8, 735.59)	573033.9 (510496.8, 653368)	801.56 (731.97, 893.8)	0.7 (0.54, 0.86)
Central Europe	697968.1 (609103.3, 805202.1)	494.65 (434.03, 565.43)	705225.4 (612792.6, 813235.1)	322.44 (280.41, 371.5)	−1.82 (−2.03, −1.6)
Central Latin America	280079.6 (229936.4, 338385.4)	343.14 (279.83, 415.19)	775551 (634023.6, 938828.8)	309.82 (253.98, 372.65)	−0.42 (−0.48, −0.36)
Central Sub-Saharan Africa	77096.6 (63116.1, 94968)	373.7 (310.54, 445.96)	181545.3 (148129, 222440.8)	344.02 (286.71, 408.91)	−0.39 (−0.43, −0.35)
East Asia	2400603 (1941066.8, 2911498.8)	316.29 (256.63, 382.83)	7541711.5 (6010952.7, 9229492.8)	363.64 (291.86, 437.51)	0.62 (0.47, 0.77)
Eastern Europe	1835281 (1497315.7, 2193024.8)	710.56 (581.31, 851.38)	2491976.7 (2009144.6, 3043099)	714.22 (578.98, 859.74)	−0.23 (−0.49, 0.03)
Eastern Sub-Saharan Africa	224084.5 (177674, 279662.8)	322.84 (254.71, 398.76)	516096 (414727.1, 642412.7)	314.4 (250.55, 386.71)	−0.22 (−0.27, −0.16)
High-income Asia Pacific	206094 (166290.5, 251344.1)	106.59 (86.55, 129.28)	460224.3 (366622.3, 577587.8)	92.4 (73.46, 115.1)	−0.58 (−0.78, −0.38)
High-income North America	1581463.5 (1246575.1, 1975540.9)	454.96 (360.35, 569.19)	1114553.2 (941548.8, 1307276.6)	174.12 (147.07, 203.07)	−3.55 (−3.82, −3.27)
North Africa and Middle East	1595178.4 (1382346.6, 1887102.9)	984.29 (859.82, 1153)	4046958.3 (3504192, 4785078.7)	895.85 (786.65, 1043.49)	−0.42 (−0.49, −0.34)
Oceania	9458.7 (7342.6, 11898)	375.43 (291.97, 470.92)	25258.4 (20071.9, 31438)	378.32 (297.3, 469.96)	0.03 (0.02, 0.05)
South Asia	3343753.6 (2669989, 4119600.5)	600.21 (482.65, 736.67)	8437391.7 (6853498.8, 10223098.7)	580.24 (472.94, 704.59)	−0.13 (−0.21, −0.05)
Southeast Asia	551918.4 (446571.9, 668746.9)	239.91 (198.42, 287.22)	1419034.9 (1164938.5, 1720664.7)	231.58 (193.09, 275.95)	−0.05 (−0.12, 0.02)
Southern Latin America	139690.4 (120951.9, 162646.2)	315.01 (275.93, 362.59)	174479.2 (150037.9, 203601.8)	201.48 (172.9, 236.15)	−1.68 (−1.93, −1.43)
Southern Sub-Saharan Africa	106341.4 (84122.9, 132913.2)	403.21 (317.75, 504.39)	213190.3 (168672, 266327.1)	378.48 (299.2, 467.87)	−0.36 (−0.44, −0.29)
Tropical Latin America	180542.1 (151059.1, 214652.6)	196.85 (162.95, 234.66)	431283.9 (349771.5, 518457.9)	167.8 (136.41, 200.76)	−0.37 (−0.44, −0.29)
Western Europe	1753090.8 (1575512.3, 1973071.5)	305.36 (274.35, 343.39)	1596982 (1383367.5, 1853127.4)	172.46 (147.8, 203.44)	−1.89 (−2, −1.79)
Western Sub-Saharan Africa	309900.9 (247586, 383976.5)	376.08 (296.96, 463.5)	718006.4 (581487.3, 884861.6)	379.25 (303.36, 464.67)	−0.07 (−0.1, −0.03)

#### 3.1.3 Mortality

From 1990 to 2021, IHD-related deaths increased by 67.53% from 5,367,136.6 (95% UI: 5,076,403.9–5,562,773.9) in 1990 to 8,991,636.7 (95% UI: 8,264,123.2–9,531,130.2) in 2021. However, ASMR decreased from 158.9/100,000 people (95% UI: 148.14–165.3) in 1990 to 108.73/100,000 people (95% UI: 99.6–115.38) in 2021 with an EAPC of −1.3 (95% CI: −1.33 to −1.27) (Table 3). In this case, both men and women showed a significant downward trend in ASMR, with that in men consistently higher than that in women (Figure 1). Compared with that of 1990, the crude mortality rate for those aged ≥45 years showed a significant downward trend, with the sex difference being most pronounced among those aged 90–94 years and the decline being greatest among those aged ≥95 years (Supplementary Figure S2). Among them, deaths occurred mainly among those aged 65–89 years and were highest among those aged 80–84 years, with slightly more women involved than men, while the highest crude mortality rate occurred among those aged ≥95 years, with slightly more men involved than women (Figure 3C).

**Table 3:** The mortality of ischemic heart disease cases and age-standardized rate at global and regional level in 1990 and 2021, with EAPC (1990–2021).

Location	1990		2021		EAPC (95% CI)
	Number	ASR	Number	ASR	
Global	5367136.6 (5076403.9, 5562773.9)	158.9 (148.14, 165.3)	8991636.7 (8264123.2, 9531130.2)	108.73 (99.6, 115.38)	−1.3 (−1.33, −1.27)
Male	2804950.1 (2673799, 2921926.6)	187.66 (177.77, 195.35)	5002681.1 (4679269.1, 5336245.4)	136.84 (127.37, 145.9)	−1.12 (−1.16, −1.09)
Female	2562186.5 (2357409.9, 2702952.8)	134.5 (122.46, 142.23)	3988955.6 (3540381.5, 4317969.4)	85.32 (75.9, 92.31)	−1.52 (−1.55, −1.49)
High SDI	1732392.1 (1591274.9, 1797849.3)	157.59 (144.19, 163.92)	1392371 (1217171.6, 1489069.7)	58.45 (52.18, 61.92)	−3.3 (−3.37, −3.22)
High-middle SDI	1604862.2 (1527808.3, 1657939.8)	193.94 (182.11, 200.6)	2450426.3 (2218836.4, 2647300.5)	127.5 (115.03, 137.72)	−1.24 (−1.39, −1.09)
Middle SDI	1038968.2 (978516.2, 1100277.1)	127.06 (118.35, 134.97)	2811121.6 (2564657.4, 3023433.6)	118.71 (107.23, 127.8)	−0.16 (−0.2, −0.11)
Low-middle SDI	753129.8 (698697.1, 804552.2)	140.99 (129.76, 151.24)	1834652.9 (1699506.2, 1964078.9)	142.1 (131.3, 151.87)	0.04 (−0.02, 0.09)
Low SDI	228811.5 (202386.1, 255182.9)	119.5 (106.26, 132.45)	493475.8 (448128.8, 543115.2)	116.41 (105.21, 127.69)	−0.05 (−0.11, 0.02)
Andean Latin America	17089.4 (15548.2, 18721.2)	92.95 (84.56, 101.24)	32964.7 (28040.2, 39366.2)	58.17 (49.56, 69.33)	−1.93 (−2.16, −1.71)
Australasia	40160.9 (37289, 41659.2)	176.76 (162.82, 184.02)	28640.4 (24369.9, 30972.5)	46.67 (40.23, 50.21)	−4.29 (−4.39, −4.18)
Caribbean	44603.1 (42479.9, 46171.1)	188.97 (179.08, 195.79)	61450 (54942.2, 69041)	112.5 (100.51, 126.54)	−1.85 (−1.98, −1.72)
Central Asia	131771.2 (124692.1, 136248.9)	320.47 (299.83, 332.36)	175394 (159202.7, 192406.5)	265.51 (240.67, 290.42)	−0.26 (−0.53, 0)
Central Europe	364089.9 (349914.7, 371867.9)	272.56 (259.22, 279.18)	331281.3 (299877.6, 352596.6)	139.98 (126.84, 148.91)	−1.86 (−2.06, −1.67)
Central Latin America	88861.8 (84857.2, 90852.2)	125.72 (118.69, 129.14)	247050.8 (221193.8, 273150.3)	103.69 (92.48, 114.59)	−0.92 (−1.06, −0.77)
Central Sub-Saharan Africa	23271.7 (18250.8, 29475.8)	134.9 (107.86, 167.2)	49850 (38888.6, 63650.1)	119.34 (93.7, 150.29)	−0.49 (−0.55, −0.44)
East Asia	570428.7 (505994.3, 639930.4)	93.92 (83.87, 105.3)	2008011.1 (1683969.7, 2335232.3)	108.9 (91.18, 125.79)	0.67 (0.47, 0.86)
Eastern Europe	786270.6 (751128.3, 803303.1)	323.17 (305.26, 331.76)	903624.6 (811062.8, 990581)	252.89 (226.96, 277.15)	−0.53 (−0.87, −0.2)
Eastern Sub-Saharan Africa	43989.3 (39100.7, 50522.2)	69.44 (61.05, 78.85)	101222.4 (87734.7, 117265.4)	72.16 (62.09, 82.99)	0.27 (0.17, 0.37)
High-income Asia Pacific	117621.6 (107063.6, 122952.5)	67.04 (59.85, 70.5)	152702.3 (123996, 168788.5)	25.56 (21.78, 27.65)	−3.14 (−3.25, −3.04)
High-income North America	644904 (580448, 675667.3)	177.72 (160.27, 186.07)	534770.4 (468399.9, 571529.3)	75.85 (67.17, 80.6)	−3.07 (−3.16, −2.98)
North Africa and Middle East	386036.5 (357949, 418208.4)	275.18 (253.62, 299.12)	769135.1 (685360, 858253.2)	202.85 (180.59, 223.68)	−0.88 (−0.94, −0.82)
Oceania	4528.2 (3745.9, 5489.1)	182.55 (155.47, 217.42)	11136.9 (9328.8, 13321.1)	170.89 (145.43, 201.15)	−0.01 (−0.07, 0.06)
South Asia	708221 (643038.5, 771051.8)	136.39 (122.87, 149.55)	1990113.5 (1824490.3, 2155696.1)	149.14 (136.97, 161.16)	0.34 (0.26, 0.43)
Southeast Asia	252647 (229370.5, 275073.8)	114.72 (103.39, 125.65)	638703.9 (575906.6, 694079.5)	110.92 (100.18, 120.2)	0.05 (−0.01, 0.11)
Southern Latin America	62788.3 (59933, 64743.2)	149.43 (141.12, 154.54)	49098.5 (45022.3, 51734.5)	54.41 (50.08, 57.26)	−3.28 (−3.42, −3.14)
Southern Sub-Saharan Africa	17901.2 (15561.9, 19842.9)	75.96 (64.95, 84.84)	39816.6 (36778, 43187.1)	83.44 (76.93, 90.19)	0.37 (0.13, 0.62)
Tropical Latin America	107545.4 (102011.5, 110638.5)	135.91 (126.39, 140.82)	162299.2 (149052.8, 170324.8)	64.49 (58.98, 67.84)	−2.42 (−2.5, −2.34)
Western Europe	879289.9 (811263.8, 910871)	148.22 (136.54, 153.85)	543038.4 (463990.2, 584339.4)	47.27 (41.45, 50.42)	−3.57 (−3.68, −3.46)
Western Sub-Saharan Africa	75117.1 (64540.8, 87128.8)	105.29 (90.24, 121.56)	161332.7 (140025.9, 185440.5)	105.97 (92.83, 120.17)	0.13 (0.05, 0.2)

#### 3.1.4 DALYs

Over the past 32 years, DALYs have increased from 119,162,957.3 (95% UI: 114,547,786.9–123,454,733) in 1990 to 188,360,557.3 (177,036,930.4–198,154,476.6) in 2021, an increase of 58.07%. In contrast, ASDR decreased from 3107.61/100,000 people (95% UI: 2966.5–3222.67) in 1990 to 2212.16/100,000 people (95% UI: 2075.54–2327.61) in 2021, with an EAPC of −1.2 (95% CI: −1.25 to −1.16) (Table 4 and Figure 1). In particular, crude DALY rate increased with age, showing a significant downward trend in those aged >40 years, with the largest percentage decrease in those aged >95 years and the largest sex difference in those aged 90–94 years. In 1990, women aged >95 years had a higher crude DALY rate than men; however, men had a higher crude DALY rate in all other age groups; in 2021, in all age groups, ASDR was consistently higher in men (Supplementary Figure S3). In addition, DALYs were highest among those aged 65–74 years, and crude DALY rate was highest among men aged >95 years (Figure 3D).

**Table 4:** The disability-adjusted life years (DALYs) of ischemic heart disease cases and age-standardized rate at global and regional level in 1990 and 2021, with EAPC (1990–2021).

Location	1990		2021		EAPC (95% CI)
	Number	ASR	Number	ASR	
Global	119162957.3 (114547786.9, 123454733)	3107.61 (2966.5, 3222.67)	188360557.3 (177036930.4, 198154476.6)	2212.16 (2075.54, 2327.61)	−1.2 (−1.25, −1.16)
Male	69617735.8 (66638971.8, 72797021.5)	3875.39 (3701.44, 4047.29)	114982353.8 (107837252.6, 123113032.7)	2890.65 (2714.81, 3091.32)	−1.04 (−1.08, −0.99)
Female	49545221.5 (46222314.9, 52216634)	2407.52 (2233, 2541.74)	73378203.5 (67274276.5, 78561320.2)	1596.14 (1463.98, 1706.95)	−1.45 (−1.5, −1.39)
High SDI	32005742.2 (30325688.9, 32877811)	2919.5 (2762.75, 3002.02)	23546711.4 (21449543.9, 24737628.8)	1134.02 (1053.56, 1186.53)	−3.26 (−3.37, −3.14)
High-middle SDI	33569030.7 (32291953.7, 34708784.6)	3589.76 (3435.61, 3712.83)	44542561.5 (41134046.5, 48044417.5)	2301.49 (2122.97, 2482.46)	−1.74 (−2, −1.48)
Middle SDI	26527074.2 (25117637.6, 28067547.6)	2593.46 (2447.25, 2745.56)	61242796.6 (56964813.1, 65533800.6)	2351.21 (2174.84, 2514.63)	−0.27 (−0.32, −0.23)
Low-middle SDI	20676778.5 (19223601.7, 22114207.2)	3221.76 (2993.33, 3437.47)	46062373.8 (42687627.6, 49488217.3)	3138.58 (2912.65, 3360.62)	0.02 (−0.03, 0.08)
Low SDI	6194944.9 (5447647.4, 6954569.8)	2668.94 (2361.3, 2967.21)	12778296.9 (11572088.2, 14121058.9)	2464.12 (2235.87, 2725.2)	−0.26 (−0.34, −0.18)
Andean Latin America	389626.7 (355358.5, 430137.3)	1858.09 (1696.99, 2045.72)	681780.8 (578423.1, 811744.2)	1150.61 (976.72, 1369.62)	−1.77 (−2.14, −1.39)
Australasia	752012.8 (712619.3, 773988)	3237.02 (3058.2, 3338.9)	446236.6 (400159.6, 474158.6)	812.69 (739.29, 858.37)	−4.68 (−4.82, −4.54)
Caribbean	950094.5 (906314.9, 988524.9)	3723.19 (3551.61, 3873.17)	1291386.8 (1138002.5, 1470659)	2397.8 (2112.34, 2730.37)	−1.43 (−1.63, −1.22)
Central Asia	2808715 (2697191.8, 2899667.3)	6206.85 (5937.37, 6407.43)	3674595.5 (3323875.3, 4049458.4)	4864.49 (4415.55, 5338.75)	−1.31 (−1.65, −0.98)
Central Europe	7442730.7 (7251102.1, 7571719.8)	5198.33 (5046.13, 5293.63)	5524245.1 (5103797.4, 5888037.3)	2471.23 (2288.2, 2635.38)	−2.75 (−2.86, −2.64)
Central Latin America	2013987.9 (1954877.9, 2051366.6)	2445.66 (2357.94, 2497.28)	5014766.5 (4523533.4, 5572802.5)	2016.97 (1821.53, 2238.28)	−0.82 (−1.05, −0.59)
Central Sub-Saharan Africa	610385.4 (476421.9, 774810.2)	2816.5 (2225.44, 3535.98)	1296119.2 (1001600.1, 1666371.5)	2433.06 (1902.53, 3100.83)	−0.64 (−0.73, −0.56)
East Asia	14164879.8 (12519625.7, 16050196.2)	1770.78 (1574, 1979.4)	36779981 (30982599.3, 42822726.1)	1839.92 (1541.21, 2135.32)	0.46 (0.22, 0.72)
Eastern Europe	15693814.3 (15174105.9, 16002683.9)	5957.77 (5735.98, 6088.42)	16349319.1 (14850165.2, 17851788.1)	4687.7 (4267.79, 5115.68)	−1.34 (−1.86, −0.81)
Eastern Sub-Saharan Africa	1209724.4 (1076473.8, 1395912.9)	1556.44 (1388.67, 1773.02)	2710701.2 (2345578.7, 3122231)	1536.64 (1333.12, 1773.89)	−0.25 (−0.34, −0.16)
High-income Asia Pacific	2221306.7 (2085563.9, 2305288.6)	1165.81 (1080.72, 1212.46)	2313089.6 (2010801.8, 2492541.9)	492.99 (446.93, 520.22)	−2.75 (−2.84, −2.67)
High-income North America	11711568.1 (10945809.4, 12090045.2)	3347.44 (3141.1, 3449.54)	9426914.8 (8621683.6, 9884596.8)	1461.9 (1350.51, 1526.21)	−2.98 (−3.13, −2.84)
North Africa and Middle East	9832454.9 (9159577.2, 10677658.8)	5763.47 (5366.03, 6251.52)	18148597.8 (16140157.2, 20526102.8)	4023.22 (3581.71, 4507.47)	−1.24 (−1.27, −1.2)
Oceania	136528.3 (111593.7, 168663.8)	4259.05 (3546.41, 5124.64)	327008.3 (270023.1, 393473.4)	3962.77 (3323.16, 4723.71)	−0.19 (−0.24, −0.14)
South Asia	20470723.9 (18722018.5, 22243363.9)	3276.85 (2983.93, 3564.63)	50666052.9 (46308000.4, 54713621.3)	3351.09 (3075.41, 3616.42)	0.17 (0.07, 0.26)
Southeast Asia	6837277.5 (6257622.5, 7403583.1)	2549.17 (2315.76, 2761.72)	15931224.4 (14296723, 17482653.9)	2415.55 (2177.87, 2635.3)	−0.16 (−0.22, −0.11)
Southern Latin America	1264775.1 (1223961.9, 1297794.2)	2821.47 (2718.03, 2901.05)	931016.8 (877897.2, 968372)	1070.86 (1012.57, 1113.22)	−2.86 (−3, −2.72)
Southern Sub-Saharan Africa	452076.8 (404055.3, 494626.3)	1626.44 (1424.74, 1789.71)	957913.5 (887404.7, 1042420.5)	1689.53 (1565.07, 1832.24)	0.11 (−0.3, 0.52)
Tropical Latin America	2675370.3 (2581470.8, 2739000.6)	2899.87 (2774.08, 2979.35)	3817675 (3591188.6, 3962635)	1476.14 (1385.48, 1533.17)	−2.13 (−2.2, −2.06)
Western Europe	15770965.2 (14972578.3, 16197676.4)	2740.65 (2604.66, 2812.94)	8262192.4 (7385900.6, 8764788.3)	843.77 (775.27, 886.1)	−4 (−4.1, −3.9)
Western Sub-Saharan Africa	1753938.9 (1491914.4, 2043990.4)	2082.33 (1785.44, 2418.3)	3809740 (3233241.9, 4452024.1)	2029.03 (1760.44, 2334.06)	−0.1 (−0.24, 0.04)

Overall, while the global burden of IHD remains high, ASIR, ASMR, and ASDR have all shown varying degrees of decline.

### 3.2 Regional trend by SDI

According to our assessment, the burden of IHD varies significantly among regions with different SDI levels.

#### 3.2.1 Prevalence

The 2021 data showed that the highest number of prevalent cases was found in the MSDI region at 85,371,063.1 (95% UI: 73,614,482.5–100,118,565.1), the lowest number of prevalent cases was found in the LSDI region at 15,420,053.3 (95% UI: 13,308,339.2–17,818,345.5). While the LMSDI region had the highest ASPR of 3,941.42 per 100,000 people (95% UI: 3,448.94–4,577.03), the HSDI region had the lowest ASPR of 1,671.6 per 100,000 people (95% UI: 1,475.88–1,910.43). There was a significant downward rate in ASPR in the HSDI region, with an EAPC of −1.21 (95% CI: −1.35 to −1.07), the largest decrease of 26.42%, while there was a significant upward trend in the MSDI region, with an EAPC of 0.39 (95% CI: 0.37–0.42), an increase of 12.48% (Table 1 and Figure 1). After further stratification by age and sex, we found that the increase in the number of cases in the MSDI region was most pronounced in the 65–69 age group in 2021. In contrast, the decline in crude prevalence rate in the HSDI region was concentrated among those aged ≥60 years (Figure 2 and Figure 3A). This difference reflects the regional and age-stratified characteristics of the disease burden, that is, the aging population in the HSDI region is more significant, resulting in a relatively large number of older adult patients in the region, but the ASPR burden of the entire population is still mainly in the LMSDI region.

#### 3.2.2 Incidence

For ASIR, there was an overall decreasing trend in all regions except for the MSDI region. In 2021, the highest number of cases was found in the MSDI region, with 10,499,399.9 (95% UI: 8,567,480.1–12,702,596.1), representing an increase of 187.27% compared with that in 1990; the lowest number of cases was found in the LSDI region, with 2,190,922.8 (95% UI: 1,807,960.1–2,673,792.3). The HSDI region had the lowest ASIR of 195.63/100,000 people (95% UI: 164.52–231.53), while the LMSDI region had the highest ASIR of 515.6/100,000 people (95% UI: 433.64–614.95). The most significant decrease in ASIR was observed in the HSDI region, with an EAPC of −2.04 (95% CI: −2.28 to −1.81), whereas the EAPC in the MSDI region was 0.22 (95% CI: 0.1–0.28), the most significant increase (Table 2 and Figure 1). In particular, the increased crude incidence rate in the MSDI region was concentrated among those aged 50–79 years, with little difference between the age groups (Figure 3B). The decreased crude incidence rate in the HSDI region was most significant among those aged 90–94 years; the increased crude incidence rate in the MSDI region was concentrated among those aged 75–89 years (Supplementary Figure S1).

#### 3.2.3 Mortality

In 2021, the highest number of deaths occurred in the MSDI region (2,811,121.6; 95% UI: 2,564,657.4–3,023,433.6) and the lowest in the LSDI region (493,475.8; 95% UI: 448,128.8–543,115.2). From 1990 to 2021, there was a decrease in ASMR across all SDI regions except the LMSDI region, with the lowest ASMR (58.45/100,000 people; 95% UI: 52.18–61.92) in the HSDI region and the highest (142.1/100,000 people; 95% UI: 131.3–151.87) in the LMSDI region. The most significant decrease was in the HSDI region, with an EAPC of −3.3 (95% CI: −3.37 to −3.22), while the LMSDI region was the only region with an increase in ASMR, with an EAPC of 0.04 (95% CI: −0.02–0.09) (Table 3 and Figure 1). In particular, the decreased crude mortality rate in the HSDI region and the increased crude mortality rate in the LMSDI region were most pronounced among those aged >95 years, with more men involved than women (Supplementary Figure S2). In addition, we found that in the HMSDI region, the highest number of deaths occurred among those aged 80–84 years; however, the LMSDI region still has the highest ASMR (Figure 3C).

#### 3.2.4 DALYs

Changes in ASDR were more similar to changes in ASMR. In 2021, ASDR was highest in the LMSDI region at 3138.58/100,000 people (95% UI: 2912.65–3360.62) and lowest in the HSDI region at 1134.02/100,000 people (95% UI: 1053.56–1186.53). From 1990 to 2021, ASDR declined to varying degrees in all five SDI regions, with the most significant decline in the HSDI region, with an EAPC of −3.26 (95% CI: −3.37 to −3.14), and the smallest decline in the LMSDI region (EAPC 0.02; 95% CI: −0.03–0.08) predominantly in women (Table 4 and Figure 1). In particular, the decline in the HSDI region was concentrated among those aged >50 years and was most significant among those aged >95 years (Supplementary Figure S3). However, the larger number of DALYs was still dominated by the MSDI region and was concentrated among those aged 45–84 years (Figure 3D).

This trend suggests that while the burden of IHD is effectively controlled in HSDI regions, treatment and health care in LMSDI regions still need more attention and effort.

### 3.3 Regional trend by GBD

#### 3.3.1 Prevalence

At the level of the 21 GBD geographic regions, East Asia and Oceania had the highest and lowest number of people with IHD in 2021 (65,378,738.6; 95% UI: 55,703,355.2–78,423,916.9 and 193,152.3, 95% UI: 172,499.8–215,291.8), respectively. ASPR was highest in North Africa and the Middle East with a ASPR of 6404.84/100,000 people (95% UI: 5872.02–7041.08) and the lowest in High-Income Asia Pacific with a ASPR of 821.73/100,000 people (95% UI: 714.58–948.26). East Asia was the region with the highest increase in prevalent cases and ASPR, with a total increase of 221.95% and an EAPC for ASPR of 0.61 (95% CI: 0.54–0.69), while High-Income North America was the only region with a decrease in prevalent cases, with a decrease of 3.19%, and it also had the largest decrease in ASPR with an EAPC of −2.47 (95% CI: −2.65 to −2.28) (Table 1). Furthermore, as shown in Supplementary Figure S4A, the ASPR of IHD was negatively correlated with the SDI, first increasing and then decreasing with increasing SDI, with the ASPR inflection point occurring at an SDI of 0.27 (*ρ* = −0.23, *p* < 0.001). The global ASPR was below the expected, while the ASPR for North Africa, and Middle East, and Eastern Europe exceeded the expected level.

#### 3.3.2 Incidence

In 2021, South Asia and Oceania were the regions with the highest and lowest ASIR (8,437,391.7; 95% CI: 6,853,498.8–10,223,098.7; and 25,258.4; 95% UI: 20071.9–31,438), respectively. The most significant increase in incident cases occurred in East Asia (214.16%), and the largest decrease occurred in High-Income North America (29.52%). Likewise, the downward trend in ASIR was most significant (EAPC −3.55; 95% CI: −3.82 to −3.27). ASIR was highest in North Africa and the Middle East, at 895.85/100,000 people (95% UI: 786.65–1043.49), and the lowest in the High-Income Asia Pacific, with only 92.4/100,000 people (95% UI: 73.46–115.1). The most significant increase in ASIR occurred in Central Asia, with an EAPC of 0.7 (95% CI: 0.54–0.86) (Table 2). In addition, three regions, North Africa and Middle East, Eastern Europe, and Central Asia, had higher ASIR than expected based on the SDI, with the peak ASIR occurring at an SDI of approximately 0.48 (*ρ* = −0.30, *p* < 0.001) (Supplementary Figure S4B).

#### 3.3.3 Mortality

In 2021, Western Europe had the highest number of IHD-related deaths, a whopping 543,038.4 (95% UI: 463,990.2–584,339.4), compared with Oceania, which had the lowest, with 11,136.9 (95% UI: 9,328.8–13,321.1). ASMR increased the most in East Asia (252.02%; EAPC 0.67; 95% CI: 0.47–0.86), while Western Europe had the largest decrease in deaths (38.24%). Central Asia was the region with the largest ASMR (265.51/100,000 people; 95% UI: 240.67–290.42), and the smallest was High-Income Asia Pacific (25.56/100,000 people; 95% UI: 21.78–27.65). The region with the greatest ASMR decline was Australasia, with an alarming EAPC of −4.29 (95% CI: −4.39 to −4.18) (Table 3). Supplementary Figure S4C further demonstrates the relationship between ASMR trends and SDI in these regions, and while there is an overall trend toward decline, North Africa and the Middle East, Central Asia, Eastern Europe, Central Europe, and High-Income North America continued to have higher ASMR than the expected SDI levels.

#### 3.3.4 DALYs

South Asia had the highest number of IHD-associated DALYs (50,666,052.9; 95% UI: 46,308,000.4–54,713,621.3), while Oceania had the lowest (327,008.3; 95% UI: 270,023.1–393,473.4). The largest increase in the number of DALYs was in East Asia (159.66%), and the largest decrease was in Western Europe (47.61%). The Central Asia region had the highest ASDR (4,864.49/100,000 people; 95% UI: 4,415.55–5,338.75), while High-Income Asia Pacific had the lowest (492.99/100,000 people; 95% UI: 446.93–520.22). South Asia and Australasia had the largest and smallest increase in ASDR with an EAPC of 0.17 (95% CI: 0.07–0.26) and EAPC of −4.68 (95% CI: −4.82 to −4.54), respectively (Table 4). Similarly, we find that the graph of the relationship between ASDR and SDI is similar to that of mortality, with an “M” shape, and the regions where ASDR is higher than the expected level of SDI remained North Africa, Middle East, Central Asia, Eastern Europe, Central Europe, and High-Income North America (Supplementary Figure S4D).

Overall, the burden of IHD remained high in Central and Southern Asia, Northern Africa, and the Southeast Coastal region, which contrasted with regions with a reduced burden of IHD, such as the Americas and Oceania.

### 3.4 National trend

#### 3.4.1 Prevalence

**Figure 4:**
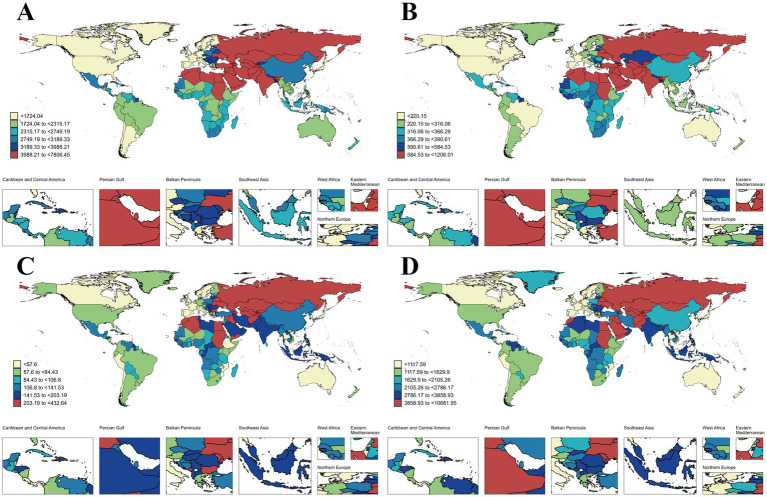
Global map of age-standardized prevalence, incidence, mortality, and DALYs rate for IHD in 2021. **(A)** Age-standardized of prevalence rate; **(B)** Age-standardized of incidence rate; **(C)** Age-standardized of mortality rate; **(D)** Age-standardized of DALYs rate. IHD, ischemic heart disease; DALYs, Disability-Adjusted Life Years.

**Figure 5:**
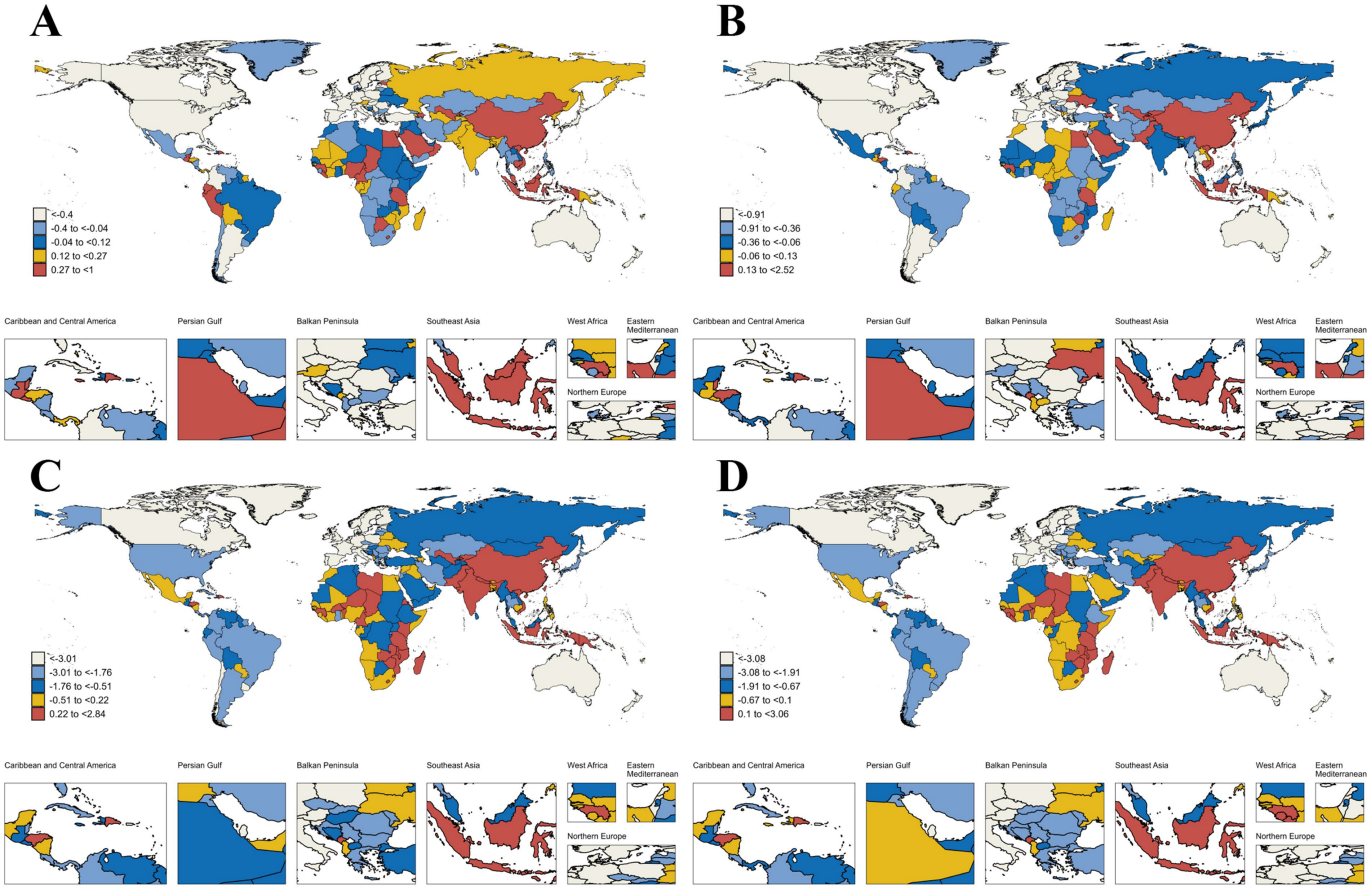
Trends in age-standardized prevalence, incidence, mortality, and DALYs rate for IHD from 1990 to 2021 (EAPC). **(A)** Age-standardized of prevalence rate; **(B)** Age-standardized of incidence rate; **(C)** Age-standardized of mortality rate; **(D)** Age-standardized of DALYs rate. IHD, ischemic heart disease; EAPC, estimated annual percentage change; DALYs, Disability-Adjusted Life Years.

Among the 204 countries, Kuwait and Japan had the highest and lowest ASPR of 7806.45/100,000 people (95% UI: 7141.59–8556.31) and 802.34/100,000 people (95% UI: 686.82–938.16), respectively. The largest increase in ASPR was in Uzbekistan (EAPC 1; 95% CI: 0.89–1.11), and the largest decline was in the United States of America (EAPC −2.57; 95% CI: −2.75 to −2.38) (Supplementary Table S1 and Figures 4A, 5A). In addition, Supplementary Figure S5 shows that country-level ASPR is roughly negatively correlated with SDI; however, this relationship is not significant (*ρ* = −0.08, *p* = 0.241). In particular, countries such as Kuwait, the United Arab Emirates, and Saudi Arabia have significantly higher levels of ASPR than would be expected based on SDI, while Portugal and Brunei Darussalam are well below the expected levels.

#### 3.4.2 Incidence

In 2021, Uzbekistan and Portugal had the highest and lowest ASIR (1206.01/100,000 people; 95% UI: 1125.02–1305.45; 72.93/100,000 people; 95% UI: 61.36–85.88), respectively. From 1990 to 2021, the country with the fastest increase and decrease in ASIR were Uzbekistan (EAPC of 2.52; 95% CI: 2.15–2.9) and the United States of America (EAPC -3.66; 95% CI: −3.94 to −3.38) (Supplementary Table S2 and Figures 4B, 5B). Spearman’s rank correlation analysis in Supplementary Figure S6 showed a significant negative correlation between ASIR and SDI in 204 countries (*ρ* = −0.27, *p* < 0.001), with the ASIR in Uzbekistan, the Syrian Arab Republic, and the United Arab Emirates being significantly higher than the expected levels, while that in Portugal, Chile, Spain, and Brunei Darussalam were significantly lower than expected levels.

#### 3.4.3 Mortality

In 2021, Nauru and Sn Marino had the highest and lowest IHD-related ASMR, at 432.64/100,000 people (95% UI: 361.02–517.42) and 23.19/100,000 people (95% UI: 15.63–32), respectively. Lesotho and Denmark were the countries with the fastest increase and decrease in ASMR (EAPC of 2.84; 95% CI: 2.31–3.38; EAPC of −5.83; 95% CI: −5.99 to −5.66), respectively (Supplementary Table S3 and Figures 4C, 5C). Supplementary Figure S7 shows a significant decrease in ASMR with SDI for 204 countries (*ρ* = −0.30, *p* < 0.001). Among them, Nauru has a significantly higher ASMR than expected level based on SDI, which is much higher than the other countries. ASMR in Portugal was well below the expected level.

#### 3.4.4 DALYs

Similar to IHD-related ASMR, by 2021, Nauru and San Marino had the highest and lowest IHD-related ASDR (10681.95/100,000 people; 95% UI: 8619.33–13238.75 and 429.12/100,000 people; 95% UI: 298.92–591.84), respectively. Further, the countries with the fastest change in ASDR remained unchanged: increased and decreased the fastest in Lesotho (EAPC 3.06; 95% CI: 2.5–3.63) and Denmark (EAPC -5.81; 95% CI: −6 to −5.63), respectively (Supplementary Table S4 and Figures 4D, 5D). In addition, the ASDR versus SDI curves for these countries were similar (*ρ* = −0.35, p < 0.001), with Nauru and Portugal remaining well above and below SDI expectations, respectively (Supplementary Figure S8).

These findings align with our regional burden analysis, revealing that countries with heavier disease burdens are primarily concentrated in Africa. In contrast, those with lighter burdens are mainly in Western Europe and North America.

### 3.5 Decomposition analysis for regional burden

**Figure 6:**
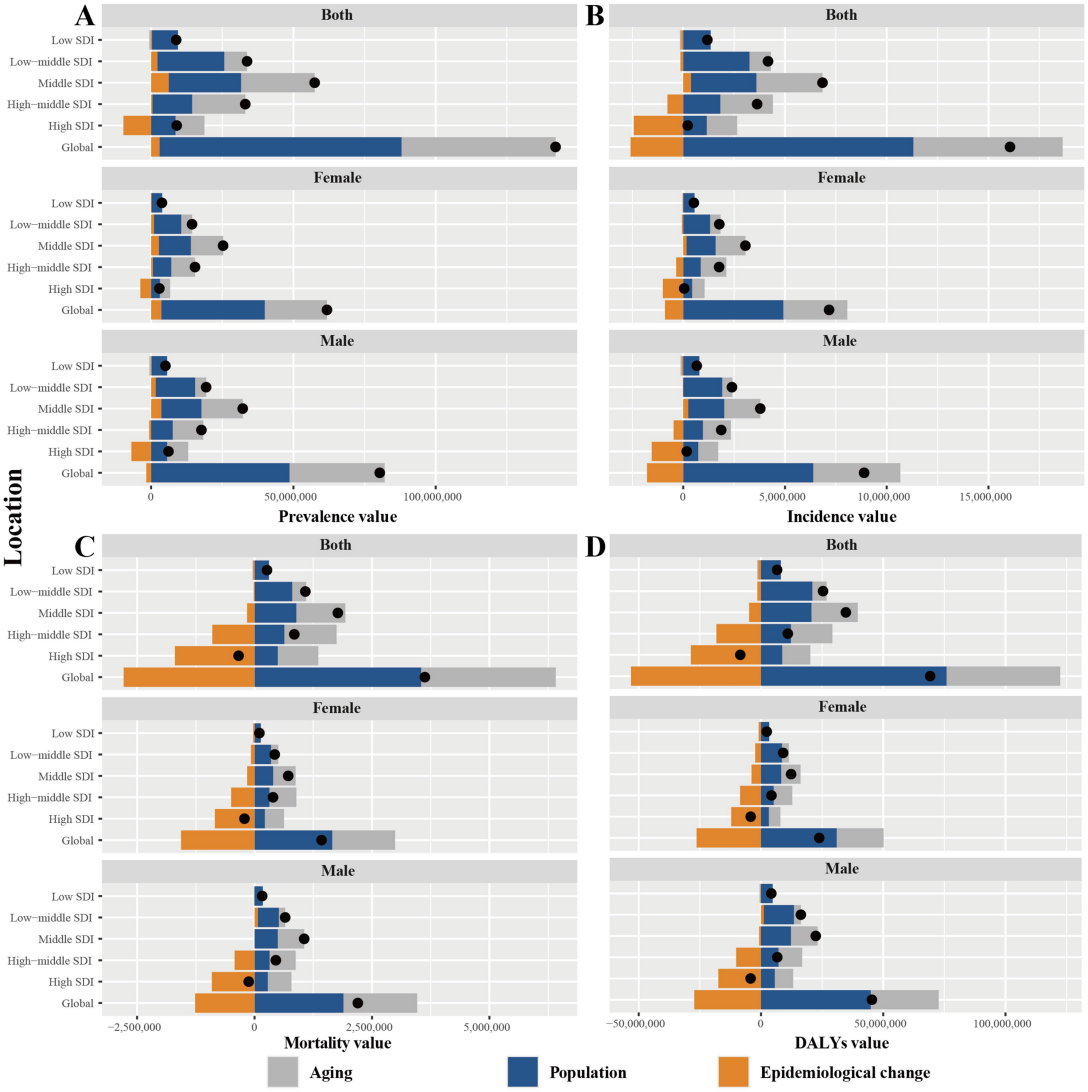
Decomposition analysis of changes in IHD prevalence, incidence, mortality, and DALYs by SDI region from 1990 to 2021. **(A)** Decomposition analysis of changes in prevalence of IHD; **(B)** Decomposition analysis of changes in incidence of IHD; **(C)** Decomposition analysis of changes in mortality of IHD; and **(D)** Decomposition analysis of changes in DALYs of IHD. IHD, ischemic heart disease; DALYs, Disability-Adjusted Life Years; SDI, sociodemographic index.

Figure 6 shows the impact of aging, population growth, and epidemiologic changes on the burden of IHD. At the global level, the prevalence, incidence, mortality, and DALYs of IHD increased significantly, and the positive impact of population growth was the greatest (59.84, 70.42, 97.81, and 109.65%, respectively). Compared with 1990, in 2021, there were 142 million new cases, with aging, population growth, and epidemiologic changes playing a positive role. There were approximately 16.06 million new cases of incidence, 3.62 million deaths, and approximately 69.2 million DALYs, with epidemiologic changes playing a negative role (−16.11, −76.89%, and −76.79%). When stratified by sex, the above phenomena did not differ significantly between men and women (Supplementary Table S5).

At the five SDI region levels, the effect of aging on IHD burden was most significant in the MSDI region, with positive contributions in prevalence, incidence, mortality and DALYs of 45, 47.46, 58.83, and 54.45%, respectively. The contribution of population growth to the burden of IHD was also relatively significant in the MSDI region, with 44.23, 46.96, 50.16, and 59.47% in prevalence, incidence, mortality and DALYs, respectively. Epidemiologic changes, on the other hand, played one of the few negative roles in the burden of IHD, specifically in incidence, mortality, and DALYs, and were most significant in the HSDI and HMSDI regions, particularly in the HSDI region (contributing −1093.3, 498.63, and 338.54%) (Supplementary Table S5 and Figure 6). Overall, IHD mortality and DALYs declined only in HSDI regions (Figures 6C,D), whereas the burden of IHD in MSDI regions increased substantially, driven by population growth and aging. Further stratifying by sex, we found that men generally faced a higher burden of the disease than women in all regions except for HSDI regions (Figure 6B).

### 3.6 Risk factors for regional burden

**Figure 7:**
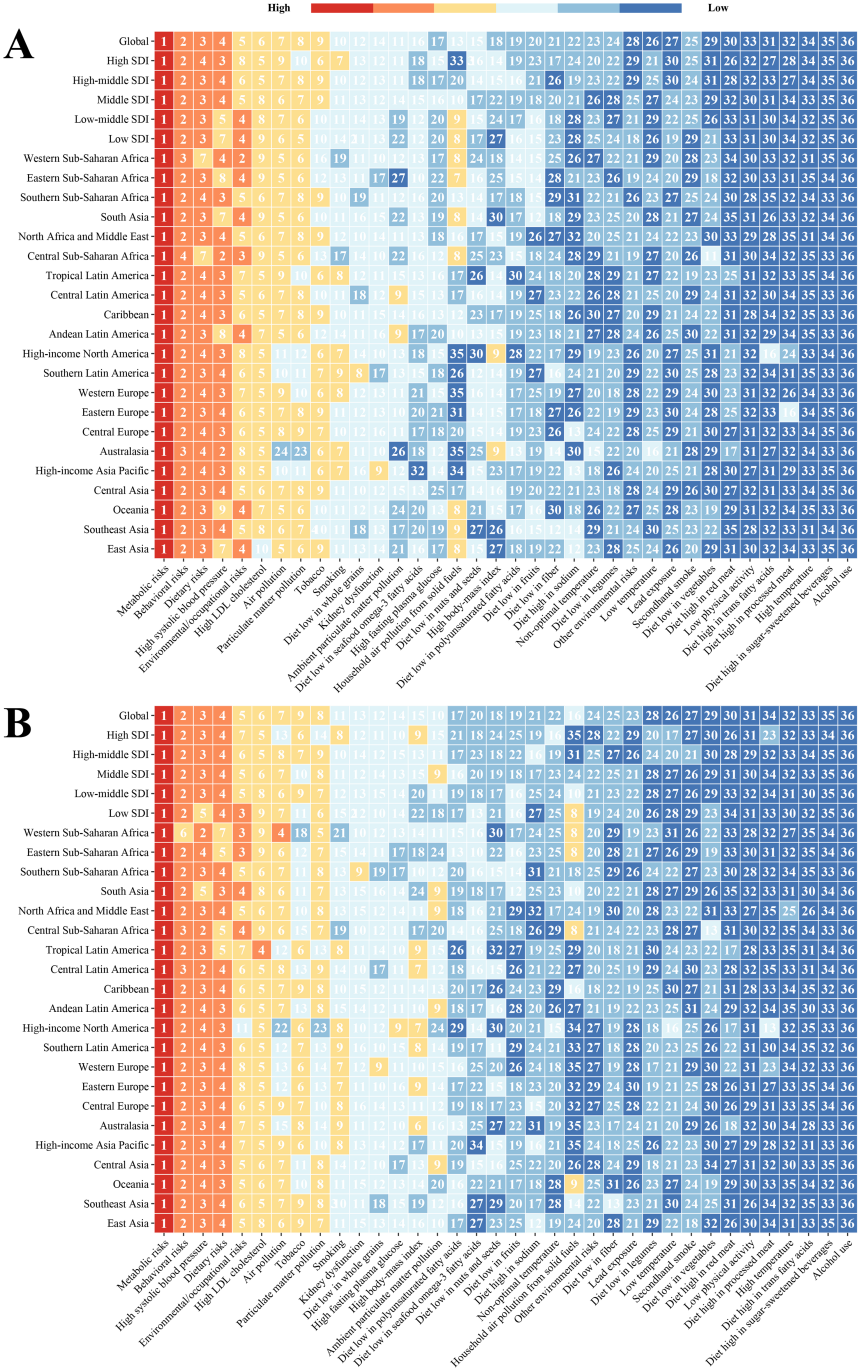
Ranking of risk factors contributing to the global and regional ASDR for IHD. **(A)** 1990; **(B)** 2021. IHD, ischemic heart disease; ASDR, age-standardized disability-adjusted life years rate.

We extracted 36 risk factors associated with IHD, analyzed their contribution to ASDR in 1990 and 2021, and ranked them at the global and regional levels according to the magnitude of their impact. Figure 7 shows that the major risk factors remained largely unchanged across global regions, with metabolic and behavioral risks consistently occupying the first and second positions. Notably, the third-ranked risk factor shifted from dietary risk to high systolic blood pressure.

To further demonstrate the changes in risk factors between 1990 and 2021, we selected the five risk factors (metabolic risk, behavioral risk, high systolic blood pressure, dietary risk, and environmental/occupational risks) that have the greatest overall impact on IHD-associated ASDR. We calculated their respective proportions of ASDR in the corresponding year relative to other risk factors and plotted them as bar graphs (Supplementary Figure S9). The results showed that metabolic factors contributed to the largest proportion of global IHD-related ASDR (12.8%, 1715.06/100,000 people). Further explored at the five SDI regional levels, metabolic and behavioral risks were greatest in the HSDI region in 1990 (13.8 and 10.4%) and similarly in 2021 (14.8 and 10.6%). The region with the greatest share of high systolic blood pressure changed from the HMSDI (9.4%) to HSDI (9.2%). For dietary risks, no changes were observed between the HMSDI (8.4%) and HSDI (8.4%). The ordering of the share of environmental/occupational risks did not change across the regions, which were all LSDI regions (8.1%). In addition, in 2021, at the 21 GBD geographic region level, the metabolic risk was largest in Australasia (16.2%), followed by Tropical Latin America (16.0%) and High-Income North America (15.9%), which is consistent with our findings in the five SDI regions. Behavioral risk, on the other hand, has the largest share in High-Income North America (11.1%). Meanwhile, we found that the proportion of IHD-associated ASDR due to high systolic blood pressure, dietary risks, and environmental/occupational risks was higher overall in Africa; however, when the proportion of IHD-associated ASDR due to high systolic blood pressure and dietary risks were the two influencing factors, Western Europe and Southern Latin America still ranked first (10.1 and 9.2%, respectively).

In addition, we present the proportion of ASDR burden of IHD attributed to the 12 level 2 risk factors in 1990 and 2021 (Supplementary Figure S10). In 1990, at the global level, dietary risks accounted for the largest proportion (21.0%); in 2021, the largest contributing factor became high systolic blood pressure. For the HSDI region, the risk of high systolic blood pressure decreased (−2.6%), but the risks provided by high body-mass index (+2.7%) and high fasting plasma glucose (+2.8%) increased significantly. For the HMSDI region, the proportion of dietary risks decreased significantly (−2.3%), the proportion of high systolic blood pressure increased slightly (+0.9%), and the changes in other risk factors were relatively small. The changes in risk factors in the MSDI region were basically consistent with those in the HMSDI region, mainly reflected in high systolic blood pressure (+2.3%) and dietary risks (−2.0%). The changing trends of risk factors in LMSDI and LSDI regions are basically the same, mainly with an increase in high systolic blood pressure and a decrease in dietary risks. In general, high systolic blood pressure is a core risk at the global level, especially in regions other than HSDI, where its contribution has increased rather than decreased; high body-mass index and high fasting plasma glucose are risks that have increased significantly in all regions, especially in HSDI, where the increase is the largest; the contribution of alcohol use to IHD is always negative, and there may be a potential “protective effect.” This reflects the fact that the contribution of different risk factors to ASDR is unique across regions, and exploring the implications and complex causes is essential to implement targeted interventions and surveillance in these regions.

### 3.7 Forecast analysis for the global burden to 2045

**Figure 8:**
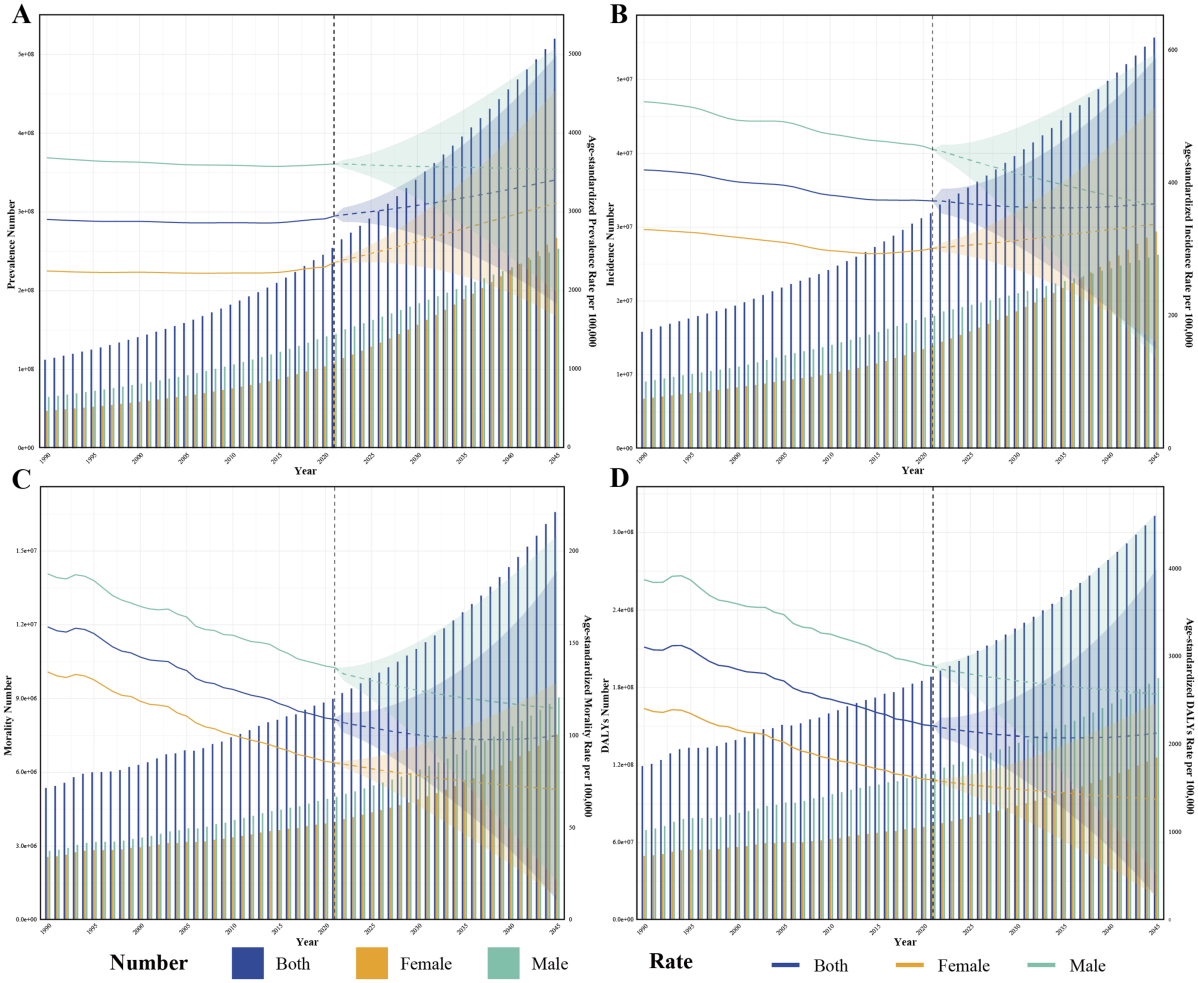
Forecasts of the global burden of ischemic heart disease from 2022 to 2045. **(A)** Number of cases and ASR projections for prevalence; **(B)** Number of cases and ASR projections for incidence; **(C)** Number of cases and ASR projections for mortality; **(D)** Number of cases and ASR projections for DALYs. ASR, age-standardized rate; DALYs, disability-adjusted life-years; ASPR, age-standardized prevalence rate; ASIR, age-standardized incidence rate; ASMR, age-standardized mortality rate; ASDR, age-standardized disability-adjusted life years rate.

Figure 8 illustrates the projected number of cases and ASRs for global IHD prevalence, morbidity, mortality, and DALYs from 2022 to 2045, where we expect the burden of IHD to increase. Specifically, the absolute number of IHD prevalent cases, incident cases, deaths and DALYs is expected to rise globally. In contrast, ASIR, ASMR, and ASDR, with the exception of ASPR, are projected to gradually decline. By 2045, ASPR, ASIR, ASMR, and ASDR were projected to reach 3412.12 per 100,000 people, 368.99 per 100,000 people, 99.86 per 100,000 people, and 2572.19 per 100,000 people (Supplementary Table S7). After further stratification by sex, in addition to ASMR and ASDR, ASPR and ASIR showed a slowly increasing trend among women in IHD. Although the burden of disease will still be greater for men than for women by 2045, there is still the possibility that the burden for women will inversely exceed that for men, and the potential dangers of this should not be ignored.

## 4 Discussion

IHD is a major cardiovascular disease worldwide with a high incidence, slow progression, and high risk of mortality. As a crucial public health issue, the dynamic changes in the burden of IHD have attracted extensive academic attention (28). This study utilizes the GBD 2021 database to provide an in-depth analysis of the global, regional, and national burden of IHD. Our results contrasted with those from the GBD 2019 database, underscoring the need for further evaluations with updated data (11). The absolute number of prevalent cases, incident cases, deaths, and DALYs caused by IHD increased worldwide between 1990 and 2021. However, when evaluated using ASR, the ASIR, ASMR, and ASDR have demonstrated some progress. Notably, the global ASPR increased significantly after 2013, particularly between 2020 and 2021 (from 2912.71–2946.38 per 100,000), likely linked to the coronavirus disease 2019 (COVID-19) pandemic’s impact on IHD prevalence (33–35). In 2021, LMSDI regions recorded the highest ASPR, ASIR, ASMR, and ASDR, whereas HSDI regions exhibited the lowest disease burden, highlighting significant disparities across socio-economic areas. The reduction in ASPR of IHD in the HSDI regions is likely due to better healthcare access, efficient public health strategies, and increased awareness of IHD, promoting healthier lifestyles (36). Conversely, LMSDI regions struggle with low economic resources, limited healthcare access, and dietary and lifestyle changes that may increase IHD risk (15, 37). Central Asia has the highest ASMR and ASDR, influenced by unhealthy dietary habits, poor healthcare access, and inadequate chronic disease management (38, 39). In North Africa and the Middle East, high ASPR and ASIR may be due to uneven economic development and underutilization of medications for hypertension control (40). These challenges are exacerbated by limited healthcare quality and poor disease management, posing significant obstacles to effective healthcare and public health policy implementation (41, 42). Additionally, high rates of sickle cell traits in parts of Africa are closely linked to thromboembolic events (43). Notably, the High-Income Asia Pacific region has the lowest disease burden, contrasting sharply with the higher burdens in South and Central Asia. This disparity reflects the positive impact of socio-economic development and healthcare policies. South and Central Asia face widespread healthcare resource shortages and income inequality, whereas the High-Income Asia Pacific region has reduced risks through effective prevention measures and improved health education (7, 44, 45). This highlights the effectiveness of primary prevention, improvements in acute coronary syndrome management, and strong secondary prevention strategies. In these areas, hypertension and cholesterol management are more effective, and the appropriate use of antiplatelet and statin medications has lowered recurrence risks. Advances in reperfusion strategies, such as coronary interventions, have significantly reduced disability and mortality rates associated with acute myocardial infarction.

Our research findings indicate that the burden of IHD has considerably increased with age between 1990 and 2021. Notably, the crude rate of prevalence, incidence, morality and DALYs in the older adult population, particularly those aged ≥95 years, surpass those in other age groups. This trend may be linked to the cumulative physiological effects of cardiovascular risk factors and age-related structural and functional changes in the circulatory system, including the progression of atherosclerosis, endothelial dysfunction, and heart failure (46). As individuals age, arterial stiffness and decreased elasticity occur, compounded by the long-term impacts of hypertension, diabetes, and smoking, which accelerate the risk of coronary artery occlusion and lead to a higher incidence of ischemic events (47, 48). Additionally, the differences in IHD burden by sex may arise from varying exposure frequencies to risk factors. Generally speaking, men are more likely to smoke and drink, both significant contributors to atherosclerosis. Moreover, sex-based differences in cardiovascular reactivity and hormone levels may result in distinct pathological mechanisms and clinical manifestations of IHD, particularly evident in underdeveloped regions (49–51). This also explains the rising disease burden among men in MSDI and LMSDI regions despite an overall decline in ASMR and ASDR for the general population.

Our study also reveals that the burden of IHD primarily affects individuals aged ≥60 years, particularly in MSDI and LMSDI regions. There are two main causes for this tendency: rapid population growth and increased aging, which continuously elevate the number of persons at high risk for IHD, raising the disease’s overall impact. Additionally, the epidemiological transition in these areas plays a critical role, with the shift from infectious diseases to chronic non-communicable diseases leading to an increased incidence of IHD (52, 53). Meanwhile, rapid economic development and changes in lifestyle are contributing to the rising burden of IHD. The widespread adoption of Western dietary habits (high fat and sugar intake) and a sedentary lifestyle have resulted in the growing prevalence of IHD risk factors, including obesity, hypertension, high cholesterol, and diabetes, particularly during periods of economic transition (54, 55). Furthermore, effective cardiovascular care, especially in terms of rapid diagnosis and intervention during acute cardiovascular events, as well as long-term rehabilitation support, is often less accessible in a lot of low- and middle-income nations, escalating cardiovascular event-related mortality and morbidity (8, 56). In contrast, HSDI and HMSDI regions can alleviate the disease burden through effective policy measures (such as smoking control, alcohol limitation, and promotion of healthy lifestyles), the use of advanced medications and treatments, and systematic chronic disease management strategies (57–59). It is noteworthy that there is a significant increase in the number of prevalent and incident cases of IHD among those aged 45–49 years compared with that among those aged <45 years, especially in men. Therefore, it is recommended that health education related to IHD be initiated and strengthened from the age of 45 years for early prevention and intervention.

Furthermore, the examination of risk factors for IHD shows that metabolic risks, behavioral risks, high systolic blood pressure, dietary risks, and environmental/occupational risks are significant contributors to disability and mortality globally and regionally. These factors’ effects change depending on the SDI region, necessitating tailored prevention strategies. In high-income regions with elevated metabolic and behavioral risks, health initiatives promoting regular physical activity can help reduce obesity-related cardiovascular disease risks (60). Conversely, in low- and middle-income regions, environmental/occupational risks and air pollution pose major health threats. Measures, such as strengthening environmental regulations, enhancing public awareness, and promoting renewable energy, can help mitigate these burdens (61, 62). After further grading the risk factors, we found that the main factors causing the increase in metabolic risks were high body-mass index, high fasting plasma glucose and high LDL cholesterol. Among them, high body-mass index and high fasting plasma glucose were the most significant major contributors to the increase in ASDR caused by IHD, especially in HSDI areas. This suggests that in HSDI regions, public health strategies should prioritize obesity and blood sugar management to curb the prevalence of metabolic syndrome, while in MSDI, LMSDI and LSDI regions, blood pressure management and environmental governance should continue to be strengthened. At the same time, formulating dietary guidelines that are in line with local culture to improve eating habits and reduce salt and cholesterol intake is equally important for lowering blood pressure and cholesterol levels (63). In addition, tobacco, a traditional risk factor for IHD, has a global decline in its related ASDR in 2021. This trend is highly consistent with the implementation of the MPOWER tobacco control policy led by the WHO (such as tobacco tax increases and smoke-free environment legislation), and the decline is particularly significant in HSDI regions with strong policy enforcement (−1.9%) (64). However, the uneven economic development between regions suggests that there are barriers to policy penetration, and differentiated taxation is needed to consolidate existing achievements in the future (65). We also found that alcohol use was the only contributor to the decrease in ASDR of IHD. In recent years, several studies have shown that there is an inverse correlation between alcohol consumption and IHD risk. Carr’s research team proposed that moderate drinking can significantly reduce the risk of IHD compared with no drinking, but the long-term impact of drinking on IHD risk still requires extensive cohort studies to provide conclusive evidence (66). Decomposition analyses showed that population growth and aging contributed significantly to the increased burden of IHD, especially in the LMSDI and MSDI regions. In contrast, the epidemiologic transition only slightly reduced the burden in the HSDI and HMSDI regions, underscoring the critical impact of the level of social development on the burden of IHD. In general, the positive effects of population growth and aging are mostly concentrated in regions with lower and higher SDI. For regions with lower SDI, due to the relatively backward level of economic development and lower efficiency of resource utilization, large population growth will lead to over-utilization of natural resources and destruction of the ecological environment; at the same time, due to the fact that the degree of improvement of social services in the region is often insufficient, the growth of employment opportunities, the increase in educational resources, and the supply of medical resources are unable to satisfy the needs of large population growth (67). As for the higher SDI regions, the high level of medical resources and affluent family environments have led to a generally high level of health, which will lead to a further increase in the aging population, and social security expenditures, such as medical insurance and retirement wages, will increase significantly. In addition, as the proportion of the older adult continues to rise, the socio-economic structure and the related industrial chain (such as health care products) will undergo a certain degree of transformation. Because of the relatively high social status of the older adult, this impact may be more significant.

It is crucial to acknowledge that advancements in IHD treatment and interventions have significantly contributed to alleviating the disease burden. In the early 1980s, patients with acute IHD primarily relied on thrombolytic therapy or conservative treatments, which resulted in low survival rates and a substantial DALY burden (68). Although antiplatelet agents and statins have been widely used to prevent thrombosis and atherosclerotic plaque formation in high-risk populations, the effectiveness of combination therapies remains inadequate (69, 70). The introduction of percutaneous coronary intervention and drug-eluting stents in the 2000s markedly improved IHD treatment outcomes. The launch of the proprotein convertase subtilisin kexin9 inhibitor Evolocumab in 2015, when used alongside statins, further enhanced lipid-lowering efficacy, leading to a substantial reduction in DALYs linked to “high LDL cholesterol.” Despite these advancements, the burden of IHD continues to increase, underscoring the need for new therapeutic targets to effectively prevent the onset and progression of IHD (71). Moreover, some studies have pointed out that COVID-19 infection may increase the risk of myocardial infarction and thrombosis by inducing myocardial injury, proinflammatory state, and endothelial dysfunction (72–74). We speculate that this may be one of the factors leading to the increase ASPR and ASIR of IHD globally in 2020–2021, but further research is still needed to distinguish the direct and indirect effects of the epidemic. Based on this, in the post-COVID-19 era, there is an urgent need to strengthen the prevention and management strategies of IHD to address these ongoing health challenges. Finally, we forecast that by 2045, the absolute number of IHD-related prevalent cases, incident cases, deaths, and DALYs will rise each year; however, ASMR and ASDR are projected to decline gradually. This trend suggests that the burden of IHD will face new challenges, particularly the interplay between population growth, aging trends, and advances in medical technology. It is the advanced level of healthcare in developed countries that is exacerbating the growth of an aging population and thus raising the number of people with the disease and its incidence. Consequently, as populations age, the focus of the burden of disease will shift to older persons. In this regard, countries need to take advantage of their geographic and economic strengths, pay close attention to changes in population dynamics, and better balance the allocation of healthcare resources in order to cope with the impact of aging societies in the future.

This study has two significant strengths. First, it employs the latest data from GBD 2021, ensuring the information’s timeliness and enhancing the accuracy and reliability of the research. Secondly, it achieves notable advancements in both breadth and depth by utilizing a comprehensive array of analytical methods, deepening our understanding of its epidemiology and offering a solid basis for optimizing public health strategies and efficiently allocating healthcare resources. However, the study also has some limitations. The impact of IHD may be underestimated in some low-income nations due to inadequate healthcare quality, which can lead to misdiagnosis and underdiagnosis. Secondly, some data from certain countries are based on comprehensive modeling by the GBD team rather than actual reports, which may introduce some errors. Nonetheless, the GBD study has implemented various strategies to mitigate these errors and has gained global recognition for its authority and reliability (29). Additionally, due to the high collinearity of multiple factors during the pandemic (such as medical disruptions and increased aging), this study was unable to quantify the independent contribution of COVID-19 and other driving factors using existing data, and further research combining individual-level and sensitivity analyses will be needed in the future. Finally, the GBD database may exhibit some lag due to the complexities of data collection, modeling methods, and disease trends. Future research should incorporate the latest epidemiological surveys to further validate our findings.

## 5 Conclusion

In summary, from 1990 to 2021, while ASMR and ASDR for IHD have decreased, the absolute burden of disease is still rising, especially among men. Significant disparities persist between developed and developing regions. The burden of IHD will increase annually due to population growth and aging by 2045. Low-income countries must tackle the risks posed by unhealthy diets and environmental pollution. Conversely, high-income countries should address the burdens associated with aging and high metabolic risks while ensuring effective healthcare resource allocation. These findings highlight the various risks and challenges in IHD management. Going forward, the development of new intervention-oriented policies that incorporate age- and sex-specific measures will be critical if we are to succeed in reducing the global burden of IHD.

## Data Availability

The datasets presented in this study can be found in online repositories. The names of the repository/repositories and accession number(s) can be found at: https://vizhub.healthdata.org/gbd-results/.

## Footnotes

^1^ https://vizhub.healthdata.org/gbd-results/
