# Supplementary material for: Global, regional, and national burden and risk factors of ischemic heart disease, 1990–2021: an analysis of the global burden of disease study
Source: Front Public Health. 2025 Apr 25;13:1563631. doi: 10.3389/fpubh.2025.1563631 (PMC12061725; doi:10.3389/fpubh.2025.1563631)
Supplement: Supplementary file 1 [file Data_Sheet_1.pdf]

Figure S1 Crude incidence rate of IHD by sex, age group, and SDI, 1990 and 2021. Abbreviations: IHD, ischemic heart disease; SDI, sociodemographic index.

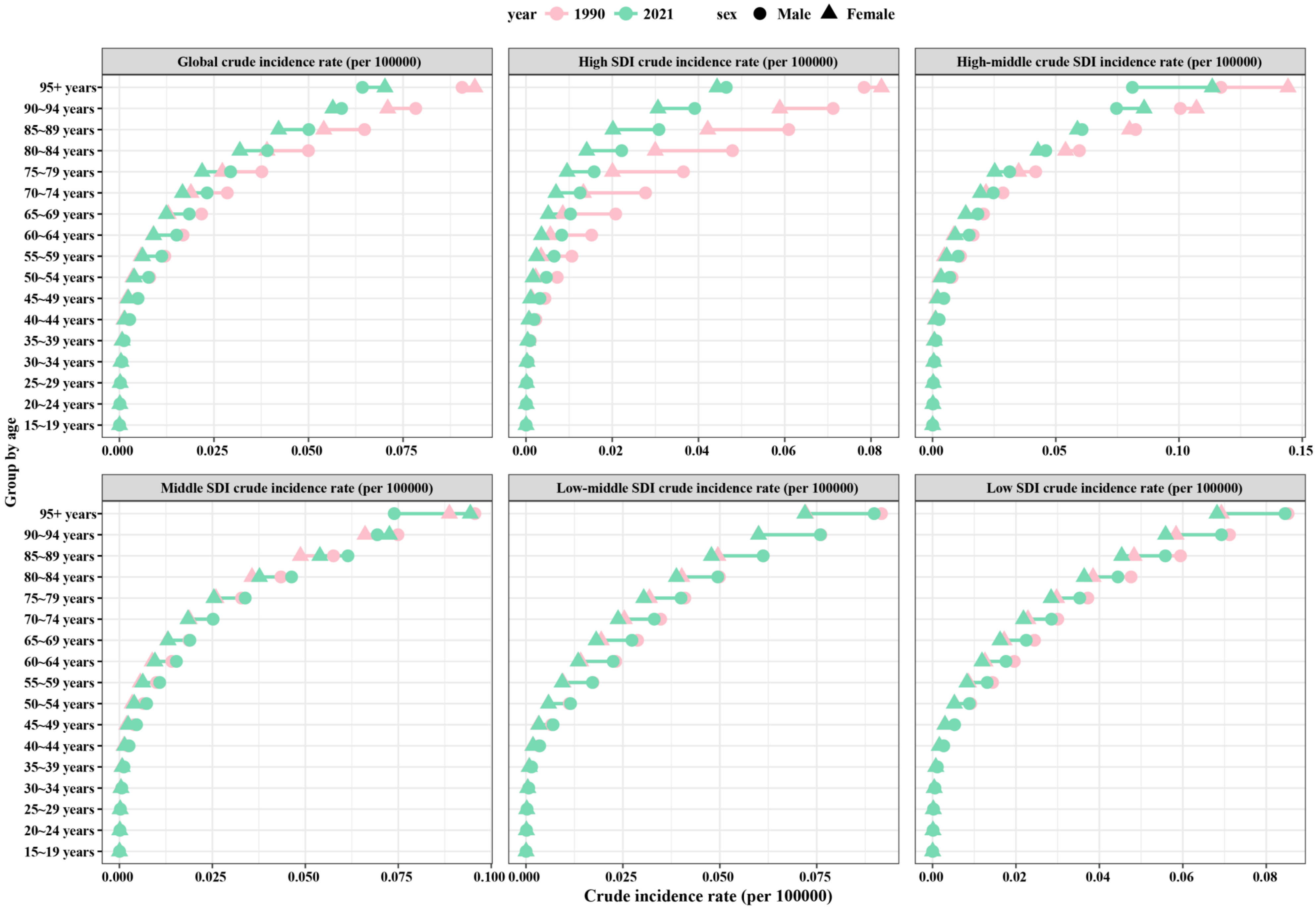

Figure S2 Crude mortality rate of IHD by sex, age group, and SDI, 1990 and 2021. Abbreviations: IHD, ischemic heart disease; SDI, sociodemographic index.

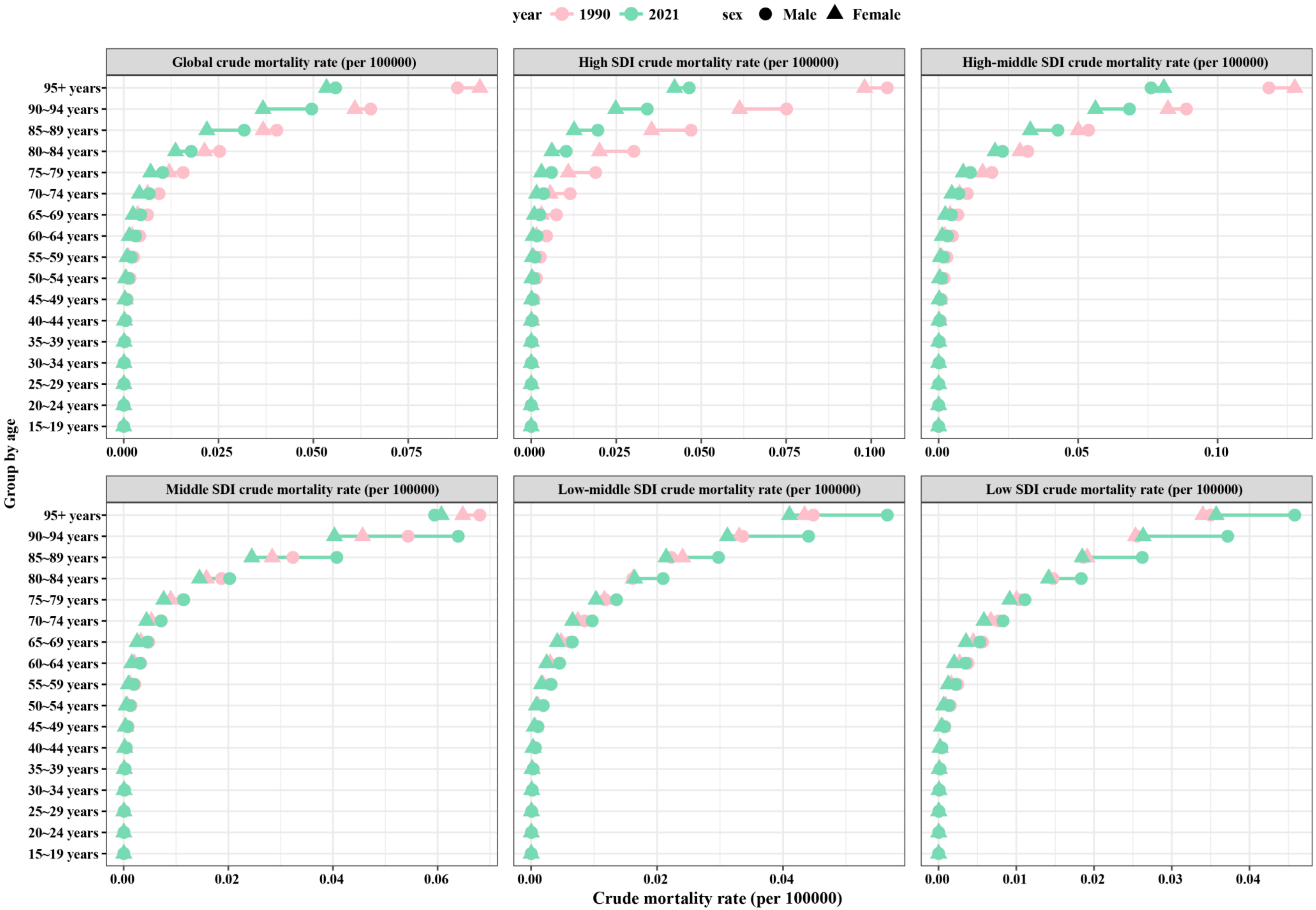

Figure S3 Crude DALYs rate of IHD by sex, age group, and SDI, 1990 and 2021. Abbreviations: IHD, ischemic heart disease; DALYs, Disability-Adjusted Life Years; SDI, sociodemographic index.

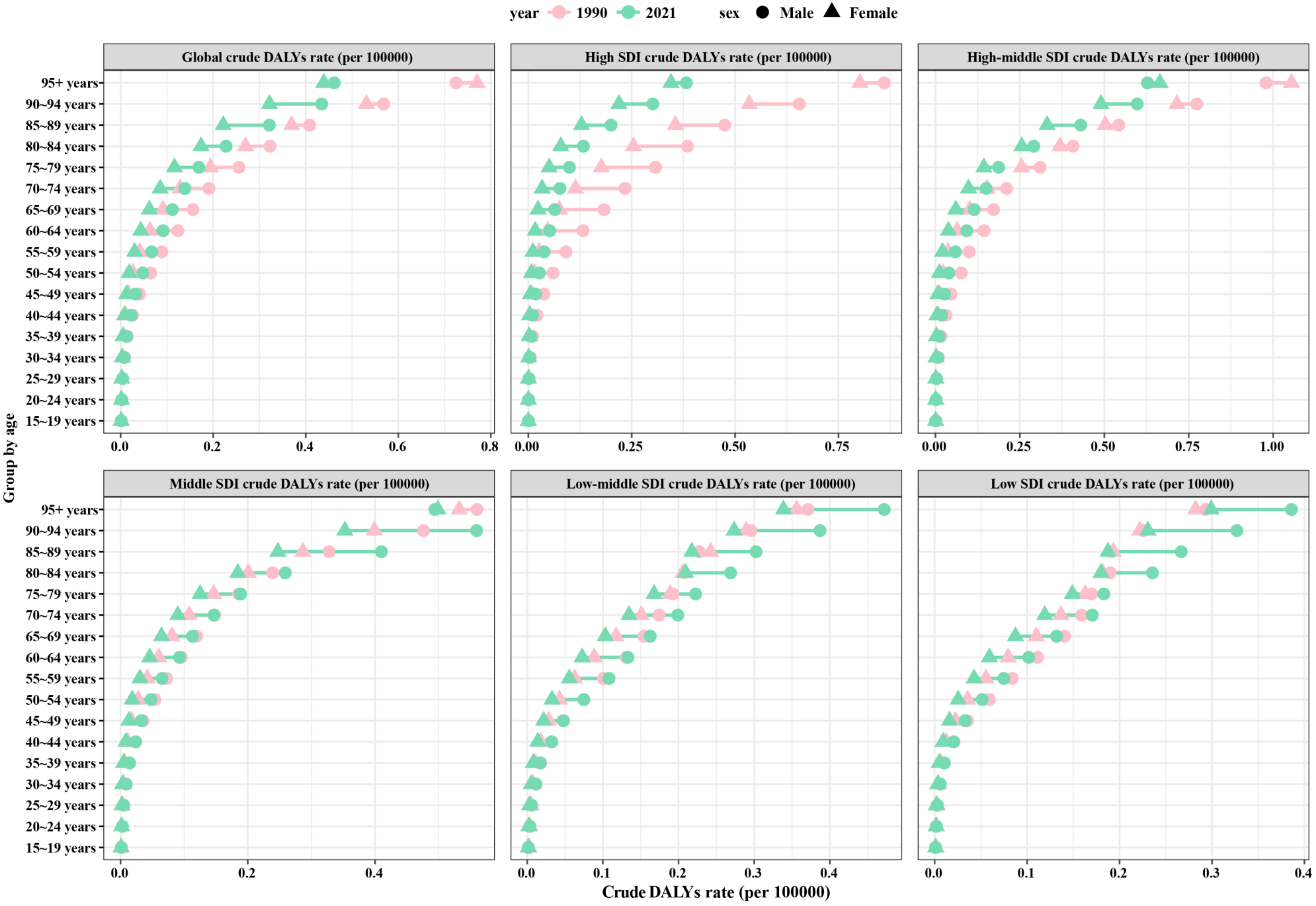

Figure S4 Relationship between SDI and the burden of IHD in 21 GBD regions in 2021. (A) ASPR for ischemic heart disease; (B) ASIR for ischemic heart disease; (C) ASMR for ischemic heart disease; and (D) ASDR for ischemic heart disease. Expected values based on sociodemographic indices and disease rates for all locations are shown as black lines. Abbreviations: IHD, ischemic heart disease; ASPR, age-standardized prevalence rate; ASIR, age-standardized incidence rate; ASMR, age-standardized mortality rate; ASDR, age-standardized disability-adjusted life years rate; DALYs, Disability-Adjusted Life Years; GBD, Global Burden of Diseases, Injuries, and Risk Factors Study; SDI, sociodemographic index.

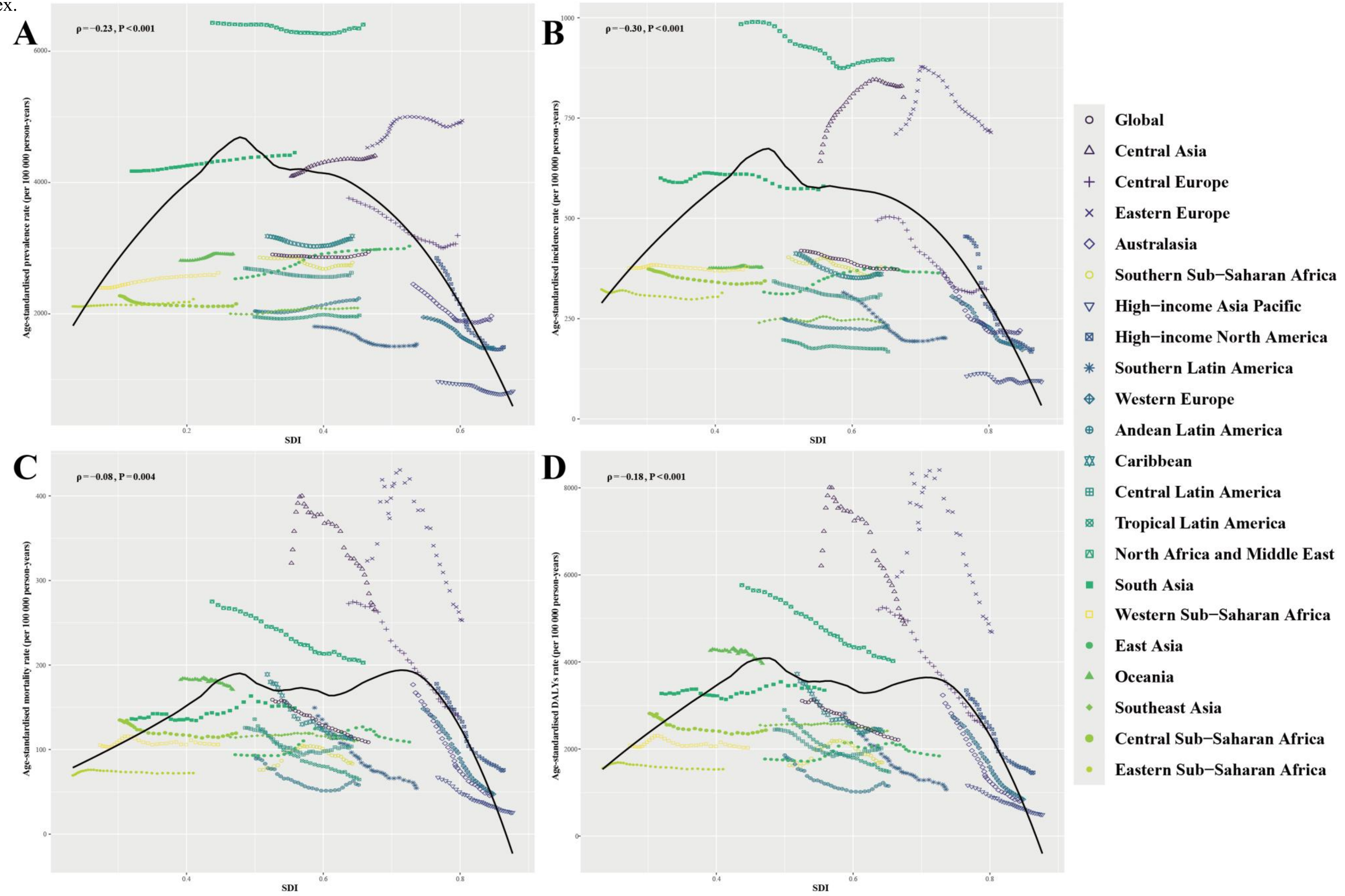

Figure S5 Relationship between SDI and the burden of ASPR for IHD in 204 countries and territories in 2021. IHD, ischemic heart disease; ASPR, age-standardized prevalence rate; SDI, sociodemographic index.

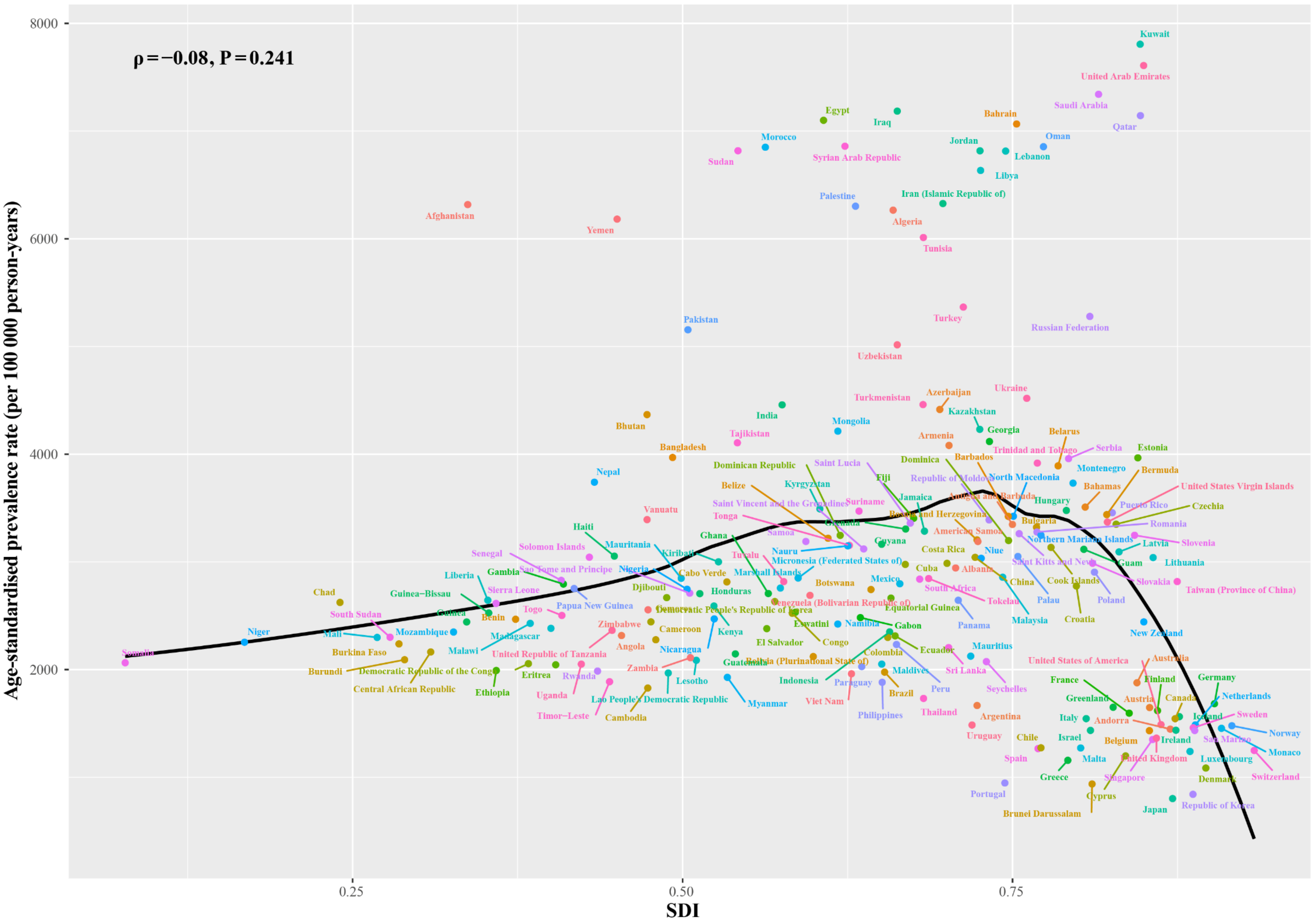

Figure S6 Relationship between SDI and the burden of ASIR for IHD in 204 countries and territories in 2021. IHD, ischemic heart disease; ASIR, age-standardized incidence rate; SDI, sociodemographic index.

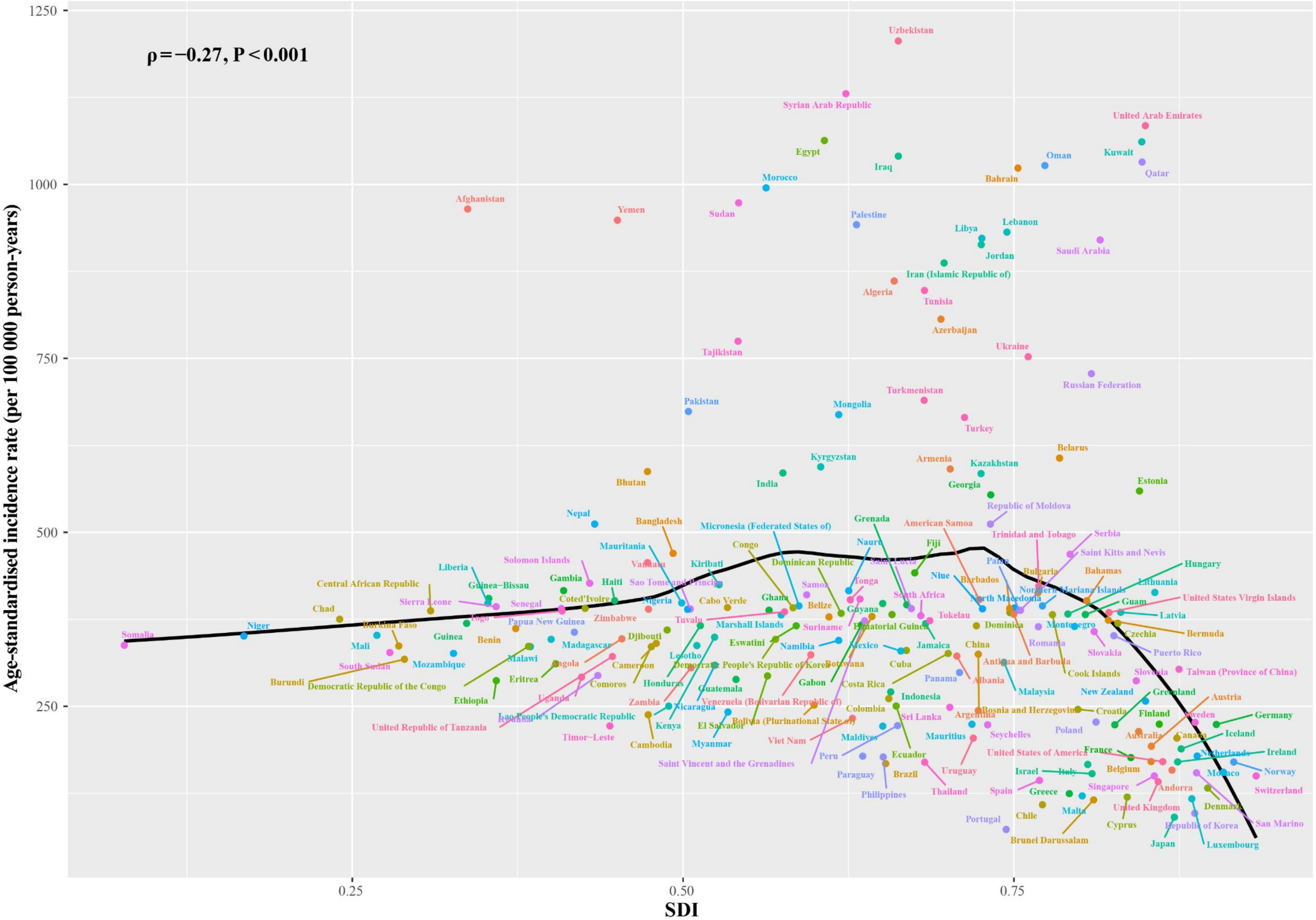

Figure S7 Relationship between SDI and the burden of ASMR for IHD in 204 countries and territories in 2021. IHD, ischemic heart disease; ASMR, age-standardized mortality rate; SDI, sociodemographic index.

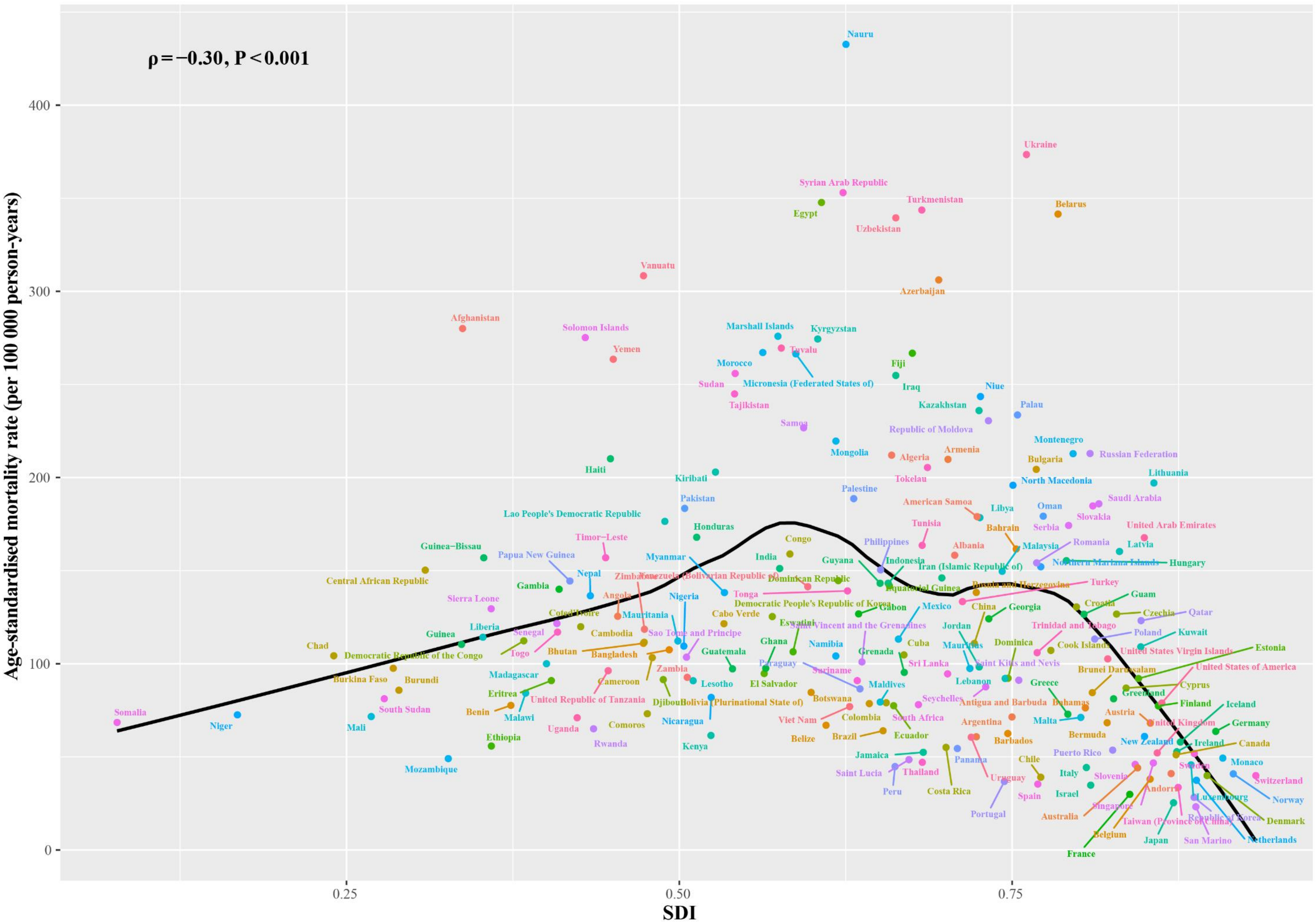

Figure S8 Relationship between SDI and the burden of ASDR for IHD in 204 countries and territories in 2021. IHD, ischemic heart disease; ASDR, age-standardized disability-adjusted life years rate; SDI, sociodemographic index.

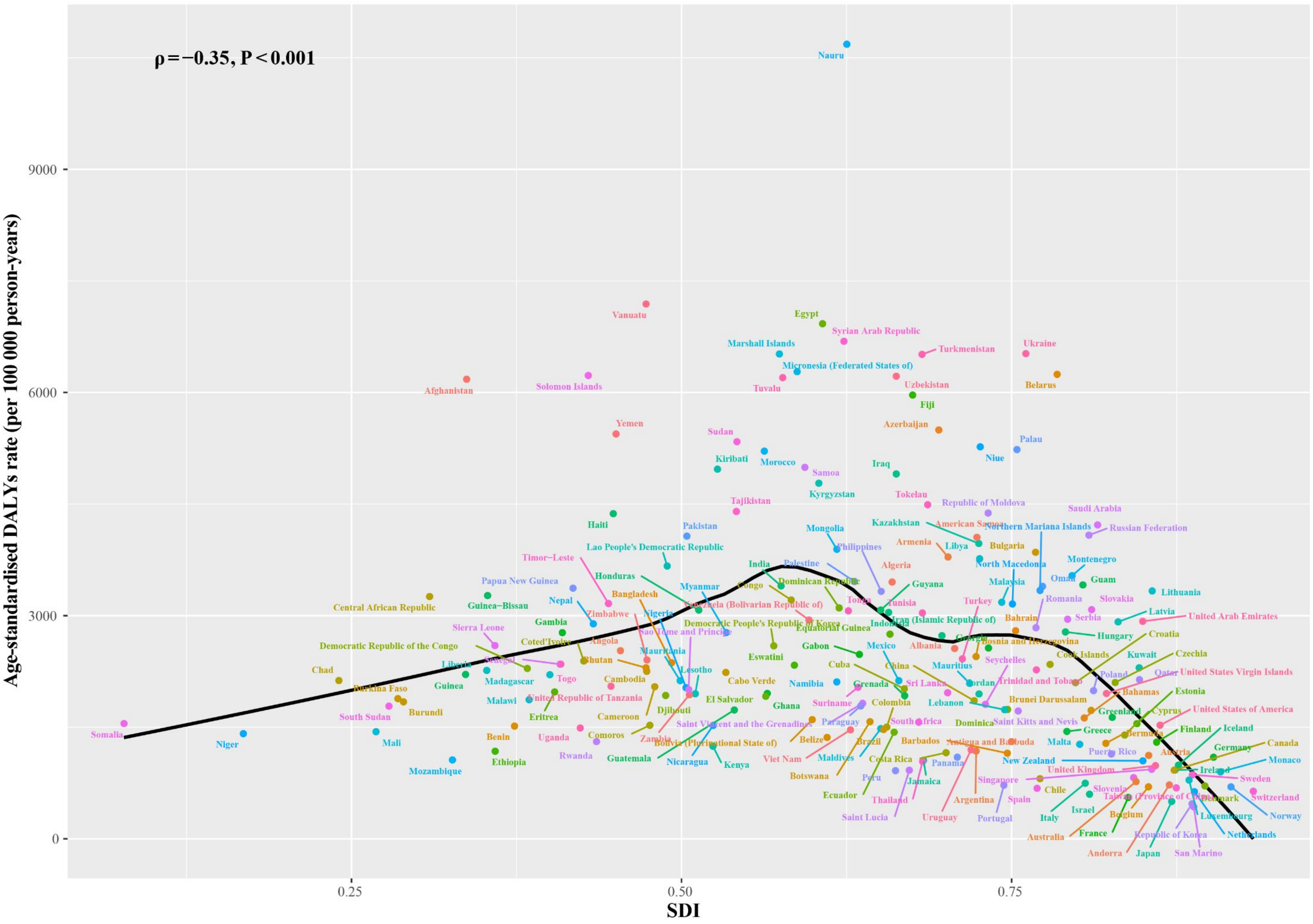

Figure S9 Share of the top five risk factors contributing to the ASDR burden of IHD in 1990 and 2021 in the corresponding year by global, 5 SDI regions, and 21 GBD regions.

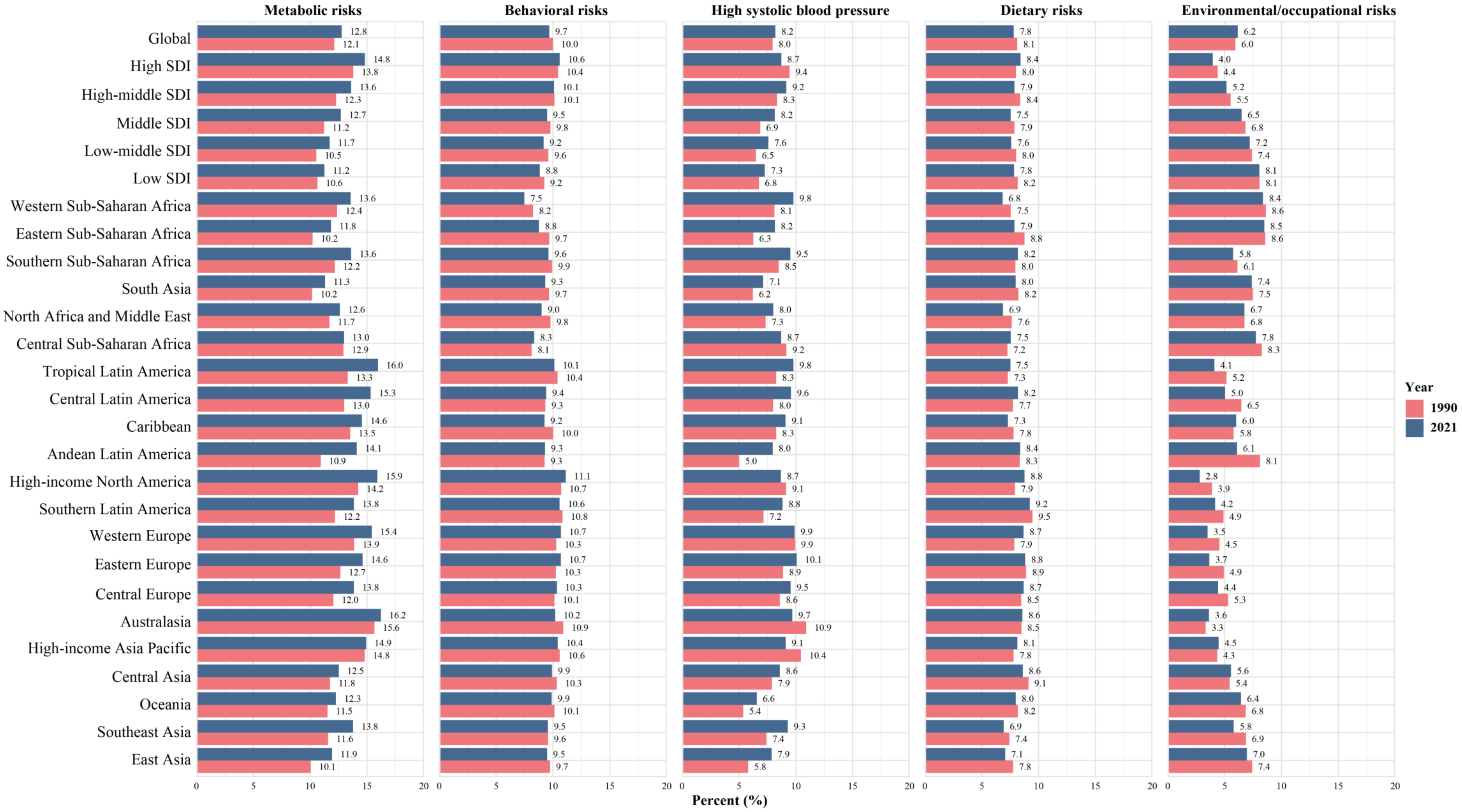

Figure S10 Contribution of different level 2 risk factors to ASDR for IHD in 1990 (A) and 2021 (B) by global and 5 SDI regions.

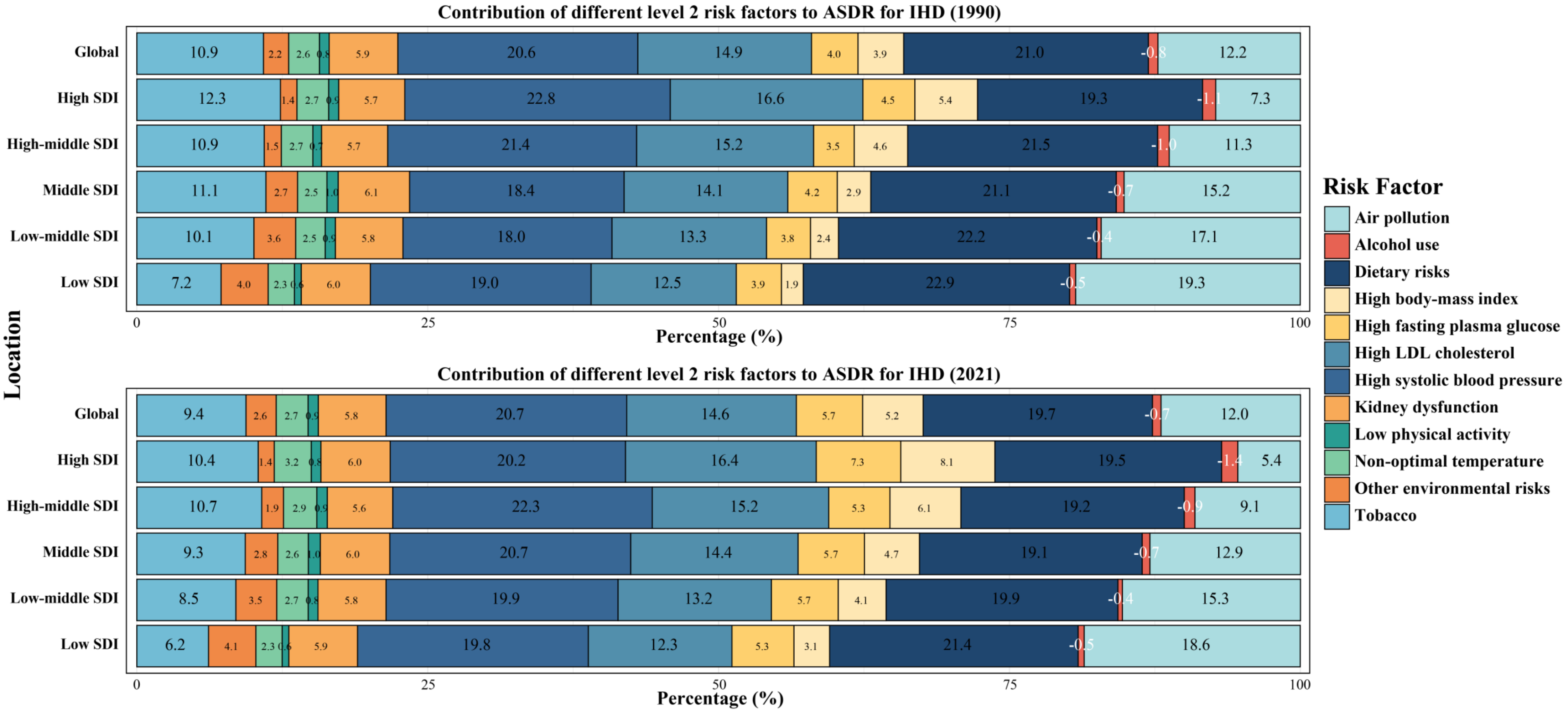

Table S1 The prevalence of ischemic heart disease cases and age-standardized rate at national level in 1990 and 2021, with EAPC (1990-2021).

Location	1990		2021		EAPC (95% CI)
	Number	ASR	Number	ASR	
Afghanistan	456839.8 (426706.6,486638.6)	6826.45 (6396.58,7275.06)	584027.7 (535534.4,639114.2)	6317.65 (5785.3,6886.79)	-0.3 (-0.34,-0.27)
Albania	63873.4 (56785.2,71356.5)	3156.95 (2786.95,3530.86)	129236.8 (113576.4,147069.1)	2943.69 (2607.84,3312.44)	-0.22 (-0.26,-0.18)
Algeria	788066 (740325.8,846697.1)	6734 (6344.73,7187.13)	2184384.4 (1996825.8,2394662.7)	6265.94 (5755.97,6836.74)	-0.34 (-0.38,-0.3)
American Samoa	601.9 (548.3,661.7)	3042.12 (2793.37,3334.1)	1420.6 (1271.5,1588.7)	3189.2 (2870.61,3544.63)	0.14 (0.13,0.14)
Andorra	935.4 (825.7,1055.7)	1612.91 (1424.51,1820.7)	2217.9 (1941,2516)	1447.15 (1267.07,1640.49)	-0.58 (-0.65,-0.5)
Angola	84597 (76821.5,92848.9)	2394.25 (2175.09,2652.14)	254517.5 (227321.6,287276.3)	2316.3 (2069.3,2609.2)	-0.14 (-0.16,-0.12)
Antigua and Barbuda	1684.4 (1539.4,1857)	3130.75 (2864.71,3466.82)	3551.5 (3172.1,3979.7)	3347.62 (2995.31,3737.23)	0.24 (0.23,0.25)
Argentina	638248 (583732.9,701215)	1977.98 (1811.7,2166.45)	932786.4 (837288.3,1049343.9)	1666.56 (1499.65,1871.35)	-0.73 (-0.82,-0.64)
Armenia	98630.4 (91062.2,107055.1)	3850.37 (3557.26,4182.44)	178262.8 (159468.7,197849.5)	4081.36 (3674.11,4505.4)	0.25 (0.22,0.28)
Australia	438617.9 (407006.6,476441.5)	2247.86 (2092.46,2437.69)	847760.6 (760154.9,952136)	1877.69 (1685.68,2103.19)	-0.65 (-0.84,-0.45)
Austria	183015.5 (161134,207715)	1552.46 (1379.85,1749.24)	288715.3 (256490.2,322309.1)	1647.51 (1470.77,1832.73)	0.22 (0.17,0.26)
Azerbaijan	191713.1 (177984.7,207894.9)	4081.71 (3790.91,4400.17)	414985.4 (377048.9,455225.7)	4415.23 (4047.75,4805.9)	0.36 (0.32,0.39)
Bahamas	4938.4 (4509.4,5411)	3264.6 (2976.23,3578.75)	14079.2 (12560.7,15678.3)	3508.61 (3146.7,3904.39)	0.25 (0.23,0.27)
Bahrain	12183.5 (11311,13117.7)	7213.28 (6732.62,7764.33)	66751.4 (59153.5,74791.3)	7066.05 (6420.79,7782.27)	-0.19 (-0.23,-0.15)
Bangladesh	1736104.1 (1597072.7,1894271)	3849.18 (3532.01,4207.36)	5436530.2 (4923888.9,6006969.3)	3969.58 (3596.91,4375.69)	0.16 (0.12,0.2)
Barbados	9005.5 (8194,9916.3)	3083.11 (2811.88,3386.1)	17565.2 (15636.7,19763.5)	3422.85 (3064.35,3832)	0.35 (0.3,0.4)
Belarus	490962 (452596.2,533116)	3852.15 (3553.41,4175.18)	627086.8 (569924.3,683129)	3891.64 (3553.03,4226.16)	0.11 (0.02,0.21)
Belgium	307940 (278945.3,339871.7)	2020.42 (1836.81,2224.5)	321065.4 (280403.8,365749.3)	1432.88 (1254.81,1632.08)	-1.27 (-1.46,-1.09)
Belize	2664.4 (2424,2925.2)	2864.23 (2595.97,3153.71)	9508.6 (8498.7,10596.6)	3219.61 (2885.16,3604.55)	0.37 (0.35,0.39)
Benin	44181 (40102,48826.6)	2282.9 (2063.92,2527.89)	123597 (110365.6,138434)	2467.27 (2189.84,2771.31)	0.25 (0.22,0.27)
Bermuda	1983.7 (1812.3,2174.4)	3210.83 (2931.2,3521.46)	4643.9 (4165.5,5182.7)	3439.03 (3079.38,3832.95)	0.25 (0.23,0.26)
Bhutan	9209.2 (8427.7,10062.6)	4101.68 (3742.61,4487.13)	25870.3 (23133.8,28751.5)	4367.48 (3913.37,4869.9)	0.19 (0.18,0.2)
Bolivia (Plurinational State of)	62261.7 (56355.3,68515.7)	1989.73 (1803.93,2192.15)	191868.7 (170187.7,215282.3)	2120.91 (1891.18,2372.51)	0.22 (0.19,0.26)
Bosnia and Herzegovina	120514.2 (108536.4,135209.9)	3068.66 (2763.66,3421.07)	198762.2 (174654.9,223016)	3204.01 (2832.71,3582.71)	0.11 (0.07,0.15)
Botswana	12765.5 (11553.9,14250.2)	2429.19 (2204.97,2707.45)	38444.1 (34081.2,42671.5)	2743.35 (2440.89,3073.51)	0.36 (0.33,0.4)
Brazil	1724721.5 (1475061,1976421.8)	1954.18 (1672.8,2253.55)	4966552.3 (4177586.9,5843929.6)	1976.74 (1669.26,2325.71)	0.07 (0.04,0.1)
Brunei Darussalam	1113.6 (995.8,1242.3)	1118.7 (1000.86,1255.59)	3109.7 (2746.8,3514.6)	937.69 (832.55,1056.19)	-0.69 (-0.75,-0.64)
Bulgaria	425336.7 (381297.8,475192.7)	3588.17 (3270.1,3955.55)	475735.3 (423695.8,529386.9)	3321.73 (2961.85,3660.73)	-0.36 (-0.43,-0.3)

Burkina Faso	88345.7 (79612.5,97919.2)	2121.96 (1911.35,2366.08)	201284.1 (178649,225058.2)	2238.55 (1979.61,2508.54)	0.14 (0.09,0.2)
Burundi	46216.1 (41532.4,51614.2)	2060.61 (1850.92,2311.74)	99685.2 (87916.1,112713)	2091.39 (1846.86,2372.87)	-0.03 (-0.04,-0.01)
Cabo Verde	5737.3 (5194.2,6383.8)	2475.12 (2250.92,2739.07)	12221.5 (10923.6,13712)	2813.85 (2504.58,3182.1)	0.46 (0.44,0.48)
Cambodia	68920.5 (62310.7,76313)	1709.59 (1550.94,1891.69)	207123.9 (185053.3,231187.3)	1828.62 (1645.61,2037.78)	0.3 (0.25,0.35)
Cameroon	84284.3 (76438.5,94262.1)	2015.29 (1825.76,2246.74)	274050.5 (243974.9,305526.3)	2276.99 (2033.42,2565.31)	0.39 (0.3,0.48)
Canada	742086.7 (684174,806721.4)	2267.31 (2093.18,2463.98)	1105820.2 (1000313.2,1252450.9)	1544.09 (1400.94,1734.92)	-1.48 (-1.62,-1.34)
Central African Republic	22225.4 (20129.6,24702.5)	2137.61 (1939.61,2387.23)	44368.6 (39170.5,50175.6)	2163.54 (1902.93,2463.37)	-0.04 (-0.06,-0.02)
Chad	65773.5 (59322.2,72906.3)	2383.07 (2145.92,2657.26)	146135 (129925.2,164465)	2623.66 (2319.45,2950.56)	0.31 (0.29,0.34)
Chile	131339.1 (118062.1,146229.9)	1331.44 (1200.02,1483.97)	327825.5 (293621.6,369291.9)	1273.73 (1143.98,1434.25)	-0.36 (-0.42,-0.29)
China	19505463.1 (16754811.3,22537174)	2526.44 (2189.97,2914.97)	63331311.5 (53812323.8,76196537)	3042.35 (2601.68,3629.87)	0.64 (0.56,0.72)
Colombia	441695.7 (406300.7,479651.3)	2581.42 (2372.54,2814.64)	1278631.1 (1144994.4,1415360.4)	2298.99 (2053.29,2544.74)	-0.45 (-0.5,-0.4)
Comoros	4423.6 (3980.1,4904.5)	2359.36 (2110.19,2612.56)	11675.7 (10297.7,13169.1)	2443.24 (2160.72,2775.85)	0.07 (0.04,0.1)
Congo	23643.5 (21465.2,26143.3)	2402.34 (2180.15,2666.71)	63683.4 (56987.7,71966)	2522.81 (2243.21,2861.25)	0.15 (0.12,0.18)
Cook Islands	327 (297.8,357.6)	2836.31 (2586.73,3113.7)	814.2 (735.6,907.5)	3134.55 (2841.95,3485.26)	0.34 (0.33,0.35)
Costa Rica	51973.3 (47413.2,56699)	2997.13 (2729.9,3272.82)	164735.7 (147434.8,183640.5)	2986.49 (2672.05,3332.15)	-0.07 (-0.09,-0.05)
Croatia	180980.5 (159422,205430.5)	3077.38 (2728.4,3458.02)	244160.4 (213311.1,280767.2)	2776.1 (2434.27,3154.31)	0.16 (0.14,0.17)
Cuba	335232 (306288.8,367149.9)	3283.99 (2999.74,3592.12)	579952.2 (519560.3,650616.8)	2976.59 (2669.05,3331.59)	-0.44 (-0.59,-0.3)
Cyprus	11053.2 (9515.9,12796.7)	1318.47 (1149.74,1507.17)	25051 (21567.2,29007.9)	1198.39 (1033.8,1385.58)	-0.43 (-0.58,-0.28)
Czechia	551153.9 (499492.6,609597.6)	4018.12 (3655.14,4426.14)	715423.1 (637291.6,796916.3)	3350.08 (2991.38,3711.04)	-0.77 (-1.01,-0.53)
Cmte d'Ivoire	94559.9 (86488,104737)	2569.02 (2332.69,2842.83)	292442.3 (261708.1,324032.7)	2737.65 (2446.21,3057.16)	-0.91 (-1.07,-0.75)
Democratic People's Republic of Korea	374058.9 (342296.8,407539.8)	2547.13 (2338.6,2779.13)	843789.2 (762378.7,940855.5)	2631.7 (2381.97,2915.77)	0.13 (0.11,0.14)
Democratic Republic of the Congo	324323.5 (294598.8,358248.9)	2239.98 (2028.72,2461.15)	722322 (645737.8,810156.4)	2055.21 (1840.49,2308.83)	-0.36 (-0.4,-0.32)
Denmark	96282.2 (82514.3,113875.8)	1180.06 (1016.75,1388.88)	125387.1 (108871.4,143684.5)	1085.82 (948.09,1240.27)	-0.4 (-0.49,-0.31)
Djibouti	3063.1 (2763.8,3420.9)	2398.48 (2159.03,2663)	16435.9 (14411.4,18516.3)	2668.37 (2349.24,3018.09)	0.36 (0.34,0.39)
Dominica	1749.5 (1593.8,1922.4)	2964.42 (2696.47,3247.61)	2668.5 (2381.9,2998.4)	3198.26 (2863.71,3587.49)	0.29 (0.25,0.32)
Dominican Republic	103796.5 (94186.9,113587.2)	2847.61 (2583.05,3119.36)	322839.6 (291882.2,358235.4)	3246.16 (2931.53,3606.37)	0.49 (0.45,0.53)
Ecuador	110734.5 (100903.8,121702.5)	2122.47 (1927.8,2338.33)	378091 (335337.1,421343.8)	2312.92 (2056.89,2582.21)	0.29 (0.28,0.31)
Egypt	1599278.4 (1500684.3,1715759.7)	6393.32 (6001.59,6810.01)	4274327.3 (3925680.6,4666756.5)	7100.29 (6580.94,7664.24)	0.45 (0.42,0.48)
El Salvador	64155.3 (58301.7,69875.5)	2187.18 (1987.12,2386.97)	148777.2 (133809.3,165515)	2380.22 (2132.17,2657.84)	0.28 (0.26,0.3)

Equatorial Guinea	4067.2 (3681,4492.3)	2272.52 (2030.49,2526.49)	12604.6 (11280.5,14116.6)	2663.51 (2380.36,3001.56)	0.67 (0.6,0.74)
Eritrea	20299.4 (18224.3,22725.3)	1959.61 (1756.87,2199.9)	54214 (47303.4,61164.6)	2044.55 (1794.84,2337.1)	0.09 (0.07,0.11)
Estonia	74835.4 (69191.6,81140.9)	3699.75 (3431.94,4007.83)	107710.5 (97610.2,120216.5)	3966.29 (3592.13,4385.34)	0.28 (0.17,0.39)
Eswatini	6231.7 (5628.5,6888.3)	2307 (2086.45,2566.55)	13397.9 (11788.5,15034.1)	2532.5 (2240.98,2848.72)	0.3 (0.25,0.36)
Ethiopia	373296.1 (313518.9,436638.1)	1955.86 (1671.06,2280.23)	854110.7 (718570.1,1007279.1)	1991.85 (1665.71,2376.33)	-0.01 (-0.07,0.05)
Fiji	10527.6 (9675,11565.8)	3340.46 (3060.94,3648.55)	23882.4 (21358.3,26618.9)	3404.98 (3076.64,3782.55)	0.13 (0.11,0.15)
Finland	175892.2 (155602.2,198577.3)	2484.52 (2202.64,2793.08)	198700.5 (173070.6,226417.9)	1618.74 (1418.61,1836.71)	-1.49 (-1.81,-1.16)
France	1614378.5 (1467211.5,1788197.6)	1959.02 (1790.62,2161.32)	2180522.6 (1942324.6,2473239.4)	1595.12 (1425.27,1801.5)	-0.78 (-0.88,-0.68)
Gabon	12655 (11579.7,14015.7)	2325.08 (2119.95,2566.42)	24454.7 (21873.9,27475.7)	2482.72 (2217.3,2808.99)	0.23 (0.22,0.24)
Gambia	8717.6 (7884.9,9654.5)	2608.49 (2342.55,2892.54)	26453.1 (23627.5,29701.1)	2794.08 (2490.2,3145.69)	0.17 (0.15,0.19)
Georgia	268775 (248174.5,291037.3)	4413.48 (4088.32,4764.84)	250566.2 (225347.4,277793.8)	4117.67 (3704.65,4551.63)	-0.35 (-0.39,-0.31)
Germany	3101107.6 (2833708.9,3409588.4)	2442.44 (2243.92,2676.81)	3151797.8 (2791317,3549641.4)	1683.17 (1496,1900.56)	-1.28 (-1.33,-1.22)
Ghana	149866.6 (136985.8,165101.9)	2562.8 (2329.57,2832.12)	434758.9 (390944.8,485634)	2706.34 (2416.5,3020.02)	0.12 (0.08,0.16)
Greece	228761.8 (200117.3,259631.2)	1490.56 (1314.08,1687.79)	261596.3 (229225.5,298980)	1157.57 (1019.15,1318.79)	-1.02 (-1.1,-0.94)
Greenland	550.8 (503.3,605.3)	1697.12 (1541.98,1868.67)	1116 (986.8,1271.5)	1650.3 (1474.68,1871.57)	-0.15 (-0.21,-0.1)
Grenada	2165.5 (1973.8,2390.3)	2996.78 (2729.07,3310.72)	3726 (3292.4,4180.9)	3305.1 (2940.5,3698.39)	0.34 (0.3,0.38)
Guam	1756.7 (1595.6,1929.6)	2646.75 (2420.35,2889.06)	6697 (6038.9,7465.9)	3116.71 (2804.27,3464.74)	0.58 (0.54,0.62)
Guatemala	65136.5 (59295.7,71743)	1965.04 (1792.32,2169.71)	233333.7 (208057.6,260276.1)	2145.5 (1924.08,2397.53)	0.34 (0.32,0.37)
Guinea	69995.7 (62905.2,77666.3)	2172.59 (1955.69,2416.63)	132930.4 (117976.7,149010.1)	2442.44 (2155.68,2746.16)	0.41 (0.4,0.43)
Guinea-Bissau	8613.7 (7790.4,9603.6)	2330.72 (2104.82,2591.09)	17192.1 (15364,19332.2)	2525.64 (2246.27,2852.32)	0.26 (0.25,0.27)
Guyana	11238.1 (10223.6,12360.6)	3022.13 (2745.16,3343.32)	19888.3 (17730.4,22303.3)	3163.78 (2832.6,3534.25)	0.11 (0.09,0.12)
Haiti	92721.6 (84750.6,101921.5)	2992.35 (2739.3,3273.36)	217236.9 (193680,243815.2)	3053.58 (2724.06,3421.2)	0.04 (0.01,0.06)
Honduras	50065.7 (45620.4,54776.8)	2514.6 (2294.7,2739.41)	167598.4 (150362.3,184997.1)	2705.22 (2431.71,2980.8)	0.25 (0.24,0.27)
Hungary	680132.1 (612240,756085.5)	4637.37 (4205.68,5116.24)	687289.2 (620332.1,765514.9)	3477.46 (3136.1,3845.7)	-1.04 (-1.11,-0.98)
Iceland	5607.8 (5062.8,6280.3)	1985.05 (1798.7,2216.62)	8906.9 (7863.6,10133.3)	1563.07 (1378.97,1767.67)	-1.15 (-1.39,-0.91)
India	17867820.1 (15107259.6,20818190.2)	4110.85 (3518.51,4788.48)	51933273.3 (43141707.5,62567272.6)	4458.18 (3744.48,5412.76)	0.27 (0.25,0.28)
Indonesia	1842025.6 (1574670.9,2126112.8)	2085.58 (1796.26,2404.55)	5158083 (4325307.1,6144844)	2349.89 (1998.83,2782.38)	0.5 (0.46,0.53)
Iran (Islamic Republic of)	1577754.2 (1359365.5,1830675.5)	6496.75 (5687.54,7443.82)	4787279.5 (4042186.8,5701615.2)	6327.23 (5393.23,7501.96)	-0.18 (-0.27,-0.1)
Iraq	528149.5 (498286.1,563416.6)	6903.89 (6511.89,7374.8)	1657453.5 (1525969.5,1824406.6)	7185.54 (6654.59,7885.02)	0.12 (0.1,0.13)
Ireland	81769.5 (72606.4,92172)	1970.07 (1761.93,2217.18)	113060.7 (98909.6,128752.3)	1436.8 (1255.1,1625.39)	-1.42 (-1.57,-1.27)
Israel	91813.8 (81253,104028.2)	1871.36 (1664.57,2112.95)	174884.5 (153117.2,199648.2)	1435.4 (1255.57,1633.61)	-1.06 (-1.13,-0.98)

Italy	1682045.6 (1429035.5,1944088.9)	1887.1 (1615.66,2179.93)	2109380.7 (1762593,2505110.6)	1544.57 (1296.3,1834.07)	-0.88 (-0.96,-0.81)
Jamaica	53270.8 (48449.2,59023.2)	2984.95 (2717.66,3301.74)	102513.4 (91701.2,114392.6)	3285.34 (2942.44,3675.94)	0.33 (0.29,0.38)
Japan	1590718 (1362924.6,1845351.1)	943.84 (811.03,1090.14)	2918517.1 (2466761.3,3474093.5)	802.34 (686.82,938.16)	-0.77 (-0.87,-0.67)
Jordan	83695.5 (77759.3,90259.1)	6662.44 (6198.39,7171.4)	502059.1 (455336.4,559952.6)	6816.99 (6204.99,7546.39)	-0.02 (-0.04,0.01)
Kazakhstan	519123.7 (482223.5,566246.2)	4331.53 (4033.68,4698.23)	718905.7 (643620.7,798577.3)	4230.88 (3828.87,4676.98)	-0.21 (-0.29,-0.13)
Kenya	199518.4 (170108,230849.6)	2506.57 (2132.88,2906.08)	580208.5 (487530.9,682696.3)	2590.54 (2181.28,3081.36)	0.05 (0,0.1)
Kiribati	981.1 (888.7,1079.3)	3021.58 (2749.25,3309.34)	1910.7 (1696.7,2153.1)	3000.51 (2696.55,3362.27)	-0.04 (-0.05,-0.02)
Kuwait	45131.6 (42134,48584.5)	7737.2 (7255.55,8322.85)	229757.9 (207386.8,253908.4)	7806.45 (7141.59,8556.31)	-0.03 (-0.09,0.02)
Kyrgyzstan	105698.9 (97344.1,115695.6)	3706.04 (3415.29,4033.23)	154882.3 (139780.6,171205.7)	3492.31 (3183.66,3858.39)	-0.21 (-0.24,-0.18)
Lao People's Democratic Republic	36248.1 (32901.8,40174.9)	1963.28 (1785.51,2154.98)	81893.3 (73304,91918)	1968.29 (1773.75,2200.46)	0.05 (-0.02,0.12)
Latvia	118663.3 (108065.6,130799.5)	3328.26 (3037.44,3662.17)	125178.7 (111383.1,141449.7)	3092.91 (2750.95,3480.56)	-0.56 (-0.7,-0.41)
Lebanon	132032.8 (123312.6,141854.7)	6340.74 (5935.01,6811.82)	410574.3 (377768.2,447702.1)	6814.65 (6242.72,7463.04)	0.26 (0.24,0.28)
Lesotho	14975.9 (13536.7,16774.8)	1820.57 (1641.24,2037.28)	21579.2 (19103.7,24142.6)	2086.28 (1849.58,2341.28)	0.49 (0.47,0.51)
Liberia	27573.5 (24822.3,30715.4)	2460.06 (2213.17,2743.3)	54411.5 (48516.3,60525.1)	2643.86 (2332.08,2977.66)	0.28 (0.25,0.31)
Libya	114281.5 (106828.2,123220.3)	6330.11 (5914.95,6817.36)	335569.1 (303899.1,370593.3)	6634.68 (6059.17,7302.98)	0.1 (0.06,0.13)
Lithuania	147394.9 (135415.4,160688.2)	3279.69 (3015.59,3573.58)	179816.4 (160798.9,201354.4)	3040.21 (2715.44,3402.97)	-0.28 (-0.44,-0.12)
Luxembourg	6817.4 (5922.6,7814.8)	1246.83 (1091.2,1428.51)	12959.6 (11274.9,14780.9)	1239.81 (1079.44,1411.72)	-0.31 (-0.47,-0.16)
Madagascar	108993.5 (98273,121021.8)	2256.55 (2027.95,2512.59)	257071.4 (226515.8,289218.6)	2382.91 (2115.55,2683.59)	0.16 (0.16,0.17)
Malawi	83758 (75343.5,94210.1)	2269.81 (2043.15,2551.87)	176505.6 (154869.9,199804.8)	2429.14 (2141.61,2767.89)	0.1 (0.03,0.17)
Malaysia	222541.4 (205104.1,242662)	2583.19 (2381.38,2807.03)	770701 (690322.8,851718.7)	2857.76 (2581.66,3143.7)	0.52 (0.46,0.57)
Maldives	1675.2 (1505.6,1869.1)	2173.4 (1956.48,2408.26)	6642.5 (5952.2,7424.7)	2050.89 (1821.79,2311.71)	-0.26 (-0.31,-0.21)
Mali	79565.4 (71844,87479.2)	2115.46 (1902.26,2347.57)	194854.8 (173414.6,219981.7)	2298.64 (2040.33,2607.61)	0.23 (0.21,0.26)
Malta	6216.8 (5403.8,7120.6)	1452.89 (1266.89,1662.64)	12175.6 (10516,14020.2)	1272.1 (1103.48,1448.13)	-0.51 (-0.69,-0.32)
Marshall Islands	387.6 (353.6,423.6)	2647.59 (2423.92,2897.31)	825.8 (732.1,921.3)	2756.98 (2461.12,3083.53)	0.12 (0.1,0.15)
Mauritania	25549.6 (23117.3,28415.6)	2635.95 (2380.15,2920.89)	59929.4 (53383.2,67251.2)	2847.47 (2543.25,3209.18)	0.22 (0.2,0.25)
Mauritius	15390.5 (13911.2,16968.8)	2301.37 (2085.17,2516.36)	38277.4 (34015.6,42990)	2124.59 (1897.29,2377)	-0.52 (-0.63,-0.42)
Mexico	1163203.4 (1006940.4,1337844.2)	2798.08 (2417.87,3240.52)	3513288.5 (2974794.3,4204715.9)	2797.04 (2384.64,3344.04)	-0.08 (-0.12,-0.03)
Micronesia (Federated States of)	1262.7 (1155.1,1389.7)	2812.8 (2568.49,3104.65)	1862.3 (1653,2087.8)	2850.1 (2550.23,3189.9)	0.07 (0.05,0.08)
Monaco	1083.1 (953.2,1225.2)	1560.24 (1381.88,1741.72)	1389.2 (1218.6,1592.7)	1454.73 (1279.26,1647.73)	-0.41 (-0.46,-0.35)
Mongolia	42884.7 (39280.5,46593)	4339.07 (3986.59,4702.99)	88775.3 (79717.2,98381.7)	4213.77 (3806.63,4658.65)	-0.2 (-0.23,-0.16)
Montenegro	22081.4 (19652,24742.9)	3600.63 (3206.87,4029.76)	36358.1 (31785.2,41189.4)	3731.24 (3294.65,4185.02)	0.13 (0.09,0.17)

Morocco	919245.2 (861026.5,986117.7)	6675.83 (6259.03,7151.03)	2312028.4 (2120992.9,2510176.8)	6850.37 (6335.79,7416.41)	0.04 (0.01,0.06)
Mozambique	126468.8 (113045.5,142025.7)	2181.67 (1943.04,2459.24)	255360.8 (223020.5,290048.8)	2348.63 (2063.78,2682.72)	0.17 (0.15,0.19)
Myanmar	431182.8 (396369.5,473301.7)	2073.35 (1905.21,2261.66)	870840.7 (784390,966707.3)	1927.91 (1745.9,2132.74)	-0.18 (-0.22,-0.15)
Namibia	15273.6 (13810.5,16987.1)	2498.06 (2258.93,2769.46)	31890.3 (28453.9,35771.6)	2422.7 (2165.07,2723.96)	-0.12 (-0.13,-0.1)
Nauru	119.4 (107.7,131.2)	3104.92 (2835.83,3392.97)	158.6 (141,178.3)	3148.77 (2827.14,3541.63)	-0.01 (-0.05,0.03)
Nepal	338025.5 (311776.4,370033.8)	3831.52 (3509.77,4210.35)	851319.7 (773009.7,940613.5)	3740.04 (3407.51,4131.26)	-0.11 (-0.14,-0.09)
Netherlands	406787.1 (362874.8,455775.3)	2042.64 (1830.74,2287.97)	509888.8 (444675.7,586574.6)	1486.27 (1306.66,1698.66)	-1.42 (-1.54,-1.3)
New Zealand	136470.5 (116660,158268.7)	3468.44 (2964.19,4032.77)	208161.5 (171656.7,255762)	2442.82 (2022.81,2978.33)	-1.49 (-1.61,-1.37)
Nicaragua	37608.4 (34475.8,41081.2)	2502.14 (2278.41,2735.71)	119492.1 (106491.8,132984.3)	2470.7 (2208.78,2752.4)	-0.05 (-0.06,-0.04)
Niger	57152.7 (51776.8,63233.8)	2179.88 (1963.37,2428.28)	179763.5 (159522,201024)	2253.94 (2012.56,2533.09)	0.05 (0,0.1)
Nigeria	1044361.5 (889685.1,1214161)	2440.67 (2081.13,2837.45)	2424485 (2031768,2865818.3)	2745.67 (2322.04,3286.99)	0.47 (0.43,0.51)
Niue	66.2 (60.6,72.5)	2894 (2641.77,3164.13)	64.7 (58.1,72.5)	3033.36 (2724.94,3399.23)	0.15 (0.13,0.17)
North Macedonia	63767.8 (57185.6,71185.4)	3528.31 (3184.41,3935.69)	111163.1 (96847.1,126102.3)	3423.57 (3014.96,3835.59)	-0.13 (-0.15,-0.11)
Northern Mariana Islands	459.4 (417.8,502.4)	3124.5 (2851.09,3416.31)	1522.3 (1352.3,1713.9)	3247.51 (2924.13,3635.82)	0.12 (0.08,0.16)
Norway	129859.8 (110186.7,151280.5)	1913.77 (1633.69,2221.35)	144840.5 (121466.7,172633.8)	1478.62 (1245.13,1762.63)	-1.04 (-1.16,-0.92)
Oman	40027.4 (37410,42915.7)	6313.54 (5888.17,6754.71)	138191.9 (124247.6,152419.6)	6854.91 (6258.97,7535.67)	0.3 (0.23,0.38)
Pakistan	2703186.9 (2312179.2,3108150.4)	4961.15 (4266.28,5698.99)	6030542.6 (5025684.1,7155613.9)	5155.28 (4349.29,6133.65)	0.15 (0.12,0.17)
Palau	240.4 (219.8,266.4)	2685.13 (2460.08,2968.16)	622.6 (555.4,698.5)	3050.32 (2730.34,3393.14)	0.44 (0.4,0.48)
Palestine	51446.4 (47694.6,55765.9)	6152.97 (5712.68,6684.65)	155780.8 (141746.1,172360.4)	6302.59 (5748.27,6947.22)	0 (-0.03,0.02)
Panama	37117.6 (34233.6,40609.4)	2524.25 (2325.81,2765.29)	117244.8 (104971.2,130723.3)	2643.61 (2361.03,2949.29)	0.16 (0.15,0.16)
Papua New Guinea	40972.5 (37240.8,45002.3)	2642.11 (2415.95,2905.09)	123045.3 (109528.4,137226.6)	2752.72 (2465.15,3062.85)	0.14 (0.12,0.17)
Paraguay	43498.5 (38618.1,48850.2)	1967.16 (1740.14,2219.72)	118299.4 (103288.8,134948.1)	2027.55 (1770.76,2311.65)	0.03 (0.01,0.05)
Peru	238055.2 (216002.6,261605.5)	2018.38 (1829.6,2223.1)	750182.6 (667829.2,838820.1)	2233.02 (1984.49,2504.74)	0.4 (0.32,0.48)
Philippines	538580.1 (460442.6,620223.6)	1985.96 (1716.59,2291.51)	1451573.1 (1221224,1716354.3)	1881.91 (1604.13,2205.58)	-0.4 (-0.57,-0.23)
Poland	1571833.7 (1352968.3,1818736)	3643.61 (3151.67,4188.22)	2087659 (1762556.8,2467760.7)	2904.45 (2469.01,3423.45)	-0.89 (-0.99,-0.79)
Portugal	168490.3 (146685.7,192606)	1193.1 (1046.12,1357.93)	223738.3 (190384.4,261377.1)	947.1 (806.24,1099.61)	-1.27 (-1.41,-1.13)
Puerto Rico	121823.3 (111597.8,133243.6)	3364.11 (3086.05,3672.31)	244061.3 (218164.1,274660.1)	3456.2 (3089.08,3862.02)	0.05 (0.04,0.06)
Qatar	8560.5 (7900.7,9304.2)	7379.77 (6864.03,7904.69)	77595.2 (69086.5,87350.1)	7143.84 (6501.02,7864.41)	-0.17 (-0.21,-0.12)
Republic of Korea	304843.3 (279161.9,336172.4)	1118.13 (1028.29,1232.87)	788589.6 (697856.9,892037.5)	841.83 (748.12,948.82)	-1.12 (-1.24,-0.99)
Republic of Moldova	141590.3 (130615.4,154770.5)	3470.79 (3219.69,3766.65)	202545.1 (183718.1,224580.2)	3389.82 (3077.65,3744.47)	-0.04 (-0.06,-0.03)
Romania	1002782.7 (903616.8,1121241.4)	3719.68 (3376.99,4120.96)	1224024.8 (1090389.1,1370116.2)	3277.42 (2918.82,3661.68)	-0.9 (-1.07,-0.73)
Russian Federation	8242544.1 (7192086.3,9399669.6)	4758.48 (4182.91,5385.01)	12624157.8	5279.14 (4568.46,6189.43)	0.23 (0.12,0.34)

			(10898100.3,14905229.2)		
Rwanda	52157.9 (46811.5,58841.2)	1945.25 (1746.36,2190.66)	122460.3 (108778.4,138326.8)	1986.34 (1756.9,2254.25)	0.01 (-0.04,0.06)
Saint Kitts and Nevis	1165.5 (1057.7,1283.6)	3113.23 (2842.72,3416.34)	2226.8 (1977.3,2495.8)	3262.02 (2934.98,3622.48)	0.06 (0.04,0.09)
Saint Lucia	2871.5 (2604,3167.7)	3302.35 (3004.91,3630.34)	8074.6 (7197,9039.3)	3359.6 (3004.06,3761.81)	0.1 (0.07,0.12)
Saint Vincent and the Grenadines	2077.8 (1883.5,2280.5)	2930.96 (2663.07,3211.63)	4450.7 (3967.8,4995.9)	3120.2 (2793.14,3482.11)	0.22 (0.21,0.23)
Samoa	2408.8 (2194.7,2635.2)	3081.36 (2816.25,3361.38)	4302.6 (3844.4,4815.2)	3189.58 (2865.16,3564.88)	0.11 (0.09,0.12)
San Marino	539.5 (477,602)	1535.82 (1364.66,1713.84)	1031.2 (901.8,1173.1)	1434.39 (1259.89,1625)	-0.37 (-0.42,-0.32)
Sao Tome and Principe	1547.4 (1394.2,1711.8)	2443.14 (2203.89,2697.37)	2919.6 (2601.8,3251.5)	2709.64 (2411.03,3048.19)	0.34 (0.32,0.37)
Saudi Arabia	344963 (321903.8,370003.1)	6267.77 (5855.7,6718.94)	1407289.4 (1272028.5,1545986.3)	7341.64 (6747.53,7966.8)	0.59 (0.54,0.64)
Senegal	85759.3 (78375.8,95089.8)	2764.45 (2515.91,3070.17)	213638.1 (190107.6,240266.3)	2828.37 (2519.16,3205.75)	0.03 (0,0.07)
Serbia	447923.7 (405627.9,501565.9)	4116.43 (3746.31,4558.29)	648643.2 (574028.8,720483.8)	3958.92 (3508.6,4363.42)	-0.43 (-0.53,-0.33)
Seychelles	1098.7 (990.8,1216.6)	1942 (1752.9,2146.05)	2297.2 (2023.4,2593.2)	2073.95 (1842.02,2344.23)	0.15 (0.13,0.17)
Sierra Leone	53275.7 (47972.6,59098)	2666.05 (2396.05,2968.98)	96035.9 (85774.6,107421.7)	2614.24 (2340.19,2943.79)	-0.12 (-0.17,-0.08)
Singapore	30835.1 (28059.6,34074.9)	1452.86 (1322.47,1604.58)	115852.3 (109921.4,122776)	1350.84 (1280.81,1431.76)	-0.29 (-0.38,-0.2)
Slovakia	206127.7 (185690.6,228939.9)	3479.83 (3145.42,3851.77)	284252 (249667.5,318799.3)	2987.22 (2644.16,3325.7)	-0.62 (-0.83,-0.41)
Slovenia	86355.9 (76770.9,96340.3)	3528.67 (3143.93,3932.41)	143108.4 (125880.4,161470.8)	3247.42 (2846.45,3641.34)	-0.58 (-0.87,-0.29)
Solomon Islands	3410.5 (3098.4,3769.2)	2904.61 (2656.18,3194.71)	9282.3 (8315.3,10400.7)	3043.63 (2737.79,3403.5)	0.21 (0.19,0.24)
Somalia	46413.3 (41560.2,52186.5)	2040.44 (1818.26,2319.14)	125219 (109604.3,142936.4)	2063.39 (1815.08,2356.54)	-0.02 (-0.04,0.01)
South Africa	599803.5 (513181.5,695631.3)	3009.73 (2580.12,3500.9)	1283629.9 (1076863.5,1532288)	2839.86 (2396.41,3389.41)	-0.31 (-0.38,-0.24)
South Sudan	55209.2 (49548.5,61830.3)	2228.94 (1995.96,2487.14)	85371.9 (75528,96302.5)	2300.26 (2025.93,2607.96)	0.12 (0.07,0.17)
Spain	803351.2 (715672,904175.3)	1467.23 (1317.56,1640.7)	1171513.3 (1033506.4,1315568.3)	1266.34 (1127.84,1404.8)	-0.47 (-0.53,-0.41)
Sri Lanka	221956.3 (203123.8,244085.7)	2252.77 (2063.82,2471.2)	582057.5 (522079.9,653340.6)	2204.12 (1990.83,2458.31)	-0.07 (-0.14,0)
Sudan	592501.7 (555033.1,638936.1)	6646.1 (6224.8,7136.07)	1286113.3 (1180802.6,1402644.5)	6817.59 (6290.96,7433.74)	0.05 (0.03,0.06)
Suriname	8306.8 (7533.2,9150.3)	3313.21 (3002.59,3649.11)	21858.3 (19576.4,24527.4)	3471.68 (3126.82,3886.67)	0.15 (0.13,0.17)
Sweden	236532.6 (200514,278874.5)	1553.7 (1328.44,1803.42)	312501.7 (264288.5,367431.7)	1459.82 (1245.57,1714.73)	-0.44 (-0.51,-0.36)
Switzerland	163957.3 (145871.2,183443.2)	1589.88 (1425.51,1768.45)	219531 (192091.2,251211.8)	1249.13 (1092.19,1416.02)	-0.81 (-0.96,-0.66)
Syrian Arab Republic	332372.6 (311651,355262.3)	6706.56 (6309.23,7146.71)	888454.1 (815278.7,977047.5)	6859.54 (6321.62,7467.57)	0.07 (0.05,0.09)
Taiwan (Province of China)	427293.6 (392382.3,466788.2)	2893.76 (2670.94,3139.25)	1203637.9 (1091226.2,1345908.6)	2817.32 (2556.51,3144.34)	-0.18 (-0.2,-0.16)
Tajikistan	100857.5 (92805.6,109489.8)	3865.59 (3560.11,4190.3)	214098.3 (192327.8,238497.9)	4105.81 (3730.25,4525.27)	0.26 (0.21,0.31)
Thailand	591461.7 (538114.2,650385.4)	1829.68 (1672.73,2010.38)	1888691.3 (1687558.5,2111282.5)	1732.21 (1551.16,1933.28)	-0.29 (-0.33,-0.25)

Timor-Leste	4258.1 (3830.1,4705)	1749.59 (1572.27,1925.51)	15244.3 (13534.2,17313.7)	1887.26 (1692.82,2134.91)	0.31 (0.28,0.34)
Togo	28539.1 (25964.8,31584.1)	2449.41 (2210.84,2717.61)	91813.7 (81483.4,102920.2)	2502.85 (2229.98,2825.33)	0.03 (0,0.05)
Tokelau	32.2 (29.4,35.5)	2458.56 (2256.19,2697.13)	42.7 (38.3,47.9)	2845.41 (2560.17,3184.56)	0.49 (0.47,0.51)
Tonga	1545.5 (1398.8,1691.7)	3009.77 (2739.6,3287.84)	2471.9 (2225.3,2751.3)	3153.6 (2832.02,3513.92)	0.14 (0.13,0.15)
Trinidad and Tobago	31020.1 (28473.5,33932.4)	3725.05 (3410.31,4075.93)	75841.3 (67214.1,85077.6)	3917.34 (3495.62,4379.84)	0.11 (0.1,0.13)
Tunisia	284854.1 (265387.8,307218.6)	5893.86 (5490.26,6342.63)	793610.5 (725567.9,870099.7)	6011.91 (5516.8,6558.82)	-0.01 (-0.03,0.02)
Turkmenistan	72880.8 (67136.8,78899.1)	4156.32 (3830.23,4481.95)	166579.3 (149588.4,184701.9)	4460.74 (4029.37,4943.29)	-0.55 (-0.6,-0.51)
Tuvalu	151.2 (137.1,167.2)	2520.99 (2305.35,2772.65)	275.8 (245.5,309.9)	2817.02 (2535.6,3142.5)	0.21 (0.19,0.22)
Timor-Leste	2046513.4 (1921459,2196549.9)	6090.46 (5727.54,6507.62)	5015695.3 (4562044.6,5507294.3)	5365.86 (4895.52,5874.65)	0.34 (0.31,0.36)
Uganda	128221.3 (114265.8,143693)	2053.6 (1835.53,2294.04)	294517.9 (259198.5,330605.4)	2050.67 (1811.64,2314.69)	-0.11 (-0.16,-0.06)
Ukraine	3017746.4 (2606459.2,3473242)	4321.81 (3757.34,4939.1)	3551142.1 (3025390.5,4189028.3)	4518.85 (3867.46,5313.07)	0.12 (0.08,0.16)
United Arab Emirates	32893.1 (30511.4,35457.1)	7276.18 (6814.51,7794.54)	384627.5 (344256.3,428314.1)	7608.89 (6995.21,8286.01)	0.12 (0.07,0.16)
United Kingdom	1755046.2 (1523281.5,2014513.5)	1953.27 (1692.63,2228.79)	1702773.5 (1453038,2001056.5)	1363.38 (1162.34,1599.93)	-1.45 (-1.62,-1.27)
United Republic of Tanzania	206089.4 (185064.8,227502.5)	1958.48 (1758.84,2172.54)	590966.5 (524387,664125)	2363.96 (2095.97,2669.73)	0.67 (0.61,0.73)
United States of America	9341215.8 (7880209.7,10980078.8)	2912.81 (2469.35,3410.96)	8655573.8 (7305087.1,10307536.7)	1488.78 (1263.92,1756.36)	-2.57 (-2.75,-2.38)
United States Virgin Islands	2501.3 (2267.7,2752)	3028.06 (2748.86,3323.02)	6155.7 (5497.1,6915.6)	3369.91 (3024.74,3757.1)	0.4 (0.37,0.43)
Uruguay	62429.1 (56389.3,69662.6)	1585.99 (1434.53,1761.53)	81365.3 (72349.7,92221)	1485.26 (1318.8,1682.6)	-0.39 (-0.47,-0.31)
Uzbekistan	425529.8 (392674,464152.5)	3845.5 (3546.49,4202.85)	1173369.3 (1079579,1274519.3)	5015.59 (4660.91,5391.59)	1 (0.89,1.11)
Vanuatu	1753.1 (1586,1930.2)	3205.82 (2924.09,3518.77)	5278.6 (4678.1,5915.9)	3392.41 (3036.9,3799.35)	0.16 (0.15,0.18)
Venezuela (Bolivarian Republic of)	275129.7 (253212.8,298752.2)	2909.28 (2669.78,3167.27)	807079.5 (724324.2,896446.1)	2688.96 (2428.9,2974.7)	-0.32 (-0.34,-0.29)
Viet Nam	677193 (618262.2,746309)	1758.77 (1608.38,1930.92)	1842804.8 (1661104.7,2043083.8)	1961.27 (1779.89,2182.1)	0.49 (0.43,0.54)
Yemen	289928.4 (270893.2,313585.4)	6270.07 (5871.81,6728.85)	835602.4 (768056,915423.8)	6182.82 (5696.13,6755.92)	-0.06 (-0.07,-0.05)
Zambia	56328 (50633.8,62766.1)	2096.22 (1881.02,2341.84)	140718.2 (123843.3,158315.3)	2109.97 (1848.31,2384.45)	-0.01 (-0.05,0.03)
Zimbabwe	94197 (85064.1,105134.3)	2415 (2179.73,2684.75)	168087.5 (150099.3,188368.3)	2555.2 (2267.36,2867.52)	0.15 (0.08,0.21)

Table S2 The incidence of ischemic heart disease cases and age-standardized rate at national level in 1990 and 2021, with EAPC (1990-2021).

Location	1990		2021		EAPC (95% CI)
	Number	ASR	Number	ASR	
Afghanistan	76474.7 (66089.8,89577.3)	1155.81 (1011.11,1350.11)	95471.5 (82028.6,111417.1)	964.61 (833.22,1125.98)	-0.78 (-0.85,-0.7)
Albania	6732.2 (5336.9,8367.8)	333.02 (266.81,412.14)	13641.3 (11112.4,16922.6)	322.15 (263.65,390.87)	-0.04 (-0.12,0.04)
Algeria	124514.3 (106099.3,149671.5)	1106.43 (963.37,1304.27)	300542.4 (259633.4,355483.7)	861.13 (753.02,1009.41)	-1.14 (-1.25,-1.02)
American Samoa	74.9 (58.4,94.2)	377.61 (292.87,473.88)	177.4 (138.6,227.6)	403.26 (312.75,519.22)	0.2 (0.18,0.21)
Andorra	105.3 (81.1,134.5)	187.32 (144.85,239.13)	246.7 (194.4,314.1)	158.24 (125.36,201.39)	-0.73 (-0.81,-0.65)
Angola	13802.5 (10938.2,17213.5)	385.89 (307.89,473.37)	39159.7 (31265.7,48236.1)	346.95 (282.03,418.75)	-0.5 (-0.55,-0.45)
Antigua and Barbuda	203.3 (158.9,253.4)	375.72 (294.5,465.25)	397.7 (312.7,504.4)	382.38 (302.88,481.55)	0.13 (0.1,0.16)
Argentina	110811.8 (97236.5,128208.9)	357.69 (315.71,410.38)	135407.3 (115179.3,160520.1)	243.87 (207.22,291.57)	-1.61 (-1.88,-1.34)
Armenia	16243.1 (13489.7,19767.1)	649.55 (547.43,772.11)	25550.7 (21096.4,30959.7)	591 (490.42,710.55)	-0.49 (-0.56,-0.42)
Australia	65818.2 (58075.7,74535)	344.16 (305.95,389.1)	94563.2 (77489.1,114341)	213.72 (176.25,262.25)	-1.43 (-1.8,-1.06)
Austria	27638.1 (24827.6,30736.3)	232.32 (208.39,258.28)	35688.7 (31672.1,40199.5)	192.62 (170.88,218.82)	-0.74 (-0.81,-0.67)
Azerbaijan	30187.7 (26013.3,35576.5)	654.64 (567.56,759.47)	70362 (61773.8,80778.5)	806.26 (725.6,906.9)	1.02 (0.88,1.17)
Bahamas	597 (484,737.9)	388.23 (308.66,486.8)	1603.5 (1266.9,2021.9)	401.65 (318.36,502.75)	0.12 (0.1,0.15)
Bahrain	1905.6 (1483.6,2384)	1090.26 (849.57,1357.19)	10199.2 (7628.2,13353.7)	1023.47 (792.34,1294.14)	-0.47 (-0.55,-0.4)
Bangladesh	237583.3 (203451.9,279662.5)	511.17 (439.9,602.08)	644472.5 (556233.8,762315.6)	469.75 (405.86,551.3)	-0.15 (-0.21,-0.08)
Barbados	1057.6 (831,1334.7)	367.46 (290.57,464.83)	1953.6 (1527.4,2505.2)	390.55 (308.04,491.17)	0.21 (0.17,0.25)
Belarus	74247.6 (66434.5,84869.9)	597.44 (539.33,674.7)	97360.9 (88334.1,109277.4)	606.69 (552.13,679.03)	0.03 (-0.16,0.22)
Belgium	48085.5 (42148.7,55167.9)	322.26 (280.82,369)	39610.3 (32608,47825.3)	170.49 (138.34,207.13)	-2.5 (-2.96,-2.03)
Belize	337.6 (268.2,419.8)	356.4 (281.48,445.42)	1136.1 (898.5,1419.6)	378.41 (294.1,474.76)	0.19 (0.16,0.22)
Benin	6974.2 (5482.6,8701.6)	365.07 (285.78,456.91)	18148.7 (14314.2,22617.8)	361.71 (286.18,449.82)	-0.12 (-0.16,-0.08)
Bermuda	227.2 (179.4,282.5)	371.42 (290.89,460.88)	496.8 (385.6,630.6)	373.73 (294.5,470.34)	0.05 (0.01,0.09)
Bhutan	1370.4 (1033.5,1761.6)	586.2 (449.14,741.76)	3525.3 (2728.5,4443.5)	587.35 (453.84,739.37)	-0.03 (-0.05,-0.01)
Bolivia (Plurinational State of)	7965.1 (6434.4,9793.2)	258.78 (209.36,316.38)	22212.7 (17983,27219.8)	251.69 (203.87,304.63)	-0.08 (-0.13,-0.03)
Bosnia and Herzegovina	14354.5 (11696.9,17612.6)	374.06 (310.6,447.28)	19807.7 (16045,24296.4)	324.88 (265.57,394.41)	-0.59 (-0.68,-0.51)
Botswana	1855.2 (1410.9,2344.7)	355.2 (277.36,449.39)	5429.8 (4218.2,6797.1)	379.05 (294.21,473.61)	0.13 (0.09,0.17)
Brazil	176403.8 (147670.1,209651.3)	197.12 (163.41,234.79)	420771.6 (341548.7,505774.2)	167.57 (136.27,200.4)	-0.37 (-0.45,-0.3)
Brunei Darussalam	130 (102.8,160.1)	130.11 (102.31,163.78)	373.5 (289.3,474.6)	115.47 (90.12,146.75)	-0.36 (-0.41,-0.32)
Bulgaria	59890 (52786.6,68201.3)	557.45 (501.97,620.73)	57467.7 (50592.4,65149.8)	413.13 (364.08,471.42)	-1.49 (-1.73,-1.24)

Burkina Faso	13572.8 (10721.4,16932.4)	339.73 (270.69,420.11)	29837.9 (24000.3,36755.4)	336.69 (273.26,411.12)	0 (-0.03,0.03)
Burundi	7104.9 (5610.2,8888.9)	326.35 (257.88,405.76)	14915.5 (11606.5,18899)	317.54 (247.49,398.05)	-0.24 (-0.28,-0.2)
Cabo Verde	852.5 (663.5,1105.8)	368.44 (284.68,474.63)	1727.6 (1352.5,2151.4)	391.91 (307.85,495.61)	0.18 (0.14,0.22)
Cambodia	8763.4 (6942,10880.6)	223.41 (178.72,274.13)	26152.8 (21309.7,31739.2)	237.77 (197.74,281.58)	0.37 (0.27,0.47)
Cameroon	13067.5 (10184.7,16377)	317.05 (250.94,393.67)	41018.8 (33568.1,50400.1)	340.42 (281.49,413.15)	0.12 (0.01,0.23)
Canada	128768.7 (113261.4,149684.4)	397.51 (349.1,463.63)	146396.5 (123174.7,174147.5)	204.24 (170.67,245.09)	-2.65 (-2.89,-2.41)
Central African Republic	3973.5 (3077.6,5067)	384.39 (301.59,483.48)	7969.6 (6152.3,10188.5)	387.14 (305,487.17)	-0.05 (-0.08,-0.02)
Chad	10036.2 (8002.6,12529.8)	374.33 (298.87,463.12)	20754.7 (16680.9,25572.9)	375.09 (298.44,461.03)	-0.06 (-0.1,-0.02)
Chile	18930.9 (15526.9,22862.2)	200.74 (165.52,239.26)	27657.8 (24809.9,32804.2)	108.52 (97.55,128.5)	-1.56 (-1.88,-1.24)
China	2301643.5 (1861968.5,2792193.4)	315.31 (255.53,382.49)	7304573.2 (5815313.2,8949994.7)	365.67 (293.32,440.07)	0.66 (0.51,0.82)
Colombia	58277.5 (49057.5,69016.3)	337.56 (286.54,395.42)	147725.7 (124311.5,176539.6)	261.03 (218.32,311.76)	-0.93 (-1.02,-0.84)
Comoros	601 (466.9,770.3)	330.62 (254.11,419.58)	1572.8 (1235.1,1991.9)	335.59 (264.21,426.51)	0.01 (0,0.03)
Congo	3796.5 (2968.4,4772.4)	388.91 (305.82,481.64)	10064.6 (7871.3,12733.8)	391.55 (307.1,482.59)	-0.08 (-0.1,-0.05)
Cook Islands	40.2 (31.5,50.9)	350.75 (275.57,446.85)	98 (75.7,125.6)	381.65 (297.16,486.76)	0.3 (0.28,0.32)
Costa Rica	6258.7 (5026.7,7616.8)	355.36 (284.05,433.15)	18170.4 (14453.8,22445.1)	325.92 (259.79,401.55)	-0.43 (-0.48,-0.38)
Croatia	23887.6 (20343.9,27774.2)	427.22 (371.35,489.84)	21712 (18855.5,25018.3)	245.54 (213.01,283.69)	-0.21 (-0.27,-0.15)
Cuba	44007 (37734.7,51094.6)	438.5 (376.31,508.38)	64140 (53167.6,76901.1)	330.27 (274.53,396.4)	-1.55 (-2.02,-1.08)
Cyprus	1185.5 (936.4,1494.8)	151.54 (122.11,186.51)	2357.4 (1857.3,2959.4)	119.58 (95.44,148.28)	-1.11 (-1.34,-0.88)
Czechia	73582.1 (66211.3,82611.9)	546.76 (493.68,612.39)	76588.7 (66263.5,89208)	369.33 (314.8,435.61)	-1.31 (-2.03,-0.59)
Cmte d'Ivoire	14671.1 (11874.8,18197.2)	400.6 (324,493.4)	42073.5 (33983.8,51739.3)	390.73 (316.46,467.78)	-1.74 (-2.04,-1.43)
Democratic People's Republic of Korea	47958.6 (38580.1,58380.2)	332.88 (269.67,396.6)	108777.8 (91292.4,130659)	346.24 (293.67,411.15)	0.15 (0.07,0.22)
Democratic Republic of the Congo	52971.1 (43743.8,64898)	369.84 (311.56,436.35)	118775.3 (98650.8,144429)	336.17 (285.3,397.27)	-0.43 (-0.47,-0.39)
Denmark	11784.4 (10305.5,13097.1)	141.76 (124.71,157.33)	15485.5 (12626.6,19089.6)	132.46 (107.93,164)	-0.73 (-1.03,-0.43)
Djibouti	429.8 (331.3,544.9)	336.09 (260.22,425.26)	2195.1 (1700,2792.5)	359.64 (278.64,456.56)	0.23 (0.22,0.24)
Dominica	217.8 (171.7,274.6)	376.42 (296.5,470.85)	313 (243.3,397.2)	384.71 (299.19,481.6)	0.14 (0.1,0.18)
Dominican Republic	12804.5 (10361,15532.7)	352.94 (284.85,424.88)	38373.7 (32237.3,45022.5)	383.62 (323.09,448.03)	0.55 (0.45,0.65)
Ecuador	13167 (10651.4,15908.3)	250.74 (202.85,303.44)	40340.4 (33020.8,48642.2)	250.34 (204.21,301.14)	-0.02 (-0.06,0.02)
Egypt	272703.8 (234606.9,315154)	1075.98 (954.18,1224.53)	653784.5 (560059.8,748573.8)	1063.03 (927.44,1200.31)	0.19 (0.09,0.28)
El Salvador	8583.8 (6946.8,10512)	287.37 (231.01,354.17)	18821.8 (15257.9,23011.4)	293.73 (237,360.88)	0.03 (0.01,0.05)
Equatorial Guinea	669.5 (519.7,840)	378.67 (297.97,475.63)	1899.7 (1492.1,2355.4)	381.85 (298.35,486.93)	0.01 (-0.04,0.06)

Eritrea	3196.4 (2455.9,4110.4)	317.81 (247.31,404)	8118.5 (6349.1,10199.7)	310.99 (243.09,391.38)	-0.14 (-0.17,-0.1)
Estonia	12260.2 (10831.3,13875.1)	624.08 (554.73,701)	15238.5 (12355.2,18761.5)	559.35 (451.61,686.34)	-0.64 (-0.83,-0.46)
Eswatini	939.1 (731.4,1179.3)	343.47 (266.88,435.34)	1938.5 (1510.6,2424.6)	365.62 (284.58,461.07)	0.22 (0.2,0.24)
Ethiopia	57152.3 (44738.9,71853.7)	315.17 (247.44,391.05)	122902.4 (97183.7,151819.8)	286.9 (225.65,356.63)	-0.52 (-0.61,-0.43)
Fiji	1384.5 (1082.1,1726)	439.93 (344.21,553.78)	3032.9 (2356.9,3830.5)	441.76 (346.45,549.38)	0.09 (0.03,0.15)
Finland	33400.3 (29890,37317.2)	484.16 (434.04,539.57)	29007.3 (24703,34154.6)	224.32 (187.44,269.62)	-2.39 (-3,-1.78)
France	233422.3 (209001.9,261151.1)	283.69 (256.56,314.88)	249807.8 (214444.4,294830.9)	176.27 (147.06,213.19)	-0.98 (-1.19,-0.78)
Gabon	1883.5 (1465.5,2414.2)	347.79 (271.21,445.28)	3676.4 (2816.3,4630.9)	366.64 (286,458.79)	0.15 (0.14,0.15)
Gambia	1325.2 (1034.9,1662.7)	405.36 (313.01,517.72)	3909.5 (3134.4,4832.9)	416.26 (325.28,522.86)	0.02 (-0.01,0.04)
Georgia	45929.1 (40557.5,52954.8)	780.37 (695.73,888.33)	33809.5 (28191.5,40642.8)	553.85 (458.86,662.12)	-1.49 (-1.6,-1.37)
Germany	551176.5 (505918.2,601471.1)	432.87 (399.23,469.58)	441721.8 (391901.3,505338.7)	223.88 (195.43,261.15)	-2.34 (-2.45,-2.24)
Ghana	24291.4 (19663.3,29424.7)	423.16 (348.72,504.62)	62153.2 (51124.1,75665.7)	387.94 (322.49,459.15)	-0.48 (-0.56,-0.41)
Greece	32137.2 (27040.4,37731)	215.21 (182.55,251.06)	31921.8 (28143.7,35863.1)	124.49 (108.66,140.87)	-1.99 (-2.12,-1.86)
Greenland	86.3 (66.6,108.7)	257.62 (202.28,324.94)	149.4 (113.2,193.8)	223.4 (175.22,284.24)	-0.52 (-0.59,-0.45)
Grenada	282.6 (221.3,352.6)	384.96 (303.12,479.49)	431.8 (341.4,544)	395.82 (313.85,493.35)	0.17 (0.12,0.22)
Guam	216 (169.2,273.4)	318.35 (249.17,407.28)	826.1 (647.6,1046)	381.86 (301.27,479.77)	0.63 (0.6,0.67)
Guatemala	9246.8 (7492,11406.9)	284.07 (231.57,345.87)	30851.1 (25193.6,37590.7)	288.62 (236.43,349.71)	0.05 (-0.02,0.12)
Guinea	10998.4 (8727.6,13748.3)	350.76 (279.63,431.25)	19975.5 (16139.5,24436.4)	369.17 (299.07,447.29)	0.23 (0.2,0.26)
Guinea-Bissau	1410.1 (1100,1773.5)	394.78 (308.38,497.42)	2714 (2134.8,3364.4)	405.01 (321.49,508.41)	0.08 (0.07,0.08)
Guyana	1549.8 (1248.8,1922.9)	414.38 (329.14,516.68)	2460.3 (1961.3,3068)	397.67 (315.74,495.41)	-0.11 (-0.13,-0.08)
Haiti	13229.5 (10853.3,15816.6)	439.75 (363.02,521.54)	28237.3 (23203.2,34102.2)	401.8 (337.38,473.47)	-0.26 (-0.32,-0.2)
Honduras	6534.1 (5263.6,8021.5)	326.84 (260.07,401.62)	22259.1 (18422.5,26788.2)	365.94 (304.09,434.34)	0.5 (0.43,0.57)
Hungary	80969.9 (71100.2,95051.1)	571.99 (504.75,661.52)	75516 (65982.8,87501.2)	382.61 (333.99,448.41)	-1.3 (-1.36,-1.23)
Iceland	742.3 (588.7,913.4)	265.91 (212.27,327.43)	1083.1 (854.5,1360.3)	188.79 (148.97,235.24)	-1.59 (-2.02,-1.16)
India	2704532.1 (2148026.3,3347899.6)	607.77 (484.82,748.75)	6849890.3 (5528403.4,8331623.2)	585.23 (470.56,713.17)	-0.14 (-0.23,-0.06)
Indonesia	211687.4 (168424.2,260333.6)	240.21 (192.05,295.2)	582119.6 (463533.4,718983.1)	270.65 (215.27,331.46)	0.61 (0.51,0.71)
Iran (Islamic Republic of)	243700.7 (190111.3,308621.5)	999.15 (787.7,1227.71)	683488.7 (547533.1,847574.6)	887.06 (698.93,1089.01)	-0.85 (-1.07,-0.63)
Iraq	80618.9 (69648.9,94680.4)	1029.54 (889.6,1214.45)	242772.5 (207509.9,286008.4)	1040.55 (915.13,1202.99)	-0.07 (-0.12,-0.02)
Ireland	13451.4 (11413,15822.9)	332.29 (284.08,389.15)	13406.3 (10774.3,16514.8)	170.13 (136.02,208.82)	-2.64 (-2.88,-2.4)
Israel	13117.5 (10816.6,15753.6)	275.55 (230.53,330.98)	18843.6 (15058.1,23169.1)	153.19 (122.65,188.99)	-2.2 (-2.39,-2.01)
Italy	227442.3 (178385.7,281938.5)	262.22 (206.08,325.3)	226379.5 (187770,263972.4)	166.39 (138.51,194.88)	-1.71 (-1.8,-1.62)
Jamaica	6304.7 (4977.5,7857.8)	353.64 (278.61,441.33)	11753.2 (9313.3,14653)	368.87 (289.31,457.19)	0.09 (0.04,0.14)

Japan	150408.7 (118335.3,188174.4)	91.85 (72.89,114.39)	358365 (279729,453220.8)	90.63 (71.46,113.97)	-0.3 (-0.46,-0.13)
Jordan	12631 (9874.4,15961.2)	973.25 (770.26,1198.42)	70386.2 (56434.2,89067.6)	913.43 (737.79,1134.18)	-0.52 (-0.63,-0.4)
Kazakhstan	74539.3 (64822.3,85938.3)	631.54 (553.86,718.39)	94339.7 (80747.4,112583.9)	584.39 (515.35,677.98)	-0.67 (-0.96,-0.38)
Kenya	27469.7 (21590.2,34739.6)	349.46 (270.75,444.77)	76757.8 (59808.2,96609)	349.26 (271.66,443.54)	-0.04 (-0.07,-0.01)
Kiribati	135.5 (106.8,172.3)	427.84 (332.78,548.02)	263.9 (203.2,337.4)	424.76 (331.65,544.55)	0.02 (-0.01,0.04)
Kuwait	6836.7 (5311.4,8594.4)	1093.38 (851.47,1394.25)	33843.9 (26412.3,42606.5)	1061.15 (836.06,1330.52)	-0.3 (-0.41,-0.2)
Kyrgyzstan	16278.9 (13136.5,19800.3)	574.33 (468.95,692.84)	25645.2 (21397.6,30618.9)	594.1 (510.66,697.78)	0.17 (0.07,0.27)
Lao People's Democratic Republic	4693.6 (3670.7,5867.5)	261.1 (208.7,320.85)	10184.4 (8109.8,12379.4)	250.17 (205.22,302.23)	-0.03 (-0.12,0.06)
Latvia	17561.7 (15221.1,20284.2)	501.13 (438.66,574.32)	16076.3 (13603.4,19114.3)	385.1 (320.59,460.54)	-1.26 (-1.58,-0.94)
Lebanon	20895.8 (16917.4,25633.1)	1002.51 (817.31,1199.42)	56100.5 (45621.5,66902.1)	931.42 (756.1,1127.78)	-0.23 (-0.27,-0.18)
Lesotho	2497.2 (1946,3161.1)	308.76 (241.17,390.8)	3419.3 (2653.5,4315.3)	337.23 (262.89,422.87)	0.34 (0.32,0.37)
Liberia	4226 (3280.6,5271.9)	390.87 (304.85,486.9)	8199.3 (6486.8,10110.9)	397.86 (309.19,493.34)	-0.01 (-0.03,0.02)
Libya	16432.4 (13153.1,20438.9)	880.78 (701.38,1083.97)	48971.5 (39601.2,60741.5)	922.52 (746.18,1115.01)	-0.02 (-0.1,0.06)
Lithuania	24844.7 (22117.8,27430.2)	559.23 (498.28,616.78)	25609.7 (22498.4,29264.8)	413.59 (360.41,476.27)	-0.68 (-1,-0.36)
Luxembourg	783.2 (618.7,982.1)	145.57 (115.57,180.51)	1243.9 (972.5,1568.3)	117.08 (91.6,147.19)	-0.65 (-1.35,0.06)
Madagascar	16055.9 (12838.6,19870.8)	344.32 (276.47,418.75)	36272.5 (28948.4,44657.8)	346.21 (282.01,415.54)	-0.04 (-0.07,-0.01)
Malawi	12043 (9422.4,15150.4)	336.54 (264.44,416.87)	24179.7 (19277.3,30063.6)	335.7 (268.96,412.55)	-0.2 (-0.27,-0.12)
Malaysia	26997 (22650.4,31701.2)	310.83 (261.41,366.6)	84419.1 (72293.8,99082.1)	313.1 (271.03,364.9)	-0.09 (-0.2,0.02)
Maldives	181.7 (138.7,229.1)	238.83 (184.28,305.01)	723.3 (581.9,885.3)	221.43 (173.67,279)	-0.26 (-0.34,-0.19)
Mali	12944.4 (10217.2,16327.5)	359.77 (286.2,449.89)	29119.4 (23008.9,36573.3)	351.99 (284.45,433.23)	-0.17 (-0.22,-0.13)
Malta	677.4 (537.5,852.3)	162.3 (128.49,202.85)	1148.9 (900.2,1462.5)	121.22 (94.34,152.42)	-1.06 (-1.69,-0.42)
Marshall Islands	53.1 (41.5,67.3)	367.9 (289.35,471.4)	112.6 (86.2,143.4)	381.21 (298.36,483.58)	0.11 (0.09,0.14)
Mauritania	3832.9 (3033.9,4812.7)	407.44 (319.76,508.2)	8307.8 (6497.3,10438)	398.48 (311.12,498.68)	-0.17 (-0.21,-0.12)
Mauritius	1825.5 (1430.7,2276.6)	276.76 (221.92,343.42)	3980.5 (3102.9,5047)	224.16 (174.89,279.31)	-1.12 (-1.26,-0.98)
Mexico	146333.3 (117620.6,178933.8)	350.94 (278.6,435.25)	412013.6 (329516.2,507005.8)	329.42 (261.01,403.11)	-0.3 (-0.37,-0.24)
Micronesia (Federated States of)	172.7 (134.7,218.9)	392.41 (305.34,501.7)	253.3 (194.5,322.6)	394.33 (309.18,495.8)	0.1 (0.06,0.13)
Monaco	121.6 (94.2,157.2)	180.04 (140.66,228.91)	145.9 (115.8,187.7)	155.29 (122.39,196.46)	-0.62 (-0.68,-0.55)
Mongolia	7216.9 (5877.1,8774.1)	754.07 (614.27,912.76)	13690.9 (11006.5,16561.7)	669.06 (547.48,799.77)	-0.9 (-1.09,-0.7)
Montenegro	2162.9 (1704.1,2689.1)	351.59 (278.56,438.44)	3457.6 (2669.7,4389.1)	364.68 (288.85,453.3)	0.22 (0.17,0.27)
Morocco	139257.7 (121259.1,161693.9)	1007.64 (875.89,1163.15)	333143.1 (288945.2,389485.7)	995.04 (874.59,1143.15)	0 (-0.06,0.05)
Mozambique	19021.8 (14742.8,23994.2)	338.36 (265.53,419.97)	35337.4 (27963.8,44055)	326 (257.17,403.42)	-0.3 (-0.39,-0.21)

Myanmar	58350.4 (48513.5,70119.8)	282.21 (239.92,334.75)	108165.2 (92677.7,128823.2)	241.69 (208.57,279.86)	-0.51 (-0.59,-0.42)
Namibia	2190.1 (1673.9,2780.2)	362.9 (282.31,456.58)	4567.7 (3558.9,5719)	344.67 (269.37,434.93)	-0.24 (-0.27,-0.22)
Nauru	15.5 (11.9,19.5)	397.68 (311.2,508.37)	20.8 (16.2,26.5)	416.09 (325.25,526.98)	0.13 (0.11,0.14)
Nepal	49265 (39088.5,61536.2)	539.48 (431.43,669.66)	116482 (97300.5,143066.7)	511.87 (431.32,615.36)	-0.19 (-0.23,-0.15)
Netherlands	61941.8 (53549.6,72206.6)	313.21 (270.67,368.01)	60149.9 (49039.2,73235.2)	178.54 (145.74,217.28)	-2.29 (-2.49,-2.08)
New Zealand	18396.2 (14430.9,23252.1)	474.92 (372.76,600.93)	21454 (18018.6,25603)	257.38 (214.88,304.93)	-2.54 (-2.74,-2.35)
Nicaragua	4866.6 (3886.6,6025.7)	318.28 (253.23,393.82)	15050.7 (12177.4,18364.8)	309.06 (251.08,377.51)	-0.11 (-0.13,-0.09)
Niger	9227.3 (7261.9,11539.9)	363.72 (285.78,451.75)	27301.3 (21724.7,34139.7)	350.99 (282.03,429.69)	-0.23 (-0.29,-0.18)
Nigeria	156801.9 (124155.6,196251.4)	374.98 (294.97,470.63)	344837.1 (275335.2,426401.4)	389.43 (306.22,488.32)	0.03 (-0.01,0.06)
Niue	8.7 (6.8,10.9)	375.64 (293.68,472.47)	8.2 (6.4,10.5)	390.06 (306.84,494.72)	0.11 (0.09,0.12)
North Macedonia	6780.8 (5409.8,8344.7)	378.22 (306.4,459.47)	11569.8 (9193.9,14313.3)	391.57 (322.76,471.36)	-0.01 (-0.08,0.06)
Northern Mariana Islands	57.9 (45.6,72.8)	371.81 (288.64,475.89)	183 (137.4,237.8)	394.3 (306.25,508.26)	0.22 (0.19,0.25)
Norway	19343.3 (15363.3,24327.8)	282.46 (224.8,351.06)	16782.8 (13119.4,21369.1)	169.91 (133.41,214.59)	-1.98 (-2.16,-1.79)
Oman	6676.3 (5440.9,8067.6)	1019.25 (823.84,1229.62)	21787 (17318.8,27024.2)	1027.08 (829.69,1253.84)	-0.15 (-0.27,-0.04)
Pakistan	351002.9 (281282,428891.3)	635.06 (508,780.59)	823021.5 (669034.4,1004960.1)	673.64 (540.86,825.31)	0.15 (0.05,0.26)
Palau	30.5 (23.9,38.8)	344.96 (271.48,437.49)	78.1 (59.9,100.7)	388.02 (305.58,500.84)	0.41 (0.38,0.45)
Palestine	7958.6 (6337.1,9894.6)	958.71 (769.23,1179.16)	23442.5 (18613.9,29518.8)	942.08 (762.36,1163.09)	-0.15 (-0.19,-0.12)
Panama	4579.7 (3676.4,5650.8)	308.23 (246.14,381.5)	13390.3 (10500.5,16736.2)	298.49 (234.77,372.79)	-0.18 (-0.21,-0.15)
Papua New Guinea	5428.3 (4183.4,6836.6)	354.93 (274.22,447.25)	16162.1 (12719.5,20163.4)	356.49 (280.14,441.74)	-0.02 (-0.06,0.01)
Paraguay	4138.3 (3314.3,5096.9)	184.95 (147.4,227.47)	10512.3 (8381.3,12938.8)	178.3 (142.09,218.31)	-0.15 (-0.17,-0.12)
Peru	29487.4 (24415.4,35869.8)	246.82 (202.19,299.47)	76549.8 (62017.3,93381.2)	222.24 (178.43,271.49)	-0.47 (-0.58,-0.36)
Philippines	58410.7 (46432.4,73225)	218.71 (171.46,279.17)	135219 (107152.1,168061.8)	177.16 (140.1,219.91)	-0.67 (-0.92,-0.43)
Poland	199624.8 (157435.5,247815.6)	475.27 (379.93,588.16)	158482.4 (135882.2,185359.1)	227.31 (195.03,261.71)	-2.98 (-3.26,-2.69)
Portugal	23767.8 (19781.4,28204.2)	178.44 (150.66,208.67)	18180.9 (15526.5,21336.5)	72.93 (61.36,85.88)	-3.44 (-3.78,-3.11)
Puerto Rico	14129.7 (11527.4,16908.8)	400.38 (328.97,476.53)	24348.2 (19484.4,30058.3)	351.5 (284.69,430.43)	-0.56 (-0.65,-0.47)
Qatar	1386.8 (1070.7,1750.8)	1066.52 (822.42,1343)	12702.5 (9554.4,16299.1)	1032.07 (800.61,1299.53)	-0.26 (-0.34,-0.19)
Republic of Korea	50995.4 (43011.5,60717)	207.36 (179.12,241.59)	88757.8 (73096.6,107551.4)	96.28 (79.26,116.32)	-2.18 (-2.57,-1.79)
Republic of Moldova	24476 (21286.5,28167.8)	651.77 (571.13,736.02)	30593.8 (26653.5,35235.1)	511.83 (444.58,586.7)	-0.9 (-1.02,-0.79)
Romania	129628.3 (115597.7,147904.9)	518.93 (468.55,576.69)	135974.6 (121076.7,155465.7)	364.29 (322.16,417.78)	-1.75 (-2.09,-1.42)
Russian Federation	1226018.7 (986689.4,1484619.6)	741.29 (599.61,898.59)	1719384.9 (1372804.3,2108619.5)	727.97 (586.2,880.94)	-0.35 (-0.65,-0.04)
Rwanda	7957.8 (6130.6,9999)	309.01 (241.39,387.22)	17769.7 (13958.9,22250.5)	294.24 (231.49,366.29)	-0.37 (-0.43,-0.3)
Saint Kitts and Nevis	140.4 (109.9,180.7)	393.42 (309.27,492.74)	258.3 (198.2,329.7)	387.91 (303.07,485.65)	-0.09 (-0.13,-0.05)

Saint Lucia	347.4 (278.4,435.5)	409.74 (322.58,513.7)	929.3 (733.5,1165.5)	390.38 (309.39,486.87)	-0.06 (-0.1,-0.01)
Saint Vincent and the Grenadines	260.8 (208.1,325.6)	376.48 (299.85,470.16)	519.5 (408.5,650.4)	372.87 (293.7,464.65)	0.04 (0,0.07)
Samoa	310.3 (242.1,395.4)	402.37 (314.76,509.26)	549.2 (426.5,697.2)	410.13 (319.2,524.12)	0.06 (0.05,0.08)
San Marino	61.1 (47.5,77.8)	177.72 (139.17,224.69)	114.6 (89.9,144.9)	154.38 (120.56,195.36)	-0.57 (-0.65,-0.49)
Sao Tome and Principe	233 (181,297.8)	376.16 (292.27,483.55)	417 (324.2,525.7)	389.79 (304.27,497.04)	0.09 (0.06,0.12)
Saudi Arabia	50790.9 (41793.1,61699.5)	877.87 (719.09,1065.04)	202757.8 (167092.4,247500.5)	920.14 (777.97,1085.34)	0.16 (0.04,0.28)
Senegal	12620.9 (9990.7,15814.2)	414.97 (327.23,514.76)	29295.4 (23527.6,35765.2)	390.43 (314.42,472.12)	-0.28 (-0.33,-0.24)
Serbia	50527.9 (44341.4,57831.5)	490.38 (436.47,548.95)	75126.4 (65033.8,87255.6)	468.45 (404.2,544.85)	-0.6 (-0.82,-0.38)
Seychelles	122.3 (94.7,157.9)	216.51 (167.97,277.64)	245.1 (191.5,314.6)	223.44 (175.39,287.93)	0.08 (0.05,0.1)
Sierra Leone	8288.4 (6674.6,10203.2)	424.05 (339.77,523.49)	14295.9 (11499,17591.1)	393.12 (315.9,485.57)	-0.29 (-0.31,-0.27)
Singapore	4559.9 (3652,5644)	218.73 (174.55,270.07)	12728 (9870.6,16139.5)	149.87 (117.59,187.95)	-0.63 (-0.83,-0.43)
Slovakia	30221.6 (26618.9,34464.8)	518.82 (458.13,586.7)	33181.8 (28545.9,38705.7)	357.35 (308.07,412.72)	-1.09 (-1.5,-0.69)
Slovenia	8443.6 (6711,10387.3)	347.64 (276.91,424.34)	12434.5 (9762.3,15795.9)	286.63 (226.17,359.69)	-1.33 (-2.14,-0.51)
Solomon Islands	475.8 (363.3,600.6)	407.89 (316.18,515.29)	1304.5 (1028.7,1626.1)	426.82 (330.7,533.32)	0.31 (0.23,0.4)
Somalia	7641.4 (5930.9,9645.6)	343.19 (270.73,430.99)	19652.7 (15353.2,24755.2)	337.69 (266.5,416.64)	-0.16 (-0.21,-0.12)
South Africa	85209.1 (67282.3,106271.4)	419.96 (329.42,528.11)	172647.2 (136318.9,217054.3)	380.13 (300.49,471.61)	-0.52 (-0.61,-0.43)
South Sudan	7535.7 (5925.2,9449.4)	312.41 (243.43,387.34)	12117.9 (9497.5,15295.3)	327.21 (259.95,413.78)	0.1 (0.06,0.13)
Spain	123713.7 (109744.9,138770)	234.68 (211.28,261.42)	138476.5 (120629.4,157997.5)	143.48 (122.62,167.12)	-1.48 (-1.7,-1.25)
Sri Lanka	26737 (22100,32134.6)	278.37 (231.96,331.75)	63890 (53189.1,75780.9)	248.5 (210.55,290.2)	-0.31 (-0.43,-0.19)
Sudan	95095 (81198.7,112123.6)	1067.64 (916.41,1248.86)	188177.6 (162200.6,221428.6)	973.5 (845.4,1130.98)	-0.39 (-0.43,-0.34)
Suriname	1026.2 (823,1265.9)	404.93 (323.51,504.26)	2525 (1997.1,3179.4)	404.09 (321.1,502.45)	-0.03 (-0.06,-0.01)
Sweden	42469.6 (38016.1,47370.1)	273.53 (247.79,301.55)	48825.5 (38765.1,60987.9)	226.81 (177.47,284.75)	-1.48 (-1.75,-1.22)
Switzerland	26352.3 (23068.4,30202.5)	251.72 (220.95,286.9)	28103.4 (23602.7,33779.7)	149.87 (122.63,182.47)	-1.4 (-1.75,-1.05)
Syrian Arab Republic	54365.2 (46712.8,64584.1)	1085.68 (945.14,1279.35)	140406.7 (120647.4,166778.9)	1130.3 (993.53,1305.6)	0.05 (0.01,0.1)
Taiwan (Province of China)	51000.9 (40349.3,62197.4)	353.29 (285.92,424.17)	128360.5 (101804,161586.7)	303.19 (243.88,375.54)	-0.72 (-0.82,-0.62)
Tajikistan	15385.7 (12646,18856.6)	587.55 (486.5,715.97)	37233.6 (31500.8,43789.1)	774.65 (678.93,883.79)	1.09 (1.01,1.18)
Thailand	69378.4 (56690.3,83548.9)	218.79 (182.14,258.62)	184313.4 (151705,222381.1)	169.58 (139.96,200.89)	-1.14 (-1.26,-1.03)
Timor-Leste	510.8 (402.3,636.2)	211.13 (164.73,271.09)	1753.6 (1369.7,2216.7)	221.76 (174.75,277.01)	0.37 (0.28,0.45)
Togo	4516.7 (3565.6,5585)	394.03 (310.04,490.77)	13911.6 (10996.7,17354.2)	386.94 (305.72,475.8)	-0.13 (-0.17,-0.08)
Tokelau	4.2 (3.2,5.4)	328.03 (256.69,415.48)	5.6 (4.3,7.2)	373.08 (292.48,478.98)	0.43 (0.42,0.44)

Tonga	195.5 (152.6,248.1)	386.93 (302.69,493.89)	315.7 (247.1,402.4)	403.04 (315.34,515.14)	0.14 (0.12,0.15)
Trinidad and Tobago	3691.1 (2990.1,4552.1)	451.35 (363.67,558.53)	8005 (6302.4,10020)	422.86 (336.91,521.43)	-0.39 (-0.43,-0.34)
Tunisia	41617.7 (33409,51173.4)	869.56 (708.79,1051.43)	111132.6 (92383.2,136953.8)	847.53 (711,1026.1)	-0.24 (-0.3,-0.18)
Turkmenistan	12202.6 (10113.9,14558.7)	714.65 (598.11,837.12)	25494.7 (21370.5,30520.6)	689.66 (588.79,796.61)	-0.75 (-0.86,-0.64)
Tuvalu	20.3 (15.5,26.2)	354.2 (274.93,453.21)	36.9 (28.8,47.6)	385.73 (301.45,495.08)	-0.44 (-0.57,-0.31)
Timor-Leste	287173.7 (248897.8,339689.6)	824.26 (718.28,963.21)	621078.9 (527076.8,740295.2)	665.07 (568.25,784.58)	0.3 (0.29,0.31)
Uganda	18935 (15148.2,23658.5)	310.75 (247.3,384.67)	42188.6 (33807.2,52288.9)	291.89 (235.73,356.69)	-0.41 (-0.49,-0.32)
Ukraine	455872.1 (369123.8,558151.9)	681.85 (558.42,831.1)	587712.6 (471323.3,730070.4)	752.32 (608.03,916.09)	0.13 (-0.1,0.37)
United Arab Emirates	5522 (4261.7,6894.8)	1084.94 (843.51,1370.02)	62589.1 (45589.5,80890.7)	1084.31 (843.39,1343.89)	-0.11 (-0.15,-0.07)
United Kingdom	258729.5 (208292.8,318199.6)	287.52 (232.83,354.16)	176843.9 (143926.4,213528.9)	141.62 (115.22,170.01)	-2.12 (-2.48,-1.77)
United Republic of Tanzania	30173.3 (24096.1,37679.3)	295.79 (239.24,361.35)	80953 (65750.3,100556.1)	321.55 (264.22,386.47)	0.24 (0.2,0.29)
United States of America	1452572.3 (1131039.1,1825893.5)	461.63 (361.24,582.38)	967989.8 (814313,1134212)	170.36 (143.9,197.67)	-3.66 (-3.94,-3.38)
United States Virgin Islands	300 (237,375.3)	366.52 (286.33,459.67)	669.3 (519.6,860.6)	384.68 (303.56,482.22)	0.28 (0.23,0.33)
Uruguay	9941.1 (8117.3,11952.5)	257.65 (211.48,308.28)	11404.5 (9039.2,14140.6)	204.18 (159.75,254.81)	-0.96 (-1.1,-0.82)
Uzbekistan	64012.3 (55945.5,73762.8)	581.99 (511.67,667.68)	246907.7 (225966.2,273978)	1206.01 (1125.02,1305.45)	2.52 (2.15,2.9)
Vanuatu	231.3 (179.9,291.8)	430.05 (334.59,542.09)	696.3 (541.7,876.5)	456.38 (354.88,574.23)	0.24 (0.21,0.27)
Venezuela (Bolivarian Republic of)	35399.1 (29607.3,41976.5)	368.65 (308.13,435.17)	97268.4 (83418.2,114140.4)	323.97 (279.3,375.28)	-0.56 (-0.65,-0.48)
Viet Nam	83462 (70352.2,99884.9)	219.4 (186.79,257.41)	215889.6 (185841.5,255361)	232.72 (201.98,270.45)	0.38 (0.3,0.46)
Yemen	47748 (40172.8,56681.1)	1016.65 (871.98,1183.22)	130404.9 (111990.2,153887.2)	948.5 (819.7,1114.36)	-0.31 (-0.36,-0.25)
Zambia	8606.2 (6827.9,10734.7)	322.5 (254.42,404.23)	20713 (16463,25608.4)	305.26 (242.23,377.4)	-0.36 (-0.43,-0.29)
Zimbabwe	13650.8 (10997.7,17141.2)	352.72 (282.07,435.2)	25187.8 (19946,31407.6)	389.53 (313.51,469.31)	0.39 (0.31,0.47)

Table S3 The mortality of ischemic heart disease cases and age-standardized rate at national level in 1990 and 2021, with EAPC (1990-2021).

Location	1990		2021		EAPC (95% CI)
	Number	ASR	Number	ASR	
Afghanistan	22339 (16819.2,27876.8)	361.39 (279.98,444.56)	23705.1 (18124.5,29907.9)	280.03 (221.41,346.2)	-0.98 (-1.08,-0.89)
Albania	2960.1 (2635,3261.7)	174.23 (153.51,192.16)	6440.2 (5465.5,7543.3)	158.29 (134.2,185.54)	0.09 (-0.12,0.3)
Algeria	24545.4 (20991.5,28788.1)	310.63 (267.49,359.89)	52898.3 (42964.1,63610.3)	212.02 (172.72,251.71)	-1.21 (-1.26,-1.16)
American Samoa	31.6 (28.5,34.7)	166.5 (150.04,183.69)	75.4 (64.9,88.1)	178.99 (155.05,207.56)	0.37 (0.32,0.43)
Andorra	40.9 (30.4,54)	83.57 (62.19,109.06)	71.8 (52.7,92.8)	41.02 (30.25,52.92)	-2.06 (-2.29,-1.83)
Angola	3832 (3014.2,4772.7)	126.92 (102.51,155.46)	10991.1 (8415,13808)	125.48 (99.59,156.4)	-0.24 (-0.32,-0.15)
Antigua and Barbuda	72.1 (67.2,76.5)	127.16 (118.69,134.92)	66.4 (62.1,73.1)	71.43 (66.59,77.89)	-2.17 (-2.39,-1.96)
Argentina	46663.2 (44422.9,48177.5)	158.8 (149.31,164.64)	35109.8 (32241.9,37063.9)	60.79 (55.95,64.11)	-2.68 (-2.86,-2.5)
Armenia	7035.9 (6608.1,7396.3)	307.44 (286.83,323.83)	9023.7 (8034.4,10044.7)	209.78 (186.39,233.39)	-1.98 (-2.2,-1.75)
Australia	32990.1 (30572,34270.3)	175.07 (160.93,182.55)	23031.9 (19557.7,24978.1)	44.08 (37.95,47.53)	-4.68 (-4.79,-4.57)
Austria	20058.7 (18449.4,20820.2)	163.5 (150.03,169.73)	15282.8 (13004.2,16523.8)	68.11 (59.02,73.18)	-3.16 (-3.35,-2.98)
Azerbaijan	15240.9 (14226.8,16284.5)	362.31 (337.11,388.22)	23930.5 (21062.9,26988.7)	306.13 (270.78,343.48)	-0.37 (-0.57,-0.18)
Bahamas	187.1 (173.7,200.1)	130.35 (120.96,139.35)	280.2 (232.3,340.4)	76.31 (63.63,92.15)	-1.89 (-2.08,-1.7)
Bahrain	474.1 (445.6,506.2)	408.07 (381.77,433.03)	830.8 (706.5,958.5)	161.62 (140.64,184.38)	-3.56 (-3.94,-3.18)
Bangladesh	54138.2 (46605.1,63036.8)	119.12 (101.74,138.51)	134940.5 (106732.2,166688.6)	107.5 (86.2,131.71)	-0.19 (-0.33,-0.06)
Barbados	362.4 (339.1,379.8)	120.8 (112.91,126.46)	325.1 (266.9,389)	62.55 (51.4,74.63)	-2.26 (-2.57,-1.95)
Belarus	40082.2 (37620.3,41507.5)	331.13 (309.29,343.52)	55916.1 (47853.6,64633.9)	341.51 (292.04,394.62)	-0.3 (-0.61,0.02)
Belgium	21029.8 (19310.4,22090.8)	134.49 (123.19,141.36)	10792.6 (9132.8,11753.5)	38.04 (33.11,40.92)	-4.09 (-4.24,-3.94)
Belize	109.4 (101.5,115.8)	119.19 (110.42,126.07)	179 (158.2,198.2)	66.97 (59.34,74.28)	-2.42 (-2.77,-2.06)
Benin	1287.8 (1087.8,1505.8)	73.34 (62.22,86)	3230.7 (2659.1,3942)	77.65 (65.25,93.07)	0.24 (0.08,0.41)
Bermuda	139.7 (132,146.9)	245.2 (230.52,257.73)	103.7 (89.2,123)	68.35 (59.38,81.13)	-4.11 (-4.44,-3.78)
Bhutan	226.5 (167.1,292.9)	106.73 (78.16,137.3)	624.4 (479.1,759)	110.98 (86.52,133.92)	0.2 (0.17,0.24)
Bolivia (Plurinational State of)	3786.8 (2950.8,4972.3)	142.74 (113.77,184.17)	6422.8 (4613.8,9294.8)	84.63 (62.4,121.7)	-1.6 (-1.83,-1.37)
Bosnia and Herzegovina	6795.5 (6208.5,7344.1)	200.09 (181.52,217.01)	8774.3 (7268.6,10281.9)	138.3 (114.1,162.53)	-1.6 (-1.9,-1.3)
Botswana	434.6 (327.7,547.7)	101.42 (75.69,127.42)	907.5 (697.6,1145)	78.6 (62.09,98.66)	-0.86 (-1.12,-0.6)
Brazil	105307.1 (100047.1,108323.4)	136.61 (127.24,141.52)	157550.1 (144833.1,165499.4)	64.01 (58.63,67.35)	-2.32 (-2.41,-2.22)
Brunei Darussalam	134.4 (120.2,150.4)	149.62 (132.75,166.48)	226.9 (200,255.5)	84.5 (74.32,95.88)	-1.42 (-1.66,-1.17)
Bulgaria	34133.5 (32738.4,35420)	403.98 (387.8,416.73)	28584.2 (25170.7,32291.8)	204.4 (180.5,230.3)	-2.85 (-3.18,-2.51)

Burkina Faso	2949.8 (2401,3633.4)	84.03 (68.86,103.12)	7291.4 (5599.8,9405.8)	97.57 (75.51,124.07)	0.75 (0.61,0.89)
Burundi	2164.7 (1649.8,2718.3)	104.94 (80.73,131.97)	3430.1 (2681.6,4379)	85.8 (68,108.32)	-1.17 (-1.36,-0.98)
Cabo Verde	171.3 (142,198.8)	71.83 (59.6,82.89)	502.5 (414.7,589.4)	121.51 (100.46,142.26)	1.14 (0.76,1.52)
Cambodia	4438.2 (3751.3,5232.2)	115.73 (97.89,135.07)	10999.6 (8639.5,13511.1)	111.55 (89.23,134.56)	-0.17 (-0.26,-0.09)
Cameroon	2799.3 (2167.2,3678.6)	79.88 (61.99,104.29)	10107.3 (7668.9,13637.9)	103.29 (79.89,137.12)	0.95 (0.41,1.49)
Canada	50105.5 (46274.4,51988.6)	156.66 (143.99,162.88)	41493.6 (36498.6,44418.7)	51.12 (45.43,54.44)	-3.88 (-4.03,-3.73)
Central African Republic	1495.2 (1143.9,2068.7)	165.96 (127.17,223.28)	2548.8 (1833.4,3656.4)	150.26 (109.43,210.57)	-0.42 (-0.5,-0.35)
Chad	2182.1 (1747,2741.1)	87.87 (70.37,109.87)	4858 (3684.2,6234.7)	104.29 (80.34,131.28)	0.47 (0.26,0.68)
Chile	10269.3 (9716.7,10639.8)	118.48 (111.12,122.93)	10187.8 (9310.8,10787.3)	39.05 (35.77,41.32)	-3.41 (-3.53,-3.28)
China	547845.1 (486106.5,617005.7)	94.14 (84.01,105.89)	1956859.4 (1634477.6,2280131.2)	110.91 (92.42,128.56)	0.97 (0.64,1.29)
Colombia	22138.9 (20992.8,22807.4)	143.52 (134.9,148.41)	44886.3 (37733,53007.9)	79.05 (66.64,93.24)	-2.13 (-2.32,-1.93)
Comoros	125.7 (94.4,165.2)	77.26 (60.01,98.64)	304 (228.4,399.1)	73.16 (55.29,95.05)	-0.44 (-0.56,-0.33)
Congo	1589 (1244.7,1970.3)	186.73 (149.01,224.12)	3249.1 (2527.6,4063)	158.95 (127.71,189.82)	-0.8 (-0.93,-0.67)
Cook Islands	17.5 (15.2,20.1)	160.27 (141.38,183.71)	26.7 (22.2,31.7)	107.15 (88.99,128.05)	-1.25 (-1.37,-1.13)
Costa Rica	2031.1 (1878.1,2133.2)	124.75 (114.39,131.17)	3110.7 (2708.1,3459)	55.1 (48.17,61.1)	-2.65 (-2.82,-2.48)
Croatia	14728.8 (14007.4,15366.3)	287.39 (271.39,300.65)	12816.3 (11288,14065)	130.52 (114.93,143.14)	-0.06 (-0.29,0.17)
Cuba	20428.3 (19512.1,20941.5)	214.24 (203.04,220.24)	21904.1 (19098.6,24566.9)	104.76 (91.34,117.29)	-2.6 (-2.72,-2.47)
Cyprus	1424.6 (1280.3,1578.4)	271.51 (244.97,299.09)	1479.9 (1278,1678.8)	86.95 (75.85,98.11)	-2.56 (-2.86,-2.27)
Czechia	39784.2 (38181.5,40977.4)	296.03 (282.63,305.49)	29571.5 (25743.2,32539)	126.68 (110.35,139.62)	-4.03 (-4.23,-3.83)
Cmte d'Ivoire	3478 (2763.9,4274)	118.09 (97.26,141.65)	10245.7 (8081.3,13569.1)	119.9 (98.75,152.67)	-2.56 (-2.66,-2.45)
Democratic People's Republic of Korea	13865 (10544.9,17353.3)	112.65 (86.11,137.19)	36374.7 (29083.9,44603.9)	125.35 (100.79,153.33)	0.38 (0.19,0.56)
Democratic Republic of the Congo	15433.7 (11448,20347.3)	130.63 (98.95,169.53)	31477.5 (23251.8,42274)	112.32 (83.28,149.12)	-0.65 (-0.74,-0.55)
Denmark	18311.4 (17083.5,18992.2)	212.53 (198.63,220.27)	5359.1 (4635,5757.7)	39.95 (34.9,42.81)	-5.83 (-5.99,-5.66)
Djibouti	72.8 (50.7,98.6)	69.59 (50.2,92.41)	447.6 (324.2,601.3)	91.49 (67.7,118.94)	0.8 (0.67,0.94)
Dominica	86.3 (80.1,92.4)	151.5 (140.13,162.34)	71.2 (62.6,83.7)	92.21 (81.49,107.84)	-1.81 (-2.07,-1.55)
Dominican Republic	4622.2 (4107.3,5175.1)	150.36 (133.62,167.93)	14089.3 (11378.1,17320)	144.66 (116.75,177.94)	0.54 (0.31,0.77)
Ecuador	4570.2 (4365.9,4719.8)	99.86 (94.74,103.33)	11432.3 (9351.4,13903.1)	77.47 (64.11,93.12)	-0.89 (-1.44,-0.34)
Egypt	76014.2 (69574.3,83588.6)	379.2 (343.43,420.07)	161696.7 (135632.7,190625)	347.73 (297.31,402.14)	0.2 (-0.01,0.41)
El Salvador	3394.2 (3128.6,3604.8)	118.02 (108.48,125.47)	6388.3 (5261.8,7691.7)	94.74 (78.35,113.9)	-0.79 (-0.99,-0.58)
Equatorial Guinea	252.4 (192.9,322.5)	159.29 (124.09,197.59)	546.4 (377.7,753.1)	141.8 (102.97,188.38)	-0.57 (-0.77,-0.37)

Eritrea	748.6 (544.7,968)	83.07 (60.89,105.6)	1994.2 (1464.8,2622.6)	90.95 (69.21,117.14)	0.28 (0.2,0.35)
Estonia	7085.2 (6718.6,7351.6)	368.95 (347.37,383.27)	3005.1 (2608.4,3348.8)	92.06 (80.72,102.43)	-4.84 (-5.25,-4.43)
Eswatini	205.2 (162.4,256.5)	90.35 (70.96,112.97)	486.9 (334.6,703.3)	106.44 (75.27,145.87)	1.06 (0.55,1.58)
Ethiopia	13974.7 (11641.6,17944.1)	81.27 (68.45,99.83)	20974.9 (16988.1,25153.3)	55.81 (45.21,66.72)	-1.51 (-1.62,-1.4)
Fiji	939.6 (818.7,1086.5)	291.09 (256.25,329.57)	1711.7 (1352.3,2122.8)	266.78 (216.97,322.31)	-0.5 (-0.64,-0.35)
Finland	15535.4 (14412.6,16111)	215.87 (199.55,224.26)	12172.6 (10105,13275.8)	77.38 (65.63,83.77)	-3.28 (-3.35,-3.22)
France	74095.1 (68294.2,77384.5)	84.26 (77.7,87.99)	56002.4 (47365.5,60965)	29.87 (25.89,32.3)	-3.48 (-3.6,-3.36)
Gabon	669.3 (532.5,797.5)	135.27 (108.08,162.06)	1036.9 (789.3,1301.7)	126.77 (99.33,155.15)	-0.33 (-0.45,-0.21)
Gambia	314.8 (246.2,399.7)	112.57 (90.28,140.89)	1139.2 (874.8,1408.8)	140.02 (108.58,171.76)	0.61 (0.47,0.75)
Georgia	20225.8 (19156.7,21126.9)	358.65 (337.55,374.96)	7869.7 (7029.6,8675.6)	124.16 (111.04,136.51)	-4.01 (-4.41,-3.61)
Germany	254432.2 (233570.9,265106.5)	192.4 (176.05,200.95)	150616.9 (129273.9,163023.3)	63.67 (55.51,68.32)	-3.72 (-3.84,-3.61)
Ghana	7140.5 (5869.3,8577)	142.8 (119.35,169.63)	12867.1 (10265.7,15724.6)	97.56 (78.35,118.19)	-1.76 (-2.09,-1.43)
Greece	20546.1 (19202.7,21346.3)	142.04 (132.21,147.9)	21284 (18464.1,22780.5)	72.9 (65.2,76.93)	-2.33 (-2.64,-2.02)
Greenland	54.5 (50.1,59.3)	210.19 (192.06,230.77)	46.3 (40.6,55.2)	81.2 (70.57,97.64)	-3.17 (-3.34,-2.99)
Grenada	110 (100.6,119.5)	143.97 (131.72,156.44)	96.3 (84.3,107.7)	95.36 (83.86,105.78)	-1.84 (-2.14,-1.55)
Guam	112.9 (104.4,120.9)	203.55 (187.2,218.36)	270.4 (239.7,299.3)	126.6 (113.51,139.39)	-0.74 (-1.07,-0.4)
Guatemala	3801.4 (3675.5,3907.3)	149.15 (142.83,153.93)	8870.9 (7757.2,10016.4)	97.26 (85.34,109.04)	-1.36 (-1.77,-0.94)
Guinea	2666.1 (2152.8,3260)	92.1 (73.83,112.19)	5252.9 (4026.2,6793.7)	110.41 (85.87,140.24)	0.84 (0.71,0.98)
Guinea-Bissau	496.9 (389.3,630.1)	150.59 (120.96,186.49)	872.7 (672.7,1086)	156.92 (123.29,192.38)	0.2 (0.11,0.29)
Guyana	781 (706.2,859)	230.59 (209.18,251.95)	810.9 (646,1014.8)	143.23 (115.5,177.3)	-1.3 (-1.56,-1.03)
Haiti	6915.9 (5794.6,8181.9)	264 (219.99,304.41)	12182.7 (9069.8,16004.3)	210.11 (159.57,271.2)	-0.57 (-0.65,-0.48)
Honduras	1900.3 (1658.3,2147.9)	112.05 (96.5,126.75)	8525.2 (7076.6,10305)	167.93 (139.63,201.92)	1.49 (1.25,1.74)
Hungary	35548.2 (34095.6,36646.6)	262.8 (250.24,271.33)	33245.9 (29341.9,36489.1)	155.36 (137.21,170.61)	-1.71 (-1.85,-1.58)
Iceland	503.3 (456.6,532.2)	166.2 (151.65,175.42)	395.2 (326.6,438.8)	57.95 (49,64.16)	-3.42 (-3.52,-3.32)
India	571505 (511213.9,624257.5)	137.87 (121.54,151.08)	1632881.8 (1487219.4,1788197.9)	151.17 (137.46,165.08)	0.48 (0.3,0.65)
Indonesia	89011.8 (76060.4,101756.3)	102.96 (85.94,119.91)	276493.6 (229238.2,322155.1)	143.25 (119.34,163.3)	1.19 (1.09,1.29)
Iran (Islamic Republic of)	50868.2 (47343.4,53912.2)	259.88 (238.06,276.23)	98297.5 (88892.1,105322.2)	146.11 (130.71,156.95)	-2.08 (-2.22,-1.93)
Iraq	18668.8 (15989.8,21776.7)	249.67 (213.86,290.86)	47232.4 (37101.2,56094)	254.83 (204.17,296.68)	-0.43 (-0.62,-0.25)
Ireland	9014.7 (8506.5,9312.8)	227.01 (212.63,235.11)	4458 (3766.1,4857.6)	52.77 (44.84,57.37)	-4.77 (-4.94,-4.59)
Israel	8054.2 (7514.7,8377.4)	177.3 (163.19,184.77)	4854.4 (4111.6,5260.2)	34.77 (29.93,37.49)	-5.69 (-5.89,-5.48)
Italy	94745.7 (86024,99143.8)	108.5 (97.93,113.85)	85030.6 (70074.6,92946.7)	44.27 (37.56,47.75)	-3.04 (-3.13,-2.95)
Jamaica	1312.9 (1219.8,1372.1)	71.38 (66.35,74.66)	1759.2 (1408.4,2212.9)	52.45 (42.06,66.21)	-0.64 (-1.14,-0.14)

Japan	102221.7 (92558.2,106893.7)	66.69 (59.5,70.22)	123164 (99194.6,136240.4)	25.36 (21.7,27.29)	-2.95 (-3.18,-2.73)
Jordan	2198.5 (1889,2525.5)	199.16 (171.57,228.44)	5682.5 (4651.4,6939.4)	98.28 (80.73,117.66)	-2.83 (-3.14,-2.52)
Kazakhstan	32667 (30425,35005.3)	298.65 (276.16,319.98)	32081.4 (28763.1,35521)	235.98 (212.04,258.87)	-1.88 (-2.44,-1.32)
Kenya	2694.8 (1965.8,3295.1)	39.81 (28.88,49.04)	10922.6 (8344.2,14043.2)	61.5 (46.7,79.16)	1.71 (1.45,1.97)
Kiribati	63.4 (52.1,75.2)	189.64 (154.84,225.35)	130.1 (105.7,160.8)	202.94 (169.31,243.41)	0.24 (0.21,0.28)
Kuwait	1033.2 (952,1109.2)	198.32 (179.17,212.96)	2885.3 (2385.4,3473.3)	109.1 (90.44,131.44)	-1.89 (-2.3,-1.47)
Kyrgyzstan	6874.3 (6331.4,7434.7)	264.38 (242.39,284.65)	10086.2 (8574,11575.6)	274.41 (234.16,313.72)	0.36 (-0.01,0.72)
Lao People's Democratic Republic	4195.5 (3321.7,5225.8)	235.34 (187.6,286.47)	6700.3 (5314.6,8333.2)	176.46 (141.65,213.26)	-1.07 (-1.17,-0.96)
Latvia	11813.2 (11167.8,12279.8)	343.06 (322.5,356.66)	7352.6 (6477.2,8117.7)	160.3 (141.76,177.14)	-2.75 (-3.05,-2.45)
Lebanon	4398.3 (3686.8,5364.3)	235.27 (199.02,287.02)	6045 (5086.3,7075.9)	92.09 (77.66,107.98)	-3.09 (-3.38,-2.79)
Lesotho	362.8 (283.1,468.3)	50.87 (39.57,66.28)	805.8 (543.3,1235.2)	90.9 (63.42,134.25)	2.84 (2.31,3.38)
Liberia	1039.7 (866,1236.7)	107.55 (91.01,125.79)	1891.2 (1462.8,2471.2)	114.25 (90.14,145.7)	0.18 (0.05,0.31)
Libya	2812.3 (2244.9,3459.4)	162.07 (128.59,199.64)	8163.1 (6372.9,10499.6)	178.47 (140.3,228.13)	0.83 (0.59,1.07)
Lithuania	15965 (15057.5,16542.1)	365.29 (343.23,379.01)	13393.7 (11903.1,14673.5)	197.08 (176.61,215.91)	-1.97 (-2.19,-1.74)
Luxembourg	814.8 (769.4,849)	153.83 (144.6,160.39)	551 (485,606.1)	45.7 (40.6,50.17)	-4.01 (-4.16,-3.86)
Madagascar	3664.1 (3112.3,4234.4)	82.43 (70.46,95.52)	9096.4 (6650.1,11694)	100.02 (74.95,125.89)	0.27 (0.12,0.41)
Malawi	2212.7 (1879.6,2602.6)	69.58 (59.67,81.51)	5347.3 (4413.8,6379.2)	84.27 (70.14,99.95)	0.39 (0.13,0.66)
Malaysia	15010.2 (13793.1,16005.5)	177.45 (162.83,189.78)	37820.9 (35174.5,40187.6)	149.66 (138.24,159.74)	-0.64 (-0.8,-0.49)
Maldives	124.1 (109.8,139.4)	167.99 (147.79,188.24)	229.4 (189.9,272.2)	79.43 (66.25,93.77)	-2.8 (-2.91,-2.69)
Mali	2345.4 (1774.1,3029.1)	75.19 (57.11,96.95)	4987.9 (3870.2,6415.4)	71.65 (56.24,90.99)	-0.04 (-0.14,0.06)
Malta	829.1 (774.9,871.3)	207.71 (192.05,218.97)	783.9 (666.6,867.2)	71.19 (61.3,78.48)	-3.43 (-3.62,-3.23)
Marshall Islands	37.3 (32.5,43.1)	263.68 (230.16,301.81)	81.2 (62.9,103.5)	275.9 (219.83,337.91)	0.23 (0.12,0.35)
Mauritania	1169.9 (923.4,1467.2)	136.72 (109.19,169.77)	2016.9 (1506.1,2603.6)	112.23 (85.32,144.29)	-0.95 (-1.14,-0.76)
Mauritius	1431.9 (1356.9,1498.8)	228.18 (214.34,239.27)	1655.1 (1532.8,1727)	97.49 (90.28,102)	-3.21 (-3.48,-2.94)
Mexico	38261.9 (36880,38955.5)	113.39 (108.31,115.87)	129103.5 (114982.5,143052.1)	113.21 (100.89,125.2)	-0.1 (-0.36,0.17)
Micronesia (Federated States of)	116.8 (95,143.2)	268.02 (220.53,324.28)	168.9 (132.5,216.4)	266.43 (213.65,330.4)	0.03 (0.01,0.05)
Monaco	81.6 (64.1,95.7)	103.52 (82.13,122.18)	58.4 (46.7,70.5)	49.33 (40.1,59.33)	-2.48 (-2.56,-2.4)
Mongolia	2787.3 (2438.9,3177.8)	324.79 (286.63,365.71)	3556.8 (3060.9,4029.4)	219.59 (190.33,247.4)	-1.73 (-1.98,-1.48)
Montenegro	956.7 (835.5,1065.1)	164.64 (142.76,183.41)	1800.2 (1576,2028.1)	212.83 (186.68,239.2)	0.97 (0.73,1.2)
Morocco	37963.8 (32567.9,44182.3)	296.2 (254.39,342.55)	78695.8 (61238.5,92878.3)	267.15 (210.84,310.86)	-0.35 (-0.4,-0.3)
Mozambique	1604.2 (1360.1,1902.9)	34.96 (29.53,41.14)	4356.2 (3260.8,5497.1)	49.06 (36.92,61.05)	1.56 (1.38,1.74)

Myanmar	38174.7 (30153.2,48213.4)	193.33 (157.01,238.03)	56701 (46130.3,70566)	138.21 (113.02,171.61)	-1.25 (-1.34,-1.16)
Namibia	526.8 (426.3,624.6)	104.53 (84.8,124.55)	1154.7 (890.6,1456.6)	104.17 (81.37,129.06)	-0.17 (-0.46,0.11)
Nauru	17 (13.4,20.7)	412.25 (337.73,489.41)	22.6 (18.2,28)	432.64 (361.02,517.42)	0.13 (-0.14,0.4)
Nepal	10694.4 (8418.6,13409.3)	130.11 (102.83,160.78)	27632.3 (22535,34206.3)	136.53 (111.67,169.87)	0.45 (0.28,0.62)
Netherlands	28810.3 (26524.6,30053.6)	141.63 (130.27,147.71)	14788.8 (12738.6,15964.4)	37.42 (32.5,40.31)	-4.82 (-5.04,-4.6)
New Zealand	7170.7 (6700.2,7477.1)	185.37 (172.37,193.64)	5608.5 (4818.2,6038.4)	60.98 (52.78,65.35)	-3.81 (-3.89,-3.72)
Nicaragua	1178.3 (1074,1266.4)	89.16 (80.6,96.17)	3410 (2907.1,3971.7)	81.93 (70.19,95.03)	0.06 (-0.16,0.28)
Niger	1418.7 (992.2,2031.7)	68.91 (49.35,98.38)	4512.5 (3147.7,6307.9)	72.56 (51.59,98.73)	0.24 (0.14,0.33)
Nigeria	38716.8 (30965.3,47886.4)	107.45 (85.67,131.35)	76296.5 (61775.8,91798.9)	109.48 (90.67,129.23)	0.14 (0.03,0.26)
Niue	5.6 (4.8,6.5)	241.28 (204.64,282.86)	5 (4.2,5.7)	243.51 (206.45,279.74)	-0.1 (-0.17,-0.03)
North Macedonia	3484.1 (3138.8,3870.2)	208.57 (187.07,232.69)	4658.5 (3973.1,5430.3)	195.86 (170.12,224.26)	-0.58 (-1.02,-0.13)
Northern Mariana Islands	19.9 (16.1,25.1)	134.08 (113.25,163.61)	65 (56.4,70.1)	152.09 (134.52,164.56)	0.67 (0.49,0.85)
Norway	12969.7 (11941.4,13482.4)	175.82 (162.81,182.41)	4858.8 (4125,5230.9)	40.88 (35.33,43.73)	-4.94 (-5.12,-4.77)
Oman	1745 (1357.7,2198.5)	301.16 (238.04,376.57)	2589.7 (2150.3,3128.6)	179.25 (150.33,211.7)	-1.4 (-1.53,-1.27)
Pakistan	71656.8 (57131.6,82011)	140.71 (111.59,162.54)	194034.5 (163122,238656.2)	183.45 (154.6,226.81)	0.81 (0.69,0.94)
Palau	21.2 (17.6,25.5)	249.87 (210.92,298.11)	42 (34.4,50.2)	233.62 (197.27,272.14)	-0.02 (-0.11,0.07)
Palestine	2107.5 (1728,2566.6)	288.99 (240.4,346.99)	3547.5 (3085.4,3981.2)	188.68 (162.69,211.7)	-1.51 (-1.77,-1.25)
Panama	1437.6 (1338.5,1509)	105.33 (97.33,110.85)	2496.4 (1955.9,2981.6)	54.48 (43.03,64.93)	-1.97 (-2.21,-1.72)
Papua New Guinea	2129.4 (1579.4,2848.6)	136.52 (103.02,179.07)	6346.2 (4746.6,8242)	144.45 (109.76,186.31)	0.26 (0.14,0.37)
Paraguay	2238.3 (1974.1,2516.2)	109.31 (95.72,122.85)	4749.1 (3675.8,5880.7)	86.53 (67.26,106.48)	-0.43 (-0.67,-0.19)
Peru	8732.4 (7614.8,9911.8)	79.05 (69.19,89.61)	15109.6 (11733.6,18833.8)	44.8 (34.86,55.87)	-2.25 (-2.73,-1.77)
Philippines	39311.4 (35601.4,42450.9)	174.14 (159.02,186.45)	107668.5 (91777.8,124077.2)	150.42 (129.19,171.93)	-0.35 (-0.43,-0.28)
Poland	109992.2 (105216.9,112660.5)	269.75 (255.72,277)	88241.7 (78193.4,95579.6)	113.26 (100.87,122.58)	-3.08 (-3.22,-2.94)
Portugal	14675.2 (13824.8,15186)	116.87 (109.19,121.4)	10702.4 (9140.1,11542.5)	36.8 (32.35,39.41)	-4.22 (-4.47,-3.98)
Puerto Rico	5438.8 (5176.5,5627.9)	161.56 (152.58,167.32)	4505.4 (3696.3,5205.4)	53.62 (44.64,61.73)	-3.84 (-4.07,-3.61)
Qatar	257.2 (218.7,298)	384.95 (334.48,437.71)	652.9 (498.5,825.9)	123.16 (97.95,147.94)	-4.51 (-5.27,-3.75)
Republic of Korea	12338.7 (10729.6,14137.6)	60.15 (51.83,68.91)	25415.4 (20651.6,29168.9)	28.27 (22.87,32.57)	-3.03 (-3.3,-2.75)
Republic of Moldova	13477.6 (12962.4,13865.3)	404.17 (382.51,417.02)	13930.2 (12632.3,15262.3)	230.52 (209.69,252.49)	-2.52 (-2.83,-2.21)
Romania	62748.5 (60268.2,64675.2)	278.03 (264.18,287.37)	62394.4 (56688.8,68403.8)	154.19 (140.01,169.13)	-2.45 (-2.63,-2.27)
Russian Federation	482850.7 (462864.9,492436.8)	311.27 (294.21,318.67)	512524.3 (470911.6,551238)	212.91 (195.61,229.02)	-1.61 (-2.1,-1.12)
Rwanda	2288.1 (1669.3,3038.3)	97.17 (72.2,127.11)	3191.4 (2246.1,4320.3)	65.09 (46.31,88.1)	-2.22 (-2.59,-1.86)
Saint Kitts and Nevis	79.2 (74.9,83.5)	220.62 (208.45,231.84)	50.2 (42.4,58.8)	91.15 (78.72,103.5)	-2.76 (-2.99,-2.54)

Saint Lucia	108 (103.2,112.4)	150.23 (143.53,156.46)	111.2 (92.3,130.3)	48.5 (40.2,56.66)	-4.25 (-4.67,-3.83)
Saint Vincent and the Grenadines	114.7 (107.5,121.6)	176.86 (165.16,187.43)	126.6 (113.7,140.5)	100.97 (90.71,111.62)	-2.03 (-2.28,-1.79)
Samoa	149.9 (124.3,175)	205.73 (170.84,240.01)	289.7 (247.5,349.9)	226.72 (194.95,270.93)	0.37 (0.33,0.41)
San Marino	28.7 (24.5,32.8)	76.92 (65.7,87.83)	23.6 (16.2,32)	23.19 (15.63,32)	-3.28 (-3.64,-2.92)
Sao Tome and Principe	45.8 (39.9,51.6)	80.51 (70.58,90.2)	92.2 (78.9,107.8)	103.54 (89.77,120.61)	0.99 (0.86,1.12)
Saudi Arabia	11469.2 (8755.1,14415.8)	225.52 (174.28,279.62)	32329.7 (26303.9,39773.8)	185.9 (158.13,219.08)	-0.72 (-0.91,-0.52)
Senegal	3469.5 (2800.6,4100.5)	128.22 (105.19,151.19)	7718.3 (6034.5,9735.6)	121.62 (95.49,151.29)	-0.39 (-0.49,-0.3)
Serbia	25355.2 (23283.9,27309.7)	309.12 (280.44,334.36)	29978.9 (25783.2,34468.2)	174.28 (150.25,200.22)	-2.56 (-2.84,-2.27)
Seychelles	75.1 (69.1,81)	134.13 (123.05,144.58)	90.1 (80.7,101)	87.6 (78.75,97.97)	-1.27 (-1.4,-1.15)
Sierra Leone	2307.2 (1920.5,2761.6)	128.79 (108.47,151.69)	4056.1 (3115,5076.1)	129.53 (101.84,159.08)	0.2 (0.01,0.39)
Singapore	2926.7 (2803.7,3011)	148.22 (140.14,153.04)	3896 (3497.4,4151.3)	46.74 (41.78,49.85)	-3.96 (-4.2,-3.72)
Slovakia	18672.7 (17790.4,19459.8)	327.25 (310.38,341.42)	17504.1 (15199.4,19596.6)	184.77 (160.51,206.7)	-1.77 (-1.84,-1.7)
Slovenia	3107.6 (2900.9,3271.5)	128.3 (119.03,135.28)	2449.1 (2068.5,2749.4)	45.95 (39.17,51.61)	-3.85 (-4.04,-3.65)
Solomon Islands	325.4 (233.9,416.4)	292.82 (239.31,353.68)	806.6 (646.5,1027.5)	275.17 (228.47,339.14)	-0.2 (-0.32,-0.08)
Somalia	1295.7 (921.7,1839.8)	66.17 (49.89,90.31)	3416.8 (2293.7,4851.4)	68.48 (48.16,95.21)	0.2 (0.07,0.34)
South Africa	13911.3 (11672.4,15652.4)	74.43 (61.29,84.33)	30301 (27568.3,32899.9)	78.04 (71.05,84.66)	0 (-0.39,0.39)
South Sudan	1629.9 (1237.2,2145.2)	72.37 (55.04,93.38)	2605.2 (1896.5,3515.4)	81.22 (61.25,106.99)	0.27 (0.2,0.34)
Spain	52067.1 (47772.9,54510.2)	98.17 (89.76,102.96)	43081.8 (36292.9,46992.7)	35.37 (30.69,38.04)	-3.48 (-3.6,-3.35)
Sri Lanka	12405.8 (11273.2,13577.1)	138.44 (124.98,151.47)	23290.6 (15803,31233.8)	94.59 (64.82,125.44)	-0.81 (-1.02,-0.59)
Sudan	27788.4 (22309.4,34470)	339.57 (276.21,415.93)	42892 (33157.3,56641)	255.88 (202.4,330.73)	-1.08 (-1.14,-1.02)
Suriname	410.4 (376.6,439.5)	172.96 (158.64,185.24)	552.8 (426.5,689.9)	90.94 (69.95,113.45)	-1.91 (-2.19,-1.63)
Sweden	30305.2 (27733.6,31600.7)	181.58 (166.78,189.16)	14043.6 (11832.9,15648.1)	51.8 (44.14,57.63)	-4 (-4.1,-3.9)
Switzerland	15835.9 (14489,16626.4)	141.46 (129.9,148.32)	9513.3 (7740.7,10459.6)	39.96 (33.2,43.53)	-4.24 (-4.33,-4.15)
Syrian Arab Republic	17123.2 (14133.2,20368)	370.8 (309.81,437.57)	35508.2 (27709.3,45179.1)	353.01 (281.64,432.02)	-0.47 (-0.58,-0.36)
Taiwan (Province of China)	8718.6 (8258.9,9028.2)	73.72 (68.6,76.86)	14776.9 (13068.1,15986.4)	33.61 (30,36.21)	-2.46 (-2.71,-2.21)
Tajikistan	7406.4 (6481.8,8164.3)	304.83 (266.01,336.17)	10078 (8551.7,11707.2)	244.92 (206.56,281.62)	-0.97 (-1.31,-0.63)
Thailand	24292.6 (20886.2,28176.6)	84.42 (71.21,97.77)	50625.3 (39490.4,62945.7)	47.05 (36.8,58.63)	-2.54 (-2.8,-2.27)
Timor-Leste	273.2 (210.8,339.5)	122.8 (98.09,150.93)	1123.8 (857.8,1416.3)	156.95 (121.11,195.97)	1.12 (0.93,1.32)
Togo	1115.3 (916.5,1333.7)	114.24 (95.44,135.97)	3391.8 (2494,4413.9)	117 (89.49,149.18)	-0.17 (-0.38,0.04)
Tokelau	2.8 (2.3,3.3)	225.76 (189.4,267.48)	3 (2.4,3.6)	205.41 (164.75,248.55)	-0.31 (-0.39,-0.24)

Tonga	67.9 (56.8,79)	138.69 (116.31,161.28)	107.5 (87.5,129.5)	139.13 (113.6,166.57)	0.15 (0.03,0.28)
Trinidad and Tobago	1674.3 (1615.2,1724.8)	234.72 (225.53,241.92)	1981.8 (1549.8,2471.8)	105.96 (83.2,131.89)	-3.12 (-3.37,-2.86)
Tunisia	8615.4 (7169.9,9916.3)	218.26 (180.41,251.41)	19263.3 (14373.7,25409.2)	163.59 (121.84,215.67)	-1.24 (-1.37,-1.12)
Turkmenistan	6352.7 (6046,6608.8)	407.01 (383.66,423.46)	11242.1 (9063.7,13846.3)	343.68 (280.94,420)	-1.29 (-1.59,-0.98)
Tuvalu	15.6 (13.3,18)	274.89 (236.08,314.05)	24.6 (21.1,28.9)	269.54 (234.98,314.27)	-1.5 (-1.9,-1.11)
Timor-Leste	61298.3 (54354.7,68192.7)	204.65 (180.71,228.18)	112054.6 (92076.8,131673.9)	133.38 (109.89,155.94)	0.01 (-0.04,0.06)
Uganda	3788.4 (2919.5,4763.8)	70.72 (54.09,87.52)	8689.4 (6836.9,11117.2)	71.03 (56.38,91.34)	-0.61 (-1,-0.23)
Ukraine	214996.6 (203977.8,221903.9)	338.06 (318.09,349.5)	297502.6 (232625.5,365310.6)	373.47 (291.58,459.07)	-0.3 (-0.82,0.23)
United Arab Emirates	904.5 (721,1127.4)	266.92 (214.61,326.84)	2885.2 (2296.3,3515.4)	167.71 (136.64,196.33)	0.13 (-0.41,0.67)
United Kingdom	184357.7 (173164.2,189356.8)	196.49 (184.68,201.92)	76354.2 (67535.9,80433.7)	52.11 (46.86,54.65)	-4.67 (-4.85,-4.49)
United Republic of Tanzania	6248.6 (4952.8,7716.4)	66.88 (52.4,83.81)	21192 (15715.1,26987.3)	96.28 (72.01,123.39)	1.04 (0.92,1.17)
United States of America	594729.1 (534528.6,623663.9)	179.83 (162.05,188.39)	493222 (432450.6,527138.6)	78.92 (69.93,83.85)	-3.01 (-3.15,-2.86)
United States Virgin Islands	140 (121.4,159.1)	213.35 (187.38,239.88)	174.2 (142.3,214.4)	102.62 (83.61,125.67)	-2.25 (-2.39,-2.11)
Uruguay	5852.8 (5529.8,6052.1)	152.28 (143.29,157.84)	3798.2 (3450.2,4019.7)	60.55 (55.5,63.77)	-3.04 (-3.23,-2.85)
Uzbekistan	33180.8 (30938,34667.2)	318.62 (295.07,333.63)	67525.7 (58786.8,76515.1)	339.48 (295.64,382.01)	0.3 (-0.05,0.65)
Vanuatu	165.6 (134.4,204.8)	312.35 (259.57,376.09)	460.2 (385.6,539.6)	308.39 (260.86,357.06)	-0.1 (-0.14,-0.05)
Venezuela (Bolivarian Republic of)	14718.2 (13944.4,15192.2)	169.56 (157.91,175.88)	40259.6 (31729.4,50646)	141.4 (112.21,177.15)	-0.98 (-1.26,-0.7)
Viet Nam	23536.9 (18689.1,29917.1)	66.4 (52.9,83.93)	64414.8 (52827.7,76110.8)	76.99 (63.45,90.68)	0.96 (0.77,1.16)
Yemen	13200.8 (10073,16958.2)	320.35 (252.11,407.07)	30562.2 (22982.5,39980.6)	263.54 (201.84,344.23)	-0.85 (-0.94,-0.75)
Zambia	1444.6 (1157.5,1792.7)	62.2 (49.58,76.43)	5166.1 (3898.3,6593.1)	92.7 (71.66,117.34)	1.29 (1.18,1.4)
Zimbabwe	2460.5 (2021.4,2888.6)	83.54 (68.17,98.94)	6160.7 (4920.2,7788.4)	118.54 (97.5,145.32)	1.75 (1.21,2.29)

Table S4 The disability-adjusted life years (DALYs) of ischemic heart disease cases and age-standardized rate at national level in 1990 and 2021, with EAPC (1990-2021).

Location	1990		2021		EAPC (95% CI)
	Number	ASR	Number	ASR	
Afghanistan	586599.9 (428843.8,738060.6)	8374.25 (6234.26,10414.15)	685744.7 (510801.3,895880.7)	6178.27 (4747.55,7813.31)	-1.2 (-1.31,-1.09)
Albania	61286.7 (55127.9,66796.5)	3173.37 (2843.65,3463.96)	107500.6 (91802.5,125962)	2558.27 (2184.88,2998.29)	-0.34 (-0.54,-0.14)
Algeria	591558.5 (503229.4,700253.8)	5491.83 (4703.56,6400.44)	1081869 (873889.5,1320137.5)	3451.99 (2806.95,4159.87)	-1.68 (-1.77,-1.59)
American Samoa	924.6 (829.1,1034.5)	3787.63 (3414.57,4166.2)	1966.2 (1663.5,2319.3)	4053.34 (3456.83,4752.36)	0.34 (0.27,0.41)
Andorra	852.4 (637.1,1134)	1555.69 (1177.61,2044.18)	1169.2 (876.7,1498.9)	724.42 (539.1,929.5)	-2.28 (-2.51,-2.05)
Angola	104142.3 (81520.9,131398.5)	2723.29 (2157.98,3350.73)	287317.8 (216394.7,366758.1)	2529.04 (1954.37,3169.14)	-0.44 (-0.52,-0.36)
Antigua and Barbuda	1359.7 (1275.4,1437.4)	2532.24 (2376.33,2673.08)	1334.1 (1247.9,1479.9)	1306.35 (1226.11,1441.33)	-2.49 (-2.69,-2.28)
Argentina	951849.7 (921724.7,978424.6)	3045.51 (2935.24,3135.27)	659381.1 (620826.6,690368.8)	1179.87 (1113.64,1234.14)	-2.74 (-2.9,-2.59)
Armenia	144959.6 (137829.4,151754.2)	5669.83 (5377.99,5942.52)	162012.6 (145598.4,180960.5)	3788.46 (3403.75,4233.65)	-1.96 (-2.15,-1.76)
Australia	612460.8 (578633.8,632274.5)	3168.21 (2983.34,3273.46)	356731.8 (318531.2,379798.2)	768.62 (696.1,814.23)	-4.78 (-4.92,-4.64)
Austria	354106.1 (334203.1,365396.7)	2995.04 (2831.23,3084.75)	222211 (197214.1,236453.8)	1121.64 (1017.81,1189.7)	-3.54 (-3.76,-3.33)
Azerbaijan	344740.3 (323377.7,367847.5)	7154.02 (6698.23,7635.33)	502771.1 (436012.6,570143.4)	5496.28 (4818.68,6182.32)	-0.95 (-1.17,-0.74)
Bahamas	4666 (4330.4,5011.7)	2923.91 (2718.72,3130.71)	6647.5 (5432,8163.4)	1624.95 (1339.72,1979.27)	-1.97 (-2.15,-1.78)
Bahrain	13082.4 (12255.4,13964)	7687.42 (7231.46,8166.72)	22926.9 (19535.6,26636.9)	2796.56 (2418.62,3179.47)	-3.98 (-4.31,-3.64)
Bangladesh	1546188.3 (1338136.6,1793572.6)	3001.81 (2602.02,3493.06)	3299090.8 (2567550.9,4098891.6)	2366.92 (1856.38,2929.73)	-0.55 (-0.63,-0.46)
Barbados	6417.8 (6072.3,6706)	2226.37 (2109.73,2327.01)	5908.7 (4794.4,7123.3)	1150.8 (936.24,1387.58)	-2.32 (-2.61,-2.03)
Belarus	757469.2 (720128.8,784279.4)	6062.14 (5746.82,6278.97)	999252.4 (842559.5,1164446.9)	6244.29 (5262.84,7270.21)	-0.42 (-0.83,0)
Belgium	378518.7 (358185.2,394018.5)	2492.88 (2362.68,2592.45)	169751.6 (152365.9,181890.8)	698.85 (638.41,740.15)	-4.17 (-4.3,-4.05)
Belize	2338 (2206.8,2472.7)	2473.96 (2332.73,2612.99)	4071.8 (3640.7,4518.8)	1363.17 (1223.34,1513.59)	-2.51 (-2.84,-2.18)
Benin	28819.6 (24174,33814)	1479.74 (1249.32,1734.07)	75681.9 (60918.7,93724.3)	1514.95 (1244.52,1848.81)	0.12 (-0.06,0.3)
Bermuda	2838.5 (2693.3,2980.3)	4662.76 (4423.45,4892.18)	1768 (1543.1,2098.2)	1283.78 (1113.11,1520.67)	-4.12 (-4.49,-3.76)
Bhutan	6664.5 (4797.6,8711.6)	2498.31 (1850.22,3203.73)	14097.5 (10698.4,17814.6)	2299.54 (1757.99,2871.27)	-0.25 (-0.28,-0.23)
Bolivia (Plurinational State of)	91096.9 (70248.6,121540.8)	2859.68 (2235.98,3774.83)	139905.3 (99244.1,200269.5)	1602.36 (1154.23,2291.24)	-1.86 (-2.1,-1.61)
Bosnia and Herzegovina	154131 (142254.3,165743.8)	3942.91 (3620.83,4253.65)	152300.2 (125722.2,177531.7)	2450.2 (2019.97,2859.4)	-1.95 (-2.2,-1.7)
Botswana	11196.2 (8356.2,14260.8)	2087.17 (1574.29,2634.47)	22404.4 (16809.7,28919.9)	1574.31 (1216.62,1984.6)	-1.06 (-1.36,-0.76)
Brazil	2626269.6 (2536094.8,2689422.8)	2916.95 (2791.89,2996.12)	3713582.8 (3497727.2,3865675.7)	1469.72 (1380.14,1530.27)	-2.16 (-2.23,-2.09)
Brunei Darussalam	3649.2 (3247.9,4138.1)	3122.14 (2784.54,3516.89)	6297.5 (5561.8,7068.5)	1726.32 (1525.62,1929.94)	-1.69 (-1.92,-1.47)
Bulgaria	693660 (664150.7,722094.5)	6709.57 (6446.84,6955.89)	513391.5 (445089.4,587042.9)	3852.25 (3333.94,4409.66)	-2.51 (-2.82,-2.19)

Burkina Faso	69315.9 (57069.2,85283.5)	1684.46 (1387.04,2066.69)	164843.7 (124913.6,214923.2)	1884.27 (1448.29,2432.51)	0.59 (0.48,0.7)
Burundi	56919.8 (42343.3,72775.8)	2361.94 (1784.77,2975.77)	95297.5 (74253.8,122241.1)	1841.44 (1452.49,2345.53)	-1.34 (-1.55,-1.14)
Cabo Verde	3371.9 (2801.7,3912.6)	1462.65 (1215.87,1697.92)	9734.3 (7915.8,11664.5)	2236.65 (1830.13,2642.4)	0.83 (0.5,1.17)
Cambodia	122894.8 (104140.1,146210.1)	2609.86 (2214.27,3068.74)	271595.8 (210702.4,344178.2)	2251.18 (1767.77,2790.06)	-0.58 (-0.66,-0.5)
Cameroon	67019.8 (51266.6,87232.1)	1587.48 (1234.71,2069.48)	249947.8 (184489.9,342517.2)	2044.68 (1551.58,2747.31)	0.93 (0.35,1.51)
Canada	929566.8 (881760.2,956834.5)	2886.93 (2735.29,2972.96)	673922.8 (612998.9,710472.3)	922.52 (849.26,968.23)	-3.88 (-4.03,-3.74)
Central African Republic	41413.1 (30993.8,58197.1)	3637.89 (2796.17,4979.81)	73539.5 (51644.8,107584.8)	3256.9 (2364.11,4604.4)	-0.48 (-0.57,-0.4)
Chad	49118.7 (39525.1,62213.8)	1784.09 (1432.24,2259.3)	120806.4 (89854.4,156835.8)	2128.18 (1619.46,2717.98)	0.47 (0.24,0.71)
Chile	200484.9 (192999.9,207110.1)	2103.45 (2010.67,2173.9)	205559.7 (193087.4,215569.4)	808.36 (760.79,847.46)	-2.9 (-3.01,-2.8)
China	13624111.9 (12056605.6,15466092.4)	1771.13 (1574.76,1990.67)	35672627 (29920272.7,41738945.8)	1856.51 (1548.73,2159.82)	0.51 (0.25,0.77)
Colombia	514566 (495169,527024.3)	2923.19 (2794.48,3003.12)	836593.4 (704338.1,977242.1)	1502.23 (1264.42,1754.08)	-2.38 (-2.58,-2.17)
Comoros	3386.2 (2456,4486.3)	1689.28 (1272.97,2206.67)	7455.1 (5445.2,9853.7)	1526.07 (1137.18,1995.75)	-0.64 (-0.79,-0.48)
Congo	41564.2 (32140.5,52427.7)	3978.97 (3127.41,4943.57)	84768.2 (64182,108163.7)	3210.45 (2515.58,3995.7)	-1.03 (-1.18,-0.87)
Cook Islands	454.3 (385.8,530.9)	3569.21 (3067.02,4118.54)	578.6 (480.5,692.3)	2344.82 (1931.57,2837.91)	-1.23 (-1.38,-1.07)
Costa Rica	41061.1 (38828,42981.6)	2365.63 (2232.46,2476.36)	64159.1 (57103.2,70862.8)	1157.79 (1033.42,1276.35)	-2.42 (-2.61,-2.24)
Croatia	272561.9 (260888.8,284574.4)	4878.65 (4658.97,5087.24)	193497.8 (171215.4,213646.6)	2099.58 (1856.5,2326.01)	-0.16 (-0.41,0.08)
Cuba	398553.1 (386154.2,407628.5)	3975.87 (3835.56,4068.48)	398498.4 (347563.4,445609.1)	2017.01 (1763.5,2255.67)	-2.76 (-2.85,-2.68)
Cyprus	26350.2 (23956.6,28792)	4121.86 (3765.18,4505.07)	26028.4 (22606.6,29450.5)	1394.96 (1225.32,1564.95)	-2.43 (-2.71,-2.14)
Czechia	788611.2 (763690.8,807511.3)	5796.5 (5603.59,5941.74)	464015.2 (409485.7,512872.5)	2101.42 (1857.11,2327.15)	-3.81 (-3.96,-3.66)
Cmte d'Ivoire	92459.6 (71541.7,116309.7)	2396.65 (1914.32,2923.62)	262037.7 (200518.5,351436.1)	2390.53 (1895.8,3151.33)	-3.16 (-3.27,-3.06)
Democratic People's Republic of Korea	346846.5 (255954.2,447464.6)	2253.96 (1711.64,2837.35)	822717.4 (649055.7,1024585.8)	2594.23 (2058.22,3211.81)	0.45 (0.27,0.63)
Democratic Republic of the Congo	401399.5 (297308.9,531113.8)	2692.38 (2015.08,3518.02)	812224.1 (594615.2,1095454.4)	2290.38 (1696.29,3056.52)	-0.67 (-0.77,-0.58)
Denmark	313538.1 (297979.5,323457)	3848.32 (3674.46,3962.64)	85462.7 (77569.3,91386.1)	710.23 (653.57,756)	-5.81 (-6,-5.63)
Djibouti	2144.4 (1501,2941.4)	1502.93 (1068.44,2009.51)	12618.7 (9005.9,16946.1)	1927.16 (1417.07,2555.52)	0.71 (0.56,0.87)
Dominica	1614.3 (1507.3,1720.5)	2764.02 (2582.61,2949.57)	1422.1 (1228.6,1695.1)	1738.9 (1513.77,2064.5)	-1.65 (-1.93,-1.37)
Dominican Republic	110997 (98582.2,124806)	2975.43 (2653.1,3344.42)	314140.2 (251927,387345.9)	3104.02 (2494.68,3824.78)	0.71 (0.54,0.88)
Ecuador	101191.6 (97710.5,104702.1)	1887.12 (1814.85,1949.64)	228672.8 (184006.6,284335)	1433.67 (1158.9,1770.44)	-1.02 (-1.53,-0.52)
Egypt	2062259 (1897203,2265103.6)	7600.04 (6957.35,8365.64)	4247263.9 (3552704.1,5000935.3)	6924.84 (5844.94,8119.21)	0.07 (-0.12,0.25)
El Salvador	76502.3 (71752.3,80895.5)	2481.2 (2315.72,2623.08)	121857 (100270.3,146378.4)	1915.93 (1577.46,2311.23)	-0.93 (-1.18,-0.68)

Equatorial Guinea	6602.9 (4913.2,8604.3)	3452.66 (2636.55,4390.44)	13574.8 (8957.7,19460.8)	2751.44 (1907.88,3773.97)	-0.99 (-1.24,-0.74)
Eritrea	23839.8 (17507.2,31293.6)	1922.98 (1416.26,2478.61)	58749.8 (41914.9,78553.1)	1974.98 (1470.4,2570.23)	0.06 (-0.01,0.13)
Estonia	130593.5 (125054.4,135557.6)	6565.71 (6260.21,6824.17)	44766.9 (39784.4,49727.3)	1547.87 (1370.4,1716.96)	-5.13 (-5.54,-4.73)
Eswatini	5249.4 (4065.4,6513.6)	1874.14 (1479.93,2334.06)	13426.3 (8924.2,19789.1)	2334.11 (1612.79,3342.38)	1.21 (0.6,1.82)
Ethiopia	408846.4 (340209.1,536011.6)	1912.96 (1606.09,2437.13)	538380.7 (437967.5,652114)	1177.38 (959.27,1410.32)	-1.91 (-2.04,-1.78)
Fiji	28786.3 (25060.1,33470.6)	7013.52 (6118.23,8083.68)	46802.8 (36460.8,58905.7)	5965.61 (4721.09,7391.81)	-0.64 (-0.75,-0.53)
Finland	297809.2 (282904.9,306673.7)	4210.69 (4000.28,4341.2)	176500.1 (154931.3,189402.9)	1299.12 (1163.23,1383.11)	-3.77 (-3.81,-3.73)
France	1265075.5 (1190602.9,1315058.8)	1519.29 (1434.08,1575.77)	841382.1 (745327.5,909860)	555.98 (503.45,597.26)	-3.36 (-3.46,-3.26)
Gabon	15263.3 (12092.7,18251.8)	2759.66 (2206.7,3292.84)	24694.8 (18136.2,32123.9)	2479.16 (1901.2,3118.09)	-0.46 (-0.58,-0.34)
Gambia	7734.6 (5914.3,9914.3)	2275.42 (1782.53,2891.12)	26630.6 (20123.6,33958.8)	2771.5 (2116.36,3471.6)	0.5 (0.33,0.67)
Georgia	411934.7 (393859.7,428835.8)	6871.09 (6545.28,7157.65)	150074.7 (134549.6,165128.4)	2565.24 (2301.9,2822.2)	-4.04 (-4.5,-3.58)
Germany	4427915.2 (4180700.2,4575610.7)	3470.96 (3277.21,3583.26)	2250722.1 (1988200.1,2398480.2)	1096.63 (993.52,1160.04)	-3.86 (-3.97,-3.74)
Ghana	182767 (147997.2,220494.9)	2961.76 (2438.98,3533.87)	319862.1 (252764.4,397101.5)	1954.33 (1558.46,2392.96)	-1.84 (-2.17,-1.51)
Greece	387615.5 (368910.8,399090.7)	2638.91 (2507.67,2717.33)	336576.8 (305960.1,353353.8)	1443.35 (1353.57,1503.27)	-2.09 (-2.32,-1.86)
Greenland	1447 (1319.4,1583.5)	4291.52 (3946.23,4661.88)	1108.1 (970.3,1303.3)	1632.37 (1432.87,1918.7)	-3.18 (-3.34,-3.03)
Grenada	2255.8 (2073,2462.7)	3226.98 (2951.41,3535.89)	2159.9 (1880.4,2429.3)	1924.27 (1678.96,2155)	-1.98 (-2.26,-1.7)
Guam	3007.1 (2752.9,3245.5)	4016.36 (3720.18,4291.82)	6968.8 (6333.1,7666.9)	3413.08 (3110.68,3756.02)	0.12 (-0.13,0.36)
Guatemala	96931.3 (94022.3,99521.6)	2841.8 (2743.73,2923.7)	184133 (159805.7,208705.5)	1732.06 (1517.52,1959.37)	-1.67 (-2.17,-1.18)
Guinea	59910 (48290.8,73835)	1856.51 (1494.13,2273.42)	122141.5 (91782.9,160016.1)	2209.38 (1690.78,2864.84)	0.82 (0.68,0.96)
Guinea-Bissau	13037.2 (9976.1,16736.5)	3282.8 (2562.9,4162.43)	23941.7 (18052.8,30348)	3270.22 (2536.54,4074.07)	0.04 (-0.05,0.14)
Guyana	19737.1 (17679.1,21764.2)	5067.09 (4569.72,5581.42)	19878 (15444,25305)	3075.23 (2425.76,3871.77)	-1.35 (-1.57,-1.14)
Haiti	179458 (149750.2,213577.5)	5615.9 (4723.23,6606.57)	314130.6 (231575.3,414030.9)	4370.88 (3271.69,5735.77)	-0.62 (-0.72,-0.53)
Honduras	45477 (39837.7,51823.7)	2249.57 (1970.79,2543.48)	182157.1 (149789.7,225383.8)	3075.05 (2550.8,3764.35)	1.23 (1.05,1.41)
Hungary	738428.1 (715625.3,758296.9)	5265.28 (5095.96,5407.83)	551287.4 (491803.2,605306.1)	2780.98 (2470.6,3060.36)	-2.2 (-2.32,-2.08)
Iceland	8819.4 (8191.5,9247.5)	3048.18 (2840.57,3193.58)	5993.7 (5243.8,6585.4)	990.46 (883.5,1083.79)	-3.73 (-3.79,-3.67)
India	16765036.3 (15156253.2,18285253.4)	3321.51 (2980.3,3628.9)	41316678.4 (37458752.6,45305507.3)	3400.03 (3098.47,3720.83)	0.18 (0.06,0.3)
Indonesia	2602187.4 (2270109.5,2942989.6)	2420.31 (2079.08,2751)	7347522.3 (6101893.3,8695819.8)	3043.08 (2527.28,3544.71)	0.87 (0.78,0.96)
Iran (Islamic Republic of)	1298775.5 (1219449.2,1376314)	5166.82 (4824.54,5465.59)	2059627.6 (1916237.3,2206642)	2731.3 (2517.27,2920.87)	-2.26 (-2.4,-2.12)
Iraq	447958.2 (382854.7,529818.2)	5521.97 (4733.05,6492.42)	1119623.5 (867514.9,1361252.2)	4905.27 (3858.32,5827.68)	-0.84 (-0.98,-0.7)
Ireland	172782 (165640.2,177832.9)	4289.56 (4107.68,4417.6)	73901.8 (65733.6,79376.4)	924.25 (829.87,991.72)	-5.04 (-5.21,-4.87)
Israel	150824.7 (143059.4,156194)	3189.07 (3014.78,3306.5)	76882.8 (68431.7,82058.6)	599.57 (537.77,637.8)	-5.8 (-6.03,-5.57)

Italy	1730943.9 (1627560,1788916)	1988.69 (1868.54,2056.66)	1194535.5 (1035883.3,1282896)	747.14 (673.73,791.65)	-3.32 (-3.4,-3.25)
Jamaica	24385 (23144.1,25479.5)	1356.97 (1290.03,1416.99)	33490.7 (26581.3,42589.6)	1054.54 (837.96,1345.47)	-0.51 (-0.97,-0.04)
Japan	1848886.1 (1727631.3,1913031.1)	1141.16 (1058.45,1184.99)	1803043 (1556452.9,1940530.4)	502.25 (460.02,526.29)	-2.51 (-2.65,-2.37)
Jordan	59478.1 (51886.4,68580.9)	4227.89 (3639.15,4842.1)	145876.2 (119698,179501.2)	1947.22 (1603.41,2349.61)	-3.09 (-3.4,-2.78)
Kazakhstan	709907.6 (658940.8,764377.6)	5827.04 (5426.21,6249.33)	628636.1 (555928.6,699123.8)	3970.5 (3569.75,4401.12)	-2.5 (-3.14,-1.84)
Kenya	67329.3 (50193.5,82276)	826 (614.45,1008.68)	281468.5 (217788.1,360825.4)	1244.93 (964.97,1595.88)	1.62 (1.3,1.94)
Kiribati	1937.6 (1589.2,2295.8)	4765.11 (3903.07,5656.94)	4038.6 (3239.8,5040)	4969.44 (4015.48,6139.26)	0.17 (0.12,0.21)
Kuwait	30516.4 (28370.4,32778.5)	4299.29 (3968.73,4614.25)	79997.1 (66367.6,96588.5)	2299.44 (1917.04,2762.48)	-1.95 (-2.37,-1.53)
Kyrgyzstan	147452.2 (135527.2,159085.2)	5149.84 (4726.51,5557.72)	205476.3 (175972.8,239144.5)	4780.48 (4093.58,5490.56)	-0.23 (-0.63,0.17)
Lao People's Democratic Republic	119024.1 (93329.8,149506)	5469.16 (4337.25,6801.47)	173830.9 (133708.1,222410.4)	3667.08 (2896.8,4573.18)	-1.44 (-1.54,-1.34)
Latvia	225093.8 (215114.5,233207.4)	6428.29 (6135.91,6666.4)	118805.5 (105561.3,131124.8)	2916.55 (2581.61,3230.55)	-3.07 (-3.43,-2.71)
Lebanon	105363.4 (87218.3,128974.4)	4913.43 (4089.92,6007.82)	107889.9 (92699.5,125657.4)	1735.3 (1488.38,2027.36)	-3.37 (-3.72,-3.01)
Lesotho	8076.1 (6312.3,10440.8)	1003.15 (787.27,1289.48)	20704 (13400,32850.6)	1949.42 (1307.36,2989.62)	3.06 (2.5,3.63)
Liberia	23761.4 (19424.8,28673.1)	2135.72 (1785.33,2543.41)	47493.2 (35856,63559.7)	2264 (1760.67,2953.76)	0.16 (0.03,0.29)
Libya	69066 (55920.2,84425.1)	3439.47 (2793.25,4231.43)	207948 (160413.5,267731.1)	3765.46 (2952.85,4839.08)	0.68 (0.46,0.89)
Lithuania	288281.8 (275258.3,298080.2)	6508.98 (6200.62,6730.6)	202513.1 (181819.7,222723.9)	3332.3 (3000.98,3676.82)	-2.2 (-2.48,-1.92)
Luxembourg	15077.5 (14362.6,15682.5)	2820.28 (2685.42,2933.74)	8778.3 (7931.7,9631.9)	789.76 (717.69,867.8)	-4.29 (-4.46,-4.13)
Madagascar	100811 (86791.3,117150.7)	1884.99 (1614.8,2175.89)	270937.9 (194927.7,351706.6)	2205.88 (1634.85,2810.29)	0.17 (0.03,0.31)
Malawi	60967.6 (51754.8,71240.1)	1531.94 (1311.86,1794.42)	149417.6 (121750.9,178679.7)	1869.9 (1556.4,2221.41)	0.37 (0.07,0.67)
Malaysia	364444.8 (340534.7,387913.9)	3880.69 (3601.16,4129.31)	901888.2 (844939.9,952013.7)	3180.36 (2984.07,3352.24)	-0.68 (-0.79,-0.56)
Maldives	3654.5 (3207.2,4222.9)	3820.03 (3377.17,4272.58)	5344 (4363.8,6361.9)	1481.02 (1238.38,1759.98)	-3.5 (-3.66,-3.35)
Mali	58522.5 (44091.2,75582.3)	1536.56 (1173.34,1971.96)	124016.6 (95312.5,161037.8)	1439.78 (1125.23,1851.95)	-0.12 (-0.22,-0.01)
Malta	15881.7 (15039.2,16628.1)	3799.45 (3582.49,3983.42)	12471.7 (10997.6,13680)	1270.51 (1140.12,1387.85)	-3.53 (-3.69,-3.37)
Marshall Islands	1082.9 (935.7,1264.2)	6177.87 (5401.36,7130.04)	2557.7 (1948.2,3292.9)	6518.18 (5093.82,8205.42)	0.26 (0.13,0.38)
Mauritania	26818.6 (21117.8,33424.2)	2760.82 (2194.02,3443.03)	43800.3 (32045.2,56811.8)	2126.35 (1570.63,2744.37)	-1.16 (-1.34,-0.97)
Mauritius	36358.3 (34520.3,37984.1)	4922.82 (4673.85,5153.62)	36457.2 (34119,38029.3)	2089.12 (1952.72,2175.06)	-3.19 (-3.46,-2.91)
Mexico	832032.2 (811517,845792.8)	2050.57 (1986.67,2089.13)	2626717 (2336523.2,2939559.1)	2125.69 (1892.12,2373.05)	-0.03 (-0.29,0.22)
Micronesia (Federated States of)	3262.8 (2613.4,4024.8)	6402.7 (5174.68,7820.23)	4980.7 (3847.5,6453.6)	6279.72 (4925.69,8031.96)	0 (-0.03,0.04)
Monaco	1359.3 (1089.6,1591.5)	1950.7 (1568.93,2295.83)	893 (732.2,1063.9)	900.15 (731.12,1090.66)	-2.59 (-2.67,-2.51)
Mongolia	59880.9 (52039,68745.5)	6067.42 (5273.21,6935.77)	80740.7 (69458.8,92449)	3892.31 (3366.09,4387.1)	-1.89 (-2.17,-1.6)
Montenegro	20165.9 (18028.9,22396)	3277.13 (2917.87,3635.95)	32323.3 (28567.7,36553.8)	3538.19 (3127.13,3990.74)	0.29 (0.17,0.42)

Morocco	914680 (785021.3,1071093)	6313.77 (5422.21,7352.03)	1736791.9 (1312915.1,2080879.2)	5211.8 (3980.8,6188.35)	-0.69 (-0.73,-0.64)
Mozambique	41436.7 (35277.6,48183.1)	728.22 (624.85,851.01)	120882.5 (90973.2,154433.3)	1060.36 (811.04,1321.95)	1.71 (1.5,1.93)
Myanmar	1052296.1 (815544.9,1358341.7)	4367.91 (3444.62,5574.92)	1313711.7 (1045166.3,1653587.4)	2773.11 (2241.08,3446.11)	-1.64 (-1.75,-1.53)
Namibia	13318.9 (10802.6,15819.1)	2153.65 (1752.17,2546.31)	28314.1 (21280.6,36855.4)	2109.1 (1621.51,2684.92)	-0.28 (-0.6,0.05)
Nauru	548.5 (423.4,679.1)	10274.81 (8155.06,12463.55)	709.6 (552.4,897.5)	10681.95 (8619.33,13238.75)	0.09 (-0.21,0.39)
Nepal	303941.3 (237964.3,386840.7)	3038.88 (2408.53,3805.54)	671411.7 (535506,840703.9)	2890.07 (2320.57,3596.09)	0.11 (-0.07,0.28)
Netherlands	545538.5 (515919.5,564434.3)	2753.53 (2609.59,2848.07)	229771.1 (204971.4,246269.8)	633.61 (573.46,675.33)	-5.26 (-5.47,-5.04)
New Zealand	139552 (133100,144518)	3586.87 (3420.28,3716.19)	89504.7 (80333.2,94838.3)	1048.58 (948.57,1108.69)	-4.23 (-4.37,-4.1)
Nicaragua	26844.3 (24852.3,28797.5)	1733.21 (1594.01,1853.9)	72206.1 (61212.7,85038.2)	1524.24 (1300.65,1784.47)	-0.19 (-0.38,-0.01)
Niger	35637.1 (24710.9,50538.5)	1374.05 (976.26,1963.22)	108963.4 (75198.1,151784.1)	1413.47 (999.77,1956.91)	0.15 (0.04,0.26)
Nigeria	873621.9 (690092.9,1095886.9)	2075.03 (1659.58,2581.75)	1748869.6 (1379467.7,2175594.2)	2031.49 (1637.96,2451.09)	-0.02 (-0.14,0.11)
Niue	117.2 (96.9,140.8)	5362.83 (4384.31,6481.75)	111.2 (91.2,131.8)	5269.82 (4331.65,6254.32)	-0.22 (-0.3,-0.14)
North Macedonia	77222.7 (70346.9,84989.7)	4233.5 (3840.99,4664.62)	89848.6 (75733.9,105218.8)	3156.91 (2698.2,3663.31)	-1.3 (-1.63,-0.96)
Northern Mariana Islands	678.8 (532.5,869.5)	3038.91 (2521.95,3741.51)	1774.7 (1543.7,1907.8)	3338.98 (2926.76,3574.47)	0.63 (0.46,0.81)
Norway	230281 (218146.2,237368.6)	3392.21 (3239.64,3487.9)	73994.4 (65857.1,78896.8)	698.31 (633.77,740.7)	-5.28 (-5.39,-5.17)
Oman	45287.1 (34886,57332.7)	6490.3 (5047.76,8203.01)	67717.7 (55409.1,82264.6)	3393.69 (2837.02,4065.11)	-1.87 (-2.05,-1.7)
Pakistan	1848893.5 (1483925,2109784.9)	3174.69 (2549.7,3615.89)	5364774.4 (4438905.7,6657382.3)	4069.01 (3417.01,4985.77)	0.72 (0.57,0.87)
Palau	580.4 (471.7,706.3)	5703.85 (4703.34,6887.85)	1167.9 (946.8,1415.3)	5232.97 (4330.71,6240.48)	-0.12 (-0.2,-0.03)
Palestine	46621.2 (37398.2,58153.5)	5535.87 (4495.57,6824.43)	83101.3 (71972.9,93812.5)	3458.69 (3014.4,3871.78)	-1.66 (-1.88,-1.44)
Panama	29041.7 (27439.1,30244.7)	1985.6 (1871.77,2070.86)	49071.7 (38936.1,58435.8)	1097.33 (870.2,1306.77)	-1.8 (-2.06,-1.54)
Papua New Guinea	65855.4 (47424.5,90419.2)	3290.13 (2466.62,4369.78)	193030.6 (142580.3,253487.7)	3369.29 (2518.53,4367.45)	0.14 (0.03,0.25)
Paraguay	49100.7 (43825.7,55487)	2211.39 (1974.71,2497.4)	104092.3 (81216.1,129757.9)	1786.15 (1391.01,2223.94)	-0.48 (-0.67,-0.29)
Peru	197338.1 (170546.9,224671.4)	1595.07 (1382.34,1816.21)	313202.6 (247475.6,390718.9)	916.07 (723.47,1141.69)	-2.16 (-2.65,-1.66)
Philippines	1090473 (992076.4,1178567.1)	3518.68 (3210.33,3790.33)	2841768.6 (2400535.9,3317750.3)	3326.47 (2831.63,3850.53)	-0.16 (-0.22,-0.1)
Poland	2331952.7 (2264226.3,2379216.1)	5481.81 (5302.92,5595.96)	1460151.4 (1321796.7,1573412.6)	1991.47 (1811.44,2148.09)	-3.53 (-3.7,-3.36)
Portugal	279158.3 (267469.3,287976.7)	2112.57 (2014.45,2179.85)	175356.1 (157728.9,187021)	720.75 (663.52,762.18)	-3.96 (-4.25,-3.67)
Puerto Rico	106274.3 (102138.7,109783)	3012.46 (2890.25,3114.46)	79009.9 (66060.6,91081.8)	1139.29 (955.18,1324.13)	-3.51 (-3.76,-3.25)
Qatar	7526 (6369.5,8773.6)	7030.8 (6060.81,8075.67)	20661.6 (15896.7,26147.4)	2139.49 (1690.41,2627.18)	-4.5 (-5.17,-3.83)
Republic of Korea	297271.1 (258621.9,336200.6)	1084.12 (948.59,1232.98)	424498.3 (362480.2,475146.8)	470.99 (402.59,526.79)	-3.26 (-3.51,-3.01)
Republic of Moldova	264212.1 (255293.3,272164.3)	6803.79 (6546.08,7009.05)	260083 (237953.3,284614.4)	4379.75 (4010.89,4794.82)	-2.05 (-2.39,-1.71)
Romania	1253718.7 (1215094.3,1291808.4)	4916.91 (4742.35,5071.42)	1057765.8 (959350.9,1158841.9)	2837.96 (2575.38,3113.27)	-2.45 (-2.68,-2.22)

Russian Federation	10020381.7 (9747362.6,10188133.5)	5901.48 (5703.59,6010.77)	9631666.3 (8865971.2,10363140.8)	4082.19 (3760.5,4389.11)	-1.75 (-2.32,-1.18)
Rwanda	63471.6 (46487.5,84656.9)	2169.15 (1591.18,2893.37)	81119 (56925.8,109919.1)	1306.89 (923.91,1754.34)	-2.69 (-3.08,-2.28)
Saint Kitts and Nevis	1576.3 (1491.3,1663.8)	4327.22 (4081.53,4569.5)	1123 (927.6,1357.6)	1718.25 (1451.68,2018.72)	-3.05 (-3.29,-2.81)
Saint Lucia	2133.4 (2030.7,2231.5)	2634.46 (2509.06,2751.09)	2183.1 (1823,2561.8)	922.07 (770.21,1080.92)	-3.83 (-4.2,-3.47)
Saint Vincent and the Grenadines	2340.7 (2191.3,2487.7)	3361.57 (3148.23,3565.74)	2468.8 (2206.1,2743.2)	1822.04 (1633.37,2020.96)	-2.2 (-2.41,-1.99)
Samoa	3804.1 (3104.9,4518.7)	4467.13 (3693.86,5231.18)	7320.5 (6096.6,9025.2)	4993.89 (4206.29,6087.24)	0.43 (0.39,0.48)
San Marino	474.4 (415.5,535.5)	1325.16 (1160.09,1494.13)	353.8 (248.6,477.7)	429.12 (298.92,591.84)	-3.14 (-3.44,-2.83)
Sao Tome and Principe	978 (839,1114.7)	1566.56 (1352.81,1774.85)	2168.1 (1804.7,2610.5)	2006.42 (1715.18,2352.64)	0.85 (0.68,1.03)
Saudi Arabia	308806.6 (233829.3,392612.5)	4888.39 (3749.14,6143.97)	1075688.9 (851631.3,1338612.9)	4219.77 (3530.63,5082.93)	-0.42 (-0.64,-0.21)
Senegal	81636.8 (65024.5,97894)	2595.3 (2097.39,3086.18)	174458.7 (134823.4,221615.4)	2346.78 (1836.03,2939.94)	-0.52 (-0.62,-0.42)
Serbia	499906.7 (461344.8,533902.1)	5206.68 (4785.54,5596.74)	491639.5 (427238.2,561466.8)	2952.02 (2558.27,3364.12)	-2.43 (-2.68,-2.19)
Seychelles	1710.4 (1583.3,1843.2)	3012.36 (2786.27,3250.43)	2077.1 (1868.6,2326.5)	1807.8 (1629.13,2020.44)	-1.57 (-1.7,-1.45)
Sierra Leone	51702.7 (41967.7,63416.5)	2584.45 (2118.8,3127.79)	97284.2 (72878.4,124912.5)	2597.78 (1984.64,3281.59)	0.24 (0.04,0.45)
Singapore	71500.3 (69431.5,73401.7)	3172.76 (3053.14,3258.38)	79250.8 (73676.5,83494)	934.09 (866.47,985.49)	-4.11 (-4.3,-3.91)
Slovakia	371970.3 (356754.5,386306.5)	6333.28 (6065.6,6585.18)	291322.2 (255376.9,325886.2)	3080.71 (2709.5,3443.39)	-2.33 (-2.42,-2.24)
Slovenia	60091.5 (57001.2,62916.8)	2452.12 (2323.74,2565.45)	38794.3 (33672.3,43424.3)	822.83 (715.65,920.55)	-4.05 (-4.23,-3.86)
Solomon Islands	9521.8 (6265.8,12490.9)	6645.51 (4883.19,8426.39)	23458 (18314.4,30388.7)	6228.54 (5006.15,7901.08)	-0.2 (-0.34,-0.06)
Somalia	39526.5 (27906.2,56907.7)	1518.92 (1108.15,2120.76)	104973.5 (70905.8,151080.8)	1549.45 (1070.27,2167.12)	0.1 (-0.03,0.23)
South Africa	358547.9 (315697,396157.6)	1647.75 (1419.91,1832.5)	712623.1 (653012.9,778831.8)	1568.46 (1435.5,1705.97)	-0.28 (-0.67,0.11)
South Sudan	41463.2 (30599.2,54254.6)	1594.88 (1202.47,2080.42)	72728.9 (51569.7,99768.6)	1783.57 (1308.71,2389.28)	0.23 (0.12,0.33)
Spain	958506.3 (898836.6,995169.3)	1812.11 (1701,1881.1)	683818.5 (608926.8,731125.3)	678.71 (620.16,718.29)	-3.4 (-3.52,-3.28)
Sri Lanka	309442.7 (282789.8,341121.4)	2865.4 (2621.95,3141.99)	516832.3 (346225.9,704708.9)	1963.57 (1330.01,2662.39)	-0.96 (-1.18,-0.74)
Sudan	731803.4 (580739.1,918529.6)	7582.53 (6084,9429.17)	1101291.9 (809661.4,1493701.4)	5338.32 (4083.7,7066.87)	-1.3 (-1.36,-1.24)
Suriname	9651.2 (8809.7,10329.4)	3737.5 (3432.49,3989.92)	13044.5 (10222.3,16230.1)	2036.82 (1594.71,2533.57)	-1.88 (-2.17,-1.6)
Sweden	500078.4 (469633.5,517228.9)	3214.59 (3039.31,3320.43)	204178.8 (177046.8,226477.2)	860.82 (751.7,958.42)	-4.15 (-4.21,-4.09)
Switzerland	265875.9 (249597.5,275759.8)	2516.93 (2372.24,2605.56)	130831.9 (113068.2,142073.4)	639.19 (566.36,688.5)	-4.57 (-4.63,-4.51)
Syrian Arab Republic	444304 (362178.3,536576.9)	7886.6 (6446.11,9443.03)	833291.2 (640325.1,1088801.7)	6688.8 (5230.44,8518.87)	-0.87 (-1.01,-0.74)
Taiwan (Province of China)	193921.4 (186558.1,201384.1)	1344.59 (1274.64,1397.45)	284636.6 (259369.9,303351.4)	684.68 (626.58,728.31)	-2.04 (-2.28,-1.8)
Tajikistan	158927.6 (142105.3,174702.1)	5959.98 (5301.73,6541.09)	225428.2 (189841.2,264544.5)	4400.38 (3740.14,5106.84)	-1.3 (-1.61,-0.99)

Thailand	594811.5 (516376.1,688469.6)	1685.44 (1453.27,1952.61)	1082563.6 (855053.1,1334622.6)	1039.67 (825.63,1278.93)	-2.23 (-2.5,-1.95)
Timor-Leste	7895.7 (6051.4,9971.4)	2609.52 (2030.31,3225.76)	26597 (20307.1,34225.6)	3162.99 (2433.53,4007.89)	0.92 (0.69,1.15)
Togo	27647.4 (22667.4,33435.4)	2308.11 (1911.97,2750.5)	87013.8 (63688.2,115027.8)	2348.16 (1747.13,3034.4)	-0.18 (-0.41,0.04)
Tokelau	64.4 (52,78.1)	4934.68 (4021.12,5987.03)	65.6 (51.7,81.3)	4491.08 (3546.59,5579.27)	-0.34 (-0.41,-0.27)
Tonga	1745.4 (1453,2049.6)	3121.08 (2608.87,3637.35)	2487.8 (1992.4,3082.4)	3065.33 (2467.28,3781.66)	0.08 (-0.03,0.19)
Trinidad and Tobago	38084.2 (36867.8,39258.6)	4701.89 (4550,4842.64)	43145.7 (33277.7,54596.8)	2270.93 (1754.62,2870.76)	-2.98 (-3.26,-2.69)
Tunisia	198322.4 (165569.5,231085.1)	4177.62 (3489.93,4821.35)	388556 (288610.1,522602)	3036.73 (2267.13,4065.15)	-1.35 (-1.48,-1.23)
Turkmenistan	142412.5 (135891.4,148798.1)	7863.83 (7511.22,8203.8)	244162.6 (193227.1,304196.8)	6512.7 (5219.52,8027.65)	-1.92 (-2.15,-1.68)
Tuvalu	436.4 (364.3,512.4)	6413.68 (5421.46,7436.47)	646.8 (543.4,765.8)	6200.06 (5251.54,7307.55)	-1.57 (-1.99,-1.15)
Timor-Leste	1477735.1 (1299333.1,1653583.8)	4255.11 (3773.12,4724.64)	2187118.6 (1808029.1,2591481.2)	2419.28 (2008.07,2856.99)	-0.01 (-0.06,0.03)
Uganda	95861.3 (73870,122236.5)	1491.74 (1159.39,1875.76)	230376 (181841.4,290151)	1490 (1191.54,1887.39)	-0.71 (-1.12,-0.3)
Ukraine	4007782.2 (3828141.7,4144576.6)	5894.15 (5605.29,6105.82)	5092232 (3923541.8,6332932)	6522.69 (5003.4,8125.67)	-0.3 (-0.83,0.24)
United Arab Emirates	27575.1 (21571.8,34756.5)	5579.12 (4473.98,6897.43)	93693.1 (74473,114450.1)	2923.67 (2395.62,3422)	-0.86 (-1.29,-0.43)
United Kingdom	3430621.4 (3292789.3,3498042.7)	3846.95 (3702.17,3918.72)	1273352.3 (1174185,1329582.2)	983.93 (921.73,1021.77)	-4.76 (-4.96,-4.56)
United Republic of Tanzania	164449.3 (130732.3,203300.2)	1471.12 (1177.1,1814.31)	545222 (401153.8,696333.8)	2051.06 (1526.6,2613.31)	0.92 (0.8,1.05)
United States of America	10780285.7 (10063311.9,11136811.7)	3395.99 (3185.06,3501.2)	8751736.2 (8013896.5,9182127.6)	1527.33 (1412.25,1595.15)	-2.9 (-3.05,-2.75)
United States Virgin Islands	3242.6 (2775.1,3733.5)	4049.78 (3501.73,4614.36)	3259.8 (2649.8,4016.4)	1953.39 (1594.1,2390.09)	-2.25 (-2.39,-2.1)
Uruguay	112380.4 (107572.6,115700.1)	2929.71 (2799.36,3017.32)	66024.6 (61778.9,69216.6)	1196.72 (1130.23,1250.88)	-2.99 (-3.17,-2.8)
Uzbekistan	688499.5 (656073.6,717977.5)	6122.18 (5815.24,6372.06)	1475293.3 (1277200.1,1693239.5)	6218.8 (5449.76,7058.07)	0.05 (-0.31,0.42)
Vanuatu	5010.6 (3971.5,6309.4)	7311.54 (5948.63,9023.92)	13660.4 (11219.1,16310.6)	7189.7 (6037.23,8411.26)	-0.14 (-0.2,-0.08)
Venezuela (Bolivarian Republic of)	351531.9 (340162,361006.5)	3553.57 (3419.38,3653.1)	877872 (680797.5,1112893.5)	2941.77 (2286.73,3718.26)	-1.08 (-1.35,-0.81)
Viet Nam	522193.6 (414617.5,667117.5)	1328.22 (1057.07,1689.64)	1388815 (1115926.5,1666229.9)	1464.59 (1198.7,1740.5)	0.86 (0.64,1.09)
Yemen	359757.8 (268180.7,464614)	7122.84 (5449.87,9124.4)	784991.7 (575646.9,1035620.1)	5442.56 (4100.16,7104.85)	-1.11 (-1.21,-1.01)
Zambia	38406.9 (31154,48078.7)	1331.36 (1077.19,1647.99)	138713.6 (102916.2,179602.9)	1937.07 (1461.43,2469.87)	1.13 (1,1.26)
Zimbabwe	55688.4 (45914.9,66142)	1533.92 (1263.97,1808.48)	160441.8 (124310.1,206667.4)	2404.18 (1938.12,3035.67)	2.02 (1.42,2.61)

Table S5 Decomposition analysis of changes in IHD prevalence, incidence, mortality, and DALYs by SDI region from 1990 to 2021

Location	gender	Overll difference	Aging	Population	Epidemiological change
**Prevalence**					
Global	Male	80327173	33404079.61 (41.59%)	48627287.79 (60.54%)	-1704193.94 (-2.12%)
Global	Female	61779606	21861705.55 (35.39%)	36362274.08 (58.86%)	3555626.33 (5.76%)
Global	Both	1.42E+08	54158351.05 (38.11%)	85033409.18 (59.84%)	2915019.18 (2.05%)
High SDI	Male	6085121	7400259.18 (121.61%)	5620234.1 (92.36%)	-6935372.67 (-113.97%)
High SDI	Female	2898958	3584878.67 (123.66%)	3083281.38 (106.36%)	-3769202.3 (-130.02%)
High SDI	Both	8984078	10161934.43 (113.11%)	8538320.3 (95.04%)	-9716176.37 (-108.15%)
High-middle SDI	Male	17670701	10758669.07 (60.88%)	7564794.64 (42.81%)	-652762.75 (-3.69%)
High-middle SDI	Female	15409801	8372222.34 (54.33%)	6403376.9 (41.55%)	634201.58 (4.12%)
High-middle SDI	Both	33080502	18707950.84 (56.55%)	13931443.37 (42.11%)	441107.58 (1.33%)
Middle SDI	Male	32176579	14539490 (45.19%)	14079124.77 (43.76%)	3557964.26 (11.06%)
Middle SDI	Female	25280793	11308326.25 (44.73%)	11229780.95 (44.42%)	2742685.68 (10.85%)
Middle SDI	Both	57457372	25856892.29 (45%)	25414899.17 (44.23%)	6185580.45 (10.77%)
Low-middle SDI	Male	19329396	3887661.28 (20.11%)	13817341.75 (71.48%)	1624393.18 (8.4%)
Low-middle SDI	Female	14369431	3802466.01 (26.46%)	9585723.67 (66.71%)	981241.71 (6.83%)
Low-middle SDI	Both	33698828	7993549.83 (23.72%)	23535030.8 (69.84%)	2170246.97 (6.44%)
Low SDI	Male	5016023	-577078.36 (-11.5%)	5451995.27 (108.69%)	141105.87 (2.81%)
Low SDI	Female	3783726	-103525.15 (-2.74%)	3720089.22 (98.32%)	167161.81 (4.42%)
Low SDI	Both	8799749	-616897.43 (-7.01%)	9190291.59 (104.44%)	226354.49 (2.57%)
**Incidence**					
Global	Male	8890997	4277936.2 (48.12%)	6389105.21 (71.86%)	-1776044.54 (-19.98%)
Global	Female	7168163	3150069.93 (43.95%)	4914364.27 (68.56%)	-896271.55 (-12.5%)
Global	Both	16059160	7337657.27 (45.69%)	11309057.24 (70.42%)	-2587554.98 (-16.11%)
High SDI	Male	171892.9	978558.4 (569.28%)	735723.6 (428.01%)	-1542389.07 (-897.3%)
High SDI	Female	49830.98	607046.94 (1218.21%)	441156.84 (885.31%)	-998372.79 (-2003.52%)
High SDI	Both	221723.9	1489289.06 (671.69%)	1156539.45 (521.61%)	-2424104.6 (-1093.3%)
High-middle SDI	Male	1867603	1371196.71 (73.42%)	965572.32 (51.7%)	-469166.33 (-25.12%)
High-middle SDI	Female	1763992	1250865.37 (70.91%)	859218.03 (48.71%)	-346091.32 (-19.62%)
High-middle SDI	Both	3631595	2583595.9 (71.14%)	1821296.2 (50.15%)	-773297.32 (-21.29%)
Middle SDI	Male	3788288	1774481.48 (46.84%)	1774180.8 (46.83%)	239625.61 (6.33%)
Middle SDI	Female	3056194	1465363.36 (47.95%)	1426606.49 (46.68%)	164224.33 (5.37%)

Middle SDI	Both	6844482	3248714.41 (47.46%)	3214272.72 (46.96%)	381494.94 (5.57%)
Low-middle SDI	Male	2395233	501374.94 (20.93%)	1913824.83 (79.9%)	-19966.67 (-0.83%)
Low-middle SDI	Female	1772542	511228.49 (28.84%)	1322065.02 (74.59%)	-60751.42 (-3.43%)
Low-middle SDI	Both	4167775	1049208.31 (25.17%)	3254535.27 (78.09%)	-135968.39 (-3.26%)
Low SDI	Male	664293.7	-71622.3 (-10.78%)	791462.99 (119.14%)	-55546.97 (-8.36%)
Low SDI	Female	522262.2	-10305.34 (-1.97%)	555647.89 (106.39%)	-23080.33 (-4.42%)
Low SDI	Both	1186556	-74405.95 (-6.27%)	1349783.35 (113.76%)	-88821.47 (-7.49%)
**Mortality**					
Global	Male	2197731	1570115.3 (71.44%)	1891066.76 (86.05%)	-1263451.09 (-57.49%)
Global	Female	1426769	1337513.69 (93.74%)	1652932.04 (115.85%)	-1563676.6 (-109.6%)
Global	Both	3624500	2866337.64 (79.08%)	3545048.62 (97.81%)	-2786886.16 (-76.89%)
High SDI	Male	-124783	502988.66 (-403.09%)	280925.1 (-225.13%)	-908696.26 (728.22%)
High SDI	Female	-215239	409164.45 (-190.1%)	217996.03 (-101.28%)	-842399.07 (391.38%)
High SDI	Both	-340021	861813.57 (-253.46%)	493626.03 (-145.18%)	-1695460.7 (498.63%)
High-middle SDI	Male	452563	557273.38 (123.14%)	318383.61 (70.35%)	-423093.99 (-93.49%)
High-middle SDI	Female	393001.2	574095.33 (146.08%)	315320.25 (80.23%)	-496414.41 (-126.31%)
High-middle SDI	Both	845564.2	1111092.75 (131.4%)	633334.19 (74.9%)	-898862.76 (-106.3%)
Middle SDI	Male	1055970	563718.7 (53.38%)	490068.17 (46.41%)	2182.66 (0.21%)
Middle SDI	Female	716183.8	477600.81 (66.69%)	395287.66 (55.19%)	-156704.62 (-21.88%)
Middle SDI	Both	1772153	1042524.34 (58.83%)	888935.64 (50.16%)	-159306.61 (-8.99%)
Low-middle SDI	Male	651900.2	134269.1 (20.6%)	449844 (69.01%)	67787.08 (10.4%)
Low-middle SDI	Female	429622.9	156026.61 (36.32%)	349149.69 (81.27%)	-75553.42 (-17.59%)
Low-middle SDI	Both	1081523	297525.85 (27.51%)	801389.76 (74.1%)	-17392.54 (-1.61%)
Low SDI	Male	161900.3	-14096.09 (-8.71%)	173930 (107.43%)	2066.38 (1.28%)
Low SDI	Female	102764.1	-151.41 (-0.15%)	131291.86 (127.76%)	-28376.38 (-27.61%)
Low SDI	Both	264664.4	-12610.92 (-4.76%)	305525.06 (115.44%)	-28249.78 (-10.67%)
**DALYs (disability-adjusted life years)**					
Global	Male	45364618	27792812.01 (61.27%)	44866052.69 (98.9%)	-27294246.71 (-60.17%)
Global	Female	23832982	19234604.75 (80.71%)	30918513.57 (129.73%)	-26320136.28 (-110.44%)
Global	Both	69197600	46456256.22 (67.14%)	75876337.76 (109.65%)	-53134993.95 (-76.79%)
High SDI	Male	-4246965	7542518.47 (-177.6%)	5618856.42 (-132.3%)	-17408339.81 (409.9%)
High SDI	Female	-4212066	4723071.69 (-112.13%)	3219534.92 (-76.44%)	-12154672.4 (288.57%)
High SDI	Both	-8459031	11509583.23 (-136.06%)	8668370.79 (-102.47%)	-28636984.74 (338.54%)

High-middle SDI	Male	6679353	9673874.5 (144.83%)	7133572.7 (106.8%)	-10128094.38 (-151.63%)
High-middle SDI	Female	4294178	7623288.38 (177.53%)	5180089.05 (120.63%)	-8509199.52 (-198.16%)
High-middle SDI	Both	10973531	16939967.99 (154.37%)	12255524.27 (111.68%)	-18221961.53 (-166.05%)
Middle SDI	Male	22398539	10917046.52 (48.74%)	12236064.41 (54.63%)	-754572.24 (-3.37%)
Middle SDI	Female	12317184	7918494.99 (64.29%)	8269764.7 (67.14%)	-3871075.96 (-31.43%)
Middle SDI	Both	34715722	18904329.39 (54.45%)	20644159.59 (59.47%)	-4832766.56 (-13.92%)
Low-middle SDI	Male	16312792	2829842.47 (17.35%)	12324966.3 (75.55%)	1157983.61 (7.1%)
Low-middle SDI	Female	9072803	2868493.2 (31.62%)	8552424.38 (94.26%)	-2348114.65 (-25.88%)
Low-middle SDI	Both	25385595	5878520.55 (23.16%)	20974860.03 (82.63%)	-1467785.28 (-5.78%)
Low SDI	Male	4223658	-411953.79 (-9.75%)	4767006.24 (112.86%)	-131394.84 (-3.11%)
Low SDI	Female	2359694	-71500.86 (-3.03%)	3325510.52 (140.93%)	-894315.23 (-37.9%)
Low SDI	Both	6583352	-439713.61 (-6.68%)	8104134.32 (123.1%)	-1081068.67 (-16.42%)

Table S6 Ranking of risk factors contributing to the global and regional ASDR for IHD.

Location	1990		2021		rank
	Cause	Rate	Cause	Rate	
Global	Metabolic risks	2401.268	Metabolic risks	1715.062	1
Global	Behavioral risks	1979.496	Behavioral risks	1299.398	2
Global	Dietary risks	1608.138	High systolic blood pressure	1101.198	3
Global	High systolic blood pressure	1577.613	Dietary risks	1049.015	4
Global	Environmental/occupational risks	1179.602	Environmental/occupational risks	827.5175	5
Global	High LDL cholesterol	1141.433	High LDL cholesterol	776.6854	6
Global	Air pollution	936.3943	Particulate matter pollution	638.4753	7
Global	Particulate matter pollution	936.3943	Air pollution	638.4753	8
Global	Tobacco	832.0006	Tobacco	499.5911	9
Global	Smoking	691.9373	Ambient particulate matter pollution	427.8054	10
Global	Ambient particulate matter pollution	479.8667	Smoking	408.7846	11
Global	Diet low in whole grains	473.6862	Diet low in whole grains	316.4518	12
Global	Household air pollution from solid fuels	456.3961	Kidney dysfunction	309.8422	13
Global	Kidney dysfunction	450.953	High fasting plasma glucose	303.487	14
Global	Diet low in nuts and seeds	331.8173	High body-mass index	277.6533	15
Global	Diet low in seafood omega-3 fatty acids	322.9323	Household air pollution from solid fuels	210.5635	16
Global	High fasting plasma glucose	304.9838	Diet low in polyunsaturated fatty acids	207.9408	17
Global	High body-mass index	300.7774	Diet low in nuts and seeds	189.8848	18
Global	Diet low in polyunsaturated fatty acids	282.829	Diet low in fruits	185.43	19
Global	Diet low in fruits	278.8656	Diet low in seafood omega-3 fatty acids	181.0693	20
Global	Diet low in fiber	243.195	Diet high in sodium	163.3716	21
Global	Diet high in sodium	213.8777	Non-optimal temperature	146.026	22
Global	Non-optimal temperature	201.9395	Other environmental risks	138.5707	23
Global	Diet low in legumes	192.6091	Lead exposure	138.5707	24
Global	Secondhand smoke	183.4312	Diet low in fiber	135.3162	25
Global	Low temperature	183.2717	Low temperature	117.3951	26
Global	Lead exposure	166.1078	Secondhand smoke	113.4428	27
Global	Other environmental risks	166.1078	Diet low in legumes	107.0196	28
Global	Diet low in vegetables	122.1213	Diet low in vegetables	71.25525	29
Global	Diet high in red meat	89.00811	Diet high in red meat	51.38045	30
Global	Diet high in trans fatty acids	82.96681	Low physical activity	46.63027	31

Global	Diet high in processed meat	75.85708	High temperature	30.57483	32
Global	Low physical activity	64.50592	Diet high in trans fatty acids	28.92239	33
Global	High temperature	20.18182	Diet high in processed meat	22.06089	34
Global	Diet high in sugar-sweetened beverages	2.653589	Diet high in sugar-sweetened beverages	2.340461	35
Global	Alcohol use	-62.3767	Alcohol use	-38.8133	36
High SDI	Metabolic risks	2395.388	Metabolic risks	897.9295	1
High SDI	Behavioral risks	1814.876	Behavioral risks	642.2162	2
High SDI	High systolic blood pressure	1640.862	High systolic blood pressure	529.3168	3
High SDI	Dietary risks	1391.048	Dietary risks	509.8892	4
High SDI	High LDL cholesterol	1189.924	High LDL cholesterol	428.9386	5
High SDI	Tobacco	887.5697	Tobacco	272.9719	6
High SDI	Smoking	786.7895	Environmental/occupational risks	240.1128	7
High SDI	Environmental/occupational risks	761.9735	Smoking	238.9292	8
High SDI	Air pollution	522.597	High body-mass index	211.8527	9
High SDI	Particulate matter pollution	522.597	High fasting plasma glucose	190.4199	10
High SDI	Ambient particulate matter pollution	489.5449	Diet low in whole grains	163.6087	11
High SDI	Kidney dysfunction	408.1834	Kidney dysfunction	156.043	12
High SDI	Diet low in whole grains	390.0583	Air pollution	140.694	13
High SDI	High body-mass index	387.6439	Particulate matter pollution	140.694	14
High SDI	High fasting plasma glucose	321.1244	Ambient particulate matter pollution	140.0725	15
High SDI	Diet low in nuts and seeds	222.5108	Non-optimal temperature	83.19506	16
High SDI	Diet low in fiber	214.7639	Low temperature	72.05705	17
High SDI	Diet low in seafood omega-3 fatty acids	204.6858	Diet low in seafood omega-3 fatty acids	71.5689	18
High SDI	Diet low in polyunsaturated fatty acids	197.8193	Diet high in sodium	69.84668	19
High SDI	Non-optimal temperature	196.5971	Diet low in legumes	65.42378	20
High SDI	Low temperature	187.1547	Diet low in polyunsaturated fatty acids	64.34434	21
High SDI	Diet low in legumes	184.9329	Diet low in fiber	63.8733	22
High SDI	Diet low in fruits	180.9541	Diet high in processed meat	61.6176	23
High SDI	Diet high in sodium	154.3537	Diet low in nuts and seeds	60.84643	24
High SDI	Secondhand smoke	152.9911	Diet low in fruits	59.97543	25
High SDI	Diet high in red meat	151.1923	Diet high in red meat	56.4043	26
High SDI	Diet high in trans fatty acids	132.898	Secondhand smoke	46.47255	27

High SDI	Diet high in processed meat	119.4087	Other environmental risks	36.17708	28
High SDI	Other environmental risks	102.4071	Lead exposure	36.17708	29
High SDI	Lead exposure	102.4071	Diet low in vegetables	31.92709	30
High SDI	Diet low in vegetables	92.61661	Low physical activity	21.76968	31
High SDI	Low physical activity	61.50332	High temperature	12.04592	32
High SDI	Household air pollution from solid fuels	32.98606	Diet high in trans fatty acids	4.848775	33
High SDI	High temperature	10.28548	Diet high in sugar-sweetened beverages	3.330847	34
High SDI	Diet high in sugar-sweetened beverages	4.583915	Household air pollution from solid fuels	0.610691	35
High SDI	Alcohol use	-81.3814	Alcohol use	-36.3189	36
High-middle SDI	Metabolic risks	2845.609	Metabolic risks	1831.82	1
High-middle SDI	Behavioral risks	2344.956	Behavioral risks	1359.794	2
High-middle SDI	Dietary risks	1940.114	High systolic blood pressure	1233.714	3
High-middle SDI	High systolic blood pressure	1932.885	Dietary risks	1060.829	4
High-middle SDI	High LDL cholesterol	1372.405	High LDL cholesterol	838.1005	5
High-middle SDI	Environmental/occupational risks	1280.03	Environmental/occupational risks	696.375	6
High-middle SDI	Air pollution	1018.956	Tobacco	593.8218	7
High-middle SDI	Particulate matter pollution	1018.956	Air pollution	501.3943	8
High-middle SDI	Tobacco	989.1981	Particulate matter pollution	501.3943	9
High-middle SDI	Smoking	805.2736	Smoking	486.9624	10
High-middle SDI	Ambient particulate matter pollution	725.4532	Ambient particulate matter pollution	466.5264	11
High-middle SDI	Diet low in whole grains	672.2576	Diet low in whole grains	367.8884	12
High-middle SDI	Kidney dysfunction	513.4557	High body-mass index	337.1055	13
High-middle SDI	Diet low in nuts and seeds	436.4398	Kidney dysfunction	310.4673	14
High-middle SDI	High body-mass index	414.3732	High fasting plasma glucose	291.5948	15
High-middle SDI	Diet low in polyunsaturated fatty acids	347.0468	Diet high in sodium	212.7428	16
High-middle SDI	High fasting plasma glucose	315.7038	Diet low in polyunsaturated fatty acids	209.9586	17
High-middle SDI	Diet low in seafood omega-3 fatty acids	312.2537	Diet low in nuts and seeds	184.8411	18
High-middle SDI	Diet high in sodium	299.8466	Non-optimal temperature	158.1359	19
High-middle SDI	Household air pollution from solid fuels	293.3016	Low temperature	146.3797	20
High-middle SDI	Diet low in fruits	283.0108	Secondhand smoke	138.3924	21
High-middle SDI	Diet low in legumes	258.7851	Diet low in fruits	122.5705	22
High-middle SDI	Non-optimal temperature	244.7826	Diet low in seafood omega-3 fatty acids	120.5908	23

High-middle SDI	Secondhand smoke	240.0636	Diet low in legumes	117.9774	24
High-middle SDI	Low temperature	236.9957	Other environmental risks	103.4494	25
High-middle SDI	Diet low in fiber	207.859	Lead exposure	103.4494	26
High-middle SDI	Diet high in processed meat	170.0028	Diet low in fiber	100.5641	27
High-middle SDI	Diet high in red meat	147.1791	Diet high in red meat	87.97461	28
High-middle SDI	Other environmental risks	132.971	Low physical activity	49.50622	29
High-middle SDI	Lead exposure	132.971	Diet low in vegetables	36.50658	30
High-middle SDI	Diet low in vegetables	96.9798	Household air pollution from solid fuels	34.76105	31
High-middle SDI	Low physical activity	66.73432	Diet high in processed meat	30.02286	32
High-middle SDI	Diet high in trans fatty acids	16.14595	High temperature	12.66894	33
High-middle SDI	High temperature	8.457728	Diet high in trans fatty acids	4.631223	34
High-middle SDI	Diet high in sugar-sweetened beverages	2.840147	Diet high in sugar-sweetened beverages	2.187922	35
High-middle SDI	Alcohol use	-89.9213	Alcohol use	-50.7237	36
Middle SDI	Metabolic risks	1918.254	Metabolic risks	1813.985	1
Middle SDI	Behavioral risks	1669.305	Behavioral risks	1357.391	2
Middle SDI	Dietary risks	1344.269	High systolic blood pressure	1165.325	3
Middle SDI	High systolic blood pressure	1174.387	Dietary risks	1077.409	4
Middle SDI	Environmental/occupational risks	1167.153	Environmental/occupational risks	927.6687	5
Middle SDI	Air pollution	965.4811	High LDL cholesterol	809.3686	6
Middle SDI	Particulate matter pollution	965.4811	Particulate matter pollution	728.1251	7
Middle SDI	High LDL cholesterol	896.0148	Air pollution	728.1251	8
Middle SDI	Tobacco	707.3345	Ambient particulate matter pollution	580.4367	9
Middle SDI	Household air pollution from solid fuels	612.28	Tobacco	524.3884	10
Middle SDI	Smoking	565.1271	Smoking	417.3004	11
Middle SDI	Kidney dysfunction	390.7428	Kidney dysfunction	335.7935	12
Middle SDI	Diet low in whole grains	361.7431	High fasting plasma glucose	321.4759	13
Middle SDI	Ambient particulate matter pollution	353.0843	Diet low in whole grains	305.9498	14
Middle SDI	Diet low in seafood omega-3 fatty acids	322.3987	High body-mass index	265.3207	15
Middle SDI	High fasting plasma glucose	270.7785	Diet low in polyunsaturated fatty acids	224.5638	16
Middle SDI	Diet low in nuts and seeds	268.0682	Diet high in sodium	202.2619	17
Middle SDI	Diet low in fruits	255.1617	Diet low in fruits	181.6367	18
Middle SDI	Diet low in polyunsaturated fatty acids	251.8533	Diet low in nuts and seeds	180.8312	19

Middle SDI	Diet low in fiber	237.2483	Diet low in seafood omega-3 fatty acids	179.7904	20
Middle SDI	Diet high in sodium	234.2297	Other environmental risks	157.0221	21
Middle SDI	High body-mass index	183.4812	Lead exposure	157.0221	22
Middle SDI	Secondhand smoke	178.2471	Non-optimal temperature	147.9841	23
Middle SDI	Lead exposure	172.5342	Household air pollution from solid fuels	147.5341	24
Middle SDI	Other environmental risks	172.5342	Diet low in fiber	139.1726	25
Middle SDI	Non-optimal temperature	161.0443	Secondhand smoke	130.3492	26
Middle SDI	Low temperature	143.3172	Low temperature	119.004	27
Middle SDI	Diet low in legumes	142.7504	Diet low in legumes	105.4065	28
Middle SDI	Diet low in vegetables	113.4483	Diet low in vegetables	66.92292	29
Middle SDI	Low physical activity	61.60825	Low physical activity	57.70523	30
Middle SDI	Diet high in trans fatty acids	56.81156	Diet high in red meat	53.53625	31
Middle SDI	Diet high in red meat	35.13489	High temperature	31.0453	32
Middle SDI	High temperature	19.45564	Diet high in trans fatty acids	29.77684	33
Middle SDI	Diet high in processed meat	3.024638	Diet high in processed meat	3.230567	34
Middle SDI	Diet high in sugar-sweetened beverages	1.632466	Diet high in sugar-sweetened beverages	2.337824	35
Middle SDI	Alcohol use	-42.6414	Alcohol use	-37.1928	36
Low-middle SDI	Metabolic risks	2321.769	Metabolic risks	2407.859	1
Low-middle SDI	Behavioral risks	2111.338	Behavioral risks	1888.629	2
Low-middle SDI	Dietary risks	1765.925	High systolic blood pressure	1561.504	3
Low-middle SDI	Environmental/occupational risks	1630.101	Dietary risks	1560.057	4
Low-middle SDI	High systolic blood pressure	1429.858	Environmental/occupational risks	1482.023	5
Low-middle SDI	Particulate matter pollution	1361.988	Particulate matter pollution	1198.302	6
Low-middle SDI	Air pollution	1361.988	Air pollution	1198.302	7
Low-middle SDI	High LDL cholesterol	1056.747	High LDL cholesterol	1032.338	8
Low-middle SDI	Household air pollution from solid fuels	1050.748	Tobacco	666.6996	9
Low-middle SDI	Tobacco	800.3018	Household air pollution from solid fuels	634.5846	10
Low-middle SDI	Smoking	648.6527	Ambient particulate matter pollution	563.5577	11
Low-middle SDI	Diet low in seafood omega-3 fatty acids	466.4603	Smoking	543.4804	12
Low-middle SDI	Kidney dysfunction	462.1629	Kidney dysfunction	457.9091	13
Low-middle SDI	Diet low in whole grains	460.2276	High fasting plasma glucose	449.5783	14
Low-middle SDI	Diet low in nuts and seeds	434.993	Diet low in whole grains	424.551	15

Low-middle SDI	Diet low in fruits	421.8402	Diet low in fruits	372.1952	16
Low-middle SDI	Diet low in polyunsaturated fatty acids	345.5736	Diet low in nuts and seeds	338.0819	17
Low-middle SDI	Diet low in fiber	328.7319	Diet low in seafood omega-3 fatty acids	329.2352	18
Low-middle SDI	Ambient particulate matter pollution	311.077	Diet low in polyunsaturated fatty acids	327.017	19
Low-middle SDI	High fasting plasma glucose	300.8437	High body-mass index	322.563	20
Low-middle SDI	Other environmental risks	285.9132	Other environmental risks	275.7079	21
Low-middle SDI	Lead exposure	285.9132	Lead exposure	275.7079	22
Low-middle SDI	Non-optimal temperature	202.0325	Diet low in fiber	238.7652	23
Low-middle SDI	High body-mass index	191.3699	Non-optimal temperature	211.7771	24
Low-middle SDI	Secondhand smoke	187.5014	Diet high in sodium	170.6053	25
Low-middle SDI	Diet low in vegetables	179.2308	Secondhand smoke	150.4041	26
Low-middle SDI	Diet low in legumes	175.2384	Low temperature	137.9253	27
Low-middle SDI	Diet high in sodium	169.6897	Diet low in legumes	135.7044	28
Low-middle SDI	Low temperature	150.2029	Diet low in vegetables	133.1675	29
Low-middle SDI	Diet high in trans fatty acids	132.7573	Diet high in trans fatty acids	84.83756	30
Low-middle SDI	Low physical activity	68.8427	High temperature	78.48548	31
Low-middle SDI	High temperature	55.56026	Low physical activity	65.79168	32
Low-middle SDI	Diet high in red meat	7.310347	Diet high in red meat	12.35027	33
Low-middle SDI	Diet high in processed meat	4.994181	Diet high in processed meat	6.323634	34
Low-middle SDI	Diet high in sugar-sweetened beverages	1.499817	Diet high in sugar-sweetened beverages	2.24696	35
Low-middle SDI	Alcohol use	-29.7659	Alcohol use	-30.2748	36
Low SDI	Metabolic risks	1870.511	Metabolic risks	1807.58	1
Low SDI	Behavioral risks	1623.644	Behavioral risks	1419.689	2
Low SDI	Dietary risks	1434.741	Environmental/occupational risks	1293.39	3
Low SDI	Environmental/occupational risks	1418.487	Dietary risks	1258.788	4
Low SDI	Particulate matter pollution	1210.908	High systolic blood pressure	1168.222	5
Low SDI	Air pollution	1210.908	Particulate matter pollution	1094.123	6
Low SDI	High systolic blood pressure	1188.708	Air pollution	1094.123	7
Low SDI	Household air pollution from solid fuels	1017.014	Household air pollution from solid fuels	853.614	8
Low SDI	High LDL cholesterol	782.4191	High LDL cholesterol	727.7379	9
Low SDI	Tobacco	454.1675	Diet low in whole grains	369.1409	10
Low SDI	Diet low in whole grains	425.0025	Tobacco	362.9488	11

Low SDI	Diet low in seafood omega-3 fatty acids	414.0725	Kidney dysfunction	348.4317	12
Low SDI	Kidney dysfunction	373.3209	Diet low in seafood omega-3 fatty acids	336.6821	13
Low SDI	Smoking	367.1596	High fasting plasma glucose	314.3273	14
Low SDI	Diet low in fruits	325.7326	Smoking	293.9081	15
Low SDI	Diet low in polyunsaturated fatty acids	294.0069	Diet low in fruits	290.3377	16
Low SDI	Diet low in nuts and seeds	278.8125	Diet low in polyunsaturated fatty acids	260.6998	17
Low SDI	Other environmental risks	253.4403	Ambient particulate matter pollution	240.424	18
Low SDI	Lead exposure	253.4403	Other environmental risks	239.6935	19
Low SDI	High fasting plasma glucose	243.2525	Lead exposure	239.6935	20
Low SDI	Diet low in vegetables	202.7354	Diet low in nuts and seeds	234.6028	21
Low SDI	Ambient particulate matter pollution	193.7641	High body-mass index	180.5927	22
Low SDI	Diet low in fiber	190.0286	Diet low in vegetables	162.0174	23
Low SDI	Diet low in legumes	143.7103	Diet low in fiber	157.1843	24
Low SDI	Non-optimal temperature	142.4257	Non-optimal temperature	133.9076	25
Low SDI	Low temperature	119.0236	Diet low in legumes	117.069	26
Low SDI	High body-mass index	118.0393	Diet high in sodium	105.3343	27
Low SDI	Diet high in sodium	107.6433	Low temperature	100.3972	28
Low SDI	Secondhand smoke	103.7217	Secondhand smoke	81.37824	29
Low SDI	Diet high in trans fatty acids	54.1412	High temperature	35.28974	30
Low SDI	Low physical activity	36.15662	Low physical activity	32.82885	31
Low SDI	High temperature	24.90302	Diet high in trans fatty acids	30.79089	32
Low SDI	Diet high in red meat	6.540563	Diet high in processed meat	5.742283	33
Low SDI	Diet high in processed meat	5.058737	Diet high in red meat	3.223635	34
Low SDI	Diet high in sugar-sweetened beverages	0.873998	Diet high in sugar-sweetened beverages	0.930757	35
Low SDI	Alcohol use	-31.7683	Alcohol use	-30.747	36
Andean Latin America	Metabolic risks	1187.933	Metabolic risks	846.8671	1
Andean Latin America	Behavioral risks	1006.136	Behavioral risks	558.7947	2
Andean Latin America	Dietary risks	906.7752	Dietary risks	502.5983	3
Andean Latin America	Environmental/occupational risks	881.4824	High systolic blood pressure	478.6229	4
Andean Latin America	Air pollution	777.8308	High LDL cholesterol	402.5818	5
Andean Latin America	Particulate matter pollution	777.8308	Environmental/occupational risks	365.6901	6
Andean Latin America	High LDL cholesterol	627.1046	Air pollution	282.8654	7

Andean Latin America	High systolic blood pressure	544.5041	Particulate matter pollution	282.8654	8
Andean Latin America	Ambient particulate matter pollution	440.5438	Ambient particulate matter pollution	236.5555	9
Andean Latin America	Household air pollution from solid fuels	337.1419	High body-mass index	190.2279	10
Andean Latin America	Diet low in whole grains	270.4692	High fasting plasma glucose	150.3755	11
Andean Latin America	Tobacco	252.6133	Diet low in whole grains	144.296	12
Andean Latin America	Diet low in nuts and seeds	224.0182	Tobacco	133.6565	13
Andean Latin America	Smoking	216.683	Kidney dysfunction	123.7923	14
Andean Latin America	High body-mass index	202.1836	Smoking	119.421	15
Andean Latin America	Kidney dysfunction	193.8659	Diet low in nuts and seeds	101.3993	16
Andean Latin America	Diet low in seafood omega-3 fatty acids	193.1771	Diet low in seafood omega-3 fatty acids	95.71687	17
Andean Latin America	Diet low in fiber	187.502	Diet low in polyunsaturated fatty acids	91.77162	18
Andean Latin America	Diet low in polyunsaturated fatty acids	158.7984	Diet low in fiber	87.63448	19
Andean Latin America	High fasting plasma glucose	146.6475	Diet high in sodium	64.69976	20
Andean Latin America	Diet high in sodium	107.9461	Other environmental risks	62.94196	21
Andean Latin America	Diet low in vegetables	99.61171	Lead exposure	62.94196	22
Andean Latin America	Diet low in fruits	97.84873	Diet low in legumes	58.13639	23
Andean Latin America	Other environmental risks	94.7922	Diet low in vegetables	57.03234	24
Andean Latin America	Lead exposure	94.7922	Low temperature	51.28759	25
Andean Latin America	Low temperature	91.97305	Non-optimal temperature	51.26037	26
Andean Latin America	Non-optimal temperature	90.54796	Household air pollution from solid fuels	46.21981	27
Andean Latin America	Diet low in legumes	89.85048	Diet low in fruits	39.89601	28
Andean Latin America	Diet high in trans fatty acids	56.29616	Diet high in red meat	22.82137	29
Andean Latin America	Secondhand smoke	41.51577	Diet high in trans fatty acids	22.2202	30
Andean Latin America	Diet high in red meat	19.79772	Secondhand smoke	16.4056	31
Andean Latin America	Low physical activity	16.63777	Low physical activity	10.3194	32
Andean Latin America	Diet high in sugar-sweetened beverages	2.12558	Diet high in sugar-sweetened beverages	1.951232	33
Andean Latin America	Diet high in processed meat	1.075956	Diet high in processed meat	0.949285	34
Andean Latin America	High temperature	-1.49618	High temperature	-0.02475	35
Andean Latin America	Alcohol use	-63.7047	Alcohol use	-38.8765	36
Australasia	Metabolic risks	2636.35	Metabolic risks	641.3243	1
Australasia	High systolic blood pressure	1837.293	Behavioral risks	403.033	2
Australasia	Behavioral risks	1836.063	High systolic blood pressure	383.2362	3

Australasia	Dietary risks	1429.738	Dietary risks	338.7463	4
Australasia	High LDL cholesterol	1293.532	High LDL cholesterol	320.6977	5
Australasia	Tobacco	760.3543	High body-mass index	151.3589	6
Australasia	Smoking	668.2322	Environmental/occupational risks	143.5184	7
Australasia	Environmental/occupational risks	560.9269	Tobacco	136.5344	8
Australasia	High body-mass index	418.2122	Smoking	119.3016	9
Australasia	Kidney dysfunction	407.5155	High fasting plasma glucose	113.3131	10
Australasia	Diet low in whole grains	344.4946	Kidney dysfunction	102.0276	11
Australasia	High fasting plasma glucose	317.5633	Diet low in whole grains	101.0933	12
Australasia	Diet low in polyunsaturated fatty acids	305.9113	Diet low in polyunsaturated fatty acids	76.18601	13
Australasia	Diet low in fiber	281.2929	Particulate matter pollution	58.18042	14
Australasia	Non-optimal temperature	233.3308	Air pollution	58.18042	15
Australasia	Low temperature	229.2251	Ambient particulate matter pollution	58.13095	16
Australasia	Diet high in red meat	220.085	Diet low in fiber	57.14108	17
Australasia	Diet low in seafood omega-3 fatty acids	219.4377	Diet high in red meat	56.14935	18
Australasia	Diet low in fruits	201.011	Non-optimal temperature	53.36884	19
Australasia	Other environmental risks	187.7313	Low temperature	52.78706	20
Australasia	Lead exposure	187.7313	Diet low in legumes	44.01793	21
Australasia	Diet low in legumes	181.3925	Diet low in fruits	43.68213	22
Australasia	Particulate matter pollution	176.0157	Other environmental risks	41.29092	23
Australasia	Air pollution	176.0157	Lead exposure	41.29092	24
Australasia	Diet low in nuts and seeds	174.4247	Diet low in seafood omega-3 fatty acids	33.98971	25
Australasia	Ambient particulate matter pollution	173.2005	Diet low in vegetables	27.33798	26
Australasia	Diet high in trans fatty acids	159.0366	Diet low in nuts and seeds	26.76198	27
Australasia	Secondhand smoke	128.7141	Diet high in trans fatty acids	22.93936	28
Australasia	Diet low in vegetables	116.2777	Secondhand smoke	22.53987	29
Australasia	Diet high in sodium	83.792	Diet high in processed meat	22.26887	30
Australasia	Low physical activity	80.87169	Diet high in sodium	21.94135	31
Australasia	Diet high in processed meat	56.60877	Low physical activity	20.67501	32
Australasia	Diet high in sugar-sweetened beverages	5.220682	Diet high in sugar-sweetened beverages	1.623075	33
Australasia	High temperature	4.428307	High temperature	0.625023	34
Australasia	Household air pollution from solid fuels	2.756294	Household air pollution from solid fuels	0.046025	35

Australasia	Alcohol use	-66.3257	Alcohol use	-40.2793	36
Caribbean	Metabolic risks	2760.838	Metabolic risks	1850.127	1
Caribbean	Behavioral risks	2045.723	Behavioral risks	1176.532	2
Caribbean	High systolic blood pressure	1691.552	High systolic blood pressure	1156.356	3
Caribbean	Dietary risks	1593.029	Dietary risks	928.0384	4
Caribbean	High LDL cholesterol	1277.066	High LDL cholesterol	859.4843	5
Caribbean	Environmental/occupational risks	1181.847	Environmental/occupational risks	765.288	6
Caribbean	Air pollution	945.4959	Air pollution	605.4293	7
Caribbean	Particulate matter pollution	945.4959	Particulate matter pollution	605.4293	8
Caribbean	Tobacco	836.1825	Tobacco	422.3357	9
Caribbean	Smoking	706.4244	Smoking	366.6543	10
Caribbean	Diet low in whole grains	578.8579	High fasting plasma glucose	359.4091	11
Caribbean	Household air pollution from solid fuels	484.0348	Diet low in whole grains	341.3916	12
Caribbean	High fasting plasma glucose	481.2795	Ambient particulate matter pollution	340.6314	13
Caribbean	Ambient particulate matter pollution	461.2644	High body-mass index	339.3094	14
Caribbean	Kidney dysfunction	445.8497	Kidney dysfunction	285.7855	15
Caribbean	Diet low in seafood omega-3 fatty acids	388.1672	Household air pollution from solid fuels	264.7036	16
Caribbean	High body-mass index	348.2266	Diet low in seafood omega-3 fatty acids	242.3667	17
Caribbean	Diet low in fiber	322.9251	Other environmental risks	197.4597	18
Caribbean	Diet low in polyunsaturated fatty acids	293.3553	Lead exposure	197.4597	19
Caribbean	Other environmental risks	293.3412	Diet low in polyunsaturated fatty acids	141.0057	20
Caribbean	Lead exposure	293.3412	Diet low in vegetables	137.7174	21
Caribbean	Diet low in vegetables	208.8445	Diet low in fiber	137.3281	22
Caribbean	Diet low in nuts and seeds	198.8441	Diet high in sodium	86.90847	23
Caribbean	Secondhand smoke	166.6634	Diet low in fruits	83.86294	24
Caribbean	Diet low in fruits	155.6644	Diet low in legumes	80.03384	25
Caribbean	Diet high in sodium	149.2657	Diet low in nuts and seeds	77.70909	26
Caribbean	Diet low in legumes	138.1546	Secondhand smoke	68.14335	27
Caribbean	Low physical activity	62.15157	Low physical activity	37.19178	28
Caribbean	Low temperature	41.02628	Non-optimal temperature	24.91031	29
Caribbean	Non-optimal temperature	40.48011	Low temperature	23.63006	30
Caribbean	Diet high in red meat	30.87527	Diet high in red meat	23.54254	31

Caribbean	Diet high in processed meat	3.789899	Diet high in sugar-sweetened beverages	3.009747	32
Caribbean	Diet high in sugar-sweetened beverages	3.608813	Diet high in processed meat	2.866145	33
Caribbean	Diet high in trans fatty acids	0.82529	High temperature	1.30512	34
Caribbean	High temperature	-0.53305	Diet high in trans fatty acids	0.123989	35
Caribbean	Alcohol use	-74.6655	Alcohol use	-56.1711	36
Central Asia	Metabolic risks	4852.757	Metabolic risks	3935.353	1
Central Asia	Behavioral risks	4266.508	Behavioral risks	3117.182	2
Central Asia	Dietary risks	3762.756	Dietary risks	2706.011	3
Central Asia	High systolic blood pressure	3262.48	High systolic blood pressure	2699.805	4
Central Asia	Environmental/occupational risks	2248.753	Environmental/occupational risks	1749.564	5
Central Asia	High LDL cholesterol	2103.918	High LDL cholesterol	1604.734	6
Central Asia	Air pollution	1745.784	Air pollution	1321.041	7
Central Asia	Particulate matter pollution	1745.784	Particulate matter pollution	1321.041	8
Central Asia	Tobacco	1463.094	Ambient particulate matter pollution	1078.166	9
Central Asia	Diet low in whole grains	1443.306	Diet low in whole grains	1071.066	10
Central Asia	Smoking	1137.616	Tobacco	1006.284	11
Central Asia	Kidney dysfunction	1134.164	Kidney dysfunction	923.2197	12
Central Asia	Diet low in seafood omega-3 fatty acids	947.5153	High body-mass index	774.2255	13
Central Asia	Diet low in nuts and seeds	933.4436	Smoking	766.9846	14
Central Asia	Ambient particulate matter pollution	922.8615	Diet low in seafood omega-3 fatty acids	678.3957	15
Central Asia	High body-mass index	830.2323	Diet low in nuts and seeds	650.7893	16
Central Asia	Household air pollution from solid fuels	822.2946	High fasting plasma glucose	615.2128	17
Central Asia	Diet low in legumes	741.6522	Diet low in legumes	535.0943	18
Central Asia	Diet low in polyunsaturated fatty acids	632.9581	Diet low in polyunsaturated fatty acids	464.3646	19
Central Asia	Diet low in fruits	568.7273	Non-optimal temperature	427.4956	20
Central Asia	Diet high in sodium	540.0702	Low temperature	369.5257	21
Central Asia	Diet low in fiber	527.2064	Diet high in sodium	294.7872	22
Central Asia	Non-optimal temperature	510.3187	Secondhand smoke	286.2379	23
Central Asia	Low temperature	474.9255	Diet low in fiber	260.0724	24
Central Asia	High fasting plasma glucose	417.8766	Diet low in fruits	258.2087	25
Central Asia	Secondhand smoke	401.3591	Household air pollution from solid fuels	242.541	26
Central Asia	Diet high in red meat	222.9891	Diet high in red meat	186.9166	27

Central Asia	Other environmental risks	210.334	Other environmental risks	179.0072	28
Central Asia	Lead exposure	210.334	Lead exposure	179.0072	29
Central Asia	Diet low in vegetables	156.4601	High temperature	63.34029	30
Central Asia	Diet high in trans fatty acids	83.48908	Low physical activity	59.70066	31
Central Asia	Low physical activity	75.95815	Diet high in processed meat	41.75704	32
Central Asia	Diet high in processed meat	57.53904	Diet high in trans fatty acids	35.50362	33
Central Asia	High temperature	38.82385	Diet low in vegetables	32.87768	34
Central Asia	Diet high in sugar-sweetened beverages	4.953517	Diet high in sugar-sweetened beverages	4.324261	35
Central Asia	Alcohol use	-101.2	Alcohol use	-50.7087	36
Central Europe	Metabolic risks	4319.173	Metabolic risks	2043.314	1
Central Europe	Behavioral risks	3634.91	Behavioral risks	1526.244	2
Central Europe	High systolic blood pressure	3080.996	High systolic blood pressure	1407.687	3
Central Europe	Dietary risks	3037.746	Dietary risks	1281.679	4
Central Europe	High LDL cholesterol	2119.325	High LDL cholesterol	946.6655	5
Central Europe	Environmental/occupational risks	1901.922	Environmental/occupational risks	654.219	6
Central Europe	Tobacco	1652.685	Tobacco	575.6832	7
Central Europe	Air pollution	1545.183	Smoking	486.3591	8
Central Europe	Particulate matter pollution	1545.183	Air pollution	444.3587	9
Central Europe	Smoking	1388.08	Particulate matter pollution	444.3587	10
Central Europe	Ambient particulate matter pollution	1148.484	High body-mass index	428.8984	11
Central Europe	Diet low in whole grains	917.6203	Ambient particulate matter pollution	415.656	12
Central Europe	Diet high in sodium	786.789	High fasting plasma glucose	411.533	13
Central Europe	High body-mass index	767.9007	Diet low in whole grains	359.6347	14
Central Europe	Diet low in nuts and seeds	765.9488	Diet high in sodium	351.0223	15
Central Europe	Kidney dysfunction	628.1309	Kidney dysfunction	306.7327	16
Central Europe	Diet low in seafood omega-3 fatty acids	625.2591	Diet low in nuts and seeds	259.5581	17
Central Europe	High fasting plasma glucose	574.9805	Diet low in seafood omega-3 fatty acids	228.7111	18
Central Europe	Diet low in polyunsaturated fatty acids	511.2269	Diet low in polyunsaturated fatty acids	214.3335	19
Central Europe	Household air pollution from solid fuels	396.2343	Non-optimal temperature	169.6402	20
Central Europe	Secondhand smoke	389.7009	Low temperature	160.9604	21
Central Europe	Diet low in legumes	385.7222	Diet low in legumes	145.0663	22
Central Europe	Diet low in fruits	365.1381	Diet low in fruits	125.6956	23

Central Europe	Non-optimal temperature	330.8711	Secondhand smoke	120.2322	24
Central Europe	Low temperature	322.8628	Diet low in fiber	116.5628	25
Central Europe	Diet low in fiber	276.7428	Diet high in red meat	112.5334	26
Central Europe	Diet high in red meat	240.0669	Other environmental risks	92.98396	27
Central Europe	Other environmental risks	190.2147	Lead exposure	92.98396	28
Central Europe	Lead exposure	190.2147	Low physical activity	50.33709	29
Central Europe	Diet low in vegetables	137.5543	Diet low in vegetables	38.8304	30
Central Europe	Low physical activity	92.52341	Diet high in processed meat	36.07102	31
Central Europe	Diet high in trans fatty acids	45.62119	Household air pollution from solid fuels	28.59244	32
Central Europe	Diet high in processed meat	42.96193	High temperature	9.397654	33
Central Europe	High temperature	8.645724	Diet high in sugar-sweetened beverages	4.679302	34
Central Europe	Diet high in sugar-sweetened beverages	7.77264	Diet high in trans fatty acids	3.549902	35
Central Europe	Alcohol use	-174.459	Alcohol use	-81.4492	36
Central Latin America	Metabolic risks	1894.238	Metabolic risks	1624.18	1
Central Latin America	Behavioral risks	1360.182	High systolic blood pressure	1014.417	2
Central Latin America	High systolic blood pressure	1165.942	Behavioral risks	997.1083	3
Central Latin America	Dietary risks	1128.45	Dietary risks	867.7398	4
Central Latin America	Environmental/occupational risks	942.3679	High LDL cholesterol	734.4371	5
Central Latin America	High LDL cholesterol	858.3625	Environmental/occupational risks	534.9502	6
Central Latin America	Air pollution	736.9929	High body-mass index	380.9291	7
Central Latin America	Particulate matter pollution	736.9929	Air pollution	341.5426	8
Central Latin America	Ambient particulate matter pollution	500.0864	Particulate matter pollution	341.5426	9
Central Latin America	Tobacco	477.2445	Kidney dysfunction	334.9183	10
Central Latin America	Smoking	409.3355	High fasting plasma glucose	330.5358	11
Central Latin America	Kidney dysfunction	399.3165	Ambient particulate matter pollution	271.364	12
Central Latin America	High fasting plasma glucose	333.8697	Tobacco	257.5092	13
Central Latin America	High body-mass index	314.805	Smoking	219.3633	14
Central Latin America	Diet low in seafood omega-3 fatty acids	279.7669	Diet low in nuts and seeds	197.146	15
Central Latin America	Diet low in nuts and seeds	252.8454	Diet low in seafood omega-3 fatty acids	194.7427	16
Central Latin America	Household air pollution from solid fuels	236.6875	Diet low in whole grains	187.3598	17
Central Latin America	Diet low in whole grains	235.863	Diet low in polyunsaturated fatty acids	165.3865	18
Central Latin America	Diet low in polyunsaturated fatty acids	205.568	Lead exposure	151.5059	19

Central Latin America	Lead exposure	202.1421	Other environmental risks	151.5059	20
Central Latin America	Other environmental risks	202.1421	Diet high in sodium	138.8645	21
Central Latin America	Diet high in sodium	179.8781	Non-optimal temperature	88.95481	22
Central Latin America	Diet low in fiber	126.2771	Diet low in vegetables	85.12891	23
Central Latin America	Diet low in vegetables	113.7549	Low temperature	82.30576	24
Central Latin America	Low temperature	106.5717	Diet low in fiber	81.68393	25
Central Latin America	Non-optimal temperature	102.6282	Diet low in fruits	74.27572	26
Central Latin America	Diet low in fruits	102.3133	Household air pollution from solid fuels	70.08785	27
Central Latin America	Diet low in legumes	93.70782	Diet high in red meat	61.55267	28
Central Latin America	Secondhand smoke	83.70199	Diet low in legumes	50.47811	29
Central Latin America	Diet high in trans fatty acids	74.31438	Secondhand smoke	44.08904	30
Central Latin America	Diet high in red meat	60.41387	Diet high in trans fatty acids	32.94545	31
Central Latin America	Low physical activity	27.97538	Low physical activity	22.13297	32
Central Latin America	Diet high in sugar-sweetened beverages	3.776434	High temperature	6.978885	33
Central Latin America	Diet high in processed meat	3.603382	Diet high in sugar-sweetened beverages	4.385761	34
Central Latin America	High temperature	-3.98639	Diet high in processed meat	4.082575	35
Central Latin America	Alcohol use	-55.6885	Alcohol use	-38.2485	36
Central Sub-Saharan Africa	Metabolic risks	2155.499	Metabolic risks	1861.601	1
Central Sub-Saharan Africa	High systolic blood pressure	1529.762	High systolic blood pressure	1249.443	2
Central Sub-Saharan Africa	Environmental/occupational risks	1379.548	Behavioral risks	1197.069	3
Central Sub-Saharan Africa	Behavioral risks	1353.686	Environmental/occupational risks	1111.995	4
Central Sub-Saharan Africa	Air pollution	1268.265	Dietary risks	1082.691	5
Central Sub-Saharan Africa	Particulate matter pollution	1268.265	Air pollution	1001.594	6
Central Sub-Saharan Africa	Dietary risks	1209.536	Particulate matter pollution	1001.594	7
Central Sub-Saharan Africa	Household air pollution from solid fuels	1109.44	Household air pollution from solid fuels	804.4101	8
Central Sub-Saharan Africa	High LDL cholesterol	781.3474	High LDL cholesterol	674.0984	9
Central Sub-Saharan Africa	Kidney dysfunction	489.994	Kidney dysfunction	427.3091	10
Central Sub-Saharan Africa	Diet low in vegetables	325.2413	High fasting plasma glucose	347.1416	11
Central Sub-Saharan Africa	High fasting plasma glucose	320.1425	Diet low in whole grains	283.227	12
Central Sub-Saharan Africa	Tobacco	311.7656	Diet low in vegetables	249.401	13
Central Sub-Saharan Africa	Diet low in whole grains	310.3955	Diet low in polyunsaturated fatty acids	247.6732	14
Central Sub-Saharan Africa	Diet low in polyunsaturated fatty acids	289.12	Tobacco	243.1078	15

Central Sub-Saharan Africa	Diet low in seafood omega-3 fatty acids	263.924	Diet low in seafood omega-3 fatty acids	238.8878	16
Central Sub-Saharan Africa	Smoking	260.7595	High body-mass index	229.7312	17
Central Sub-Saharan Africa	Diet low in fruits	206.4286	Diet low in fruits	224.9776	18
Central Sub-Saharan Africa	Other environmental risks	163.6435	Smoking	203.9487	19
Central Sub-Saharan Africa	Lead exposure	163.6435	Ambient particulate matter pollution	197.0972	20
Central Sub-Saharan Africa	Diet low in legumes	159.8559	Other environmental risks	156.0519	21
Central Sub-Saharan Africa	Ambient particulate matter pollution	158.7378	Lead exposure	156.0519	22
Central Sub-Saharan Africa	High body-mass index	126.0726	Diet low in legumes	146.5852	23
Central Sub-Saharan Africa	Diet low in fiber	106.126	Diet low in fiber	134.4973	24
Central Sub-Saharan Africa	Diet low in nuts and seeds	69.80027	Diet low in nuts and seeds	82.63752	25
Central Sub-Saharan Africa	Secondhand smoke	57.193	Diet high in sodium	45.34314	26
Central Sub-Saharan Africa	Low temperature	57.07239	Secondhand smoke	44.10156	27
Central Sub-Saharan Africa	Diet high in sodium	45.70925	Low temperature	40.1513	28
Central Sub-Saharan Africa	Non-optimal temperature	41.89915	Non-optimal temperature	34.88416	29
Central Sub-Saharan Africa	Low physical activity	36.04051	Low physical activity	31.88149	30
Central Sub-Saharan Africa	Diet high in red meat	7.430536	Diet high in red meat	9.183256	31
Central Sub-Saharan Africa	Diet high in processed meat	4.79102	Diet high in processed meat	3.715798	32
Central Sub-Saharan Africa	Diet high in sugar-sweetened beverages	1.307675	Diet high in sugar-sweetened beverages	0.963182	33
Central Sub-Saharan Africa	Diet high in trans fatty acids	1.22135	Diet high in trans fatty acids	0.476181	34
Central Sub-Saharan Africa	High temperature	-15.4742	High temperature	-5.34706	35
Central Sub-Saharan Africa	Alcohol use	-77.0826	Alcohol use	-59.2919	36
East Asia	Metabolic risks	1234.903	Metabolic risks	1378.396	1
East Asia	Behavioral risks	1196.403	Behavioral risks	1098.164	2
East Asia	Dietary risks	952.4395	High systolic blood pressure	910.1902	3
East Asia	Environmental/occupational risks	911.7021	Dietary risks	816.0485	4
East Asia	Air pollution	773.4508	Environmental/occupational risks	805.2054	5
East Asia	Particulate matter pollution	773.4508	Air pollution	635.6762	6
East Asia	High systolic blood pressure	713.583	Particulate matter pollution	635.6762	7
East Asia	Household air pollution from solid fuels	604.6752	High LDL cholesterol	585.2206	8
East Asia	Tobacco	572.5046	Tobacco	535.3468	9
East Asia	High LDL cholesterol	572.3317	Ambient particulate matter pollution	519.4749	10
East Asia	Smoking	435.682	Smoking	417.5418	11

East Asia	Diet high in sodium	263.3562	Diet high in sodium	256.4917	12
East Asia	Diet low in whole grains	243.2248	Diet low in whole grains	246.7908	13
East Asia	Kidney dysfunction	238.2558	High fasting plasma glucose	225.888	14
East Asia	Diet low in nuts and seeds	206.3248	Kidney dysfunction	216.2662	15
East Asia	Diet low in seafood omega-3 fatty acids	188.036	High body-mass index	172.7685	16
East Asia	High fasting plasma glucose	182.2887	Diet low in polyunsaturated fatty acids	169.2717	17
East Asia	Diet low in polyunsaturated fatty acids	182.2222	Secondhand smoke	148.2795	18
East Asia	Diet low in fruits	181.9804	Non-optimal temperature	139.4139	19
East Asia	Secondhand smoke	171.4598	Other environmental risks	131.2357	20
East Asia	Ambient particulate matter pollution	168.7393	Lead exposure	131.2357	21
East Asia	Diet low in fiber	146.4906	Low temperature	126.6957	22
East Asia	Non-optimal temperature	137.2479	Diet low in nuts and seeds	125.618	23
East Asia	Low temperature	128.6681	Household air pollution from solid fuels	116.0603	24
East Asia	Other environmental risks	119.4154	Diet low in fruits	84.94924	25
East Asia	Lead exposure	119.4154	Diet high in red meat	83.70385	26
East Asia	High body-mass index	79.12833	Diet low in seafood omega-3 fatty acids	77.72721	27
East Asia	Diet low in legumes	75.19351	Diet low in fiber	67.85166	28
East Asia	Diet low in vegetables	61.99345	Diet low in legumes	57.71391	29
East Asia	Low physical activity	40.23013	Low physical activity	45.01695	30
East Asia	Diet high in red meat	39.8798	High temperature	13.74936	31
East Asia	Diet high in trans fatty acids	11.23918	Diet low in vegetables	8.986972	32
East Asia	High temperature	9.344088	Diet high in trans fatty acids	5.085408	33
East Asia	Diet high in processed meat	0.816267	Diet high in processed meat	1.815856	34
East Asia	Diet high in sugar-sweetened beverages	0.355257	Diet high in sugar-sweetened beverages	1.037782	35
East Asia	Alcohol use	-43.1413	Alcohol use	-39.9485	36
Eastern Europe	Metabolic risks	4866.948	Metabolic risks	3872.39	1
Eastern Europe	Behavioral risks	3945.287	Behavioral risks	2833.295	2
Eastern Europe	Dietary risks	3420.695	High systolic blood pressure	2672.557	3
Eastern Europe	High systolic blood pressure	3412.084	Dietary risks	2334.137	4
Eastern Europe	High LDL cholesterol	2421.361	High LDL cholesterol	1866.185	5
Eastern Europe	Environmental/occupational risks	1896.715	Tobacco	1129.561	6
Eastern Europe	Air pollution	1524.415	Smoking	970.3273	7

Eastern Europe	Particulate matter pollution	1524.415	Environmental/occupational risks	969.2575	8
Eastern Europe	Tobacco	1485.103	High body-mass index	844.7328	9
Eastern Europe	Ambient particulate matter pollution	1415.393	Diet low in whole grains	824.144	10
Eastern Europe	Smoking	1225.946	Kidney dysfunction	713.2644	11
Eastern Europe	Diet low in whole grains	1218.921	Air pollution	595.3387	12
Eastern Europe	Kidney dysfunction	910.6327	Particulate matter pollution	595.3387	13
Eastern Europe	Diet low in nuts and seeds	885.0604	Ambient particulate matter pollution	566.4684	14
Eastern Europe	High body-mass index	790.5441	Diet low in nuts and seeds	540.242	15
Eastern Europe	Diet high in processed meat	599.466	High fasting plasma glucose	490.9979	16
Eastern Europe	Diet low in polyunsaturated fatty acids	591.4592	Diet low in polyunsaturated fatty acids	445.0248	17
Eastern Europe	Diet low in fruits	585.8803	Diet low in fruits	362.8427	18
Eastern Europe	Diet low in legumes	570.1779	Diet low in legumes	362.6093	19
Eastern Europe	Diet low in seafood omega-3 fatty acids	423.9095	Non-optimal temperature	316.1388	20
Eastern Europe	High fasting plasma glucose	403.4771	Low temperature	303.0767	21
Eastern Europe	Non-optimal temperature	374.771	Diet low in seafood omega-3 fatty acids	272.1946	22
Eastern Europe	Low temperature	369.5551	Diet high in sodium	240.0491	23
Eastern Europe	Secondhand smoke	322.3527	Diet low in fiber	226.0349	24
Eastern Europe	Diet high in red meat	308.2769	Secondhand smoke	209.7816	25
Eastern Europe	Diet high in sodium	303.1014	Diet high in red meat	164.8694	26
Eastern Europe	Diet low in fiber	288.2177	Diet high in processed meat	139.418	27
Eastern Europe	Diet low in vegetables	167.8889	Diet low in vegetables	121.6685	28
Eastern Europe	Other environmental risks	132.8163	Other environmental risks	120.103	29
Eastern Europe	Lead exposure	132.8163	Lead exposure	120.103	30
Eastern Europe	Household air pollution from solid fuels	108.6632	Low physical activity	75.631	31
Eastern Europe	Low physical activity	96.57398	Household air pollution from solid fuels	28.72319	32
Eastern Europe	Diet high in trans fatty acids	27.47011	High temperature	14.07554	33
Eastern Europe	High temperature	5.619136	Diet high in sugar-sweetened beverages	3.556841	34
Eastern Europe	Diet high in sugar-sweetened beverages	3.790653	Diet high in trans fatty acids	2.718378	35
Eastern Europe	Alcohol use	-159.721	Alcohol use	-108.951	36
Eastern Sub-Saharan Africa	Metabolic risks	958.5122	Metabolic risks	1074.285	1
Eastern Sub-Saharan Africa	Behavioral risks	907.5127	Behavioral risks	795.1455	2
Eastern Sub-Saharan Africa	Dietary risks	822.2536	Environmental/occupational risks	771.2466	3

Eastern Sub-Saharan Africa	Environmental/occupational risks	805.461	High systolic blood pressure	740.448	4
Eastern Sub-Saharan Africa	Air pollution	704.8786	Dietary risks	714.6881	5
Eastern Sub-Saharan Africa	Particulate matter pollution	704.8786	Air pollution	682.8124	6
Eastern Sub-Saharan Africa	Household air pollution from solid fuels	648.8727	Particulate matter pollution	682.8124	7
Eastern Sub-Saharan Africa	High systolic blood pressure	587.7006	Household air pollution from solid fuels	607.075	8
Eastern Sub-Saharan Africa	High LDL cholesterol	392.8566	High LDL cholesterol	414.6689	9
Eastern Sub-Saharan Africa	Diet low in seafood omega-3 fatty acids	223.1466	Diet low in seafood omega-3 fatty acids	184.7274	10
Eastern Sub-Saharan Africa	Diet low in whole grains	209.9269	Diet low in whole grains	178.9731	11
Eastern Sub-Saharan Africa	Tobacco	205.688	Tobacco	175.7168	12
Eastern Sub-Saharan Africa	Smoking	175.264	Diet low in polyunsaturated fatty acids	160.5745	13
Eastern Sub-Saharan Africa	Diet low in fruits	170.2649	Kidney dysfunction	157.9404	14
Eastern Sub-Saharan Africa	Diet low in polyunsaturated fatty acids	168.861	Smoking	152.6925	15
Eastern Sub-Saharan Africa	Diet low in nuts and seeds	162.4004	Diet low in fruits	142.3919	16
Eastern Sub-Saharan Africa	Kidney dysfunction	156.4702	High fasting plasma glucose	128.5637	17
Eastern Sub-Saharan Africa	Diet low in vegetables	152.8424	High body-mass index	113.015	18
Eastern Sub-Saharan Africa	Other environmental risks	122.8828	Diet low in vegetables	111.7925	19
Eastern Sub-Saharan Africa	Lead exposure	122.8828	Other environmental risks	106.9607	20
Eastern Sub-Saharan Africa	Diet high in sodium	119.939	Lead exposure	106.9607	21
Eastern Sub-Saharan Africa	High fasting plasma glucose	95.67383	Diet low in nuts and seeds	105.7184	22
Eastern Sub-Saharan Africa	Non-optimal temperature	67.48428	Diet high in sodium	95.38548	23
Eastern Sub-Saharan Africa	Low temperature	67.16984	Ambient particulate matter pollution	75.69647	24
Eastern Sub-Saharan Africa	High body-mass index	62.49893	Non-optimal temperature	57.36964	25
Eastern Sub-Saharan Africa	Diet low in legumes	56.12876	Low temperature	55.20678	26
Eastern Sub-Saharan Africa	Ambient particulate matter pollution	55.96683	Diet low in legumes	46.2451	27
Eastern Sub-Saharan Africa	Diet low in fiber	47.11083	Diet low in fiber	35.38397	28
Eastern Sub-Saharan Africa	Secondhand smoke	34.92557	Secondhand smoke	26.29269	29
Eastern Sub-Saharan Africa	Low physical activity	8.409176	Low physical activity	8.619089	30
Eastern Sub-Saharan Africa	Diet high in processed meat	2.569366	Diet high in processed meat	2.76936	31
Eastern Sub-Saharan Africa	Diet high in red meat	2.040517	High temperature	2.237181	32
Eastern Sub-Saharan Africa	Diet high in trans fatty acids	0.78968	Diet high in red meat	1.290717	33
Eastern Sub-Saharan Africa	Diet high in sugar-sweetened beverages	0.571384	Diet high in sugar-sweetened beverages	0.780951	34
Eastern Sub-Saharan Africa	High temperature	0.325566	Diet high in trans fatty acids	0.359558	35

Eastern Sub-Saharan Africa	Alcohol use	-30.6387	Alcohol use	-32.7424	36
High-income Asia Pacific	Metabolic risks	932.1928	Metabolic risks	379.7469	1
High-income Asia Pacific	Behavioral risks	667.4329	Behavioral risks	265.2056	2
High-income Asia Pacific	High systolic blood pressure	658.7163	High systolic blood pressure	231.7616	3
High-income Asia Pacific	Dietary risks	491.8513	Dietary risks	206.809	4
High-income Asia Pacific	High LDL cholesterol	407.1413	High LDL cholesterol	185.3007	5
High-income Asia Pacific	Tobacco	344.623	Tobacco	119.4503	6
High-income Asia Pacific	Smoking	289.9477	Environmental/occupational risks	113.8414	7
High-income Asia Pacific	Environmental/occupational risks	274.2526	Smoking	104.9477	8
High-income Asia Pacific	Kidney dysfunction	176.1584	Air pollution	73.05316	9
High-income Asia Pacific	Air pollution	166.5447	Particulate matter pollution	73.05316	10
High-income Asia Pacific	Particulate matter pollution	166.5447	Ambient particulate matter pollution	73.01921	11
High-income Asia Pacific	Ambient particulate matter pollution	164.2606	High fasting plasma glucose	69.78329	12
High-income Asia Pacific	Diet high in sodium	159.1219	Kidney dysfunction	68.17361	13
High-income Asia Pacific	High fasting plasma glucose	153.987	Diet low in whole grains	56.65385	14
High-income Asia Pacific	Diet low in nuts and seeds	108.9272	Diet low in nuts and seeds	50.16548	15
High-income Asia Pacific	Diet low in whole grains	98.95437	Diet high in sodium	46.71473	16
High-income Asia Pacific	Diet low in polyunsaturated fatty acids	97.82504	High body-mass index	40.64474	17
High-income Asia Pacific	Non-optimal temperature	90.52291	Diet low in fiber	40.15065	18
High-income Asia Pacific	Diet low in fruits	88.52609	Diet low in fruits	36.79656	19
High-income Asia Pacific	Low temperature	85.5457	Diet low in polyunsaturated fatty acids	36.34866	20
High-income Asia Pacific	Secondhand smoke	72.99366	Non-optimal temperature	35.17571	21
High-income Asia Pacific	Diet low in fiber	69.97382	Low temperature	33.24829	22
High-income Asia Pacific	High body-mass index	60.02053	Secondhand smoke	19.76668	23
High-income Asia Pacific	Other environmental risks	38.40106	Other environmental risks	13.88106	24
High-income Asia Pacific	Lead exposure	38.40106	Lead exposure	13.88106	25
High-income Asia Pacific	Diet low in legumes	26.69115	Diet low in legumes	13.79831	26
High-income Asia Pacific	Low physical activity	25.2941	Diet high in red meat	12.1902	27
High-income Asia Pacific	Diet low in vegetables	24.74902	Diet high in processed meat	12.10102	28
High-income Asia Pacific	Diet high in processed meat	19.51887	Low physical activity	10.08504	29
High-income Asia Pacific	Diet high in red meat	13.01403	Diet low in vegetables	8.083695	30
High-income Asia Pacific	Diet high in trans fatty acids	8.591667	Diet high in trans fatty acids	4.23637	31

High-income Asia Pacific	Diet low in seafood omega-3 fatty acids	6.346592	High temperature	2.078792	32
High-income Asia Pacific	High temperature	5.420905	Diet high in sugar-sweetened beverages	0.699769	33
High-income Asia Pacific	Household air pollution from solid fuels	2.253527	Diet low in seafood omega-3 fatty acids	0.515344	34
High-income Asia Pacific	Diet high in sugar-sweetened beverages	1.126353	Household air pollution from solid fuels	0.031103	35
High-income Asia Pacific	Alcohol use	-54.443	Alcohol use	-23.1597	36
High-income North America	Metabolic risks	2736.294	Metabolic risks	1150.273	1
High-income North America	Behavioral risks	2061.05	Behavioral risks	804.501	2
High-income North America	High systolic blood pressure	1757.64	Dietary risks	633.3169	3
High-income North America	Dietary risks	1521.587	High systolic blood pressure	628.6719	4
High-income North America	High LDL cholesterol	1398.393	High LDL cholesterol	515.4291	5
High-income North America	Tobacco	1059.924	Tobacco	345.8333	6
High-income North America	Smoking	955.2695	High body-mass index	316.7504	7
High-income North America	Environmental/occupational risks	748.8517	Smoking	310.6432	8
High-income North America	High body-mass index	520.2505	High fasting plasma glucose	295.1961	9
High-income North America	Kidney dysfunction	488.4571	Kidney dysfunction	222.8095	10
High-income North America	Air pollution	438.3165	Environmental/occupational risks	203.1193	11
High-income North America	Particulate matter pollution	438.3165	Diet low in whole grains	197.5697	12
High-income North America	Ambient particulate matter pollution	437.9897	Diet high in processed meat	127.1771	13
High-income North America	Diet low in whole grains	419.546	Diet low in seafood omega-3 fatty acids	125.9406	14
High-income North America	High fasting plasma glucose	410.4786	Non-optimal temperature	104.1541	15
High-income North America	Diet high in trans fatty acids	306.744	Low temperature	95.43299	16
High-income North America	Diet low in fiber	297.0877	Diet high in red meat	86.20844	17
High-income North America	Diet low in seafood omega-3 fatty acids	252.099	Diet low in legumes	86.07967	18
High-income North America	Non-optimal temperature	246.2369	Diet low in fiber	85.81153	19
High-income North America	Low temperature	230.1773	Diet low in fruits	71.51954	20
High-income North America	Diet high in red meat	191.6872	Diet high in sodium	70.68454	21
High-income North America	Diet low in fruits	191.5287	Air pollution	68.41025	22
High-income North America	Diet low in legumes	176.1237	Particulate matter pollution	68.41025	23
High-income North America	Diet high in processed meat	175.2305	Ambient particulate matter pollution	68.38409	24
High-income North America	Secondhand smoke	167.0396	Secondhand smoke	49.31442	25
High-income North America	Other environmental risks	120.9038	Diet low in vegetables	45.61066	26
High-income North America	Lead exposure	120.9038	Other environmental risks	40.19127	27

High-income North America	Diet low in polyunsaturated fatty acids	117.6909	Lead exposure	40.19127	28
High-income North America	Diet high in sodium	105.9519	Diet low in polyunsaturated fatty acids	24.38582	29
High-income North America	Diet low in nuts and seeds	105.5723	Diet low in nuts and seeds	22.55591	30
High-income North America	Diet low in vegetables	98.13508	Low physical activity	21.15171	31
High-income North America	Low physical activity	47.10865	High temperature	9.406753	32
High-income North America	High temperature	17.39545	Diet high in sugar-sweetened beverages	5.529184	33
High-income North America	Diet high in sugar-sweetened beverages	6.602268	Household air pollution from solid fuels	0.021049	34
High-income North America	Household air pollution from solid fuels	0.300501	Diet high in trans fatty acids	0.012999	35
High-income North America	Alcohol use	-44.3792	Alcohol use	-40.5866	36
North Africa and Middle East	Metabolic risks	4372.529	Metabolic risks	3227.559	1
North Africa and Middle East	Behavioral risks	3657.657	Behavioral risks	2303.041	2
North Africa and Middle East	Dietary risks	2854.672	High systolic blood pressure	2051.54	3
North Africa and Middle East	High systolic blood pressure	2745.952	Dietary risks	1756.149	4
North Africa and Middle East	Environmental/occupational risks	2523.814	Environmental/occupational risks	1723.843	5
North Africa and Middle East	High LDL cholesterol	2055.248	High LDL cholesterol	1442.56	6
North Africa and Middle East	Air pollution	1863.487	Air pollution	1261.481	7
North Africa and Middle East	Particulate matter pollution	1863.487	Particulate matter pollution	1261.481	8
North Africa and Middle East	Tobacco	1567.273	Ambient particulate matter pollution	1129.043	9
North Africa and Middle East	Diet low in whole grains	1233.407	Tobacco	943.5386	10
North Africa and Middle East	Ambient particulate matter pollution	1218.586	High body-mass index	824.6271	11
North Africa and Middle East	Smoking	1191.454	Diet low in whole grains	806.1867	12
North Africa and Middle East	Diet low in seafood omega-3 fatty acids	804.8426	Smoking	716.3685	13
North Africa and Middle East	Kidney dysfunction	798.1473	High fasting plasma glucose	704.9733	14
North Africa and Middle East	High body-mass index	728.662	Kidney dysfunction	563.5001	15
North Africa and Middle East	Household air pollution from solid fuels	644.5445	Diet low in seafood omega-3 fatty acids	420.8404	16
North Africa and Middle East	Diet low in nuts and seeds	612.5252	Non-optimal temperature	380.1013	17
North Africa and Middle East	High fasting plasma glucose	590.9812	Diet low in polyunsaturated fatty acids	363.9698	18
North Africa and Middle East	Diet low in polyunsaturated fatty acids	558.8434	Other environmental risks	335.8378	19
North Africa and Middle East	Non-optimal temperature	521.6004	Lead exposure	335.8378	20
North Africa and Middle East	Other environmental risks	515.9543	Diet low in nuts and seeds	312.7224	21
North Africa and Middle East	Lead exposure	515.9543	Secondhand smoke	279.8899	22
North Africa and Middle East	Secondhand smoke	477.0544	Low temperature	263.2206	23

North Africa and Middle East	Low temperature	427.3605	Household air pollution from solid fuels	132.3816	24
North Africa and Middle East	Diet low in legumes	262.1555	High temperature	125.7947	25
North Africa and Middle East	Diet low in fruits	254.1171	Diet high in trans fatty acids	125.6728	26
North Africa and Middle East	Diet low in fiber	199.3189	Low physical activity	125.4283	27
North Africa and Middle East	Diet high in trans fatty acids	189.8646	Diet low in legumes	125.2541	28
North Africa and Middle East	Low physical activity	178.2344	Diet low in fruits	115.6594	29
North Africa and Middle East	Diet low in vegetables	168.8476	Diet low in fiber	113.1409	30
North Africa and Middle East	High temperature	102.9961	Diet low in vegetables	80.96469	31
North Africa and Middle East	Diet high in sodium	81.84234	Diet high in sodium	56.61798	32
North Africa and Middle East	Diet high in red meat	17.9789	Diet high in red meat	13.84154	33
North Africa and Middle East	Diet high in sugar-sweetened beverages	4.025336	Diet high in sugar-sweetened beverages	4.403525	34
North Africa and Middle East	Diet high in processed meat	3.809609	Diet high in processed meat	3.524872	35
North Africa and Middle East	Alcohol use	-9.04876	Alcohol use	-4.35835	36
Oceania	Metabolic risks	3037.988	Metabolic risks	2991.31	1
Oceania	Behavioral risks	2669.146	Behavioral risks	2409.548	2
Oceania	Dietary risks	2154.393	Dietary risks	1949.749	3
Oceania	Environmental/occupational risks	1806.864	High systolic blood pressure	1604.043	4
Oceania	Air pollution	1682.143	Environmental/occupational risks	1571.61	5
Oceania	Particulate matter pollution	1682.143	High LDL cholesterol	1497.485	6
Oceania	High LDL cholesterol	1618.128	Air pollution	1456.397	7
Oceania	Household air pollution from solid fuels	1503.523	Particulate matter pollution	1456.397	8
Oceania	High systolic blood pressure	1415.504	Household air pollution from solid fuels	1220.519	9
Oceania	Tobacco	1091.769	Tobacco	956.7609	10
Oceania	Smoking	940.007	Smoking	803.8434	11
Oceania	Diet low in whole grains	820.0263	Diet low in whole grains	761.7688	12
Oceania	High fasting plasma glucose	608.125	High fasting plasma glucose	696.8704	13
Oceania	Kidney dysfunction	562.5047	High body-mass index	608.8088	14
Oceania	High body-mass index	514.3719	Kidney dysfunction	541.0778	15
Oceania	Diet low in fruits	494.061	Diet low in polyunsaturated fatty acids	420.8922	16
Oceania	Diet low in polyunsaturated fatty acids	413.5256	Diet low in fruits	402.4962	17
Oceania	Diet high in sodium	281.3965	Diet high in sodium	277.7904	18
Oceania	Diet low in vegetables	281.0327	Diet low in vegetables	236.5035	19

Oceania	Diet low in seafood omega-3 fatty acids	246.804	Ambient particulate matter pollution	235.7772	20
Oceania	Diet low in nuts and seeds	240.6818	Diet low in nuts and seeds	212.364	21
Oceania	Diet low in legumes	232.2303	Diet low in seafood omega-3 fatty acids	211.9113	22
Oceania	Secondhand smoke	197.3298	Diet low in legumes	207.4297	23
Oceania	Ambient particulate matter pollution	178.3591	Secondhand smoke	195.0308	24
Oceania	Low temperature	113.3643	Other environmental risks	98.86452	25
Oceania	Non-optimal temperature	109.617	Lead exposure	98.86452	26
Oceania	Other environmental risks	107.7476	Low temperature	95.73113	27
Oceania	Lead exposure	107.7476	Non-optimal temperature	94.8011	28
Oceania	Diet high in red meat	105.0103	Diet high in red meat	67.53519	29
Oceania	Diet low in fiber	81.48827	Low physical activity	63.30955	30
Oceania	Low physical activity	72.64965	Diet low in fiber	26.97416	31
Oceania	Diet high in trans fatty acids	3.185251	Diet high in sugar-sweetened beverages	2.737413	32
Oceania	Diet high in sugar-sweetened beverages	2.746911	Diet high in processed meat	2.557261	33
Oceania	Diet high in processed meat	2.589888	Diet high in trans fatty acids	1.122959	34
Oceania	High temperature	-3.82169	High temperature	-0.94345	35
Oceania	Alcohol use	-33.7557	Alcohol use	-29.3981	36
South Asia	Metabolic risks	2341.173	Metabolic risks	2520.539	1
South Asia	Behavioral risks	2222.112	Behavioral risks	2076.126	2
South Asia	Dietary risks	1891.819	Dietary risks	1779.656	3
South Asia	Environmental/occupational risks	1721.85	Environmental/occupational risks	1646.828	4
South Asia	Air pollution	1445.215	High systolic blood pressure	1587.155	5
South Asia	Particulate matter pollution	1445.215	Air pollution	1343.271	6
South Asia	High systolic blood pressure	1432.201	Particulate matter pollution	1343.271	7
South Asia	Household air pollution from solid fuels	1156.796	High LDL cholesterol	1089.407	8
South Asia	High LDL cholesterol	1059.33	Ambient particulate matter pollution	703.1656	9
South Asia	Tobacco	807.2863	Household air pollution from solid fuels	639.9038	10
South Asia	Smoking	671.687	Tobacco	631.8401	11
South Asia	Diet low in fruits	552.2605	Diet low in fruits	532.2518	12
South Asia	Diet low in seafood omega-3 fatty acids	530.5995	Smoking	514.11	13
South Asia	Diet low in nuts and seeds	497.3461	High fasting plasma glucose	497.7924	14
South Asia	Kidney dysfunction	476.8594	Kidney dysfunction	487.4149	15

South Asia	Diet low in whole grains	440.9277	Diet low in whole grains	440.151	16
South Asia	Diet low in polyunsaturated fatty acids	376.1758	Diet low in nuts and seeds	428.9683	17
South Asia	Diet low in fiber	359.7111	Diet low in seafood omega-3 fatty acids	391.0697	18
South Asia	High fasting plasma glucose	340.2817	Diet low in polyunsaturated fatty acids	367.7259	19
South Asia	Other environmental risks	296.1025	Other environmental risks	293.6374	20
South Asia	Lead exposure	296.1025	Lead exposure	293.6374	21
South Asia	Ambient particulate matter pollution	288.2422	Diet low in fiber	253.8451	22
South Asia	Non-optimal temperature	223.4654	Non-optimal temperature	241.7929	23
South Asia	Diet low in vegetables	196.4834	High body-mass index	241.269	24
South Asia	Diet low in legumes	181.0242	Diet high in sodium	182.6035	25
South Asia	Diet high in trans fatty acids	170.7025	Diet low in vegetables	158.8863	26
South Asia	Secondhand smoke	167.2443	Low temperature	157.4435	27
South Asia	Low temperature	160.6423	Diet low in legumes	153.6	28
South Asia	Diet high in sodium	155.0845	Secondhand smoke	140.9048	29
South Asia	High body-mass index	107.3108	Diet high in trans fatty acids	93.74593	30
South Asia	Low physical activity	70.87951	High temperature	89.90698	31
South Asia	High temperature	67.50798	Low physical activity	72.2461	32
South Asia	Diet high in processed meat	4.43611	Diet high in processed meat	5.521579	33
South Asia	Diet high in sugar-sweetened beverages	1.675031	Diet high in sugar-sweetened beverages	2.631437	34
South Asia	Diet high in red meat	-0.90495	Diet high in red meat	0.618849	35
South Asia	Alcohol use	-30.8536	Alcohol use	-30.6823	36
Southeast Asia	Metabolic risks	1925.538	Metabolic risks	1900.462	1
Southeast Asia	Behavioral risks	1588.836	Behavioral risks	1316.957	2
Southeast Asia	Dietary risks	1233.727	High systolic blood pressure	1282.033	3
Southeast Asia	High systolic blood pressure	1232.753	Dietary risks	956.8203	4
Southeast Asia	Environmental/occupational risks	1143.162	High LDL cholesterol	844.8869	5
Southeast Asia	Air pollution	1048.152	Environmental/occupational risks	800.3739	6
Southeast Asia	Particulate matter pollution	1048.152	Air pollution	687.1727	7
Southeast Asia	High LDL cholesterol	903.7655	Particulate matter pollution	687.1727	8
Southeast Asia	Household air pollution from solid fuels	792.2252	Tobacco	599.0595	9
Southeast Asia	Tobacco	701.1135	Smoking	504.405	10
Southeast Asia	Smoking	584.004	Kidney dysfunction	403.8588	11

Southeast Asia	Diet low in fiber	434.548	Ambient particulate matter pollution	385.7298	12
Southeast Asia	Kidney dysfunction	417.9955	Diet low in fiber	327.2574	13
Southeast Asia	Diet high in sodium	326.258	Household air pollution from solid fuels	301.2938	14
Southeast Asia	Diet low in fruits	292.5001	High fasting plasma glucose	280.3256	15
Southeast Asia	Diet low in polyunsaturated fatty acids	280.2316	Diet low in polyunsaturated fatty acids	258.9667	16
Southeast Asia	Ambient particulate matter pollution	255.8642	Diet high in sodium	245.9287	17
Southeast Asia	Diet low in whole grains	236.9337	Diet low in whole grains	206.7088	18
Southeast Asia	High fasting plasma glucose	223.4858	High body-mass index	184.4335	19
Southeast Asia	Diet low in seafood omega-3 fatty acids	198.6321	Diet low in fruits	175.4416	20
Southeast Asia	Diet low in legumes	193.1095	Diet low in legumes	161.4647	21
Southeast Asia	Diet low in vegetables	156.3779	Other environmental risks	120.6204	22
Southeast Asia	Secondhand smoke	145.1854	Lead exposure	120.6204	23
Southeast Asia	Other environmental risks	127.8904	Secondhand smoke	115.2104	24
Southeast Asia	Lead exposure	127.8904	Diet low in vegetables	109.3143	25
Southeast Asia	High body-mass index	108.0052	Low physical activity	66.36546	26
Southeast Asia	Diet low in nuts and seeds	100.0928	Diet low in seafood omega-3 fatty acids	61.36655	27
Southeast Asia	Low physical activity	59.27882	Non-optimal temperature	42.32276	28
Southeast Asia	Non-optimal temperature	38.23742	Diet low in nuts and seeds	39.04079	29
Southeast Asia	Low temperature	36.56972	Low temperature	27.21892	30
Southeast Asia	High temperature	1.788491	Diet high in red meat	16.79395	31
Southeast Asia	Diet high in trans fatty acids	1.704444	High temperature	15.36511	32
Southeast Asia	Diet high in processed meat	1.144226	Diet high in sugar-sweetened beverages	1.74085	33
Southeast Asia	Diet high in sugar-sweetened beverages	1.004363	Diet high in processed meat	1.707491	34
Southeast Asia	Diet high in red meat	0.693194	Diet high in trans fatty acids	0.659195	35
Southeast Asia	Alcohol use	-30.0495	Alcohol use	-33.8055	36
Southern Latin America	Metabolic risks	2086.201	Metabolic risks	855.875	1
Southern Latin America	Behavioral risks	1855.991	Behavioral risks	654.3612	2
Southern Latin America	Dietary risks	1619.783	Dietary risks	570.4123	3
Southern Latin America	High systolic blood pressure	1226.74	High systolic blood pressure	546.4455	4
Southern Latin America	High LDL cholesterol	1045.065	High LDL cholesterol	413.2137	5
Southern Latin America	Environmental/occupational risks	838.463	Environmental/occupational risks	258.1555	6
Southern Latin America	Tobacco	718.9938	Tobacco	237.9202	7

Southern Latin America	Diet low in whole grains	609.5037	High body-mass index	208.3048	8
Southern Latin America	Smoking	604.5774	Smoking	204.0093	9
Southern Latin America	Air pollution	598.0289	Diet low in whole grains	196.0077	10
Southern Latin America	Particulate matter pollution	598.0289	Diet low in nuts and seeds	171.6706	11
Southern Latin America	Diet low in nuts and seeds	507.0446	Air pollution	165.3533	12
Southern Latin America	Ambient particulate matter pollution	457.4631	Particulate matter pollution	165.3533	13
Southern Latin America	High body-mass index	391.5217	Ambient particulate matter pollution	161.61	14
Southern Latin America	Diet low in seafood omega-3 fatty acids	331.1981	High fasting plasma glucose	159.7537	15
Southern Latin America	Diet low in fiber	288.0807	Kidney dysfunction	106.8983	16
Southern Latin America	Kidney dysfunction	283.0395	Diet low in seafood omega-3 fatty acids	99.66972	17
Southern Latin America	High fasting plasma glucose	278.3787	Diet low in fiber	94.03161	18
Southern Latin America	Diet low in polyunsaturated fatty acids	253.2755	Diet low in polyunsaturated fatty acids	88.95862	19
Southern Latin America	Diet low in legumes	249.8347	Diet low in legumes	79.89558	20
Southern Latin America	Non-optimal temperature	225.1289	Non-optimal temperature	75.78295	21
Southern Latin America	Low temperature	221.2595	Diet high in red meat	74.60049	22
Southern Latin America	Diet high in red meat	185.8371	Low temperature	74.5375	23
Southern Latin America	Diet high in sodium	169.4918	Diet high in sodium	66.67909	24
Southern Latin America	Secondhand smoke	157.9787	Secondhand smoke	45.66036	25
Southern Latin America	Household air pollution from solid fuels	140.362	Diet low in vegetables	38.21694	26
Southern Latin America	Diet low in fruits	137.0507	Other environmental risks	36.24203	27
Southern Latin America	Diet low in vegetables	115.0247	Lead exposure	36.24203	28
Southern Latin America	Other environmental risks	85.83929	Diet low in fruits	28.59267	29
Southern Latin America	Lead exposure	85.83929	Diet high in processed meat	19.79369	30
Southern Latin America	Diet high in processed meat	23.3276	Low physical activity	7.765052	31
Southern Latin America	Low physical activity	20.91064	Diet high in sugar-sweetened beverages	3.686932	32
Southern Latin America	Diet high in sugar-sweetened beverages	6.314918	Household air pollution from solid fuels	3.679835	33
Southern Latin America	Diet high in trans fatty acids	4.64275	High temperature	1.341568	34
Southern Latin America	High temperature	4.215917	Diet high in trans fatty acids	0.774036	35
Southern Latin America	Alcohol use	-108.908	Alcohol use	-46.8632	36
Southern Sub-Saharan Africa	Metabolic risks	1284.628	Metabolic risks	1367.934	1
Southern Sub-Saharan Africa	Behavioral risks	1047.238	Behavioral risks	965.222	2
Southern Sub-Saharan Africa	High systolic blood pressure	897.2665	High systolic blood pressure	957.4684	3

Southern Sub-Saharan Africa	Dietary risks	840.6942	Dietary risks	822.1013	4
Southern Sub-Saharan Africa	Environmental/occupational risks	647.4114	Environmental/occupational risks	578.8165	5
Southern Sub-Saharan Africa	High LDL cholesterol	532.3728	High LDL cholesterol	530.5379	6
Southern Sub-Saharan Africa	Air pollution	519.4448	Air pollution	438.9886	7
Southern Sub-Saharan Africa	Particulate matter pollution	519.4448	Particulate matter pollution	438.9886	8
Southern Sub-Saharan Africa	Tobacco	453.3318	Kidney dysfunction	303.5794	9
Southern Sub-Saharan Africa	Smoking	384.2783	High body-mass index	303.4582	10
Southern Sub-Saharan Africa	Kidney dysfunction	277.3564	Tobacco	284.3724	11
Southern Sub-Saharan Africa	Ambient particulate matter pollution	263.1451	Ambient particulate matter pollution	276.1876	12
Southern Sub-Saharan Africa	Household air pollution from solid fuels	256.2054	Smoking	235.9873	13
Southern Sub-Saharan Africa	Diet low in nuts and seeds	243.598	Diet low in fruits	219.1212	14
Southern Sub-Saharan Africa	Diet low in fruits	223.3599	Diet low in nuts and seeds	218.5836	15
Southern Sub-Saharan Africa	Diet low in seafood omega-3 fatty acids	221.0714	Diet low in seafood omega-3 fatty acids	214.7054	16
Southern Sub-Saharan Africa	High body-mass index	208.2024	High fasting plasma glucose	210.7234	17
Southern Sub-Saharan Africa	Diet low in polyunsaturated fatty acids	160.3284	Household air pollution from solid fuels	162.7091	18
Southern Sub-Saharan Africa	Diet low in whole grains	138.5459	Diet low in whole grains	140.0737	19
Southern Sub-Saharan Africa	High fasting plasma glucose	137.3821	Diet low in polyunsaturated fatty acids	129.9233	20
Southern Sub-Saharan Africa	Diet low in legumes	119.775	Non-optimal temperature	109.1469	21
Southern Sub-Saharan Africa	Non-optimal temperature	112.3009	Low temperature	108.449	22
Southern Sub-Saharan Africa	Low temperature	112.0776	Diet low in vegetables	101.1732	23
Southern Sub-Saharan Africa	Diet low in vegetables	101.4347	Diet low in legumes	99.42676	24
Southern Sub-Saharan Africa	Secondhand smoke	93.91317	Other environmental risks	86.0862	25
Southern Sub-Saharan Africa	Other environmental risks	81.86753	Lead exposure	86.0862	26
Southern Sub-Saharan Africa	Lead exposure	81.86753	Secondhand smoke	58.33845	27
Southern Sub-Saharan Africa	Low physical activity	54.38417	Low physical activity	56.87996	28
Southern Sub-Saharan Africa	Diet low in fiber	49.82613	Diet low in fiber	54.10878	29
Southern Sub-Saharan Africa	Diet high in red meat	47.89795	Diet high in red meat	46.84341	30
Southern Sub-Saharan Africa	Diet high in sodium	46.0101	Diet high in sodium	37.74236	31
Southern Sub-Saharan Africa	Diet high in processed meat	2.261404	Diet high in processed meat	2.698215	32
Southern Sub-Saharan Africa	Diet high in sugar-sweetened beverages	1.879474	Diet high in sugar-sweetened beverages	2.052585	33
Southern Sub-Saharan Africa	High temperature	0.24576	High temperature	0.747973	34
Southern Sub-Saharan Africa	Diet high in trans fatty acids	0.119827	Diet high in trans fatty acids	0.005571	35

Southern Sub-Saharan Africa	Alcohol use	-47.9496	Alcohol use	-38.1256	36
Tropical Latin America	Metabolic risks	2294.935	Metabolic risks	1195.814	1
Tropical Latin America	Behavioral risks	1795.734	Behavioral risks	757.9223	2
Tropical Latin America	High systolic blood pressure	1428.953	High systolic blood pressure	733.2898	3
Tropical Latin America	Dietary risks	1259.114	High LDL cholesterol	630.1504	4
Tropical Latin America	High LDL cholesterol	1185.541	Dietary risks	563.0514	5
Tropical Latin America	Tobacco	982.9511	Tobacco	325.8044	6
Tropical Latin America	Environmental/occupational risks	891.9395	Environmental/occupational risks	306.961	7
Tropical Latin America	Smoking	844.6078	Smoking	275.7243	8
Tropical Latin America	Air pollution	672.6778	High body-mass index	269.3659	9
Tropical Latin America	Particulate matter pollution	672.6778	High fasting plasma glucose	209.3138	10
Tropical Latin America	Kidney dysfunction	417.6971	Kidney dysfunction	203.0813	11
Tropical Latin America	Diet low in whole grains	384.1082	Air pollution	194.7407	12
Tropical Latin America	Diet low in seafood omega-3 fatty acids	364.285	Particulate matter pollution	194.7407	13
Tropical Latin America	High body-mass index	355.1854	Diet low in whole grains	171.602	14
Tropical Latin America	Ambient particulate matter pollution	348.994	Ambient particulate matter pollution	162.6466	15
Tropical Latin America	High fasting plasma glucose	323.8411	Diet low in seafood omega-3 fatty acids	151.3853	16
Tropical Latin America	Household air pollution from solid fuels	323.4672	Diet high in red meat	104.3056	17
Tropical Latin America	Diet low in fiber	256.8797	Diet low in fiber	93.87728	18
Tropical Latin America	Secondhand smoke	203.6162	Diet high in sodium	89.51468	19
Tropical Latin America	Diet high in sodium	191.6987	Other environmental risks	81.52536	20
Tropical Latin America	Other environmental risks	182.3504	Lead exposure	81.52536	21
Tropical Latin America	Lead exposure	182.3504	Diet low in vegetables	67.04375	22
Tropical Latin America	Diet low in vegetables	149.7837	Secondhand smoke	63.91165	23
Tropical Latin America	Diet low in fruits	132.0956	Low temperature	50.85986	24
Tropical Latin America	Diet high in red meat	129.2004	Non-optimal temperature	50.20951	25
Tropical Latin America	Diet low in nuts and seeds	118.8464	Diet low in polyunsaturated fatty acids	44.67858	26
Tropical Latin America	Low temperature	112.874	Diet low in fruits	39.95618	27
Tropical Latin America	Non-optimal temperature	108.9857	Low physical activity	36.03258	28
Tropical Latin America	Diet low in legumes	78.61427	Household air pollution from solid fuels	32.04051	29
Tropical Latin America	Diet low in polyunsaturated fatty acids	75.69458	Diet low in legumes	18.67302	30
Tropical Latin America	Low physical activity	70.70786	Diet high in trans fatty acids	14.17392	31

Tropical Latin America	Diet high in trans fatty acids	55.00286	Diet low in nuts and seeds	11.38193	32
Tropical Latin America	Diet high in processed meat	3.98893	Diet high in processed meat	4.188685	33
Tropical Latin America	Diet high in sugar-sweetened beverages	3.570581	Diet high in sugar-sweetened beverages	3.0938	34
Tropical Latin America	High temperature	-3.87428	High temperature	-0.62412	35
Tropical Latin America	Alcohol use	-68.0805	Alcohol use	-40.9885	36
Western Europe	Metabolic risks	2283.831	Metabolic risks	675.1254	1
Western Europe	Behavioral risks	1694.181	Behavioral risks	468.1711	2
Western Europe	High systolic blood pressure	1638.495	High systolic blood pressure	432.7141	3
Western Europe	Dietary risks	1295.98	Dietary risks	379.4525	4
Western Europe	High LDL cholesterol	1140.847	High LDL cholesterol	329.8019	5
Western Europe	Tobacco	833.1012	Tobacco	189.4449	6
Western Europe	Environmental/occupational risks	748.0867	Smoking	169.4516	7
Western Europe	Smoking	742.1905	Environmental/occupational risks	153.0323	8
Western Europe	Air pollution	532.6358	Diet low in whole grains	136.2731	9
Western Europe	Particulate matter pollution	532.6358	High body-mass index	130.7014	10
Western Europe	Ambient particulate matter pollution	530.4767	High fasting plasma glucose	114.039	11
Western Europe	Diet low in whole grains	410.5306	Kidney dysfunction	111.9975	12
Western Europe	Kidney dysfunction	364.0144	Air pollution	79.83942	13
Western Europe	High body-mass index	342.3064	Particulate matter pollution	79.83942	14
Western Europe	High fasting plasma glucose	268.0846	Ambient particulate matter pollution	79.78995	15
Western Europe	Diet low in nuts and seeds	228.0974	Diet low in polyunsaturated fatty acids	63.84427	16
Western Europe	Diet low in polyunsaturated fatty acids	225.1264	Diet low in legumes	53.61257	17
Western Europe	Diet low in legumes	188.8053	Non-optimal temperature	53.59767	18
Western Europe	Diet low in fiber	184.9558	Diet low in fiber	52.91967	19
Western Europe	Non-optimal temperature	175.9581	Diet low in nuts and seeds	52.72296	20
Western Europe	Diet low in seafood omega-3 fatty acids	174.9392	Low temperature	52.42165	21
Western Europe	Low temperature	173.4289	Diet high in red meat	45.7142	22
Western Europe	Diet high in red meat	154.9368	Diet high in processed meat	42.9348	23
Western Europe	Secondhand smoke	141.4845	Diet high in sodium	37.40879	24
Western Europe	Diet low in fruits	130.0558	Diet low in seafood omega-3 fatty acids	37.0164	25
Western Europe	Diet high in processed meat	122.8364	Diet low in fruits	34.23989	26
Western Europe	Diet high in sodium	112.9534	Other environmental risks	29.37394	27

Western Europe	Other environmental risks	98.1655	Lead exposure	29.37394	28
Western Europe	Lead exposure	98.1655	Secondhand smoke	28.26718	29
Western Europe	Diet low in vegetables	86.43871	Diet low in vegetables	25.27255	30
Western Europe	Low physical activity	71.61222	Low physical activity	20.21367	31
Western Europe	Diet high in trans fatty acids	51.23477	Diet high in trans fatty acids	6.908032	32
Western Europe	Diet high in sugar-sweetened beverages	3.960595	Diet high in sugar-sweetened beverages	1.574075	33
Western Europe	High temperature	2.73138	High temperature	1.271697	34
Western Europe	Household air pollution from solid fuels	2.124935	Household air pollution from solid fuels	0.047826	35
Western Europe	Alcohol use	-102.427	Alcohol use	-38.1356	36
Western Sub-Saharan Africa	Metabolic risks	1496.789	Metabolic risks	1590.939	1
Western Sub-Saharan Africa	Environmental/occupational risks	1044.698	High systolic blood pressure	1148.216	2
Western Sub-Saharan Africa	Behavioral risks	998.2212	Environmental/occupational risks	983.295	3
Western Sub-Saharan Africa	High systolic blood pressure	982.6155	Air pollution	880.1885	4
Western Sub-Saharan Africa	Air pollution	949.4367	Particulate matter pollution	880.1885	5
Western Sub-Saharan Africa	Particulate matter pollution	949.4367	Behavioral risks	877.5177	6
Western Sub-Saharan Africa	Dietary risks	914.0487	Dietary risks	800.6306	7
Western Sub-Saharan Africa	Household air pollution from solid fuels	718.0863	Household air pollution from solid fuels	600.1521	8
Western Sub-Saharan Africa	High LDL cholesterol	578.3342	High LDL cholesterol	567.2503	9
Western Sub-Saharan Africa	Kidney dysfunction	341.6366	Kidney dysfunction	337.5842	10
Western Sub-Saharan Africa	Diet low in whole grains	300.7041	Ambient particulate matter pollution	279.932	11
Western Sub-Saharan Africa	Ambient particulate matter pollution	231.2556	Diet low in whole grains	257.7226	12
Western Sub-Saharan Africa	Diet low in seafood omega-3 fatty acids	224.1272	High fasting plasma glucose	237.28	13
Western Sub-Saharan Africa	Diet low in polyunsaturated fatty acids	217.6423	High body-mass index	220.4182	14
Western Sub-Saharan Africa	Diet low in fruits	174.6027	Diet low in polyunsaturated fatty acids	206.5058	15
Western Sub-Saharan Africa	Tobacco	172.2279	Diet low in seafood omega-3 fatty acids	171.1389	16
Western Sub-Saharan Africa	High fasting plasma glucose	161.9974	Diet low in fruits	158.6679	17
Western Sub-Saharan Africa	High body-mass index	133.6523	Tobacco	146.4608	18
Western Sub-Saharan Africa	Smoking	132.823	Lead exposure	135.5508	19
Western Sub-Saharan Africa	Lead exposure	127.9615	Other environmental risks	135.5508	20
Western Sub-Saharan Africa	Other environmental risks	127.9615	Smoking	112.2063	21
Western Sub-Saharan Africa	Diet low in legumes	114.946	Diet low in vegetables	95.62648	22
Western Sub-Saharan Africa	Diet low in vegetables	107.5693	Diet low in legumes	81.99184	23

Western Sub-Saharan Africa	Diet low in nuts and seeds	67.79416	Diet high in sodium	66.5436	24
Western Sub-Saharan Africa	Diet low in fiber	63.47259	Non-optimal temperature	54.01446	25
Western Sub-Saharan Africa	Diet high in sodium	60.84847	Secondhand smoke	37.11421	26
Western Sub-Saharan Africa	Non-optimal temperature	51.63484	High temperature	36.0118	27
Western Sub-Saharan Africa	Secondhand smoke	43.18162	Low physical activity	26.9333	28
Western Sub-Saharan Africa	Low temperature	32.03325	Diet low in fiber	23.06209	29
Western Sub-Saharan Africa	Low physical activity	28.18446	Diet low in nuts and seeds	21.11562	30
Western Sub-Saharan Africa	High temperature	20.19468	Low temperature	18.49225	31
Western Sub-Saharan Africa	Diet high in processed meat	7.824898	Diet high in processed meat	9.737859	32
Western Sub-Saharan Africa	Diet high in trans fatty acids	1.110456	Diet high in red meat	2.62959	33
Western Sub-Saharan Africa	Diet high in red meat	0.763711	Diet high in sugar-sweetened beverages	0.736513	34
Western Sub-Saharan Africa	Diet high in sugar-sweetened beverages	0.340129	Diet high in trans fatty acids	0.501556	35
Western Sub-Saharan Africa	Alcohol use	-40.0957	Alcohol use	-37.5628	36

Table S7 Forecast case number and ASR for incidence, prevalence, mortality, and DALYs of IHD to 2045 globally.

Year	Both		Male		Female	
	Case number (95% CI)	ASR (95% CI)	Case number (95% CI)	ASR (95% CI)	Case number (95% CI)	ASR (95% CI)
**Prevalence**						
2022	265215841.7 (257108234.9-273323448.5)	2963.05 (2872.43-3053.67)	150854139.4 (146533771-155174507.8)	3609.63 (3558.59-3660.67)	114361702.3 (110574463.9-118148940.7)	2386.66 (2348.67-2424.64)
2023	273607311.7 (264093038.1-283121585.2)	2976.28 (2872.75-3079.81)	154690179.6 (149615616.5-159764742.7)	3604.7 (3528.04-3681.37)	118917132.1 (114477421.6-123356842.5)	2415.06 (2358.69-2471.43)
2024	282403094.1 (270680751.2-294125437.1)	2989.93 (2865.79-3114.08)	158687278.7 (152423074.9-164951482.6)	3600.09 (3490.1-3710.07)	123715815.4 (118257676.3-129173954.5)	2443.74 (2362.95-2524.52)
2025	291519955.1 (276793044.1-306246866.2)	3004.2 (2852.41-3156)	162787263.3 (154914655.7-170659870.8)	3595.87 (3446.96-3744.78)	128732691.9 (121878388.3-135586995.4)	2472.98 (2363.03-2582.94)
2026	300874191.9 (282384381.4-319364002.4)	3019.24 (2833.67-3204.81)	166935156.9 (157068048.5-176802265.2)	3592.09 (3399.6-3784.57)	133939035 (125316332.9-142561737.1)	2503.03 (2359.71-2646.36)
2027	310352121.2 (287378506.7-333325735.7)	3034.83 (2810.16-3259.5)	171078224.3 (158859147.9-183297300.7)	3588.67 (3348.45-3828.9)	139273896.9 (128519358.7-150028435.1)	2533.51 (2352.87-2714.14)
2028	320085768.5 (291936306.1-348235230.9)	3050.78 (2782.47-3319.09)	175288962.1 (160384225.1-190193699.2)	3585.36 (3293.79-3876.92)	144796806.4 (131552081.1-158041531.7)	2564.15 (2342.58-2785.72)
2029	330218880.9 (296197634-364240127.8)	3067.16 (2751.16-3383.17)	179640411.4 (161722275.5-197558547.2)	3582.17 (3236.03-3928.3)	150578469.5 (134475358.4-166681580.5)	2595 (2329.03-2860.97)
2030	340656142.4 (300063442.4-381248842.5)	3084.21 (2716.7-3451.72)	184070067.5 (162817470.5-205322664.5)	3579.21 (3175.48-3982.93)	156586074.9 (137245971.9-175926177.9)	2626.33 (2312.53-2940.13)
2031	351308529.9 (303428926.3-399188133.5)	3102.11 (2679.34-3524.87)	188524301 (163616390.2-213432211.7)	3576.56 (3112.31-4040.82)	162784229 (139812536.2-185755921.8)	2658.35 (2293.23-3023.48)
2032	362052260.4 (306158922.9-417945598)	3120.73 (2638.99-3602.46)	192953050.2 (164068710.2-221837390.2)	3574.31 (3046.59-4102.02)	169099210.2 (142090212.7-196108207.8)	2690.72 (2270.74-3110.69)
2033	372980924.8 (308326498.3-437635351.3)	3139.75 (2595.55-3683.94)	197407508 (164222849.9-230592166.1)	3572 (2978.19-4165.8)	175573416.8 (144103648.4-207043185.2)	2723.1 (2244.9-3201.29)
2034	384238971 (310029637-458448305)	3159.14 (2549.1-3769.19)	201960379.5 (164136296.4-239784462.5)	3569.57 (2907.25-4231.89)	182278591.5 (145893340.6-218663842.5)	2755.45 (2215.72-3295.17)
2035	395750461.2 (311171864.9-480329057.4)	3179.12 (2499.81-3858.43)	206563150.9 (163760033.8-249366268)	3567.09 (2833.92-4300.26)	189187310.3 (147411831.1-230962789.4)	2788.01 (2183.4-3392.62)

2036	407439220.5 (311651527.2-503226913.9)	3199.91 (2447.78-3952.04)	211172621.6 (163047531.8-259297711.4)	3564.72 (2758.37-4371.08)	196266599 (148603995.4-243929202.5)	2821.01 (2148.04-3493.98)
2037	419194018.1 (311342605.1-527045431.1)	3221.46 (2392.83-4050.09)	215750152.3 (161957084.2-269543220.4)	3562.63 (2680.68-4444.58)	203443865.8 (149385520.9-257502210.7)	2854.13 (2109.31-3598.95)
2038	431096375.9 (310288539.8-551904212)	3243.39 (2334.72-4152.06)	220335288.2 (160518534.2-280152042.2)	3560.29 (2600.64-4519.94)	210761087.7 (149770005.6-271752169.8)	2887.04 (2067.03-3707.05)
2039	443283213.6 (308557370.8-578009056.5)	3265.63 (2273.41-4257.85)	224991950.8 (158772250.3-291211651.3)	3557.54 (2518.27-4596.82)	218291262.8 (149785120.5-286797405.2)	2919.67 (2021.19-3818.15)
2040	455695617 (306063884.4-605327349.5)	3288.38 (2208.96-4367.8)	229680818.7 (156678336.6-302683300.8)	3554.46 (2433.71-4675.2)	226014798.3 (149385547.8-302644048.7)	2952.27 (1971.98-3932.57)
2041	468282537 (302722709-633842364.9)	3311.99 (2141.45-4482.52)	234373675.6 (154202902.4-314544448.8)	3551.28 (2347.16-4755.41)	233908861.4 (148519806.6-319297916.2)	2985.15 (1919.53-4050.78)
2042	480944159.5 (298417772.5-663470546.4)	3336.44 (2070.67-4602.2)	239042804.6 (151312805.3-326772803.8)	3548.24 (2258.73-4837.76)	241901354.9 (147104967.2-336697742.6)	3017.99 (1863.54-4172.43)
2043	493740032.2 (293158448.7-694321615.8)	3361.32 (1996.32-4726.31)	243711217.2 (148019233.5-339403200.8)	3544.76 (2168.2-4921.33)	250028815.1 (145139215.1-354918415)	3050.44 (1803.87-4297.01)
2044	506788651 (286974671.8-726602630.1)	3386.48 (1918.24-4854.72)	248427910.3 (144343204.3-352512616.3)	3540.6 (2075.55-5005.65)	258360740.7 (142631467.5-374090013.8)	3082.4 (1740.5-4424.3)
2045	520045566.8 (279786629.2-760304504.4)	3412.12 (1836.42-4987.82)	253159705.7 (140251149.1-366068262.3)	3535.77 (1980.9-5090.64)	266885861.1 (139535480.1-394236242.1)	3114.1 (1673.59-4554.62)
**Incidence**						
2022	33057315.1 (31402688.7-34711941.5)	372.53 (353.88-391.19)	18576845.9 (17695145.2-19458546.6)	447.61 (437.83-457.39)	14480469.2 (13707543.5-15253394.9)	302.91 (295.96-309.87)
2023	33781820.3 (31924663.2-35638977.3)	371.04 (350.63-391.44)	18861442.5 (17860958.4-19861926.7)	443.08 (428.88-457.28)	14920377.7 (14063704.8-15777050.6)	303.97 (294.52-313.43)
2024	34554657.9 (32381942.1-36727373.8)	369.65 (346.4-392.89)	19163966 (17975183.5-20352748.5)	438.67 (418.78-458.56)	15390691.9 (14406758.6-16374625.3)	305.09 (292.32-317.87)
2025	35355993.8 (32753117.7-37958869.9)	368.38 (341.25-395.5)	19473835.4 (18029462.5-20918208.2)	434.38 (407.93-460.84)	15882158.5 (14723655.2-17040661.7)	306.28 (289.56-323.01)
2026	36170292 (33028436.8-39312147.3)	367.23 (335.33-399.13)	19782952.9 (18022115.2-21543790.6)	430.22 (396.51-463.92)	16387339.2 (15006321.6-17768356.8)	307.55 (286.33-328.76)
2027	36984500.1 (33202678.8-40766321.4)	366.2 (328.75-403.66)	20085134.7 (17953596.6-22216672.8)	426.17 (384.65-467.69)	16899365.3 (15249082.1-18549648.5)	308.87 (282.7-335.05)

2028	37823016.9 (33304913.8-42341120)	365.29 (321.64-408.94)	20392195.9 (17839999.5-22944392.2)	422.22 (372.43-472.01)	17430821 (15464914.3-19396727.7)	310.25 (278.68-341.82)
2029	38706956.2 (33357015.2-44056897.2)	364.5 (314.1-414.9)	20714099.4 (17693002.5-23735196.4)	418.37 (359.89-476.84)	17992856.8 (15664012.8-20321700.8)	311.67 (274.32-349.03)
2030	39620283.1 (33345290.4-45895275.8)	363.84 (306.18-421.49)	21042179.9 (17506336.3-24578023.5)	414.61 (347.1-482.13)	18578103.2 (15838954.1-21317252.2)	313.16 (269.64-356.69)
2031	40549016.6 (33256906.4-47841126.7)	363.31 (297.92-428.69)	21369323.5 (17274533.2-25464113.9)	410.94 (334.06-487.83)	19179693 (15982373.3-22377012.8)	314.71 (264.64-364.79)
2032	41481059.4 (33080762.2-49881356.6)	362.91 (289.35-436.47)	21690613.1 (16993954.8-26387271.4)	407.37 (320.81-493.93)	19790446.3 (16086807.4-23494085.1)	316.31 (259.31-373.31)
2033	42435868.3 (32832394.2-52039342.4)	362.63 (280.47-444.79)	22015456.3 (16672861.9-27358050.7)	403.86 (307.36-500.37)	20420412 (16159532.3-24681291.8)	317.94 (253.66-382.21)
2034	43433182.8 (32524494.3-54341871.2)	362.47 (271.31-453.63)	22353227.2 (16317888.2-28388566.2)	400.43 (293.74-507.12)	21079955.6 (16206606.2-25953305)	319.59 (247.7-391.48)
2035	44458220.6 (32141513.7-56774927.4)	362.44 (261.87-463.01)	22696627 (15922305.8-29470948.1)	397.05 (279.96-514.14)	21761593.6 (16219207.8-27303979.3)	321.27 (241.42-401.12)
2036	45495612.3 (31667833.5-59323391)	362.53 (252.15-472.91)	23038475.5 (15479701.8-30597249.1)	393.72 (266.03-521.41)	22457136.8 (16188131.8-28726141.9)	322.97 (234.82-411.12)
2037	46534129.9 (31091760.4-61976499.4)	362.75 (242.13-483.37)	23375082.7 (14986518.5-31763646.9)	390.45 (251.97-528.93)	23159047.2 (16105241.9-30212852.5)	324.7 (227.9-421.49)
2038	47591407.3 (30422783.8-64760030.7)	363.09 (231.82-494.37)	23714755.8 (14447654.5-32981857.1)	387.22 (237.78-536.66)	23876651.5 (15975129.4-31778173.6)	326.41 (220.65-432.17)
2039	48687872.9 (29669227.9-67706517.8)	363.55 (221.19-505.92)	24066812.5 (13867124.2-34266500.8)	384.03 (223.49-544.57)	24621060.4 (15802103.7-33440017)	328.12 (213.08-443.17)
2040	49811245.6 (28816885.7-70805605.5)	364.14 (210.25-518.03)	24425380.3 (13238945.3-35611815.3)	380.87 (209.1-552.65)	25385865.3 (15577940.4-35193790.2)	329.83 (205.19-454.48)
2041	50947271.5 (27850841.9-74043701.1)	364.85 (198.96-530.73)	24783962.6 (12557064.7-37010860.4)	377.72 (194.61-560.84)	26163308.9 (15293777.2-37032840.6)	331.53 (196.97-466.1)
2042	52087527.6 (26760368.7-77414686.6)	365.69 (187.31-544.08)	25140499.5 (11818168.3-38462830.7)	374.61 (180.04-569.18)	26947028.1 (14942200.4-38951855.9)	333.22 (188.42-478.03)
2043	53248989.5 (25549469.1-80948509.8)	366.66 (175.27-558.05)	25502475 (11023948-39981002)	371.52 (165.41-577.64)	27746514.5 (14525521.1-40967507.9)	334.88 (179.54-490.23)
2044	54451262	367.76	25878461.7	368.46	28572800.3	336.51

	(24220129.2-84682394.7)	(162.82-572.7)	(10174965.8-41581957.6)	(150.72-586.19)	(14045163.5-43100437.2)	(170.34-502.68)
2045	55682527.7	368.99	26263072.4	365.4	29419455.3	338.11
	(22757371-88607684.5)	(149.93-588.05)	(9264900.1-43261244.7)	(135.99-594.82)	(13492470.9-45346439.8)	(160.82-515.39)
**Mortality**						
2022	9227657.6	106.96	5124747.7	133.29	4102909.8	84.44
	(8747580-9707735.2)	(101.4-112.51)	(4824471.7-5425023.8)	(129.62-136.97)	(3923108.2-4282711.4)	(82.49-86.39)
2023	9411605.9	106	5229001.4	132.1	4182604.6	83.63
	(8847751.9-9975459.9)	(99.66-112.35)	(4884091.4-5573911.3)	(126.71-137.48)	(3963660.6-4401548.6)	(80.56-86.69)
2024	9621091.1	105.08	5346334.9	130.93	4274756.2	82.82
	(8923365.2-10318816.9)	(97.46-112.7)	(4929998.6-5762671.2)	(123.34-138.52)	(3993366.6-4556145.7)	(78.34-87.3)
2025	9839669.1	104.17	5467616.8	129.75	4372052.3	82.02
	(8961114-10718224.2)	(94.87-113.47)	(4953821.3-5981412.3)	(119.62-139.88)	(4007292.7-4736811.8)	(75.92-88.12)
2026	10057370.2	103.28	5588160.9	128.58	4469209.2	81.22
	(8955731-11159009.3)	(91.96-114.59)	(4953033.3-6223288.5)	(115.63-141.53)	(4002697.7-4935720.8)	(73.34-89.11)
2027	10270386.5	102.44	5706269	127.47	4564117.5	80.45
	(8907230.4-11633542.6)	(88.84-116.04)	(4928037.7-6484500.3)	(111.48-143.46)	(3979192.7-5149042.3)	(70.64-90.26)
2028	10498475	101.69	5832254.3	126.45	4666220.7	79.71
	(8835725.7-12161224.3)	(85.58-117.79)	(4889465.2-6775043.3)	(107.21-145.68)	(3946260.5-5386181)	(67.85-91.57)
2029	10751760.8	100.99	5970642.1	125.46	4781118.7	79
	(8749686-12753835.6)	(82.18-119.8)	(4841256.8-7100027.4)	(102.82-148.1)	(3908429.2-5653808.2)	(64.99-93.01)
2030	11018485.3	100.35	6114542	124.49	4903943.3	78.32
	(8637939.4-13399031.1)	(78.66-122.04)	(4777161.4-7451922.6)	(98.29-150.69)	(3860778-5947108.5)	(62.07-94.57)
2031	11290541.9	99.75	6260683.9	123.55	5029858	77.65
	(8492493.5-14088590.2)	(75.03-124.48)	(4693941.5-7827426.2)	(93.65-153.45)	(3798552-6261164)	(59.08-96.23)
2032	11564485.6	99.24	6407714.2	122.67	5156771.5	77.01
	(8309316.3-14819655)	(71.3-127.18)	(4589948-8225480.3)	(88.93-156.41)	(3719368.3-6594174.6)	(56.02-97.99)
2033	11856320.7	98.82	6563840.1	121.88	5292480.6	76.41
	(8098873.6-15613767.9)	(67.49-130.14)	(4470571.6-8657108.6)	(84.16-159.6)	(3628302-6956659.2)	(52.93-99.88)
2034	12174256.5	98.47	6732623.5	121.13	5441633	75.84
	(7863404.9-16485108)	(63.59-133.35)	(4336552.3-9128694.8)	(79.3-162.95)	(3526852.7-7356413.3)	(49.81-101.88)
2035	12507101	98.19	6907787.3	120.38	5599313.7	75.3
	(7591365.1-17422836.8)	(59.58-136.79)	(4181695.7-9633878.9)	(74.36-166.41)	(3409669.5-7788957.9)	(46.64-103.96)
2036	12846495.8	97.96	7086466.3	119.67	5760029.5	74.76

	(7273904.6-18419087)	(55.45-140.47)	(4002378.5-10170554.1)	(69.33-170)	(3271526.1-8248532.9)	(43.41-106.1)
2037	13189359.1<br>(6905971.5-19472746.8)	97.83<br>(51.2-144.45)	7267496.2<br>(3796192.4-10738800)	119.01<br>(64.25-173.77)	5921862.9<br>(3109779.1-8733946.8)	74.23<br>(40.15-108.32)
2038	13551374.8<br>(6492719-20610030.6)	97.79<br>(46.83-148.75)	7458313<br>(3565366.2-11351259.8)	118.41<br>(59.1-177.73)	6093061.8<br>(2927352.8-9258770.8)	73.75<br>(36.86-110.65)
2039	13939892.8<br>(6031843.6-21847942.1)	97.84<br>(42.31-153.37)	7662001.1<br>(3308372.4-12015629.8)	117.83<br>(53.88-181.78)	6277891.8<br>(2723471.2-9832312.3)	73.3<br>(33.54-113.06)
2040	14343546.7<br>(5511562.9-23175530.5)	97.95<br>(37.61-158.29)	7872593.6<br>(3019117.1-12726070.1)	117.24<br>(48.58-185.9)	6470953<br>(2492445.7-10449460.3)	72.85<br>(30.17-115.52)
2041	14755727.5<br>(4923125.6-24588329.4)	98.12<br>(32.71-163.54)	8088407.7<br>(2693698.9-13483116.6)	116.66<br>(43.21-190.12)	6667319.8<br>(2229426.8-11105212.9)	72.39<br>(26.77-118.02)
2042	15175561.8<br>(4260195.7-26090927.8)	98.4<br>(27.59-169.22)	8309191.3<br>(2328702.8-14289679.7)	116.13<br>(37.77-194.49)	6866370.5<br>(1931492.8-11801248.1)	71.96<br>(23.33-120.59)
2043	15619065.9<br>(3521139.5-27717318.9)	98.81<br>(22.23-175.39)	8541602.7<br>(1922690.3-15160720.8)	115.64<br>(32.28-199)	7077463.3<br>(1598449.3-12556598.2)	71.57<br>(19.87-123.28)
2044	16092533.2<br>(2697168.4-29488864)	99.3<br>(16.6-182.02)	8788316.6<br>(1470959.7-16106222.2)	115.15<br>(26.71-203.58)	7304216.6<br>(1226208.7-13382641.8)	71.2<br>(16.38-126.03)
2045	16583932.6<br>(1775430.4-31395125.7)	99.86<br>(10.65-189.1)	9044032.8<br>(967166.6-17122488.9)	114.62<br>(21.08-208.16)	7539899.9<br>(808263.8-14272636.8)	70.82<br>(12.85-128.8)
**DALYs (disability-adjusted life years)**						
2022	193131894.7<br>(182782090.8-203481698.7)	2853.68<br>(2777.85-2929.5)	118073489<br>(111162454.9-124984523.2)	2187.61<br>(2070.47-2304.76)	75058405.7<br>(71619635.9-78497175.5)	1574.84<br>(1539.26-1610.43)
2023	196731578.5<br>(184710513.7-208752643.4)	2835.72<br>(2723.13-2948.32)	120233529.4<br>(112334936.5-128132122.3)	2173.49<br>(2040.72-2306.25)	76498049.1<br>(72375577.2-80620521.1)	1563.32<br>(1506.96-1619.68)
2024	200596025.8<br>(185914223.8-215277827.8)	2818.28<br>(2658.55-2978.01)	122525889.4<br>(113056923.9-131994855)	2160.02<br>(2001.93-2318.12)	78070136.3<br>(72857299.9-83282972.8)	1552.1<br>(1469.43-1634.76)
2025	204552733.8<br>(186270786.3-222834681.4)	2800.69<br>(2586.43-3014.94)	124850614.3<br>(113243329.5-136457899.1)	2146.84<br>(1954.92-2338.77)	79702119.5<br>(73027456.8-86376782.3)	1540.98<br>(1428.12-1653.84)
2026	208516755.9<br>(185783461.8-231250050)	2783.48<br>(2508.77-3058.19)	127178189.3<br>(112910054.5-141446324.1)	2134.17<br>(1901.4-2366.94)	81338566.6<br>(72873407.3-89803725.9)	1529.84<br>(1383.65-1676.03)
2027	212479329.7<br>(184516159.9-240442499.6)	2767.58<br>(2427.19-3107.97)	129512626.5<br>(112104517.2-146920735.8)	2122.7<br>(1843.21-2402.19)	82966703.2<br>(72411642.7-93521763.7)	1519.1<br>(1336.83-1701.37)
2028	216638722	2753.19	131953599.1	2112.79	84685123	1509.1

	(182704733-250572711)	(2342.6-3163.78)	(110953542.1-152953656.1)	(1781.67-2443.91)	(71751191-97619055)	(1288.36-1729.84)
2029	221061397.8	2739.31	134522492.2	2103.91	86538905.7	1499.53
	(180422489-261700306.6)	(2254.58-3224.04)	(109489380.9-159555603.4)	(1716.91-2490.9)	(70933108.1-102144703.2)	(1238.22-1760.84)
2030	225600199.8	2725.33	137134238.4	2095.66	88465961.4	1490.11
	(177541575.9-273658823.6)	(2162.95-3287.72)	(107642408.8-166626067.9)	(1648.96-2542.37)	(69899167.1-107032755.7)	(1186.35-1793.86)
2031	230191680	2711.78	139775343.4	2088.24	90416336.6	1480.66
	(173996648.2-286386711.8)	(2068.35-3355.22)	(105396169.8-174154516.9)	(1578.12-2598.36)	(68600478.4-112232194.9)	(1132.75-1828.56)
2032	234839487.6	2699.44	142457972.7	2082.29	92381514.9	1471.59
	(169771236.4-299907738.9)	(1971.48-3427.4)	(102750159.1-182165786.3)	(1504.94-2659.64)	(67021077.2-117741952.6)	(1077.8-1865.37)
2033	239707305.5	2688.34	145261399.8	2078.07	94445905.7	1463.24
	(164972201.4-314442409.6)	(1872.61-3504.08)	(99755749.7-190767050)	(1429.7-2726.44)	(65216451.7-123675359.6)	(1021.91-1904.56)
2034	244845556.7	2677.55	148200806.6	2075.04	96644750.2	1455.23
	(159592806.4-330098307)	(1771.22-3583.88)	(96401255.7-200000357.4)	(1351.97-2798.12)	(63191550.7-130097949.6)	(964.9-1945.55)
2035	250115656.3	2666.5	151200439.3	2072.79	98915216.9	1447.18
	(153489515.2-346741797.3)	(1667.1-3665.9)	(92608649.4-209792229.3)	(1271.35-2874.22)	(60880865.8-136949568)	(906.6-1987.76)
2036	255464011.8	2655.7	154254749.4	2071.54	101209262.4	1438.96
	(146578043.3-364349980.3)	(1560.67-3750.74)	(88345510.7-220163988.2)	(1187.81-2955.26)	(58232532.6-144185992.2)	(846.99-2030.92)
2037	260908576.3	2645.86	157381479.5	2071.93	103527096.8	1430.99
	(138815782.4-383001370.2)	(1452.35-3839.37)	(83590111.2-231172847.8)	(1101.46-3042.39)	(55225671.2-151828522.4)	(786.38-2075.61)
2038	266612749	2636.86	160652566.9	2074.19	105960182.1	1423.66
	(130240883.3-402984614.7)	(1342.2-3931.52)	(78351238-242953895.8)	(1012.19-3136.19)	(51889645.3-160030718.9)	(725.04-2122.28)
2039	272616672.9	2627.81	164078617.3	2077.77	108538055.6	1416.55
	(120790590.6-424442755.3)	(1229.85-4025.76)	(72587413.5-255569821.2)	(919.39-3236.15)	(48203177.1-168872934.1)	(662.81-2170.28)
2040	278784575.2	2618.18	167589618.6	2082.26	111194956.6	1409.23
	(110309925-447259225.5)	(1115.21-4121.16)	(66213913-268965324.2)	(822.48-3342.03)	(44096012-178293901.2)	(599.56-2218.9)
2041	285083344.1	2608.54	171192020.5	2087.98	113891323.6	1401.68
	(98693077.2-471473611)	(998.59-4218.48)	(59178751.9-283205289)	(721.18-3454.77)	(39514325.3-188268322)	(535.33-2268.03)
2042	291549835.7	2599.5	174912318.3	2095.57	116637517.4	1394.31
	(85853124-497246547.4)	(880.25-4318.76)	(51429907.6-298394729)	(615.16-3575.97)	(34423216.4-198851818.4)	(470.31-2318.31)
2043	298349732.8	2590.92	178815214.3	2105.26	119534518.5	1387.49
	(71739270.6-524965349.3)	(760.22-4421.63)	(42928094-314707488.9)	(504.02-3706.56)	(28811176.6-210257860.4)	(404.68-2370.3)
2044	305515479.9	2581.96	182909162.8	2116.5	122606317.1	1380.78
	(56240631.9-554837156.7)	(638.37-4525.56)	(33603091.9-332244434.7)	(387.3-3846.29)	(22637540.1-222592721.9)	(338.34-2423.22)

2045	312912212.5<br>(39167740.5-586785694.8)	2572.19<br>(514.72-4629.65)	187133431<br>(23355156.4-350992631)	2128.9<br>(264.22-3995.2)	125778781.5<br>(15812584.1-235793063.8)	1373.74<br>(271.23-2476.24)
